# Substrate‐Affinitive P‐Type Azapolycyclic Photosensitizer for Chemoselective Nitrilization Under Mild Conditions

**DOI:** 10.1002/advs.75144

**Published:** 2026-04-07

**Authors:** Seongwoo Bae, Dongwook Kim, Jinwoo Kim

**Affiliations:** ^1^ Department of Chemistry Chungnam National University Daejeon South Korea; ^2^ Institute for Sciences of the Universe Chungnam National University Daejeon South Korea; ^3^ Center for Catalytic Hydrocarbon Functionalization Institute of Basic Science Daejeon South Korea

**Keywords:** hydrogen‐bonding interaction, nitrilization, photocatalysis, reductive photosensitizer, substrate affinity

## Abstract

Herein we report azapolycyclic quinoxalinoquinoxaline (QQ) photosensitizers featuring both tunable reducing ability and hydrogen‐bonding assisted substrate affinity. The **QQ‐Ar** photosensitizers are easily accessible on the gram scale and can be purified via vacuum sublimation, highlighting their potential for vapor‐phase deposition in semiconductor applications. Their strong excited‐state reduction potentials (*E*(**PC** ^**•+**^/**PC** ^*^) = −1.76 to −2.05 V vs SCE) arise from tunable ground‐state oxidation potentials modulated by the aryl substituent. Despite the high reducing ability, the formation of honeycomb hydrogen‐bonding network with carbonyl substates enables direct nitrilization of primary amides under mild conditions. A broad range of aryl, heteroaryl, olefinic, and alkyl amides, as well as pharmaceutical derivatives, were converted to nitriles with high functional group tolerance and scalability. Spectroscopic and computational studies support a mechanism involving oxidative quenching and selective hydrogen atom abstraction. This work establishes QQ photosensitizers as a practical and versatile platform for amide‐to‐nitrile conversions under mild conditions, with potential utility in both industrial and synthetic applications.

## Graphical abstract

We developed a series of quinoxalinoquinoxaline (QQ) photosensitizers featuring high and tunable excited‐state reduction potentials (*E*(**PC^•+^**/PC^*^) = –1.76 to –2.05 V vs. SCE). Their hydrogen‐bonding interaction with carbonyl substrates enables selective single‐electron transfer events for selective nitrilization of 1° amides, offering a tunable photoredox platform for mild functional group transformations.

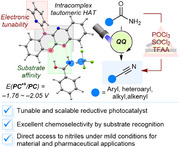

## 1 Introduction

**FIGURE 1:**
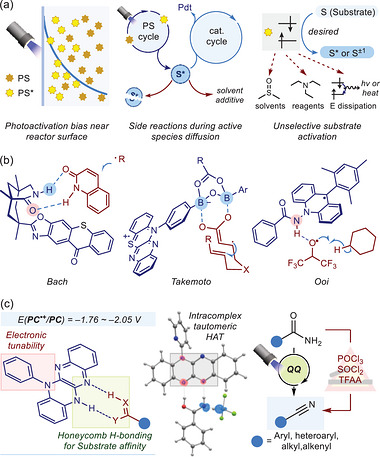
Design of quinoxalinoquinoxaline (QQ) photosensitizer for substrate‐affinitive transformation. (a) Spatial challenges of conventional photocatalystic cascade. (b) Substrate‐attractive photocatalysts for organic transformations. (c) This work: Highly reductive QQ photosensitizer with honeycomb H‐bonding network capable of chemoselective nitrilization.

The urgent challenges of climate change and energy depletion have called for significant efforts toward implementing sustainable photoenergy in industrial processes [1, 2]. In this context, photocatalytic organic synthesis has emerged as a powerful platform to access high‐value chemicals, leading to notable advances in fine chemical production [3, 4, 5, 6, 7]. These advances have been underpinned by the design of diverse photoactive scaffolds [8], as well as diverse strategies that integrate photocatalytic and conventional catalytic systems [9, 10, 11, 12, 13, 14, 15]. Despite these advances, the construction of efficient photocatalytic cascades remains challenging, particularly due to spatial constraints that markedly reduce catalytic performance (Figure 1a) [16, 17, 18]. Because light intensity decreases exponentially with depth in a reactor, photoactivation predominantly occurs near the surface, creating a depth‐dependent bias. As a result, reactive species generated by photoactivation must migrate over long distances to engage in subsequent catalytic cycles, rendering them susceptible to decomposition or undesired side reactions. In addition, excited photosensitizers may fail to encounter the substrate in time, instead dissipating their energy as heat or light or reacting with unintended species.

Within this framework, a range of photoactive organic scaffolds has been designed to introduce specific interactions with substrates, thereby enhancing chemo‐ and regioselectivity in photocatalytic transformations (Figure 1b for selected examples) [19]. As an early example, Hammond and Cole demonstrated that optically active chiral amides can control the stereochemical ring opening of cyclopropanes [20]. In the 1990s, Inoue and coworkers introduced chiral carboxylate photosensitizers and systematically investigated solvent and temperature effects [21, 22, 23]. Bach and co‐workers developed bifunctional xanthone photocatalysts that enabled stereoselective cyclization [24, 25, 26, 27, 28, 29] and deracemization [30, 31, 32, 33]. Krische et al. [34] and Sivaguru et al. [35] employed amide and urea moieties to facilitate hydrogen‐bonding interactions with substrates for stereoselective cyclization. More recently, Nanjo and Takemoto developed pyridine‐based organophotocatalyst for reductive C─Br bond cleavage [36, 37] and phenothiazine‐boronic acid hybrid catalyst for cooperative cyclizations [38, 39]. Ooi et al. developed acridinium [40, 41] and thiophenol [42] photosensitizers for direct single‐electron or hydrogen atom transfer. Mori reported an oxazaborolidine catalyst for enantio‐differentiating photocyclization [43]. In parallel, organometallic hybrid photocatalysts using Ni [44, 45], Pd [46, 47, 48, 49], Ru [50, 51], Ir [52, 53, 54, 55, 56], Au [57], and Rh [58, 59, 60] complexes have been extensively studied [61]. A notable Ir‐based ion‐pair photocatalyst reported by Knowles and Alexanian [62] represents a milestone for ion‐pair assisted intracluster electron transfer [63, 64, 65, 66].

Although diverse photocatalyst systems have been tailored to introduce specific substrate interactions, the availability of effective organic photosensitizers remains finite. Considering that organic photosensitizers have attracted considerable attention for their reducing capability compared to inorganic photocatalysts [67], we envisioned that the development of a tunable, highly‐reducing photosensitizer with specific substrate affinity would provide a versatile platform for chemoselective transformations initiated via an oxidative quenching mechanism. In this context, we herein describe a novel series of *N*‐monoaryl quinoxalinoquinoxaline (QQ) [68] azapolycycle photosensitizers (Figure 1c). While retaining the strong reducing ability of the conjugation‐disrupted framework (*E*(**PC^•+^**/PC^*^) = −1.76 to −2.05 V vs SCE) akin to thiazinoquinoxaline derivatives developed by Carreira and co‐workers [69, 70, 71, 72, 73], the auxiliary aryl moiety enables redox potential tuning by modulating the substituent. Unlike acridinium‐based acceptors, the QQ scaffold acts as a reductant via internal electron transfer from the unconjugated amine to the quinoxaline core, followed by external single electron transfer (SET). A free imidamide moiety on the opposite side forms a honeycomb hydrogen‐bonding network with carboxylate substrates, enabling intracomplex hydrogen‐atom transfer (HAT) via tautomerization to minimize side reactions and confer high functional group selectivity. We also demonstrate its application to the direct nitrilization of primary amides under mild conditions, substantiating the substrate‐affinitive mechanism and underscoring its pharmaceutical utility.

## 2 Results and Discussion

### 2.1 Catalyst Preparation

**FIGURE 2:**
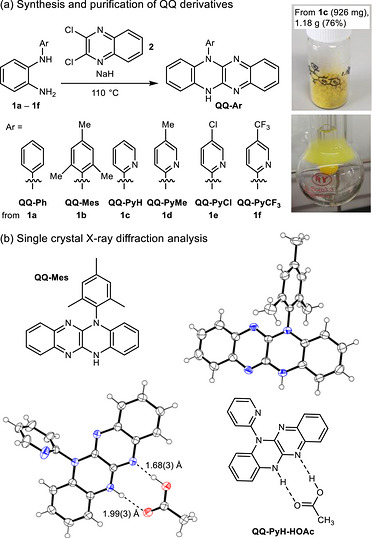
(a) Preparation of **QQ‐Ar** catalyst derivatives. (right up) Gram scale synthesis of **QQ‐PyH**. (right bottom) **QQ‐PyH** sublimate after vacuum sublimation. (b) Single crystal X‐ray diffraction structure of **QQ‐Mes** and **QQ‐Py‐HOAc** with 50% ellipsoid probability.

At the onset of this study, a series of *N*‐aryl QQ derivatives (**QQ‐Ar**) was synthesized from the corresponding *N*‐monoaryl phenylenediamines (**1**) and 2,3‐dichloroquinoxaline (**2**) through a straightforward S_N_Ar process (Figure 2a). Precursor **1** was effectively prepared from 2‐fluoronitrobenzene and the corresponding anilines (see Supporting Information) to prevent the formation of diarylphenylenediamines, which often form as side products when o‐phenylenediamine is used as the starting material. Due to low solubility of the QQ‐Ar derivatives (except for **QQ‐Mes**), they required an acid additive (0.2% formic acid) for column chromatographic purification. A gram‐scale synthesis of **QQ‐PyH** (1.18 g, 62%) was performed using trituration in lieu of column chromatography. Additional purification by vacuum sublimation at 200°C–220°C afforded yellow sublimate of even higher purity, highlighting its potential for vapor‐deposition in various industrial applications including semiconductor processing.

Single‐crystal X‐ray diffraction analysis of **QQ‐Mes** and **QQ‐PyH** confirmed the expected structures (Figure 2b). The QQ polycycles displayed outer C─N bond lengths of 1.378–1.418 Å, close to *sp* ^2^ C─N bond character. The structure of **QQ‐PyH** was obtained as its acetic acid adduct, where the hydrogen‐bonding interactions formed a honeycomb network as designed, with clear H‐bond lengths of 1.68(3) and 1.99(3) Å. Notably, the nitrogen atom within the QQ polycycle is slightly basic, and its protonation under acidic conditions is known to dramatically change its optical properties [74].

### 2.2 Characterization of QQ Photosensitizer

**FIGURE 3:**
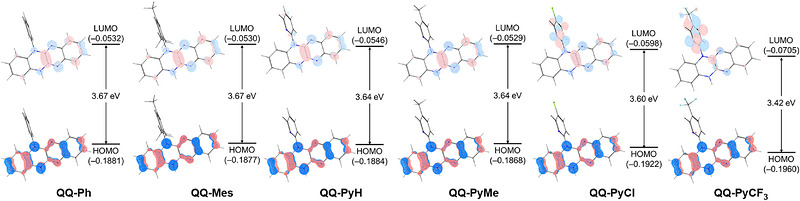
Kohn–Sham frontier orbital diagram of **QQ‐Ar** derivatives at the uB3LYP‐D3/def2‐TZVP level of theory. Geometry optimization was performed under the same level of theory. Isovalue = 0.5. Orbital energies are given in Hartrees.

With established synthetic access and verified structures in hand, we next analyzed the optical and redox properties of **QQ‐Ar** derivatives. Density functional theory (DFT) calculations revealed that the HOMO is localized on the *N*‐aryl substituent side of QQ polycycle, with little variation across the series (Figure 3). The LUMO resides at the center of QQ core for electron‐neutral or electron‐rich substituents, indicating that the excitation corresponds to a π_QQ_‐π_QQ_^*^ transition. In contrast, for **QQ‐PyCl** and **QQ‐PyCF_3_**, the LUMO mixes with the π_Ar_^*^ of the *N*‐aryl group, lowering the LUMO energy. Accordingly, the HOMO‐LUMO gaps ranged from 3.67 to 3.42 eV. It should be noted that in **QQ‐PyCF_3_**, the LUMO+1 localized on the QQ core was more favorable for excitation (3.66 eV) than the π_QQ_‐π_QQ_^*^ transition due to better orbital overlapping (see Supporting Information for natural transition orbital analysis).

**FIGURE 4:**
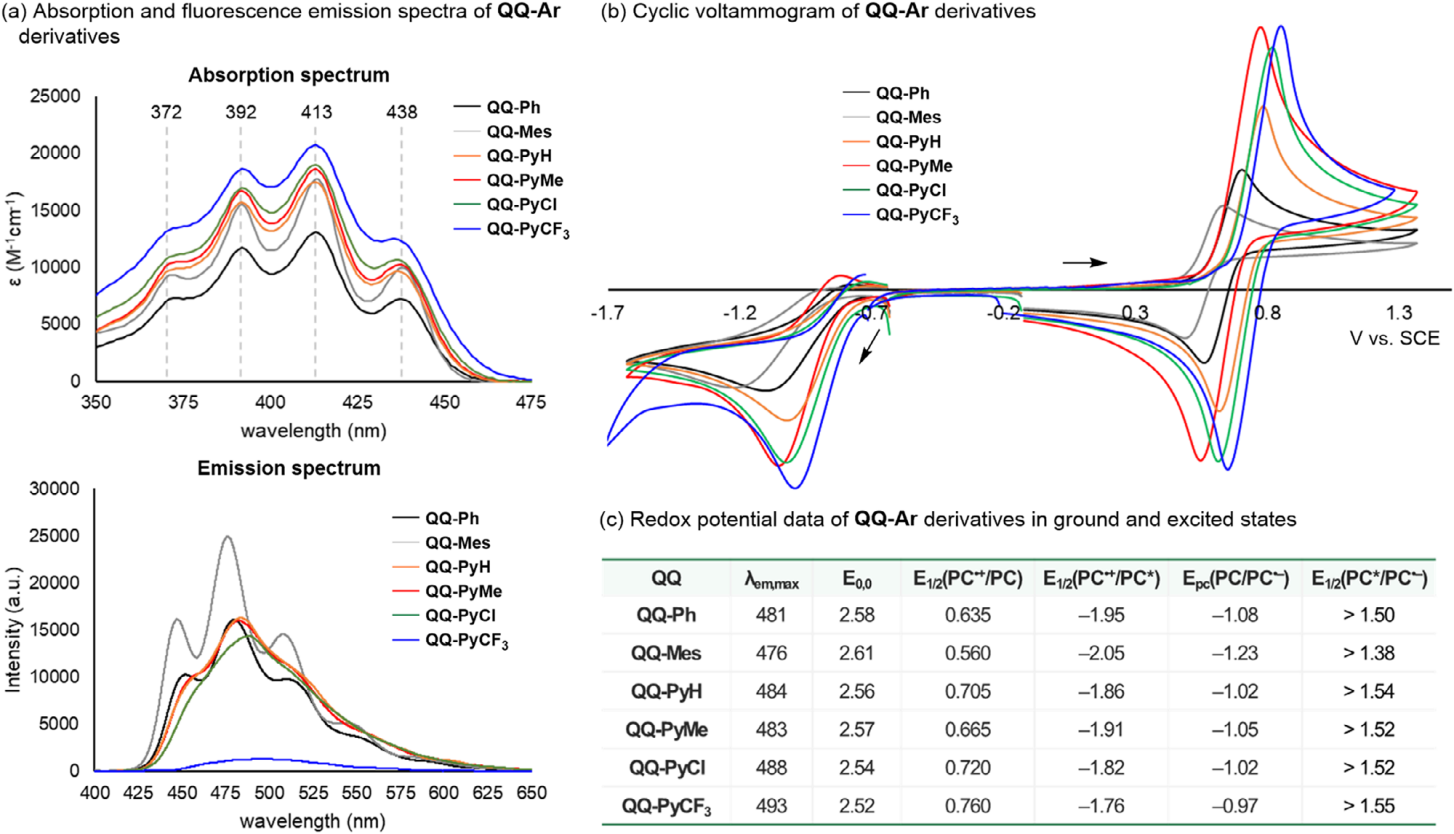
(a) UV–vis absorption and emission spectra of **QQ‐Ar** derivatives (20 µm in DMF). (b) Cyclic voltammograms of **QQ‐Ar** derivatives (3 mm in DMF, 800 mV/s, vs SCE). Oxidation (right) and reduction (left) waves were measured separately and overlaid. (c) Calculated ground‐ and excited‐state redox potentials of **QQ‐Ar** derivatives.

Experimentally, the UV–vis absorption spectra of **QQ‐Ar** derivatives consistently showed λ_abs,max_ = 413 nm with a vibronic coupling constant of ∼1350 cm^−1^ at room temperature (Figure 4a). In fluorescence emission spectra, electron‐deficient **QQ‐Ar** derivatives displayed a red‐shifted λ_em,max_, consistent with the calculated HOMO‐LUMO gaps, and also showed diminished emission intensity. Cyclic voltammetry of QQ‐Ar species exhibited highly reversible oxidation waves, with half‐ wave oxidation potentials *E*_1/2_(**QQ‐Ar^•+^**/**QQ‐Ar**) ranging from 0.560 V (**QQ‐Mes**) to 0.760 V (**QQ‐CF_3_**) vs SCE, reflecting the electron richness of the *N*‐aryl substituents (Figure 4b). In contrast, reduction signals were irreversible, with cathodic peak potentials (*E*_pc_) ranging from −1.23 V (**QQ‐Mes**) to ‒0.97 V (**QQ‐PyCF_3_**). From the emission maxima and ground‐state oxidation potentials, the excited‐state oxidation potentials *E*_1/2_(**QQ‐Ar^•+^**/QQ‐Ar^*^) were calculated to be −2.05 V (**QQ‐Mes**) to −1.76 V (**QQ‐PyCF_3_**), highlighting the strong reduction ability of **QQ‐Ar** derivatives (Figure 4c) [5, 8]. On the other hand, the excited‐state reduction potentials, estimated as a lower bound from *E*_pc_(**QQ‐Ar**/**QQ‐Ar^•‒^**) + *E*_0,0_, were greater than ∼1.50 V for all but **QQ‐Mes**.

### 2.3 Synthetic Utilization

Having established the tunable reducing ability of the **QQ‐Ar** photocatalysts, we next sought an appropriate catalytic reaction to demonstrate their substrate affinity. We envisioned that a honeycomb H‐bonding network formed with primary amide substrates would facilitate their direct conversion into nitrile products, via a tautomerization‐coupled HAT mechanism. Nitrile is a synthetically versatile functional group that can be readily converted into primary amines, unsymmetrical ketones, and *N*‐heterocycles [75, 76, 77]. It also serves as a key pharmaceutical motif as a metabolically robust bioisostere of carbonyl, hydroxyl, and halide groups [78, 79, 80], as found in fadrozole, finrozole, and tanaproget. However, conventional approaches to access nitriles from amides require sequential deprotonation and dehydration, which demand high activation energy and thus rely on corrosive reagents such as POCl_3_, SOCl_2_, or trifluoroacetic anhydride (TFAA) [81]. Therefore, developing a mild and direct catalytic access to nitriles is highly desirable in pharmaceutical and materials chemistry. Considering the versatile synthetic platforms using easily accessible amides [82, 83, 84, 85, 86], it would also provide a novel catalytic strategy for the strategic transformation of the robust amide bond, a recently highlighted synthetic handle [87].

Pleasingly, in the presence of CCl_3_Br as a primary electron acceptor and DMAP as an additive in DMF, **QQ‐PyH** efficiently transformed 4‐methoxybenzamide (**3a**) into 4‐methoxybenzonitrile (**4a**) in 95% isolation yield under visible light irradiation (Table 1, **4a)**. Other QQ‐based photocatalysts also exhibited comparable nitrilization reactivity (96, 91, 95, and 97% for **QQ‐Mes**, **QQ‐PyMe**, **QQ‐PyCl**, and **QQ‐PyCF_3_**, respectively, Table S4). In contrast, when Ir(ppy)_3_, which features a high reduction potential (*E*(**PC^•+^**/PC^*^) = −1.7 V vs SCE) but lacks substrate affinity, was employed in place of **QQ‐PyH**, significant side reactions were observed, affording only trace amount of **4a**. In fact, when Ir(ppy)_3_ was used under milder conditions (10 W, 1 h), **3a** was fully consumed, yet only a 61% yield of **4a** was obtained, suggesting rapid product decomposition under these photocatalytic conditions. Other aryl carboxamide substrates were generally well tolerated. 4‐Methoxybenzonitrile (**4a**) was isolated in 95% yield under the optimized conditions. Although 4‐methylbenzamide underwent quantitative nitrilization, the volatility of the product led to a lower isolated yield of **4b** (75%). Halogenated and sulfonyl derivates were converted to the corresponding nitriles in high yields (87, 92, and 87% for **4c**, **4d**, and **4e**, respectively), with the relatively lower yield of **4c** attributable to sublimation under high vacuum. Unprotected phenol and nitro groups were compatible, affording **4f** and **4** **g** in 97% and 93% yield, respectively. The dibenzylic position of 9*H*‐fluorene moiety well‐survived the reaction conditions, yielding 89% of the nitrile product (**4** **h**). An amide bearing a 2‐pyridyl substituent was also well tolerated, providing the corresponding nitrile **4i** in 91% yield. It should be noted that, despite the high reducing ability of the excited QQ species, nitrilization of the primary amide substrates generally exhibited broad functional group tolerance, attributable to substrate‐affinitive H‐bonding interactions.

**TABLE 1:** Selected examples of QQ‐Ar‐catalyzed direct nitrilization of primary amides.^a)^

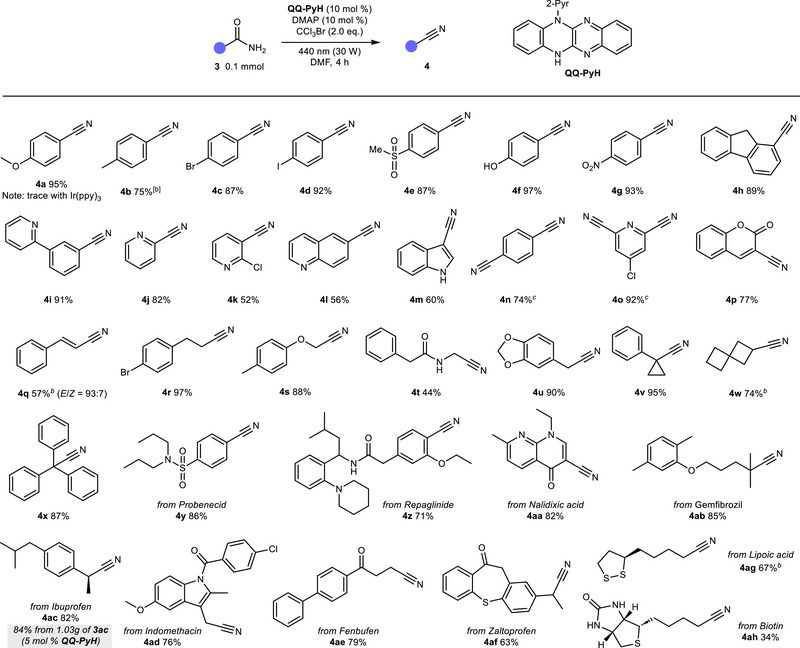

^a)^ Isolated yields are reported.

^b)^ 0.2 mmol scale.

^c)^ 4.0 eq. of CCl_3_Br was used.

Amides on *N*‐heterocyclic scaffolds such as pyridine and quinoline provided the corresponding nitriles in moderate to good yields (82, 52, and 56% for **4j**, **4k**, and **4l**, respectively). Notably, the unprotected indole substrate was also compatible, delivering **4m** in 60% yield. Terephthalamide was smoothly converted to terephthalonitrile (**4n**, 74%), showing that both amide groups within a single molecule can undergo nitrilization. Furthermore, pyridine‐2,6‐dicarboxamide furnished pyridine‐2,6‐dicarbonitrile (**4o**, 92%), a valuable precursor to tridentate pincer‐type ligands. Olefinic amides were also amenable to the present conditions. Chromene‐3‐ carboxamide underwent selective nitrilization without side reactions at either the lactone or β‐position of unsaturated carbonyl group (**4p**, 77%). (*E*)‐Cinnamamide was converted to cinnamonitrile (**4q**, 57%), with slight stereochemical erosion (*E*/*Z* = 93:7). Indeed, prolonged evacuation of **4q** under vacuum increased the relative proportion of the *Z* form, owing to greater loss of the major *E* isomer.

Alkyl amides also readily participated in the nitrilization with high functional group tolerance. For instance, 3‐(4‐bromophenyl)propanamide **3r** provided nitrile **4r** in 97% yield without benzylic side reactions. Amides bearing α‐protons adjacent to oxygen or nitrogen gave **4s** (88%) and **4t** (44%), with the internal amide in **3t** was left intact under the reaction conditions. A methylenedioxybenzene derivative (**3u**) underwent nitrilization in 90% yield without acetal deprotection. Cyclopropane and spiro[3,3]heptane substrates delivered the corresponding nitriles (95% and 74% for **4v** and **4w**, respectively) without ring opening. Even sterically demanding substrates such as 2,2,2‐triphenylacetamide afforded the corresponding trityl nitrile (**4x**) in high yield (87%).

To showcase the synthetic utility of the present catalyst system for the pharmaceutical industry, we examined the direct nitrilization of primary amides derived from bioactive carboxylic acids. Probenecid containing a sulfonamide moiety afforded **4y** in 86% yield, while a repaglinide nitrile derivative (**4z**) was obtained in 71% yield in the presence of tertiary amine and secondary amide. The primary amide obtained from nalidixic acid underwent smooth transformation without affecting the conjugated ketone, providing **4aa** in 82% yield. Alkyl amides from gemfibrozil and ibuprofen gave the nitriles in high yields (85% and 82% for **4ab** and **4ac**, respectively). Additional carbonyl groups in indomethacin and fenbufen were well tolerated (76% and 79% for **4ad** and **4ae**, respectively). On the other hand, zaltoprofen, bearing a thioether unit, also provided **4af** in 63% yield. Encouraged by this observation, we next examined other sulfur‐containing substrates. Lipoamide furnished liponitrile **4ag** in 67% yield, although the product tended to polymerize upon storage. In addition, biotin‐derived amide also underwent nitrilization, affording **4ah** in 34% yield after column purification. Importantly, 5 mol % of **QQ‐PyH** converted 1.03 g of ibuprofen derivative **3ac** (5.00 mmol) into **4ac** in 84% yield, underscoring the gram‐scale utility of this protocol.

### 2.4 Mechanistic Studies

**FIGURE 5:**
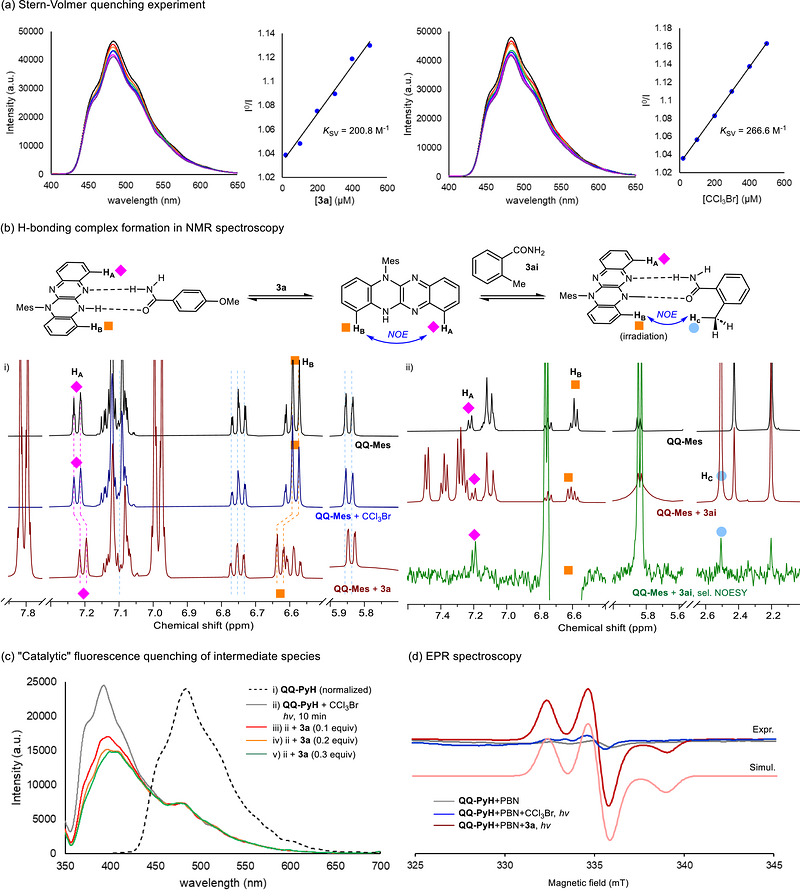
Spectroscopic analyses for mechanistic investigation. (a) Stern–Volmer quenching plots of **QQ‐PyH** (20 µm) wth **3a** and CCl_3_Br as quenchers. (b) (left) ^1^H NMR chemical shifts of **QQ‐Mes** (4.0 mm) upon addition of **3a** (20 mm). (right) Selective ^1^H NOESY experiment showing reversible complex formation between **QQ‐Mes** (4.0 mm) and **3ai** (20 mm). Mixing time = 2.0 sec. (c) Fluorescence quenching of intermediate species (20 µm) by catalytic amount of **3a**. (d) *X*‐band EPR spectroscopy analysis using PBN as a radical scavenger. Frequency = 9.38 GHz. Simulation parameters: *g* = [2.0066 2.0055 2.0011], *A*(^14^N) = [11.07 11.44 90.14]. PBN = phenyl *N*‐*tert*‐butylnitrone.

Spectroscopic studies were conducted to shed light on the reaction mechanism (Figure 4). Stern–Volmer quenching experiments using a 20 µm solution of **QQ‐PyH** in DMF revealed linear plots for both **3a** and CCl_3_Br, with *K*_SV_ values of 200.8 and 266.6 m ^−1^, respectively (Figure 5a). In contrast, UV–vis spectra of dilute **QQ‐PyH** solutions showed negligible changes upon addition of either **3a** or CCl_3_Br, suggesting that the substrate remains in a dynamic equilibrium between bound and unbound states. To probe this interaction further, **3a** was added to a solution of **QQ‐Mes**, which is soluble in conventional NMR solvents without acid additive. Distinct downfield shifts of two *peri*‐proton signals (from δ7.18 and δ6.54 to δ7.17 and δ6.59, respectively) were observed (Figure 5b, left), indicating hydrogen‐bonding between the amide and the QQ framework. Additional evidence was obtained when 2‐methylbenzamide (**3ai**) was added to **QQ‐Mes**: a nuclear Overhauser effect (NOE) was detected between the substrate methyl protons and the *peri* proton of **QQ‐Mes** (Figure 5b, right). In contrast, no NOE was observed for the *para*‐methyl protons of the mesityl substituent, confirming that the observed NOE arises from reversible hydrogen‐bonding between **3ai** and the QQ scaffold. On the other hand, upon irradiation of a solution of **QQ‐PyH** and CCl_3_Br, an instantaneous change in both color and fluorescence was observed, suggesting the formation of a CCl_3_Br‐derived adduct. Fluorescence quenching studies revealed that the addition of only 0.1 equiv of **3a** to the irradiated solution effectively quenches its emission (Figure 5c). Further addition of **3a** did not alter the fluorescence significantly, indicating that the catalytic intermediate also binds reversibly to the amide substrate. To further probe radical formation, electron paramagnetic resonance (EPR) spectroscopy was performed using phenyl *N*‐*tert*‐ butylnitrone (PBN) as a spin trap (Figure 5d). A mixture of **QQ‐PyH** and **3a** generated a radical species upon 440 nm irradiation, displaying the characteristic signal of a PBN‐ trapped radical [88]. In contrast, no *N*‐oxide radical signal was observed for a **QQ‐PyH**/CCl_3_Br mixture, presumably due to rapid recombination of [**QQ‐PyH**]^•+^ and CCl_3_ radical.

### 2.5 Computational Analysis

**FIGURE 6:**
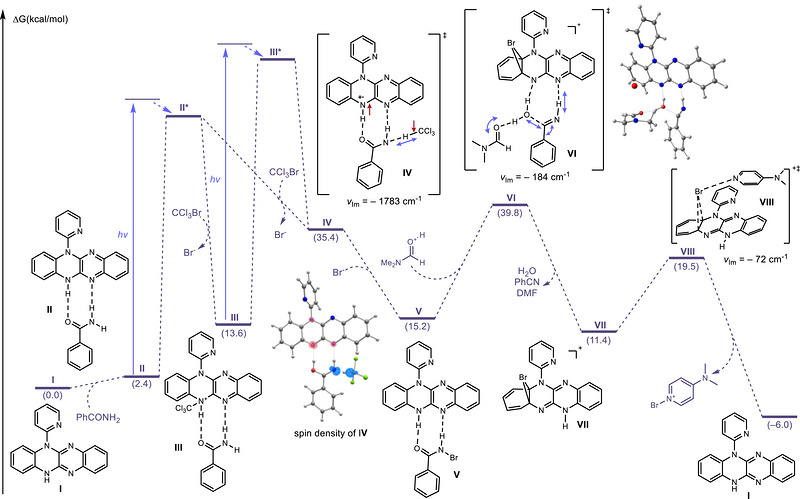
DFT computational analysis of the **QQ‐PyH** catalyzed direct nitrilization in SMD(DMF, *ε*_ps_ = 37.219)/uB3LYP‐D3/def2‐TZVP//uB3LYP‐D3/def2‐SVP level of theory. Gibbs free energies (kcal/mol) are given in parentheses.

Based on the mechanistic studies, we carried out DFT calculations to further substantiate the reaction pathway (Figure 6). The **QQ‐PyH** photocatalyst (**I**) is proposed to form adduct **II** through a reversible hydrogen‐bonding network with the amide substrate. Upon photoexcitation, **II** reduces CCl_3_Br to generate a CCl_3_ radical and bromide ion, the former recombining with the photosensitizer radical to give intermediate **III**. Subsequent excitation of **III** induces homolytic cleavage of the *N*─CCl_3_ bond (Figure S46 for the frontier orbital diagrams), releasing CCl_3_ radical, which abstracts the amide hydrogen via transition structure **IV** in the excited singlet state. Alternatively, the CCl_3_ radical generated directly from **II** can also abstract the amide hydrogen. Concomitant recombination with bromide affords the *N*‐ bromo intermediate **V**. Bromide rearrangement, followed by oxygen protonation, furnishes the nitrile product through the dehydration transition structure **IV** (Δ*G* ^‡^ = 25.6 kcal/mol). Catalyst turnover is achieved by DMAP‐mediated removal of the bromonium ion (Δ*G* ^‡^ = 8.1 kcal/mol). The bromonium ion is expected to be captured by residual water or solvent, subsequently reacting with another equivalent of CCl_3_Br under photolytic conditions to yield dibromine to close the catalytic cycle (Figure S49).

## 3 Conclusion

In conclusion, we report azapolycyclic QQ photosensitizers as effective catalysts for the light‐driven direct nitrilization of primary amides. This method accommodates aryl, heteroaryl, olefinic, and alkyl substrates, including bioactive derivatives, with broad functional group tolerance. Spectroscopic and computational analyses support a mechanism involving reversible substrate binding via hydrogen bonding and hydrogen‐atom abstraction. Collectively, these findings establish a practical platform for the synthesis of nitriles from amides under mild conditions.

## 4 Experimental Section

### 4.1 General Procedure to Synthesize QQ‐Ar

To a mixture of **1** (0.250 mmol) and solvent (toluene for **1a** and DMF for **1b**‐**f**, respectively, 5.0 mL) was added NaH (60% in mineral oil, 20 mg, 0.500 mmol) at 0°C and the mixture was stirred at 0°C for 30 min. To the mixture was added 2,3‐ dichloroquinoxaline (**2**, 50 mg, 0.250 mmol) and the mixture was stirred at 110°C for 16 h. The mixture was concentrated under reduced pressure and the residue was taken into a biphasic mixture of H_2_O (50 mL) and 1% formic acid in CH_2_Cl_2_ (50 mL). The aqueous layer was extracted with CH_2_Cl_2_ (with 1% formic acid, 50 mL x 2) and the combined organic layer was washed with H_2_O (20 mL x 2) and brine (10 mL) sequentially. The combined organic layer was dried over Na_2_SO_4_, concentrated, and triturated with EtOAc/Hx = 1:4 (10 mL). The precipitant was collected by filtration and washed with Hx (10 mL) to obtain the product. If necessary, the residue was further purified by sublimation at 220°C∼240°C under high vacuum.

### 4.2 General Procedure for Direct Nitrilization

To a 4 mL vial equipped with a stir bar were added amide **3** (1 equiv), DMAP (10 mol%), **QQ‐PyH** (10 mol.%), CCl3Br (2 equiv) and DMF (0.25 m) in glove box. The mixture was stirred for 4 h with irradiation of blue LED (440 nm, 30 W) by using Kessil lamp. The reaction mixture was diluted with EtOAc and brine, and the combined organic layer was dried over MgSO_4_ then concentrated under reduced pressure. The residue was purified by SiO_2_ column chromatography to obtain the product.

[CCDC 2492856 (**QQ‐Mes**) and 2492876 (**QQ‐PyH‐HOAc**) contain the supplementary crystallographic data for this paper. These data can be obtained free of charge from The Cambridge Crystallographic Data Centre via www.ccdc.cam.ac.uk/data_request/cif.]

## Funding

This research was supported by a National Research Foundation of Korea (NRF) grant funded by the Korea government (MSIT) (RS‐2025‐16069907) and by Global‐Learning & Academic research institution for Master's·PhD students, and Postdocs (G‐LAMP) Program of the National Research Foundation of Korea (NRF) grant funded by the Ministry of Education(RS‐2025‐25442707).

## Conflicts of Interest

The authors declare no conflicts of interest.

## Supporting information




**Supporting File 1**: advs75144‐sup‐0001‐SuppMat.pdf.

**Supporting File 2**: advs75144‐sup‐0002‐Data.zip.

## Data Availability

The data that supports the findings of this study are available in the supplementary material of this article.
