# Supplementary material for: Substrate‐Affinitive P‐Type Azapolycyclic Photosensitizer for Chemoselective Nitrilization Under Mild Conditions
Source: Adv Sci (Weinh). 2026 Apr 7;13(36):e75144. doi: 10.1002/advs.75144 (PMC13317559; doi:10.1002/advs.75144)

# *Supporting Information*

## Substrate-affinitive P-type azapolycyclic photosensitizer for chemoselective nitrilization under mild conditions

Seongwoo Bae,[1,2] Dongwook Kim[3] and Jinwoo Kim*[1,2]

[1]*Department of Chemistry, Chungnam National University, Daejeon 34134, South Korea*
[2]*Institute for Sciences of the Universe, Chungnam National University, Daejeon 34134, South Korea*
[3]*Center for Catalytic Hydrocarbon Functionalization, Institute of Basic Science, Daejeon 34134, South Korea*

## Table of contents

| | | |
|---|---|---|
| **I.** | **General methods** | **S2** |
| **II.** | **Synthesis of starting materials** | **S3** |
| **III.** | **Catalyst characterization** | **S8** |
| **IV.** | **Reaction parameter variation** | **S25** |
| **V.** | **QQ-Ar-catalyzed direct amide nitrilization** | **S27** |
| **VI.** | **Mechanistic studies** | **S35** |
| **VII.** | **Computational analysis for catalytic cycle** | **S40** |
| **VIII.** | **References** | **S46** |
| | ***Appendix I. Crystallographic data*** | **S47** |
| | ***Appendix II. Copies of $^{1}H$, $^{13}C$, and $^{19}F$ NMR spectra of obtained products*** | **S73** |

## I. General methods

Unless otherwise stated, all commercial reagents were used without additional purification. All the chemical reaction was performed under Ar or $N_2$ atmosphere, using glove box or Schlenk technique. Solvents were sparged with argon and dried over activated 3 Å molecular sieves before use. Analytical thin layer chromatography (TLC) was performed on Supelco silica gel $F_{254}$ plates. Visualization on TLC was achieved by using UV light (254 nm), treatment with acidic anisaldehyde, 5% phosphormolybdic acid in ethanol, iodine vapor, or aqueous potassium permanganate stain followed by heating. Reaction was monitored by LC-MS-MS using Thermo Fisher Scientific TSQ Quantis Plus (NFEC-2025-10-308785, supported by National Research Foundation of Korea grant funded by the Korea government, RS-2025-00521768) Column chromatography was performed on silica gel (SiliCycle® Silica Flash® P60, 230-400 mesh).

$^1$H NMR was recorded on Bruker Avance Neo 500 (BBFO, 500 MHz) in Institute of Basic Science, Daejeon, South Korea, Bruker Avance III 600 (BBFO, 600 MHz) and Avance Neo 400 (BBO, 400 MHz) in Chungnam National University, Daejeon, South Korea. Chemical shifts (Chemical shifts (δ) were quoted in parts per million (ppm) referenced to the residual solvent peak or 0.0 ppm for tetramethylsilane ($^1$H). The following abbreviations were used to describe peak splitting patterns when appropriate: s (singlet), br. s (broad singlet), d (doublet), t (triplet), q (quartet), p (pentet), hept (heptet), dd (doublet of doublet), and m (multiplet). Coupling constants, *J*, were reported in hertz (Hz). $^{13}$C{$^1$H} NMR was recorded on Buker Avance Neo 500 (BBFO, 126 MHz) or Bruker Avance III 600 (BBFO, 151 MHz) and was fully decoupled by broad band proton decoupling. Chemical shifts were reported in ppm referenced to the residual solvent peak. $^{19}$F{$^1$H} NMR were recorded on Buker Avance Neo 500 (BBFO, 470 MHz) referenced to $CCl_3F$. Infrared spectra (IR) were collected on Thermo Fischer Nicolet iS10 in Chungnam National University. Frequencies are given in wave numbers (cm$^{-1}$) and only selected peaks were reported. Cyclic voltammetry analysis was performed using PalmSens PS4 in Chungnam National University (20231437-02-015). UV-Vis absorption spectra were obtained using KLAB Optizen POP UV-Vis spectrometer. Fluorescence emission spectra were obtained using Scinco FS-2 fluorescence spectrometer. X-band CW electron paramagnetic resonance (EPR) spectroscopy was performed using Bruker EMXplus spectrometer equipped with standard resonator. The spectral simulation was performed using Easyspin 5.2.28 package run under MATLAB interface. High resolution mass spectra (HRMS) were obtained from Supercritical Fluid Chromatograph combined with Waters Xevo G2-XS QTOF Mass Spectrometer (NFEC-2022-12-283850) at the Chiral Material Core Facility Center of Sungkyunkwan University, Suwon, South Korea (ESI) and Jeol JMS 700 High resolution mass spectrometer (NFEC-2009-12-077562) at the Korea Basic Science Institute, Daegu, South Korea (EI). The data collection for single crystal structure analysis of **QQ-Mes** and **QQ-PyH-HOAc** was carried out on a Bruker D8 QUEST diffractometer equipped with Iμs 3.0 Mo x-ray tube and Photon II 14 detector under cryo-condition at 173 K by N2 (g) flow in the Center for Catalytic Hydrocarbon Functionalizations, Institute for Basic Science (IBS). The diffraction data were integrated, scaled, and reduced by using the Bruker APEX5 software. The crystal structures were solved by the SHELX structure solution program and refined by full-matrix least-squares calculations with the SHELXL on the ShelXle interface. All non-hydrogen atoms are refined anisotropically.

## II. Synthesis of starting materials

### II-1. Photocatalyst synthesis

*Step 1. Preparation of* $N^1$*-arylbenzene-1,2-diamines*

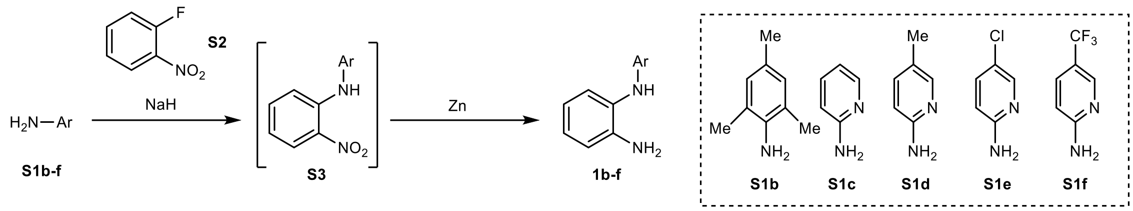

$N^1$-Phenylbenzene-1,2-diamine (**1a**) was purchased from TCI Chemicals and used without further purification. For **1b** to **1f**, to a solution of aniline **S1b-f** (3.00 mmol) in THF (5 mL) was added NaH (60 % in mineral oil, 120 mg, 3.00 mmol) at 0 °C and the mixture was stirred at 25 °C for 30 min then cooled to 0 °C. To the mixture was added 1-fluoro-2-nitrobenzene (**S2**, 423 mg, 3.00 mmol) and the mixture was further stirred at 70 °C for 12 h. The reaction mixture was concentrated under reduced pressure, dissolved in a minimal amount of $CH_2Cl_2$, filtered through a pad of celite, washed with $CH_2Cl_2$, and the filtrate was concentrated under reduced pressure to obtain crude **S3**. The residue was dissolved in MeOH (10 mL), cooled to 0 °C, and zinc powder (1.89 g, 30.0 mmol) was added. To the mixture was then added sat. aq. $NH_4Cl$ (10 mL) and stirred at 25 °C for 16 h. The biphasic mixture was filtered through a pad of celite, concentrated to ca. 50% of its original volume, diluted with $CH_2Cl_2$ (30 mL) and $H_2O$ (30 mL), and the aqueous layer was extracted with $CH_2Cl_2$ (30 mL x 2). The combined organic layer was dried over $Na_2SO_4$, concentrated, and the residue was purified by $SiO_2$ column chromatography to obtain the product.

$N^1$-Mesitylbenzene-1,2-diamine (**1b**)

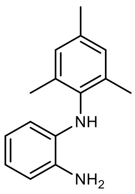

551 mg, 81%; Brown solid; $R_f$ = 0.50 ($CH_2Cl_2$/Hx = 1:1); m.p. 84-86 ºC; IR (diamond) 3425, 3343, 3024, 2947, 2915, 2854, 1623, 1588, 1500, 1482, 1277, 739 cm$^{-1}$; $^1$H NMR (500 MHz, $CDCl_3$) δ 6.94 (s, 2H), 6.80 (d, $J$ = 7.5 Hz, 1H), 6.77 – 6.71 (m, 1H), 6.69 – 6.63 (m, 1H), 6.25 (d, $J$ = 7.8 Hz, 1H), 4.78 (s, 1H), 3.57 (s, 2H), 2.31 (s, 3H), 2.14 (s, 6H); $^{13}$C NMR (126 MHz, $CDCl_3$) δ 137.0, 135.6, 134.9, 134.1, 133.5, 129.4, 120.4, 120.3, 116.4, 115.1, 21.0, 18.2; HRMS (ESI) m/z: [M+H]$^+$ Calcd for $C_{15}H_{19}N_2$: 227.1543, found: 227.1546.

$N^1$-(Pyridin-2-yl)benzene-1,2-diamine (**1c**)

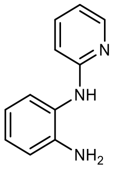

372 mg, 67%; Brown solid; $R_f$ = 0.15 (EtOAc/$CH_2Cl_2$ = 1:4); m.p. 122-124 ºC; IR (diamond) 3477, 3385, 3196, 3089, 3031, 3004, 2964, 2943, 1620, 1584, 1450, 1435, 1254, 750 cm$^{-1}$; $^1$H NMR (500 MHz, $CDCl_3$) δ 8.14 (dd, $J$ = 5.2, 1.2 Hz, 1H), 7.44 (ddd, $J$ = 8.8, 7.2, 1.9 Hz, 1H), 7.17 (dd, $J$ = 7.8, 1.4 Hz, 1H), 7.08 (td, $J$ = 7.9, 1.4 Hz, 1H), 6.82 (dd, $J$ = 8.0, 1.3 Hz, 1H), 6.77 (td, $J$ = 7.6, 1.4 Hz, 1H), 6.766 (br. s., 1H), 6.68 (ddd, $J$ = 7.0, 5.2, 0.8 Hz, 1H), 6.43 (d, $J$ = 8.5 Hz, 1H), 3.86 (s, 2H); $^{13}$C NMR (126 MHz, $CDCl_3$) δ 157.9,

148.1, 143.2, 138.3, 127.4, 127.2, 125.6, 119.0, 116.4, 114.5, 107.6; HRMS (ESI) m/z: $[M+H]^+$ Calcd for $C_{11}H_{12}N_3$: 186.1026, found: 186.1031.

*N*[1]-(5-Methylpyridin-2-yl)benzene-1,2-diamine (**1d**)

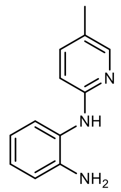

443 mg, 74%; Brown solid; $R_f$ = 0.15 (EtOAc/$CH_2Cl_2$ = 1:4); m.p. 82-84 ºC; IR (diamond) 3479, 3381, 3179, 3151, 3012, 2952, 2921, 1609, 1499, 1463, 1296, 744 $cm^{-1}$; $^1$H NMR (500 MHz, $CDCl_3$) δ 7.96 (s, 1H), 7.30 – 7.24 (m, 1H), 7.16 (dd, *J* = 7.7, 1.3 Hz, 1H), 7.09 – 7.04 (m, 1H), 6.81 (d, *J* = 7.9 Hz, 1H), 6.79 – 6.73 (m, 1H), 6.38 (br. s., 1H), 6.38 (d, *J* = 8.5 Hz, 1H), 3.80 (br. s, 2H), 2.20 (s, 3H); $^{13}$C NMR (151 MHz, $CDCl_3$) δ 155.7, 147.3, 143.0, 139.3, 127.1, 126.8, 126.3, 123.4, 119.1, 116.3, 107.4, 17.6; HRMS (ESI) m/z: $[M+H]^+$ Calcd for $C_{12}H_{14}N_3$: 200.1182, found: 200.1188.

*N*[1]-(5-Chloropyridin-2-yl)benzene-1,2-diamine (**1e**)

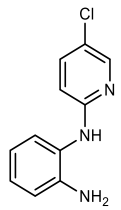

524 mg, 80%; Brown solid; $R_f$ = 0.25 (EtOAc/Hx = 1:4); m.p. 120-122 ºC; IR (diamond) 3423, 3339, 3281, 3052, 2929, 1600, 1586, 1500, 1475, 1468, 1434, 1132, 1107, 745 $cm^{-1}$; $^1$H NMR (500 MHz, $CDCl_3$) δ 8.08 (d, *J* = 2.4 Hz, 1H), 7.39 (dd, *J* = 8.9, 2.5 Hz, 1H), 7.15 (dd, *J* = 7.8, 1.5 Hz, 1H), 7.12 – 7.06 (m, 1H), 6.82 (dd, *J* = 7.9, 1.5 Hz, 1H), 6.78 (td, *J* = 7.6, 1.4 Hz, 1H), 6.58 (s, 1H), 6.38 (d, *J* = 8.9 Hz, 1H), 3.77 (s, 2H); $^{13}$C NMR (126 MHz, $CDCl_3$) δ 156.1, 146.2, 143.1, 138.1, 127.8, 127.2, 125.2, 121.3, 119.2, 116.5, 108.4; HRMS (ESI) m/z: $[M+H]^+$ Calcd for $C_{11}H_{11}ClN_3$: 220.0636, found: 220.0643.

*N*[1]-[5-(Trifluoromethyl)pyridin-2-yl]benzene-1,2-diamine (**1f**)

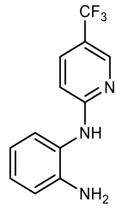

520 mg, 68%; Brown solid; $R_f$ = 0.25 (EtOAc/Hx = 1:4); m.p. 123-125 ºC; IR (diamond) 3432, 3350, 3216, 3149, 3080, 3059, 2991, 1618, 1608, 1509, 1498, 1319, 1072, 756 $cm^{-1}$; $^1$H NMR (500 MHz, $CDCl_3$) δ 8.38 (s, 1H), 7.61 (dd, *J* = 8.8, 2.1 Hz, 1H), 7.20 – 7.12 (m, 2H), 6.99 (s, 1H), 6.85 (dd, *J* = 8.0, 1.0 Hz, 1H), 6.82 – 6.77 (m, 1H), 6.45 (d, *J* = 8.8 Hz, 1H), 3.68 (s, 2H); $^{13}$C NMR (126 MHz, $CDCl_3$) δ 159.8, 145.8 (q, *J* = 4.6 Hz), 143.2, 135.4 (q, *J* = 3.3 Hz), 128.4, 127.6, 124.4 (d, *J* = 270.2 Hz), 124.1, 119.3, 117.2 (q, *J* = 33.1 Hz), 116.6, 106.7; $^{19}$F NMR (471 MHz, $CDCl_3$) δ -61.31; HRMS (ESI) m/z: $[M+H]^+$ Calcd for $C_{12}H_{11}F_3N_3$: 254.0900, found: 254.0903.

*Step 2. Preparation of QQ derivatives*

*i) 0.500 mmol scale*

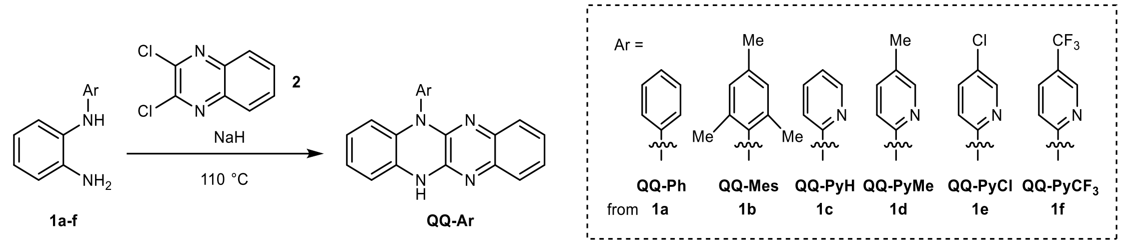


To a mixture of **1** (0.250 mmol) and solvent (toluene for **1a** and DMF for **1b**-**f**, respectively, 5.0 mL) was added NaH (60% in mineral oil, 20 mg, 0.500 mmol) at 0 °C and the mixture was stirred at 0 °C for 30 min. To the mixture was added 2,3-dichloroquinoxaline (**2**, 50 mg, 0.250 mmol) and the mixture was stirred at 110 °C for 16 h. The mixture was concentrated under reduced pressure and the residue was taken into a biphasic mixture of $H_2O$ (50 mL) and 1 % formic acid in $CH_2Cl_2$ (50 mL). The aqueous layer was extracted with $CH_2Cl_2$ (with 1% formic acid, 50 mL x 2) and the combined organic layer was washed with $H_2O$ (20 mL x 2) and brine (10 mL) sequentially. The combined organic layer was dried over $Na_2SO_4$, concentrated, and triturated with EtOAc/Hx = 1:4 (10 mL). The precipitant was collected by filtration and washed with Hx (10 mL) to obtain the product. If necessary, the residue was further purified by sublimation at 220~240 °C under high vacuum.

5-Phenyl-5,12-dihydroquinoxalino[2,3-b]quinoxaline (**QQ-Ph**)

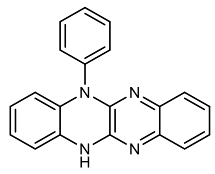


49.1 mg, 63%; yellow-green solid; $R_f$ = 0.46 (EtOAc/Hx = 1:4); m.p. 322-324 ºC; IR (diamond) 3187, 3060, 2960, 2923, 2853, 1716, 1682, 1577, 1523, 1458, 1394, 745 $cm^{-1}$; $^1H$ NMR (500 MHz, $CD_2Cl_2$/acetic acid-$d_4$=20:1) δ 7.63 (t, $J$ = 7.7 Hz, 2H), 7.53 (t, $J$ = 7.5 Hz, 1H), 7.38 – 7.31 (m, 2H), 7.08 (dd, $J$ = 3.6, 1.1 Hz, 2H), 7.02 (ddd, $J$ = 8.3, 5.1, 3.3 Hz, 1H), 6.98 (d, $J$ = 7.8 Hz, 1H), 6.74 (td, $J$ = 7.6, 1.3 Hz, 1H), 6.65 (dd, $J$ = 7.9, 1.5 Hz, 1H), 6.59 (td, $J$ = 7.8, 1.5 Hz, 1H), 5.95 (dd, $J$ = 8.2, 1.3 Hz, 1H); $^{13}C$ NMR (126 MHz, $CDCl_3$/acetic acid-$d_4$=20:1) δ 145.0, 144.5, 138.3, 137.1, 135.7, 132.8, 130.6, 129.8, 129.0, 128.8, 126.3, 126.3, 125.6, 123.4, 123.2, 121.2, 115.4, 115.0; HRMS (EI) m/z: $[M]^+$ Calcd for $C_{20}H_{14}N_4$: 310.1218, found: 310.1215.

5-Mesityl-5,12-dihydroquinoxalino[2,3-b]quinoxaline (**QQ-Mes**)

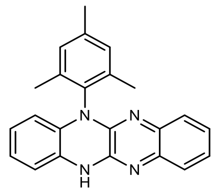


75.5 mg, 86%; yellow solid; $R_f$ = 0.57 (EtOAc/Hx = 1:4); m.p. 148-150 ºC; IR (diamond) 3191, 2973, 2947, 2916, 2853, 1582, 1524, 1457, 1396, 1301, 740 $cm^{-1}$; $^1H$ NMR (500 MHz, $CD_2Cl_2$) δ 7.21 (dd, $J$ = 7.6, 1.3 Hz, 1H), 7.12 – 7.03 (m, 5H), 6.68 (td, $J$ = 7.6, 1.3 Hz, 1H), 6.55 (t, $J$ = 7.6 Hz, 2H), 5.82 (dd, $J$ = 8.3, 1.1 Hz, 1H), 2.40 (s, 3H), 2.19 (s, 6H); $^{13}C$ NMR (126 MHz, $CDCl_3$) δ 145.0, 144.5, 138.3, 137.1, 135.7, 132.8, 130.6, 129.8, 129.0, 128.8, 126.3, 126.3, 125.6, 123.4, 123.2, 121.2,

115.4, 115.0; HRMS (EI) m/z: $[M]^+$ Calcd for $C_{23}H_{20}N_4$: 352.1688, found: 352.1687. A crystal of **QQ-Mes** is obtained by vapor diffusion of *n*-hexane into the $CHCl_3$ solution.

5-(Pyridin-2-yl)-5,12-dihydroquinoxalino[2,3-b]quinoxaline (**QQ-PyH**)

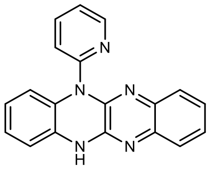

55.6 mg, 71%; yellow solid; $R_f$ = 0.22 (EtOAc/Hx = 1:4); m.p. 327-329 ºC; IR (diamond) 3207, 3058, 1588, 1579, 1522, 1460, 1422, 1395, 739 $cm^{-1}$; $^1H$ NMR (500 MHz, $CD_2Cl_2$/acetic acid-$d_4$=20:1) δ 8.79 (ddd, *J* = 4.9, 2.0, 0.9 Hz, 1H), 8.04 (td, *J* = 7.7, 2.0 Hz, 1H), 7.51 (ddd, *J* = 7.6, 4.9, 1.1 Hz, 1H), 7.45 (d, *J* = 7.8 Hz, 1H), 7.10 (d, *J* = 3.6 Hz, 2H), 7.06 – 7.01 (m, 1H), 6.98 (dt, *J* = 8.1, 1.1 Hz, 1H), 6.77 (td, *J* = 7.6, 1.2 Hz, 1H), 6.66 (dd, *J* = 7.9, 1.4 Hz, 1H), 6.60 (ddd, *J* = 8.8, 7.5, 1.4 Hz, 1H), 5.88 (dd, *J* = 8.1, 1.2 Hz, 1H); $^{13}C$ NMR (126 MHz, $CDCl_3$/acetic acid-$d_4$=20:1) δ 150.9, 150.4, 144.5, 144.2, 140.1, 137.9, 135.8, 131.4, 128.7, 126.6, 126.3, 125.6, 124.3, 124.3, 123.7, 123.3, 121.4, 115.6, 114.5; HRMS (EI) m/z: $[M]^+$ Calcd for $C_{19}H_{13}N_5$: 311.1171, found: 311.1174. A crystal of **QQ-PyH-HOAc** is obtained by slow evaporation of the $CHCl_3$/HOAc =20:1 solution.

5-(5-Methylpyridin-2-yl)-5,12-dihydroquinoxalino[2,3-b]quinoxaline (**QQ-PyMe**)

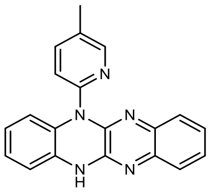

52.4 mg, 64%; yellow solid; $R_f$ = 0.23 (EtOAc/Hx = 1:4); m.p. 316-318 ºC; IR (diamond) 3208, 3062, 1578, 1522, 1463, 1422, 1397, 1307, 748, 741 $cm^{-1}$; $^1H$ NMR (500 MHz, $CDCl_3$/acetic acid-$d_4$=20:1) δ 8.61 (s, 1H), 7.80 (dd, *J* = 8.1, 2.4 Hz, 1H), 7.32 (dd, *J* = 8.0, 2.4 Hz, 1H), 7.12 (d, *J* = 7.2 Hz, 1H), 7.08 (ddt, *J* = 8.1, 5.9, 2.6 Hz, 1H), 7.01 (dt, *J* = 6.5, 1.8 Hz, 2H), 6.78 – 6.72 (m, 1H), 6.70 (d, *J* = 7.9 Hz, 1H), 6.60 (tt, *J* = 7.9, 1.5 Hz, 1H), 5.89 (dd, *J* = 8.4, 1.7 Hz, 1H), 2.47 (s, 3H); $^{13}C$ NMR (126 MHz, $CDCl_3$) δ 151.1, 147.8, 144.6, 144.2, 140.7, 138.0, 135.7, 134.4, 131.6, 128.6, 126.5, 126.3, 125.6, 124.8, 123.6, 123.3, 121.3, 115.6, 114.5, 18.4; HRMS (EI) m/z: $[M]^+$ Calcd for $C_{20}H_{15}N_5$: 325.1327, found: 325.1325.

5-(5-Chloropyridin-2-yl)-5,12-dihydroquinoxalino[2,3-b]quinoxaline (**QQ-PyCl**)

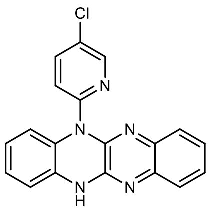

49.9 mg, 58%; yellow solid; $R_f$ = 0.38 (EtOAc/Hx = 1:4); m.p. 331-333 ºC; IR (diamond) 3185, 3059, 3001, 1575, 1526, 1457, 1422, 1395, 1367, 1305, 750, 740 $cm^{-1}$; $^1H$ NMR (500 MHz, $CDCl_3$/acetic acid-$d_4$=20:1) δ 8.73 (d, *J* = 2.6 Hz, 1H), 7.95 (dd, *J* = 8.4, 2.6 Hz, 1H), 7.41 (d, *J* = 8.3 Hz, 1H), 7.14 – 7.07 (m, 2H), 7.07 – 7.01 (m, 2H), 6.76 (t, *J* = 7.5 Hz, 1H), 6.68 (d, *J* = 7.7 Hz, 1H), 6.61 (t, *J* = 7.7 Hz, 1H), 5.93 (d, *J* = 8.1 Hz, 1H); $^{13}C$ NMR (126 MHz, $CDCl_3$/acetic acid-$d_4$=20:1) δ 149.9, 148.7, 144.3, 144.1, 139.6, 137.8, 136.1, 132.4, 131.1, 128.8, 126.8, 126.5, 126.4, 125.7, 123.9, 123.2, 121.6, 115.6, 114.4; HRMS (EI) m/z: $[M]^+$ Calcd for $C_{19}H_{12}ClN_5$: 345.0781, found: 345.0779.

5-[5-(trifluoromethyl)pyridin-2-yl]-5,12-dihydroquinoxalino[2,3-b]quinoxaline (**QQ-PyCF$_3$**)

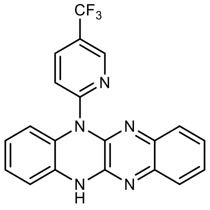


66.3 mg, 70%; yellow solid; $R_f$ = 0.38 (EtOAc/Hx = 1:4); m.p. 334-336 ºC; IR (diamond) 3211, 3101, 3059, 1598, 1578, 1526, 1465, 1425, 1397, 1327, 1126, 1082, 748, 742 cm$^{-1}$; $^1$H NMR (500 MHz, $CD_2Cl_2$/acetic acid-$d_4$=20:1) δ 9.05 (d, $J$ = 2.5 Hz, 1H), 8.25 (dd, $J$ = 8.3, 2.5 Hz, 1H), 7.65 (d, $J$ = 8.2 Hz, 1H), 7.16 – 7.11 (m, 2H), 7.06 (dt, $J$ = 8.3, 3.9 Hz, 1H), 7.02 (d, $J$ = 8.0 Hz, 1H), 6.81 (td, $J$ = 7.6, 1.2 Hz, 1H), 6.68 (dd, $J$ = 7.9, 1.4 Hz, 1H), 6.63 (t, $J$ = 7.7 Hz, 1H), 5.96 (d, $J$ = 9.3 Hz, 1H); $^{13}$C NMR (151 MHz, $CD_2Cl_2$/acetic acid-$d_4$=20:1) δ 154.2, 148.3 (t, $J$ = 4.1 Hz), 144.6, 144.5, 138.2, 137.3 (d, $J$ = 3.6 Hz), 137.1, 131.2, 129.4, 127.1, 126.9, 126.4, 126.2, 126.0, 124.3, 123.8 (q, $J$ = 272.5 Hz), 123.2, 122.3, 115.7, 114.8; $^{19}$F NMR (471 MHz, $CD_2Cl_2$/acetic acid-$d_4$=20:1) δ -62.60; HRMS (EI) m/z: [M]$^+$ Calcd for $C_{20}H_{12}F_3N_5$: 379.1045, found: 379.1044.

*ii) Gram-scale synthesis of* ***QQ-PyH***

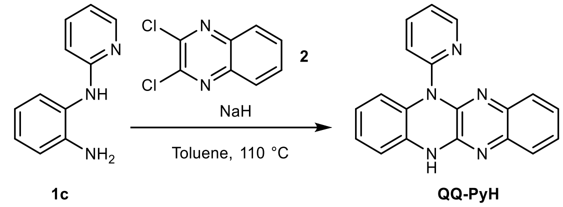


To compound **1c** (926 mg, 5.00 mmol) in toluene (50 mL) was added NaH (60% in mineral oil, 400 mg, 10.0 mmol) potionwise at 0 °C and the mixture was stirred at 0 °C for 30 min. To the mixture was added 2,3-dichloroquinoxaline (**2**, 1.00 g, 5.00 mmol) and the mixture was stirred at 110 °C for 16 h. The mixture was concentrated under reduced pressure and the residue was triturated in a biphasic mixture of $H_2O$ (100 mL) and $CH_2Cl_2$ (100 mL) for 12 h. The yellow solid was sequentially washed with $H_2O$ (50 mL), EtOH (30 mL) and $CH_2Cl_2$ (50 mL), triturated again in EtOAc/Hx = 1:1 (100 mL) for 4 h, and washed with Hx (50 mL) to obtain the product (1.18 g, 76%).

The following photocatalysts could not be synthesized, presumably due to the steric congestion.

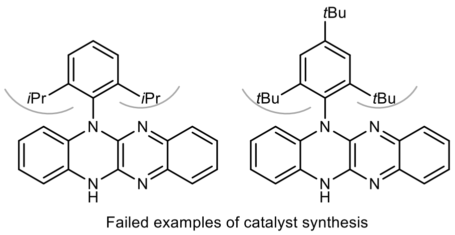


Failed examples of catalyst synthesis

## III. Catalyst characterization

### III-1. Frontier orbital analysis of QQ-Ar derivatives

#### III-1-1) General computational method

For frontier orbital analysis, DFT calculations were carried out with Gaussian 16 quantum chemical package.[1] Geometry optimizations were performed with uB3LYP[2-6] level of theory with Grimme's D3 correction[7] and def2-TZVP basis set. Vibrational frequency calculations were carried out at the same calculation theory level as the geometry optimization calculations, wherein thermochemistry correction energy ($G–E$) was acquired. No imaginary frequencies were found for all optimized intermediates. The single-point energy calculations of the optimized geometries were performed with same calculation level of theory. TD-DFT computation was performed at the same calculation theory level (uB3LYP-D3/def2TZVP) using nstates=10. Natural transition orbital (NTO) analysis for each transition mode was performed using Gaussian 16 quantum chemical package. Graphical structures are visualized with ChemCraft.

### III-1-2) Potential energy data of optimized photocatalyst structures

| Species | QQ-Ph | QQ-Mes | QQ-PyH | QQ-PyMe | QQ-PyCl | QQ-PyCF3 |
|---|---|---|---|---|---|---|
| Charge | 0 | 0 | 0 | 0 | 0 | 0 |
| Multiplicity | 1 | 1 | 1 | 1 | 1 | 1 |
| *E* (Hartree) | -989.989583 | -1107.998911 | -1006.033223 | -1045.368408 | -1465.659067 | -1343.229200 |
| *G-E* (Hartree) | 0.249766 | 0.326102 | 0.238130 | 0.262660 | 0.226280 | 0.235834 |
| *G* (Hartree) | -989.739818 | -1107.672809 | -1005.795093 | -1045.105748 | -1465.432787 | -1342.993366 |

**Table S1.** Energy data of optimized photocatalyst structures obtained in this study.

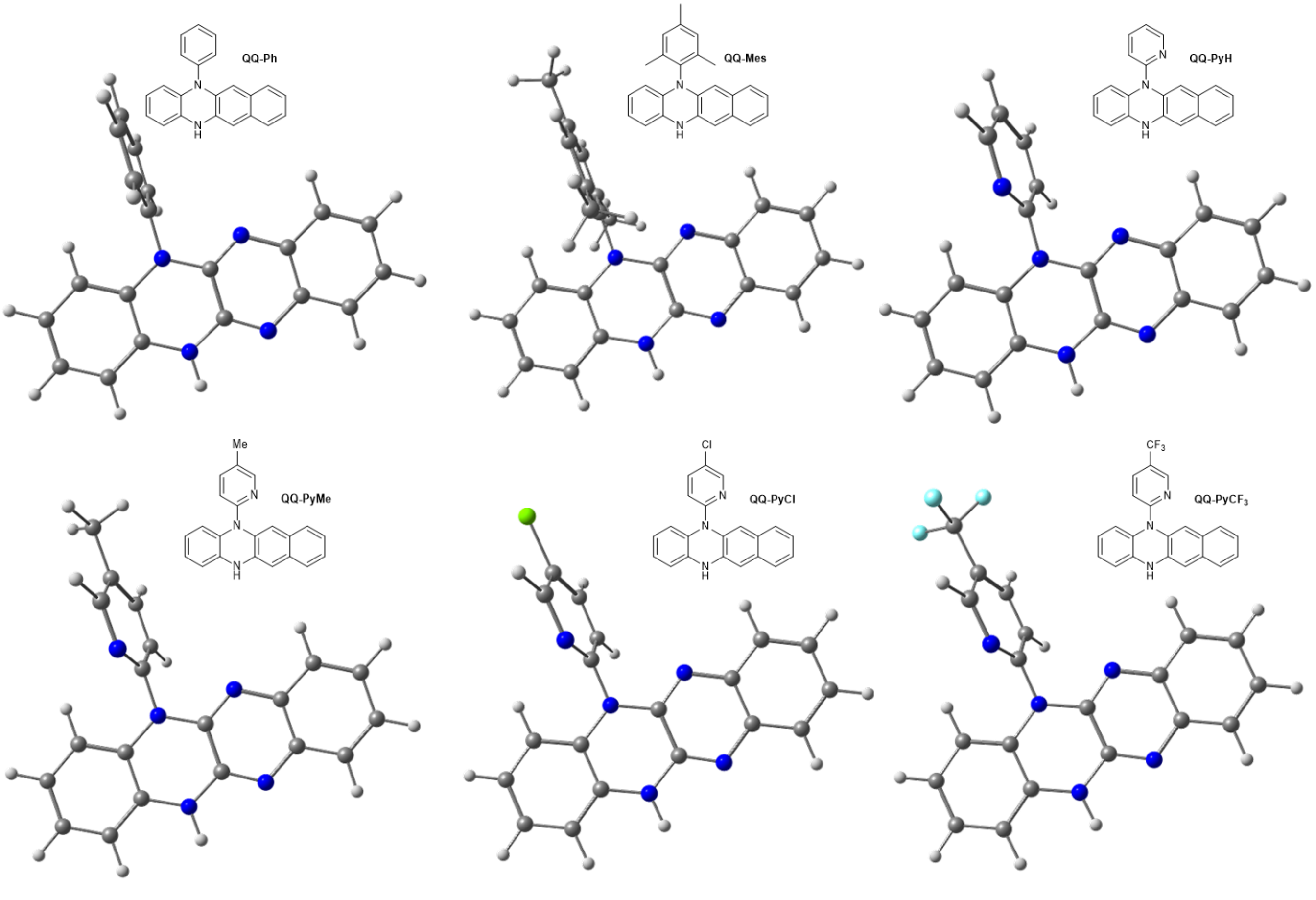


**Figure S1.** Structure of the optimized photocatalyst species

### III-1-3) Kohn-Sham orbital diagram of photocatalyst frontier orbitals

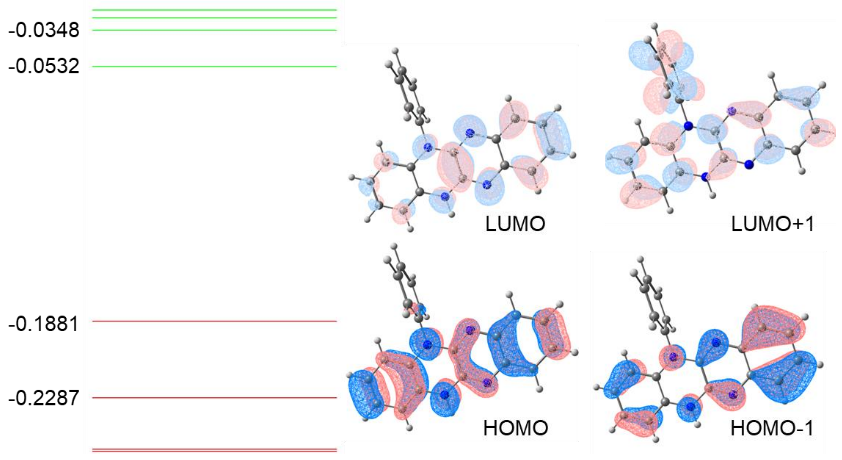


**Figure S2.** Frontier orbitals of **QQ-Ph**. Isovalue = 0.03.

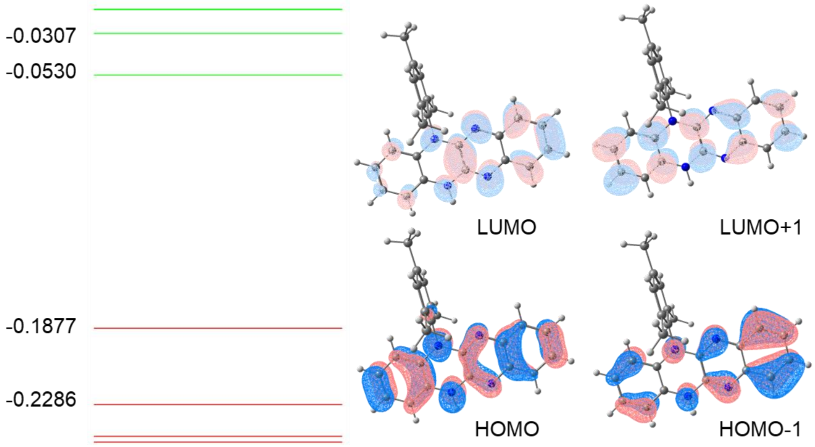


**Figure S3.** Frontier orbitals of **QQ-Mes**. Isovalue = 0.03.

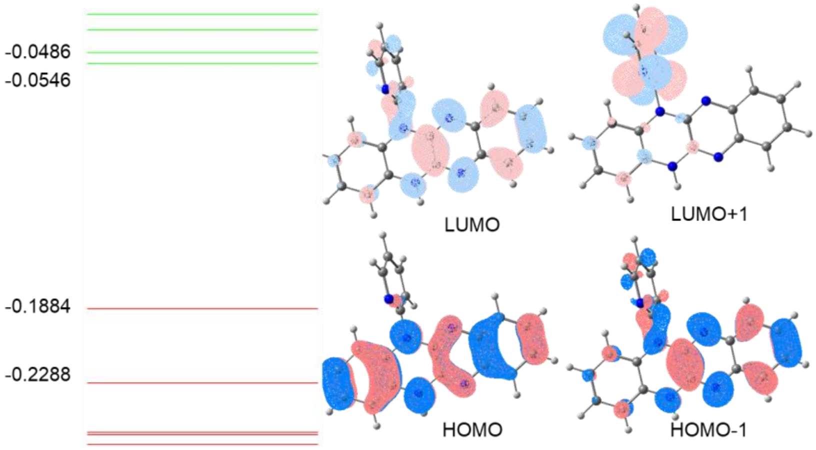


**Figure S4.** Frontier orbitals of **QQ-PyH**. Isovalue = 0.03.

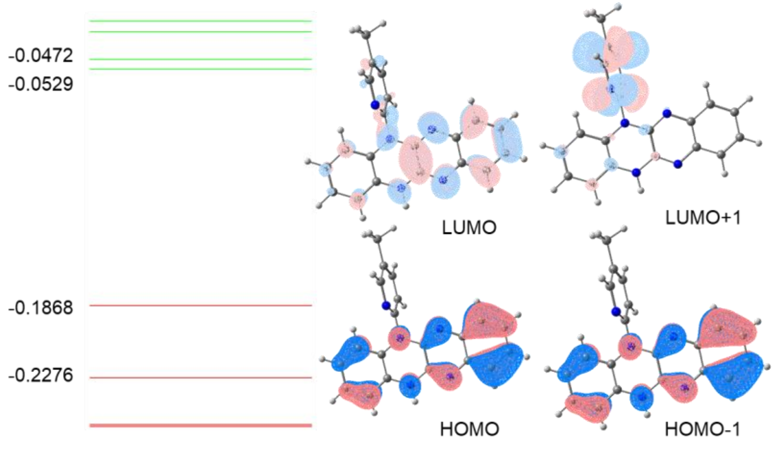


**Figure S5.** Frontier orbitals of **QQ-PyMe**. Isovalue = 0.03.

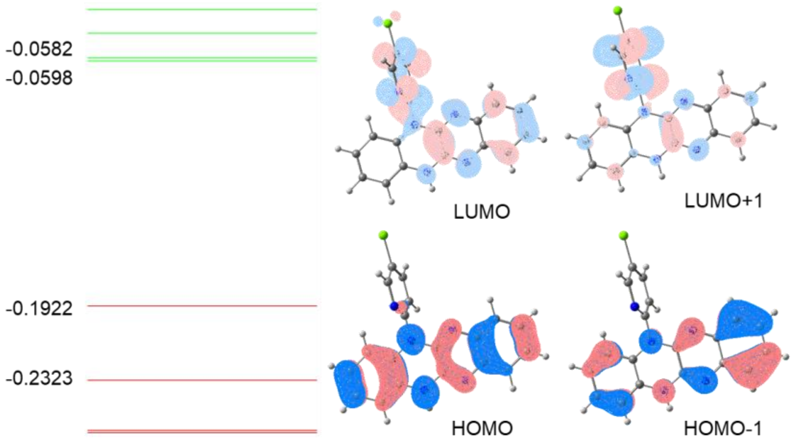


**Figure S6.** Frontier orbitals of **QQ-PyCl**. Isovalue = 0.03.

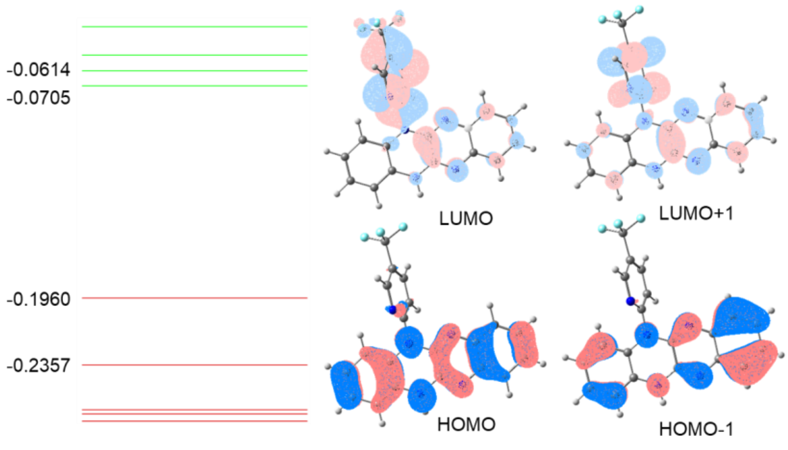


**Figure S7.** Frontier orbitals of **QQ-PyCF$_3$**. Isovalue = 0.03.

### III-2. Absorption and emission spectra of QQ-Ar derivatives

### III-2-1) UV-Vis absorption spectroscopy

**QQ-Ar** photocatalyst (1.2 mg, 0.004 mmol) was dissolved in 20 mL of DMF, followed by 10-fold dilution to prepare 20 μM solution. The solution was mixed thoroughly to ensure homogeneity. A 3 mL aliquot of the sample was placed in a cuvette and analyzed using UV-Vis spectroscopy over the wavelength range of 300 nm to 700 nm. Natural transition orbital (NTO) analysis was performed for selected simulated transitions.

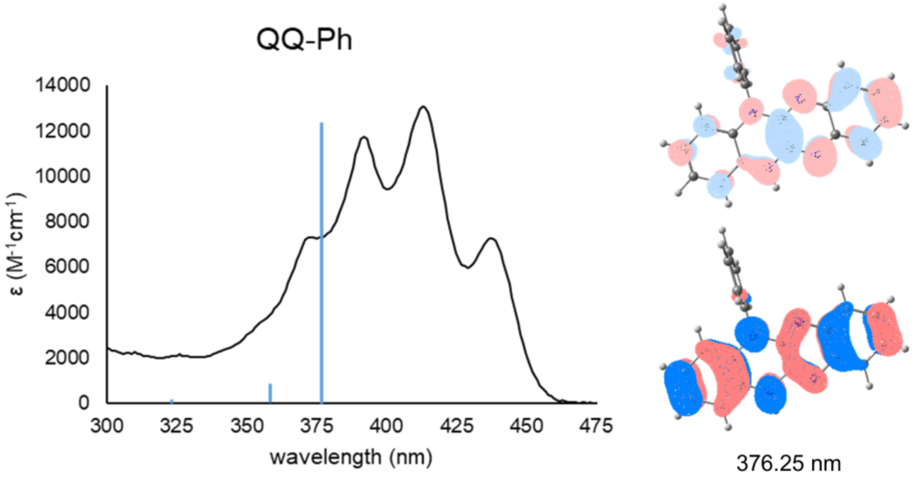


**Figure S8.** UV-Vis spectrum of **QQ-Ph** with TD-DFT simulation and corresponding **NTO** diagram (Isovalue = 0.03).

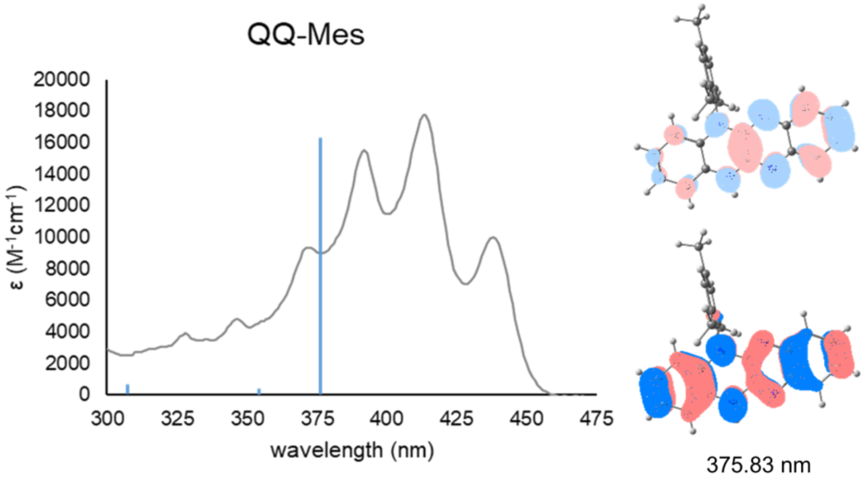


**Figure S9.** UV-Vis spectrum of **QQ-Mes** with TD-DFT simulation and corresponding **NTO** diagram (Isovalue = 0.03).

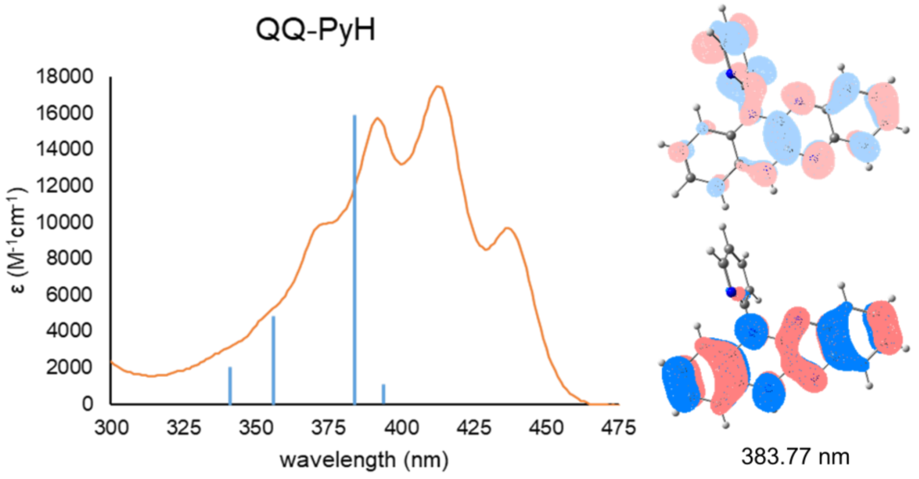


**Figure S10.** UV-Vis spectrum of **QQ-PyH** with TD-DFT simulation and corresponding **NTO** diagram (Isovalue = 0.03).

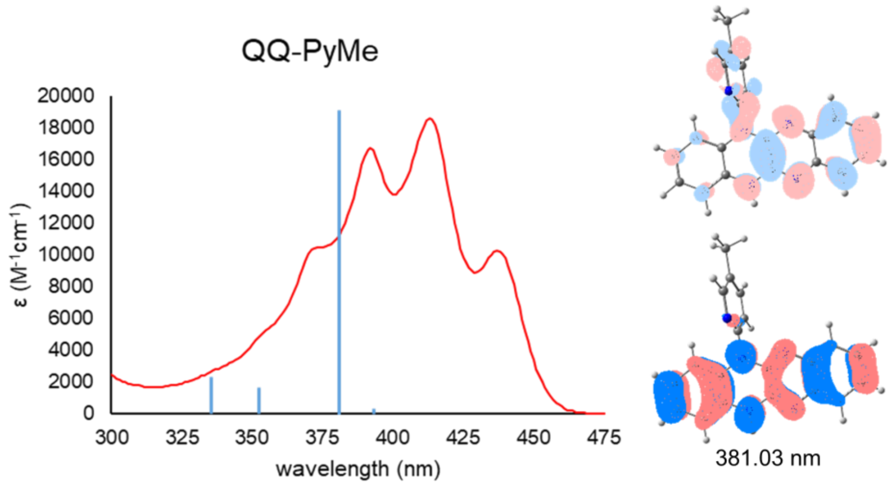


**Figure S11.** UV-Vis spectrum of **QQ-PyMe** with TD-DFT simulation and corresponding **NTO** diagram (Isovalue = 0.03).

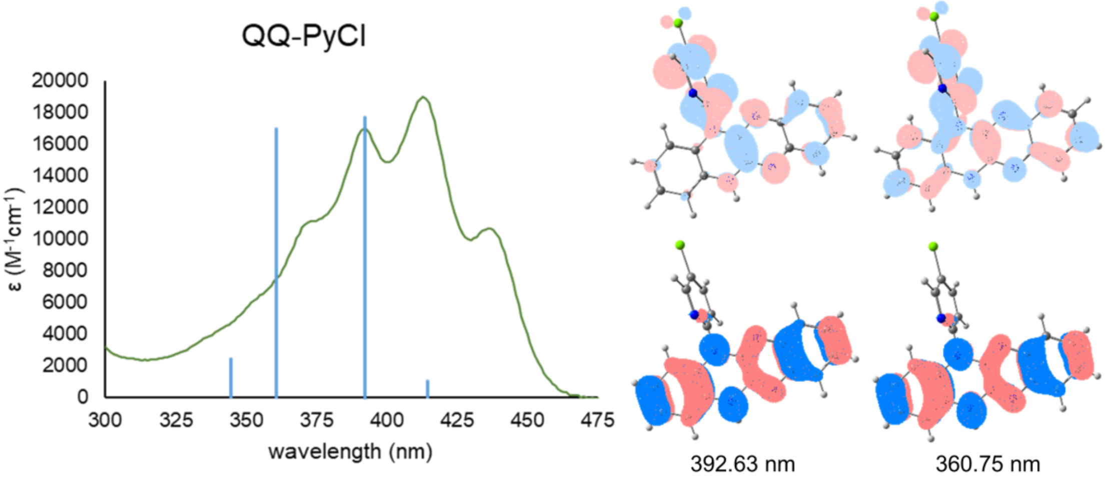


**Figure S12.** UV-Vis spectrum of **QQ-PyCl** with TD-DFT simulation and corresponding **NTO** diagram (Isovalue = 0.03).

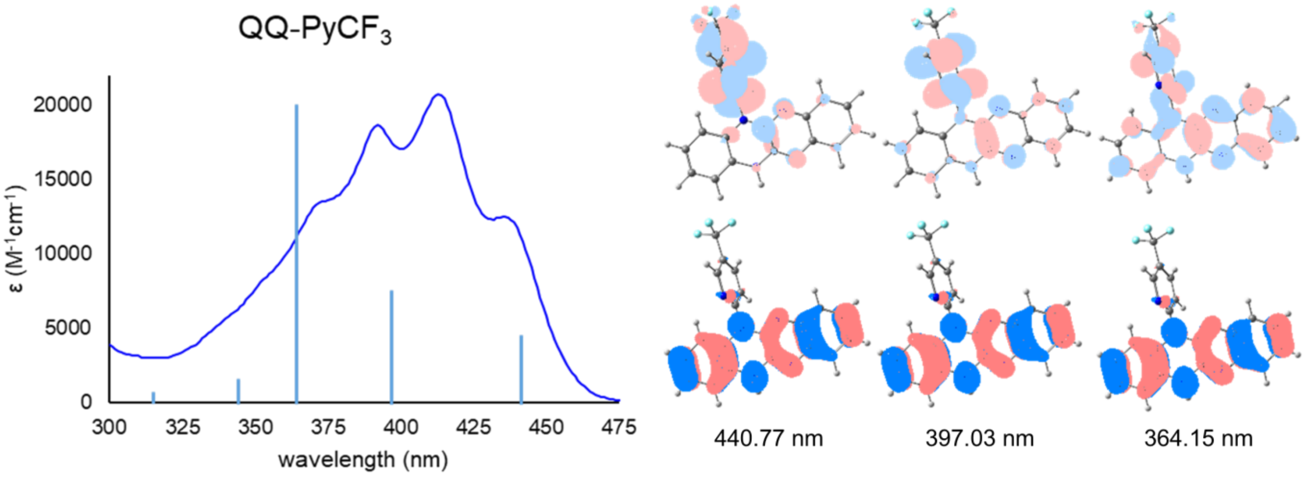


**Figure S13.** UV-Vis spectrum of **QQ-PyCF$_3$** with TD-DFT simulation and corresponding **NTO** diagram (Isovalue = 0.03).

### III-2-2) Fluorescence emission spectroscopy

**QQ-Ar** photocatalyst (1.2 mg, 0.004 mmol) was dissolved in 20 mL of DMF, followed by 10-fold dilution to prepare 20 μM solution. The solution was mixed thoroughly to ensure homogeneity. A 3 mL aliquot of the sample was placed in a cuvette and the fluorescence was observed using 350 nm excitation light over the wavelength range of 400 nm to 650 nm.

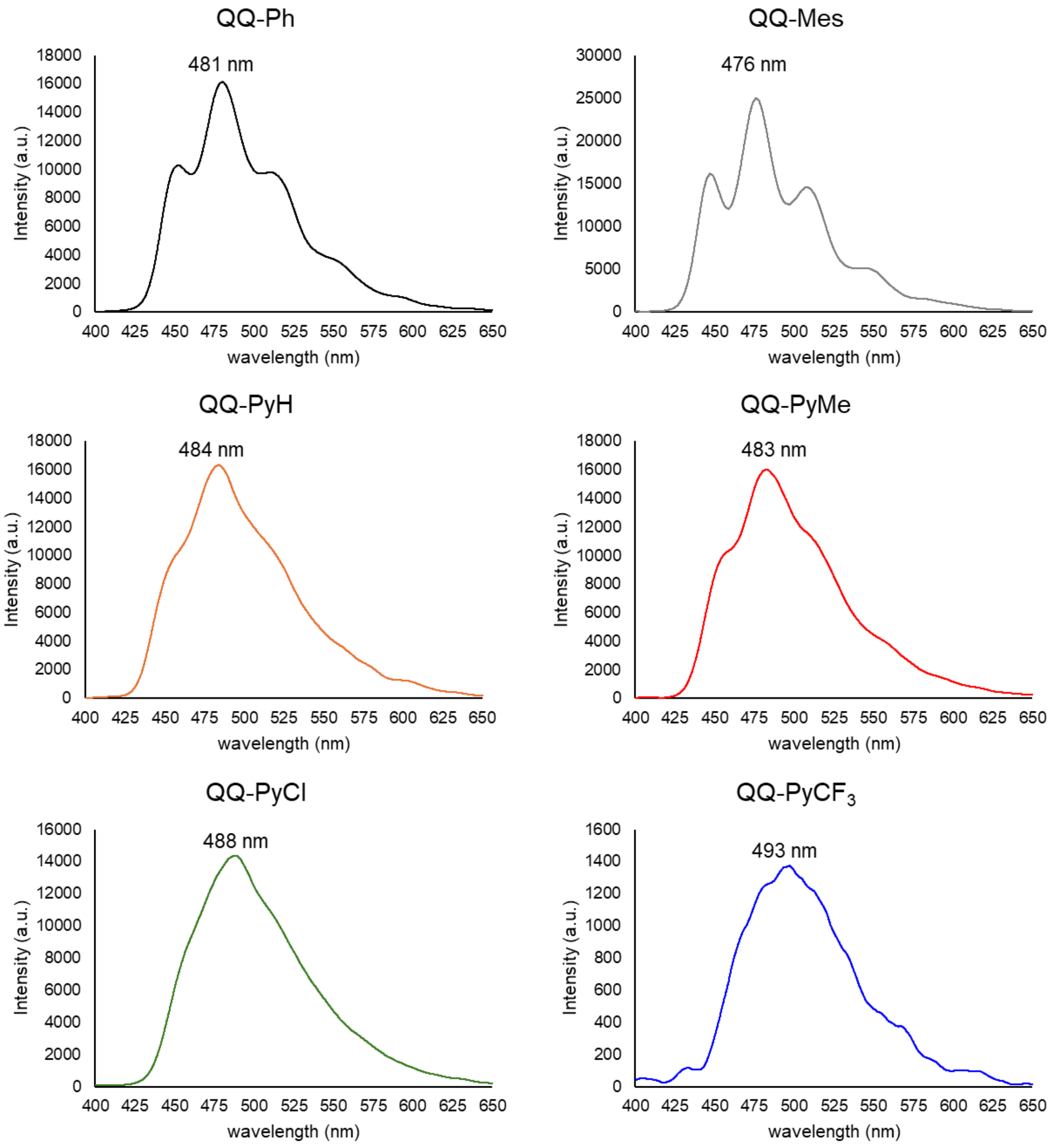


**Figure S14.** Fluorescence emission spectra of QQ-**Ar** species.

### III-3. Cyclic voltammetry analysis of QQ-Ar derivatives

Cyclic voltammetry analysis on **QQ-Ar** derivatives was performed in a 3-electrode cell consisting of Ag wire reference and counter electrode and 3 mm glassy carbon disc for working electrode. A solution of each compound (0.003 M) and $n$BuNPF$_6$ (0.1 M) in DMF (10 mL) was added to the electrochemical cell. Cyclic voltammetry scans were taken at the selected scan rates and in the selected potential window. The cyclic voltammogram of each complex was referenced to Fc/Fc$^+$ redox couple as an external standard.

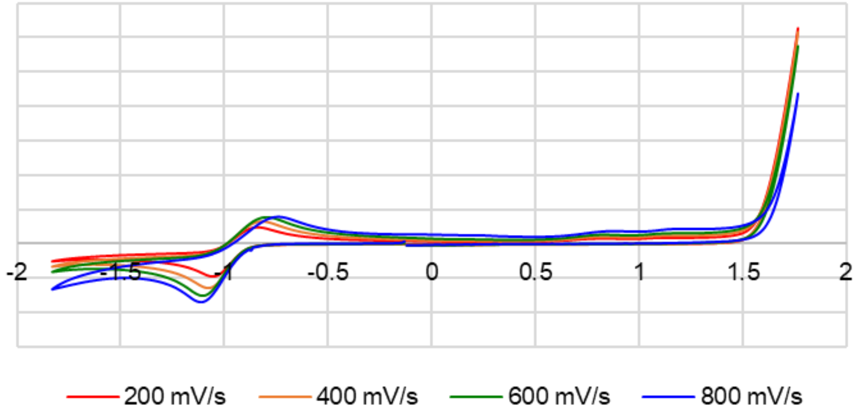


**Figure S15.** Full CV spectrum of **3a**.

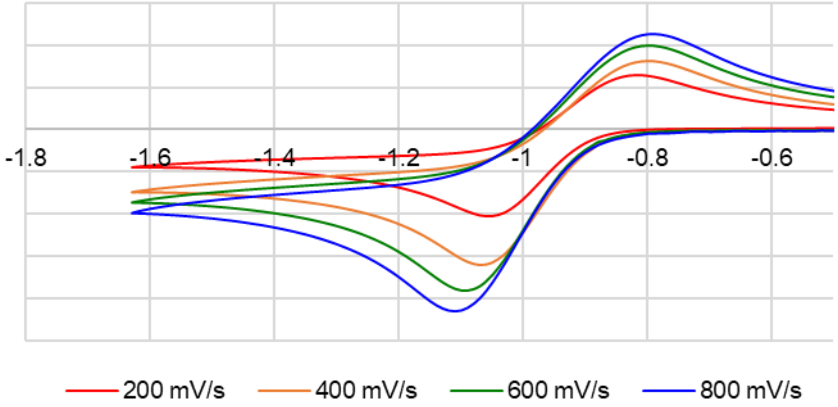


| Scan rate | $E_{1/2}$ |
|---|---|
| 200 mV/s | –0.940 |
| 400 mV/s | –0.935 |
| 600 mV/s | –0.945 |
| 800 mV/s | –0.955 |

**Figure S16.** Narrow CV spectrum of **3a** at the reductive region.

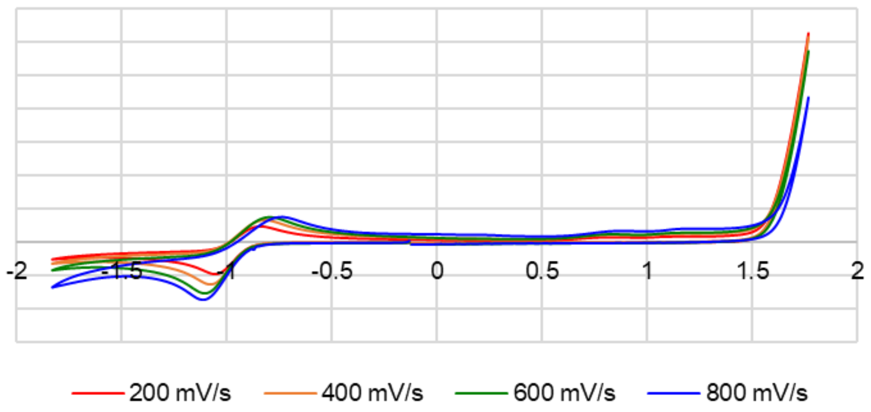


**Figure S17.** Full CV spectrum of $CCl_3Br$.

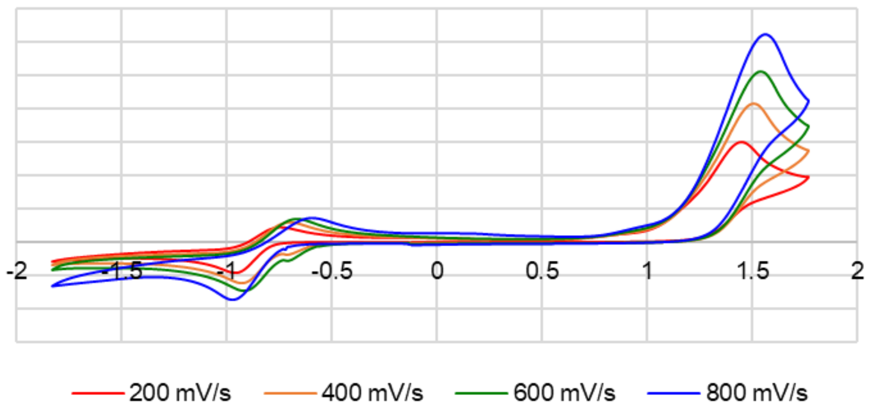


**Figure S18.** Full CV spectrum of DMAP.

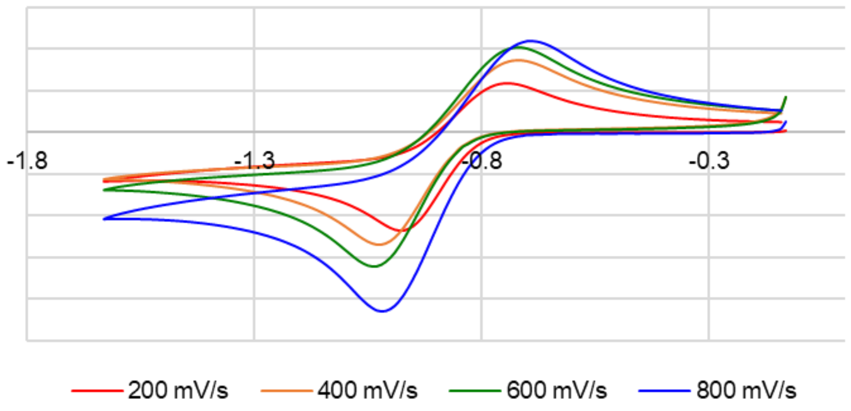


| Scan rate | $E_{1/2}$ |
|---|---|
| 200 mV/s | –0.865 |
| 400 mV/s | –0.870 |
| 600 mV/s | –0.875 |
| 800 mV/s | –0.860 |

**Figure S19.** Narrow CV spectrum of DMAP at the reductive region.

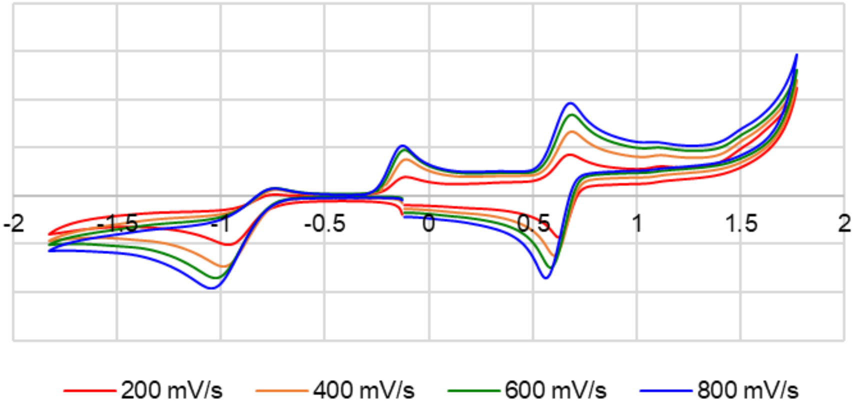


**Figure S20.** Full CV spectrum of **QQ-Ph**.

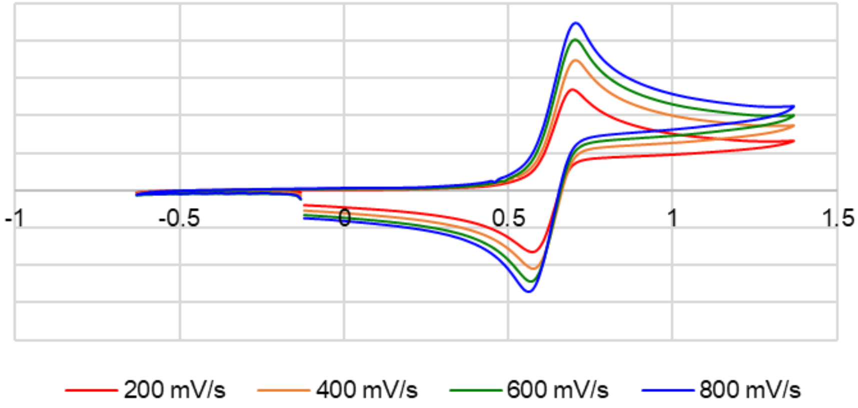


| Scan rate | $E_{1/2}$ |
|---|---|
| 200 mV/s | 0.635 |
| 400 mV/s | 0.640 |
| 600 mV/s | 0.635 |
| 800 mV/s | 0.635 |

**Figure S21.** Narrow CV spectrum of **QQ-Ph** at the oxidative region.

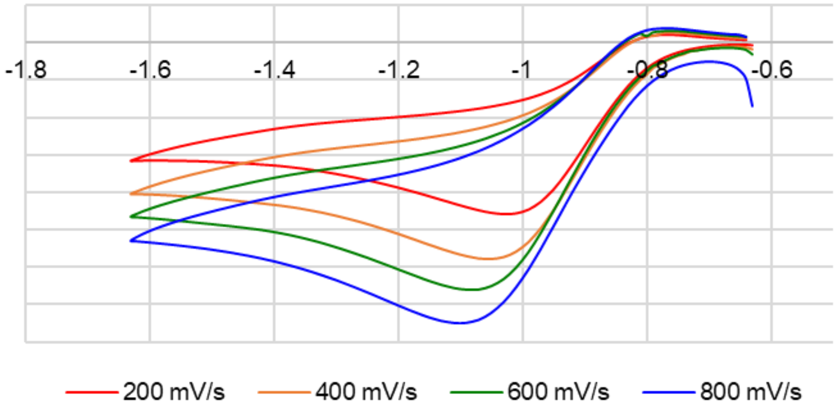


| Scan rate | $E_{pc}$ |
|---|---|
| 200 mV/s | –1.030 |
| 400 mV/s | –1.050 |
| 600 mV/s | –1.080 |
| 800 mV/s | –1.100 |

**Figure S22.** Narrow CV spectrum of **QQ-Ph** at the reductive region.

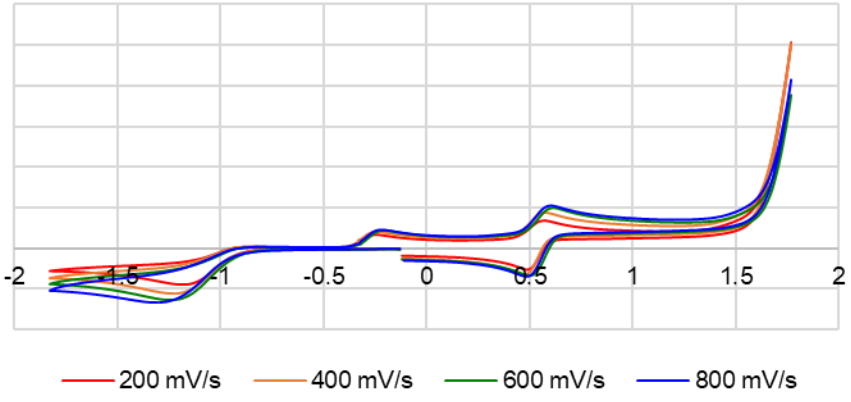


**Figure S23.** Full CV spectrum of **QQ-Mes**.

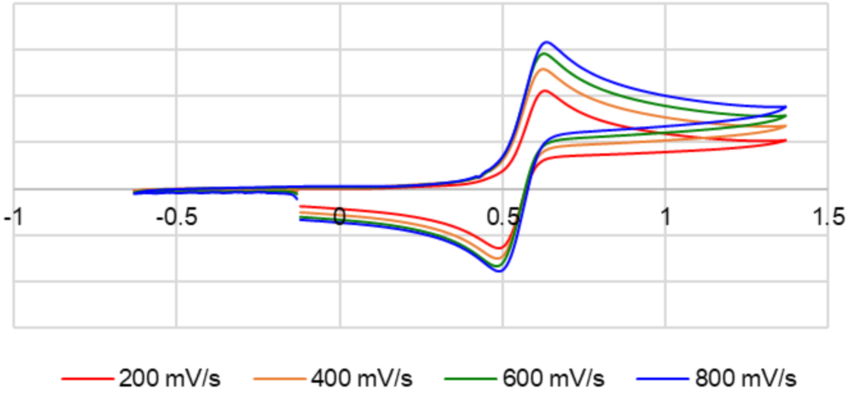


| Scan rate | $E_{1/2}$ |
|---|---|
| 200 mV/s | 0.555 |
| 400 mV/s | 0.550 |
| 600 mV/s | 0.550 |
| 800 mV/s | 0.555 |

**Figure S24.** Narrow CV spectrum of **QQ-Mes** at the oxidative region.

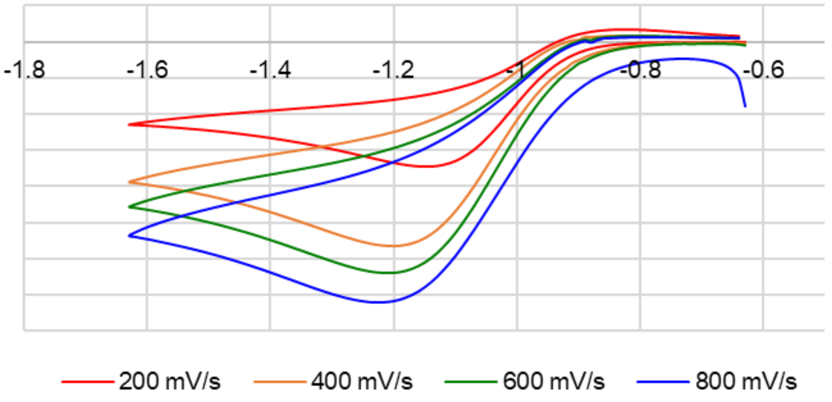


| Scan rate | $E_{pc}$ |
|---|---|
| 200 mV/s | –1.155 |
| 400 mV/s | –1.205 |
| 600 mV/s | –1.215 |
| 800 mV/s | –1.235 |

**Figure S25.** Narrow CV spectrum of **QQ-Mes** at the reductive region.

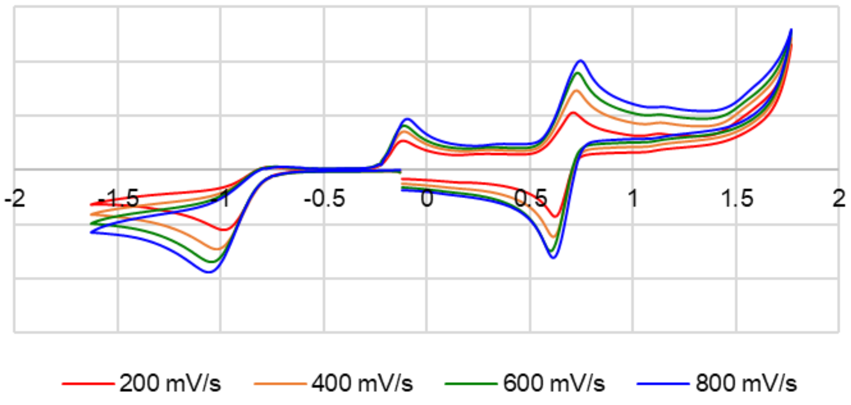


**Figure S26.** Full CV spectrum of **QQ-PyH**.

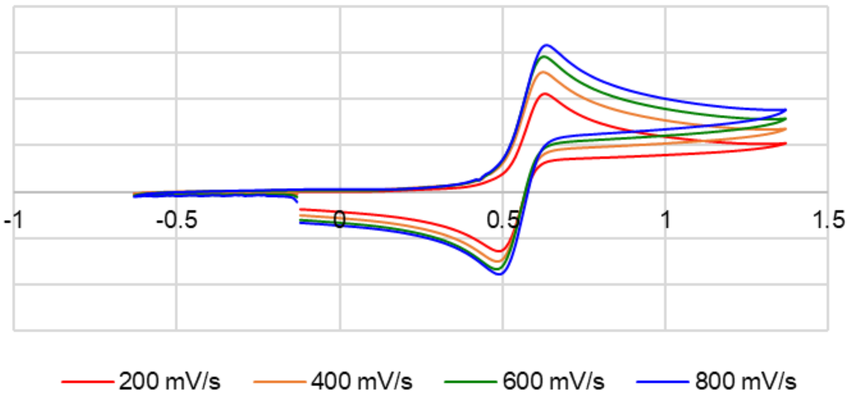


| Scan rate | $E_{1/2}$ |
|---|---|
| 200 mV/s | 0.690 |
| 400 mV/s | 0.700 |
| 600 mV/s | 0.695 |
| 800 mV/s | 0.705 |

**Figure S27.** Narrow CV spectrum of **QQ-PyH** at the oxidative region.

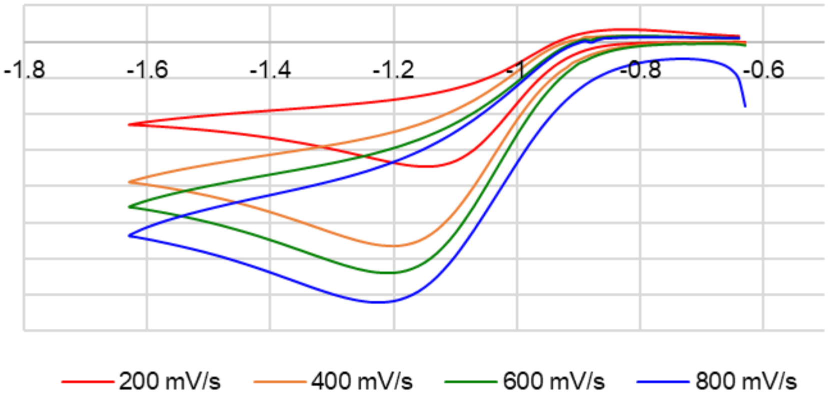


| Scan rate | $E_{pc}$ |
|---|---|
| 200 mV/s | –0.985 |
| 400 mV/s | –1.005 |
| 600 mV/s | –1.015 |
| 800 mV/s | –1.025 |

**Figure S28.** Narrow CV spectrum of **QQ-PyH** at the reductive region.

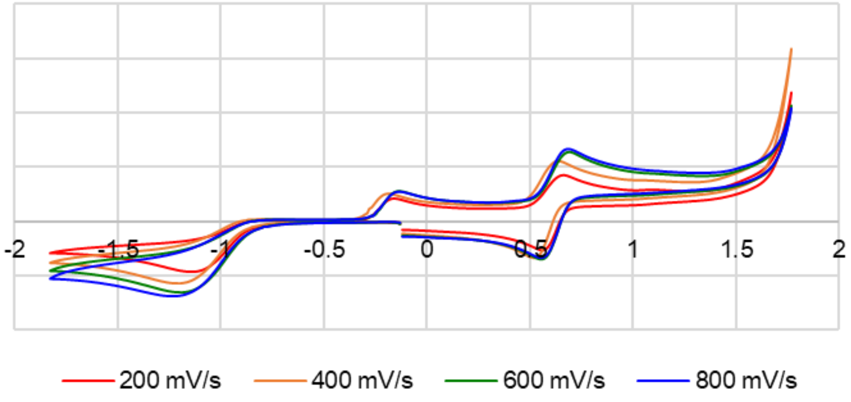


**Figure S29.** Full CV spectrum of **QQ-PyMe**.

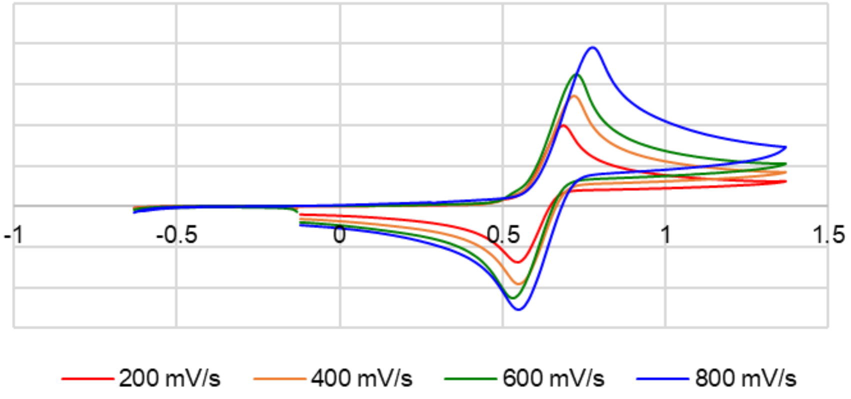


| Scan rate | $E_{1/2}$ |
|---|---|
| 200 mV/s | 0.615 |
| 400 mV/s | 0.630 |
| 600 mV/s | 0.625 |
| 800 mV/s | 0.660 |

**Figure S30.** Narrow CV spectrum of **QQ-PyMe** at the oxidative region.

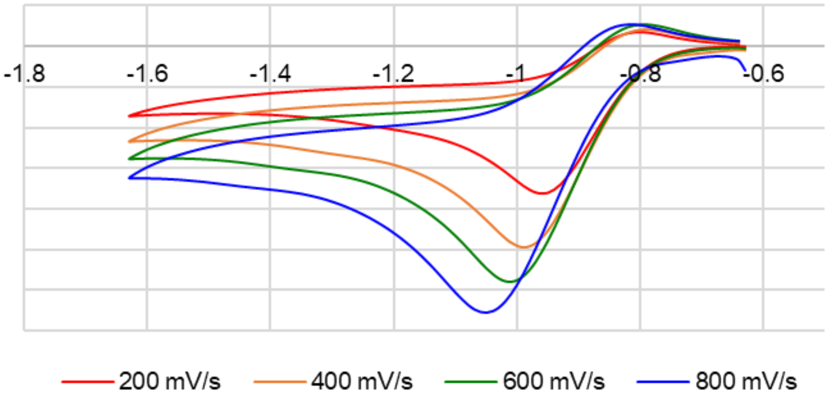


| Scan rate | $E_{pc}$ |
|---|---|
| 200 mV/s | –0.960 |
| 400 mV/s | –0.990 |
| 600 mV/s | –1.010 |
| 800 mV/s | –1.050 |

**Figure S31.** Narrow CV spectrum of **QQ-PyMe** at the reductive region.

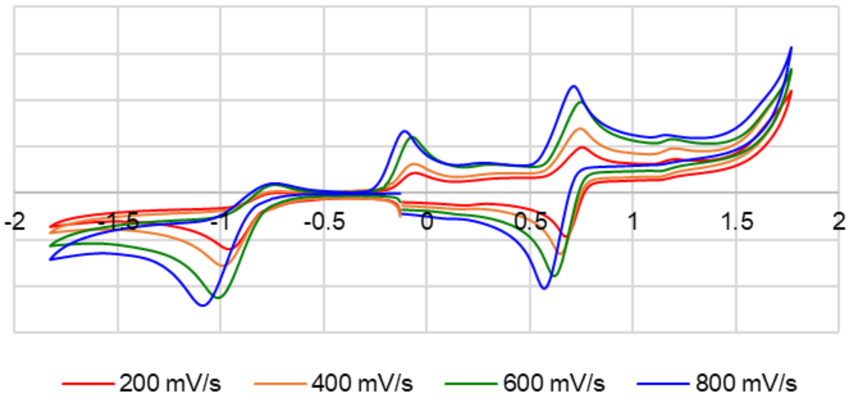


**Figure S32.** Full CV spectrum of **QQ-PyCl**.

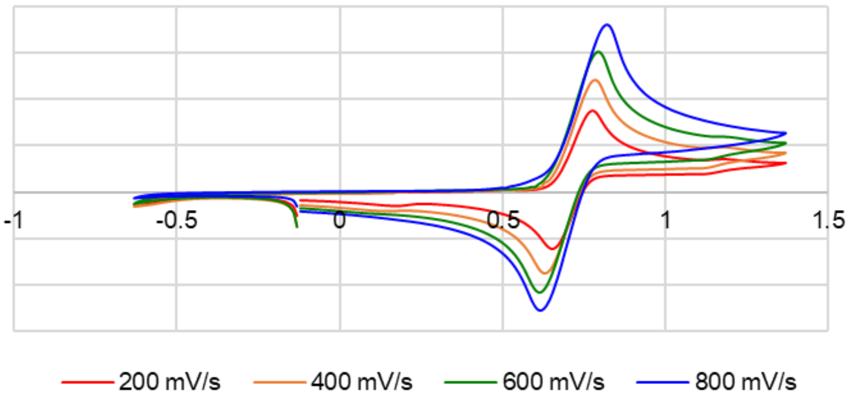


| Scan rate | $E_{1/2}$ |
|---|---|
| 200 mV/s | 0.715 |
| 400 mV/s | 0.705 |
| 600 mV/s | 0.700 |
| 800 mV/s | 0.720 |

**Figure S33.** Narrow CV spectrum of **QQ-PyCl** at the oxidative region.

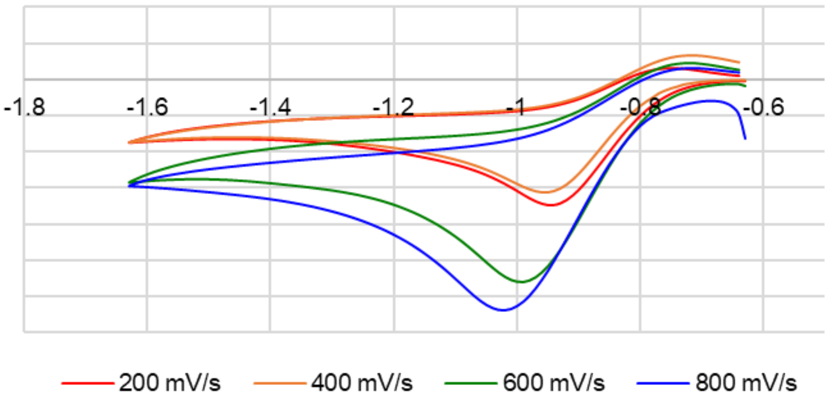


| Scan rate | $E_{pc}$ |
|---|---|
| 200 mV/s | –0.950 |
| 400 mV/s | –0.960 |
| 600 mV/s | –0.990 |
| 800 mV/s | –1.020 |

**Figure S34.** Narrow CV spectrum of **QQ-PyCl** at the reductive region.

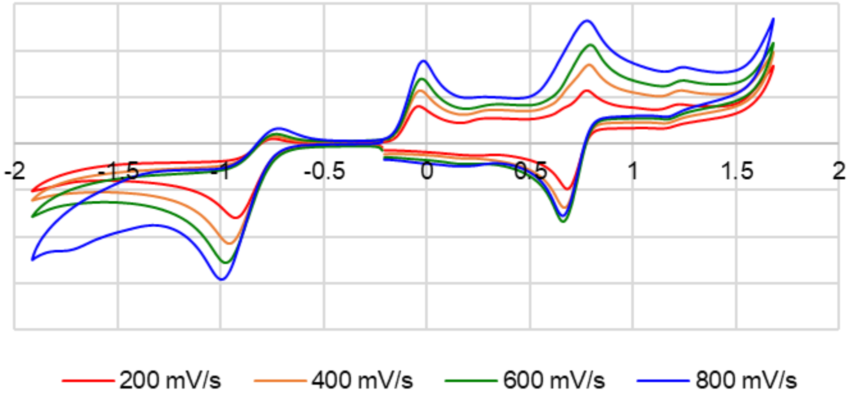


**Figure S35.** Full CV spectrum of **QQ-PyCF₃**.

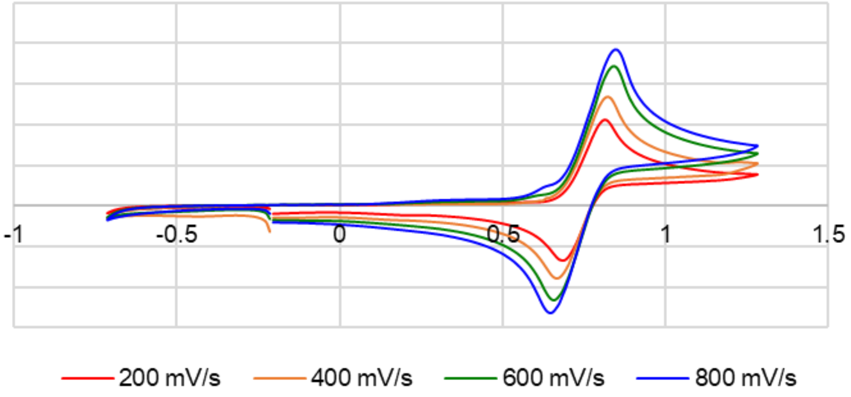


| Scan rate | $E_{1/2}$ |
|---|---|
| 200 mV/s | 0.750 |
| 400 mV/s | 0.746 |
| 600 mV/s | 0.750 |
| 800 mV/s | 0.745 |

**Figure S36.** Narrow CV spectrum of **QQ-PyCF₃** at the oxidative region.

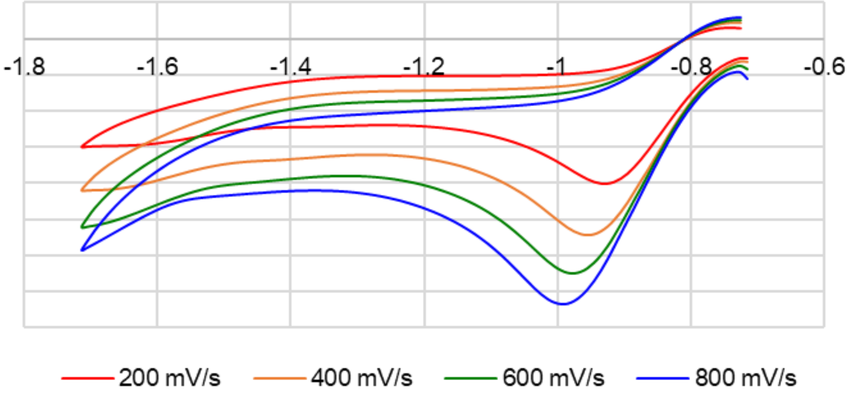


| Scan rate | $E_{pc}$ |
|---|---|
| 200 mV/s | –0.925 |
| 400 mV/s | –0.955 |
| 600 mV/s | –0.985 |
| 800 mV/s | –1.005 |

**Figure S37.** Narrow CV spectrum of **QQ-PyCF₃** at the reductive region.

**III-4. Redox potential of excited photocatalytic species**

Redox potential of excited photocatalytic species was calculated following the approximation provided by Nicewicz.[8] Thus, the excited reduction potential of **QQ-Ar**, $E_{Red}$*, was calculated by:

$E^*_{Red}$ (**QQ-Ar*/QQ-Ar**$^{•–}$) = $E_{Red}$ (**QQ-Ar/QQ-Ar**$^{•–}$) + $E_{0,0}$

where $E_{0,0}$ is approximated to $hc/\lambda_{em,max}$.

Because of the high irreversibility of the reduction, $E^*_{Red,pc}$ (**QQ-Ar*/QQ-Ar**$^{•–}$) was calculated using the cathodic peak potential $E_{Red,pc}$ (**QQ-Ar/QQ-Ar**$^{•–}$) at 800 mV/s scan rate, which is lower than the half reduction potential. Therefore, the actual $E^*_{Red,1/2}$ (**QQ-Ar*/QQ-Ar**$^{•–}$) is higher than calculated $E^*_{Red,pc}$ (**QQ-Ar*/QQ-Ar**$^{•–}$).

On the other hand, the excited oxidation potential, $E_{Ox}$*, was calculated by:

$E^*_{Ox}$ (**QQ-Ar**$^{•+}$/**QQ-Ar***) = $E_{Ox}$ (**QQ-Ar**$^{•+}$/**QQ-Ar***) – $E_{0,0}$

In this case, due to high reversibility of the oxidation, $E^*_{Ox,1/2}$ (**QQ-Ar**$^{•+}$/**QQ-Ar***) was calculated using the half potential $E_{Ox,1/2}$ (**QQ-Ar**$^{•+}$/**QQ-Ar***) at 800 mV/s scan rate.

| QQ-Ar | $\lambda_{em,max}$ (nm) | $E_{0,0}$ (eV) | $E_{1/2}$(PC$^{•+}$/PC) | $E_{1/2}$(PC$^{•+}$/PC*) | $E_{pc}$(PC/PC$^{•–}$) | $E_{pc}$(PC*/PC$^{•–}$) |
|---|---|---|---|---|---|---|
| **QQ-Ph** | 481 | 2.58 | 0.635 | −1.95 | −1.23 | 1.35 |
| **QQ-Mes** | 476 | 2.61 | 0.560 | −2.05 | −1.08 | 1.53 |
| **QQ-PyMe** | 483 | 2.57 | 0.665 | −1.91 | −1.05 | 1.70 |
| **QQ-PyH** | 484 | 2.56 | 0.705 | −1.86 | −1.02 | 1.54 |
| **QQ-PyCl** | 488 | 2.54 | 0.720 | −1.82 | −1.02 | 1.52 |
| **QQ-PyCF$_3$** | 493 | 2.52 | 0.760 | −1.76 | −0.97 | 1.55 |

**Table S2.** Redox potential of excited photocatalytic species.

### IV. Reaction parameter variation

To a 4 mL vial equipped with a stir bar were added 4-methoxybenzamide (0.1 mmol), DMAP (0.01 mmol), **QQ-Ar** (0.01 mmol), $CCl_3Br$ (0.2 mmol) and solvent (0.25M) in glove box. The mixture was stirred for 4 h with irradiation of blue LED (440 nm, 30 W) using a Kessil lamp. The reaction mixture was diluted with EtOAc and brine, and the combined organic layer was dried over $MgSO_4$ then concentrated under reduced pressure. The crude yield was determined by $^1H$ NMR spectroscopy using dibromomethane as an internal standard in $CDCl_3$.

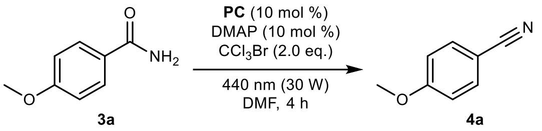


| Entry | Photocatalyst | Yield (%) |
|---|---|---|
| 1 | (none) | 0 |
| 2 | **QQ-Ph** | 97 |
| 3 | **QQ-Mes** | 96 |
| 4 | **QQ-PyH** | >99 |
| 5 | **QQ-PyMe** | 91 |
| 6 | **QQ-PyCl** | 95 |
| 7 | **QQ-PyCF$_3$** | 97 |
| 8 | Ir(ppy)$_3$ | trace < 5% |

**Table S3.** Effect of photocatalyst variation on the nitrilization reactivity.

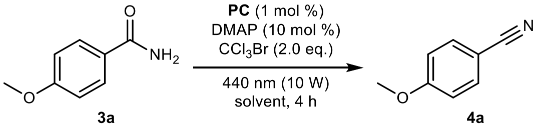


| Entry | Photocatalyst | Yield (%) |
|---|---|---|
| 1 | **QQ-Ph** | 9 |
| 2 | **QQ-Mes** | 29 |
| 3 | **QQ-PyH** | 7 |
| 4 | **QQ-PyMe** | 3 |
| 5 | **QQ-PyCl** | 13 |
| 6 | **QQ-PyCF$_3$** | 10 |

**Table S4.** Effect of photocatalyst variation on the nitrilization with low catalyst loading.

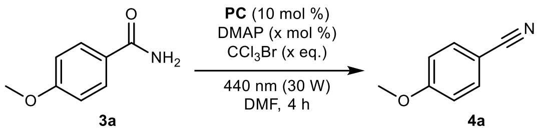


| Entry | PC | Additive control | Yield (%) |
| --- | --- | --- | --- |
| 1 | **QQ-PyH** | without $CCl_3Br$ | 0 |
| 2 | **QQ-PyH** | without DMAP | 90 |
| 3 | **QQ-Ph** | without DMAP | 38 |

**Table S5.** Effect of $CCl_3Br$ and DMAP additive on the nitrilization reactivity.

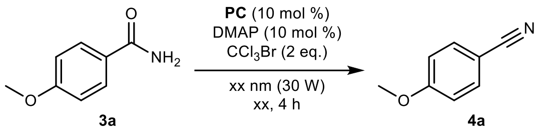


| Entry | Reaction environment | Yield (%) |
| --- | --- | --- |
| 1 | without light, 80 °C | 0 |
| 2 | 525 nm, 30W Kessil green light | 15 |
| 3 | MeCN solvent | 25 |
| 4 | $PhCF_3$ solvent | 36 |

**Table S6.** Effect of reaction environment on the nitrilization reactivity.

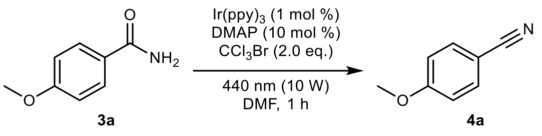


| Entry | solvent | Yield (%) | Conversion (%) |
| --- | --- | --- | --- |
| 1 | DMF | 61 | 100 |
| 2 | $CHCl_3$ | 9 | 44 |
| 3 | MeCN | 18 | 32 |
| 4 | THF | <5 | 58 |
| 5 | MeOH | <5 | 4 |
| 6 | $CF_3Ph$ | 7 | 45 |

**Table S7.** Effect of photocatalyst variation on the nitrilization reactivity.

## V. QQ-Ar-catalyzed direct amide nitrilization

### V-1. Reaction substrate scope

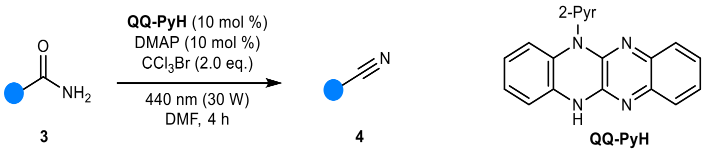


To a 4 mL vial equipped with a stir bar were added amide (1 equiv), DMAP (10 mol%), **QQ-PyH** (10 mol%), $CCl_3Br$ (2 equiv) and DMF (0.25M) in glove box. The mixture was stirred for 4 h with irradiation of blue LED (440 nm, 30 W) by using Kessil lamp. The reaction mixture was diluted with EtOAc and brine, and the combined organic layer was dried over $MgSO_4$ then concentrated under reduced pressure. The residue was purified by $SiO_2$ column chromatography to obtain the product.

4-Methoxybenzonitrile (**4a**)

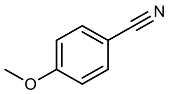

12.7 mg, 95%; yellow solid; $R_f$ = 0.40 (EtOAc/Hx = 1:9); $^1$H NMR (400 MHz, $CDCl_3$) δ 7.58 (d, $J$ = 8.9 Hz, 2H), 6.95 (d, $J$ = 8.9 Hz, 2H), 3.86 (s, 3H). $^{13}$C NMR (101 MHz, $CDCl_3$) δ 163.0, 134.1, 119.4, 114.9, 104.1, 55.7.

4-Methylbenzonitrile (**4b**) (0.2 mmol scale)

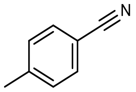

17.5 mg, 75%; solid/oil; $R_f$ = 0.72 (EtOAc/Hx = 1:10); $^1$H NMR (400 MHz, $CD_2Cl_2$) δ 7.55 (d, $J$ = 8.2 Hz, 2H), 7.29 (d, $J$ = 7.9 Hz, 2H), 2.42 (s, 3H); $^{13}$C NMR (101 MHz, $CD_2Cl_2$) δ 144.3, 132.4, 130.2, 119.5, 109.7, 22.0.

4-Bromobenzonitrile (**4c**)

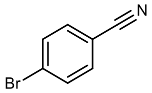

15.9 mg, 87%; white solid; $R_f$ = 0.65 (EtOAc/Hx = 1:10); $^1$H NMR (600 MHz, $CDCl_3$) δ 7.63 (d, $J$ = 8.6 Hz, 2H), 7.52 (d, $J$ = 8.6 Hz, 2H); $^{13}$C NMR (151 MHz, $CDCl_3$) δ 133.5, 132.8, 128.1, 118.2, 111.4.

4-Iodobenzonitrile (**4d**)

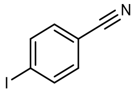

21.0 mg, 92%; white solid; $R_f$ = 0.68 (EtOAc/Hx = 1:10); $^1$H NMR (600 MHz, $CDCl_3$) δ 7.84 (d, $J$ = 8.5 Hz, 2H), 7.36 (d, $J$ = 8.5 Hz, 2H); $^{13}$C NMR (151 MHz, $CDCl_3$) δ 138.6, 133.3, 118.3, 111.9, 100.4.

4-(Methylsulfonyl)benzonitrile (**4e**)

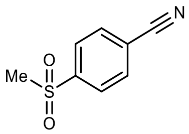

15.7 mg, 87%; colorless solid; $R_f$ = 0.17 (EtOAc/Hx = 1:3); $^1$H NMR (500 MHz, $CDCl_3$) δ 8.08 (d, *J* = 8.4 Hz, 2H), 7.89 (d, *J* = 8.5 Hz, 2H), 3.09 (s, 3H). $^{13}$C NMR (126 MHz, $CDCl_3$) δ 144.6, 133.3, 128.3, 117.8, 117.2, 44.4.

4-Hydroxybenzonitrile (**4f**)

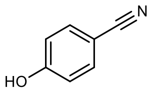

11.5 mg, 97%; yellow solid; $R_f$ = 0.30 (EtOAc/Hx = 1:3); $^1$H NMR (500 MHz, $CDCl_3$) δ 7.55 (d, *J* = 8.7 Hz, 2H), 6.93 (d, *J* = 8.7 Hz, 2H), 6.58 (br. s, 1H). $^{13}$C NMR (126 MHz, $CDCl_3$) δ 160.2, 134.5, 119.4, 116.6, 103.4.

4-Nitrobenzonitrile (**4g**)

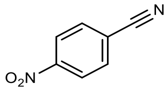

13.6 mg, 93%; white solid; $R_f$ = 0.67 (EtOAc/Hx = 1:3); $^1$H NMR (500 MHz, $CDCl_3$) δ 8.36 (d, *J* = 8.9 Hz, 2H), 7.89 (d, *J* = 8.9 Hz, 2H). $^{13}$C NMR (126 MHz, $CDCl_3$) δ 150.2, 133.6, 124.4, 118.5, 116.9.

9*H*-Fluorene-1-carbonitrile (**4h**)[9]

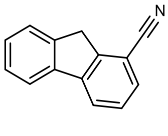

17.1 mg, 89%; white solid; $R_f$ = 0.65 (EtOAc/Hx = 1:9); $^1$H NMR (400 MHz, $CDCl_3$) δ 7.96 (dd, *J* = 7.7, 1.1 Hz, 1H), 7.79 (d, *J* = 6.5 Hz, 1H), 7.59 (d, *J* = 1.1 Hz, 1H), 7.57 (dd, *J* = 7.7, 1.1 Hz, 1H), 7.50 – 7.45 (m, 1H), 7.45 – 7.36 (m, 2H), 4.06 (s, 2H); $^{13}$C NMR (101 MHz, $CDCl_3$) δ 147.1, 143.1, 142.4, 140.1, 129.8, 128.2, 127.8, 127.4, 125.4, 124.1, 120.5, 117.8, 109.5, 36.8.

3-(Pyridin-2-yl)benzonitrile (**4i**)

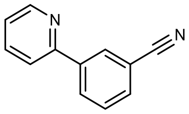

16.4 mg, 91%; yellow solid; $R_f$ = 0.30 (acetone/Hx = 1:9); $^1$H NMR (400 MHz, acetone-$d_6$) δ 8.72 (ddd, *J* = 4.8, 1.9, 1.0 Hz, 1H), 8.50 (dd, *J* = 1.8, 1.8 Hz, 1H), 8.45 (ddd, *J* = 8.0, 1.5, 1.5 Hz, 1H), 8.06 (ddd, *J* = 8.0, 1.1, 1.1 Hz, 1H), 7.93 (ddd, *J* = 7.8, 7.8, 1.8 Hz, 1H), 7.83 (ddd, *J* = 7.7, 1.4, 1.4 Hz, 1H), 7.72 (dd, *J* = 7.8, 7.8 Hz, 1H), 7.41 (ddd, *J* = 7.5, 4.7, 1.1 Hz, 1H). $^{13}$C NMR (101 MHz, acetone-$d_6$) δ 155.3, 150.8, 141.3, 138.2, 133.1, 131.8, 131.0, 130.8, 124.2, 121.3, 119.3, 113.7.

Picolinonitrile (**4j**)

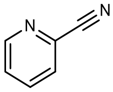

8.5 mg, 82%; yellow oil; $R_f$ = 0.20 (EtOAc/Hx = 1:9); $^1$H NMR (400 MHz, $CDCl_3$) δ 8.73 (dd, *J* = 4.8, 0.8 Hz, 1H), 7.85 (ddd, *J* = 7.8,7.8, 1.7 Hz, 1H), 7.71 (ddd, *J* = 7.8, 1.1, 1.1 Hz, 1H), 7.53 (ddd,

$J$ = 7.8, 4.9, 1.2 Hz, 1H). $^{13}$C NMR (126 MHz, $CDCl_3$) δ 151.3, 137.2, 134.2, 128.7, 127.0, 117.3.

2-Chloronicotinonitrile (**4k**)

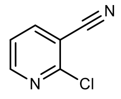

7.2 mg, 52%; yellow oil; $R_f$ = 0.37 (EtOAc/Hx = 1:4); $^1$H NMR (400 MHz, $CDCl_3$) δ 8.61 (dd, $J$ = 4.9, 2.0 Hz, 1H), 8.01 (dd, $J$ = 7.7, 2.0 Hz, 1H), 7.39 (dd, $J$ = 7.7, 4.9 Hz, 1H); $^{13}$C NMR (101 MHz, $CDCl_3$) δ 153.1, 153.0, 142.7, 122.3, 114.7, 111.2.

Quinoline-6-carbonitrile (**4l**)

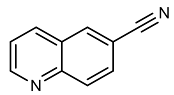

8.7 mg, 56%; off-white solid; $R_f$ = 0.17 (acetone/Hx = 1:9); $^1$H NMR (500 MHz, $CDCl_3$) δ 9.06 (dd, $J$ = 4.2, 1.8 Hz, 1H), 8.25 (d, $J$ = 2.0 Hz, 1H), 8.25 (dd, $J$ = 8.4, 1.6 Hz, 1H), 8.22 (d, $J$ = 8.8 Hz, 1H), 7.87 (dd, $J$ = 8.8, 1.8 Hz, 1H), 7.56 (dd, $J$ = 8.3, 4.2 Hz, 1H). $^{13}$C NMR (126 MHz, $CDCl_3$) δ 153.2, 149.0, 136.7, 134.3, 131.1, 130.4, 127.7, 122.9, 118.6, 110.7.

1*H*-Indole-3-carbonitrile (**4m**)

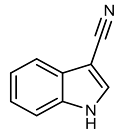

8.6 mg, 60%; white solid; $R_f$ = 0.23 (EtOAc/Hx = 1:4); $^1$H NMR (400 MHz, $CDCl_3$) δ 8.78 (br. s, 1H), 7.79 (d, $J$ = 7.3 Hz, 1H), 7.74 (d, $J$ = 2.9 Hz, 1H), 7.48 (dd, $J$ = 7.3, 1.7 Hz, 1H), 7.38 – 7.28 (m, 2H); $^{13}$C NMR (101 MHz, $CDCl_3$) δ 135.0, 132.0, 127.1, 124.5, 122.6, 119.9, 116.0, 112.2, 87.7.

Terephthalonitrile (**4n**)

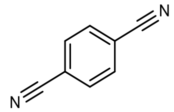

9.5 mg, 74%; white solid; $R_f$ = 0.34 (EtOAc/Hx = 1:10); $^1$H NMR (600 MHz, $CDCl_3$) δ 7.80 (s, 4H); $^{13}$C NMR (151 MHz, $CDCl_3$) δ 132.93, 117.14, 116.87.

4-Chloropyridine-2,6-dicarbonitrile (**4o**)

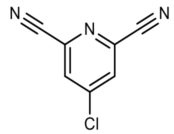

15.1 mg, 92%; yellow solid; $R_f$ = 0.27 (acetone/Hx = 1:9); $^1$H NMR (500 MHz, $CDCl_3$) δ 7.92 (s, 2H). $^{13}$C NMR (126 MHz, $CDCl_3$) δ 147.4, 136.1, 131.6, 114.7.

2-Oxo-2*H*-chromene-3-carbonitrile (**4p**)

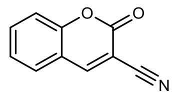

13.1 mg, 77%; off-white solid; $R_f$ = 0.10 (acetone/Hx = 1:9); $^1$H NMR (400 MHz, $CDCl_3$) δ 8.29 (s, 1H), 7.73 (ddd, $J$ = 8.7, 7.3, 1.6 Hz, 1H), 7.62 (dd, $J$ = 8.0, 1.6 Hz, 1H), 7.42 (dd, $J$ = 7.7, 4.8 Hz, 2H). $^{13}$C NMR (101 MHz, $CDCl_3$) δ 156.6, 154.7, 152.0, 135.7, 129.4, 125.9, 117.6, 117.3, 113.7,

103.5.

Cinnamonitrile (**4q**) (0.2 mmol scale)

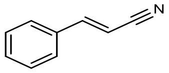

14.7 mg, 57% (E/Z = 93:7).; yellow oil; $R_f$ = 0.73 (EtOAc/Hx = 1:3); $^1$H NMR (400 MHz, $CDCl_3$) δ 7.48 – 7.35 (m, 6H), 5.88 (d, $J$ = 16.8 Hz, 1H). $^{13}$C NMR (101 MHz, $CDCl_3$) δ 150.7, 133.7, 131.4, 127.5, 118.3, 96.5.

3-(4-Bromophenyl)propanenitrile (**4r**)

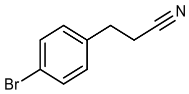

20.3 mg, 97%; yellow oil; $R_f$ = 0.53 (EtOAc/Hx = 1:3); $^1$H NMR (400 MHz, $CDCl_3$) δ 7.47 (d, $J$ = 8.4 Hz, 2H), 7.12 (d, $J$ = 8.4 Hz, 2H), 2.92 (t, $J$ = 7.3 Hz, 2H), 2.61 (t, $J$ = 7.3 Hz, 2H). $^{13}$C NMR (101 MHz, $CDCl_3$) δ 137.1, 132.2, 130.2, 121.4, 118.9, 31.1, 19.4.

2-(*p*-Tolyloxy)acetonitrile (**4s**)

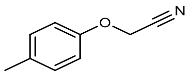

12.9 mg, 88%; yellow oil; $R_f$ = 0.63 (EtOAc/Hx = 1:3); $^1$H NMR (400 MHz, $CDCl_3$) δ 7.15 (d, $J$ = 8.1 Hz, 2H), 6.89 (d, $J$ = 8.7 Hz, 2H), 4.73 (s, 2H), 2.32 (s, 3H). $^{13}$C NMR (101 MHz, $CDCl_3$) δ 154.7, 132.8, 130.5, 115.4, 115.2, 54.1, 20.7.

*N*-(Cyanomethyl)-2-phenylacetamide (**4t**)[10]

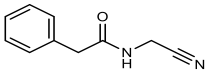

7.7 mg, 44%; off-white solid; $R_f$ = 0.10 (EtOAc/Hx = 1:3); $^1$H NMR (500 MHz, $CDCl_3$) δ 7.38 (dd, $J$ = 7.1, 7.1 Hz, 2H), 7.33 (dd, $J$ = 6.9, 6.9 Hz, 1H), 7.25 (d, $J$ = 6.4 Hz, 2H), 5.92 (br. s, 1H), 4.11 (d, $J$ = 5.7 Hz, 2H), 3.63 (s, 2H). $^{13}$C NMR (126 MHz, $CDCl_3$) δ 171.1, 133.7, 129.6, 129.4, 128.0, 116.0, 43.3, 27.7.

2-(Benzo[*d*][1,3]dioxol-5-yl)acetonitrile (**4u**)

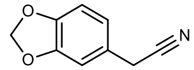

14.5 mg, 90%; yellow oil; $R_f$ = 0.53 (EtOAc/Hx = 1:3); $^1$H NMR (500 MHz, $CDCl_3$) δ 6.81 – 6.75 (m, 3H), 5.98 (s, 2H), 3.65 (s, 2H). $^{13}$C NMR (126 MHz, $CDCl_3$) δ 148.4, 147.6, 123.4, 121.4, 118.1, 108.8, 108.5, 101.5, 23.5.

1-Phenylcyclopropane-1-carbonitrile (**4v**)

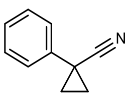

13.6 mg, 95%; colorless oil; $R_f$ = 0.47 (EtOAc/Hx = 1:9); $^1$H NMR (400 MHz, $CDCl_3$) δ 7.37 – 7.22 (m, 5H), 1.72 – 1.67 (m, 2H), 1.41 – 1.36 (m, 2H). $^{13}$C NMR (101 MHz, $CDCl_3$) δ 136.1, 129.0, 127.7, 125.9, 122.7, 29.8, 18.3, 13.9.

Spiro[3.3]heptane-2-carbonitrile (**4w**) (0.2 mmol scale)

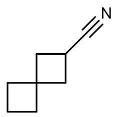

18.0 mg, 74%; yellow oil; $R_f$ = 0.97 (Hx); $^1$H NMR (400 MHz, $CD_2Cl_2$) δ 2.93 (p, *J* = 8.3 Hz, 1H), 2.42 – 2.27 (m, 4H), 2.06 – 1.96 (m, 4H), 1.86 – 1.75 (m, 2H). $^{13}$C NMR (101 MHz, $CD_2Cl_2$) δ 123.2, 42.2, 39.6, 34.8, 17.6, 16.4.

2,2,2-Triphenylacetonitrile (**4x**)

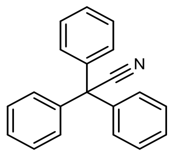

23.5 mg, 87%; white solid; $R_f$ = 0.72 (EtOAc/Hx = 1:10); $^1$H NMR (600 MHz, $CDCl_3$) δ 7.39 – 7.32 (m, 9H), 7.23 (dd, *J* = 7.9, 1.8 Hz, 6H); $^{13}$C NMR (151 MHz, $CDCl_3$) δ 140.3, 129.0, 128.8, 128.3, 123.6, 57.6.

4-Cyano-*N*,*N*-dipropylbenzenesulfonamide (**4y**)[11]

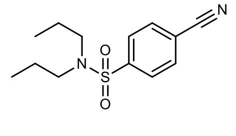

22.8 mg, 86%; yellow oil; $R_f$ = 0.60 (EtOAc/Hx = 1:3); $^1$H NMR (500 MHz, $CDCl_3$) δ 7.91 (d, *J* = 8.5 Hz, 2H), 7.80 (d, *J* = 8.5 Hz, 2H), 3.12 – 3.07 (m, 4H), 1.49 – 1.59 (m, 4H), 0.86 (t, *J* = 7.4 Hz, 6H). $^{13}$C NMR (126 MHz, CDCl3) δ 144.7, 133.0, 127.7, 117.5, 116.1, 50.0, 22.0, 11.2.

2-(4-Cyano-3-ethoxyphenyl)-*N*-{3-methyl-1-[2-(piperidin-1-yl)phenyl]butyl}ccacetamide (**4z**)[12]

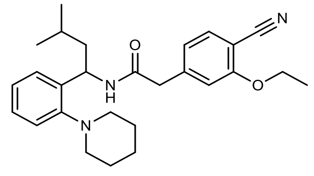

30.8 mg, 71%; yellow solid; $R_f$ = 0.23 (EtOAc/Hx = 1:4); $^1$H NMR (400 MHz, $CDCl_3$) δ 7.46 (d, *J* = 7.9 Hz, 1H), 7.22 (dd, *J* = 5.9, 1.8 Hz, 2H), 7.13 – 7.09 (m, 1H), 7.06 (ddd, *J* = 7.8, 6.0, 2.4 Hz, 1H), 6.95 (d, *J* = 9.5 Hz, 1H), 6.86 (s, 1H), 6.84 (d, *J* = 7.9 Hz, 1H), 5.34 (td, *J* = 8.6, 6.5 Hz, 1H), 4.04 (dtt, *J* = 16.4, 9.4, 7.1 Hz, 2H), 3.52 (d, *J* = 2.4 Hz, 2H), 2.99 – 2.87 (m, 2H), 2.63 (t, *J* = 10.0 Hz, 2H), 1.80 – 1.66 (m, 3H), 1.66 – 1.48 (m, 5H), 1.47 – 1.36 (m, 4H), 0.91 (dd, *J* = 6.6, 3.9 Hz, 6H); $^{13}$C NMR (101 MHz, $CDCl_3$) δ 168.2, 160.9, 152.6, 142.7, 138.6, 133.9, 128.2, 128.1, 125.4, 123.2, 121.5, 116.6, 113.0, 100.8, 64.8, 50.4, 46.8, 44.4, 26.9, 25.5, 24.3, 22.9, 22.7, 14.6.

1-Ethyl-7-methyl-4-oxo-1,4-dihydro-1,8-naphthyridine-3-carbonitrile (**4aa**)

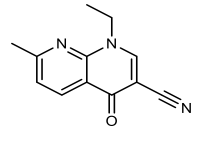

17.5 mg, 82%; yellow solid; $R_f$ = 0.52 (EtOAc/Hx = 1:1); m.p. 215 – 217 °C; IR(diamond) 3063, 2982, 2222, 1628, 1585, 1439, 1362, 1269, 791 cm$^{-1}$; $^1$H NMR (400 MHz, $CDCl_3$) δ 8.56 (dd, *J* = 8.1, 1.0 Hz, 1H), 8.21 (d, *J* = 1.0 Hz, 1H), 7.29 (d, *J* = 8.1 Hz, 1H), 4.50 (q, *J* = 7.6 Hz, 2H), 2.67 (d, *J* = 1.1 Hz, 3H), 1.50 (t, *J* = 6.7 Hz, 3H); $^{13}$C NMR (101 MHz, $CDCl_3$) δ 175.1, 164.0, 148.7, 148.4,

136.4, 122.0, 119.8, 115.5, 97.0, 47.1, 25.3, 15.3; HRMS (ESI) m/z: $[M+H]^+$ Calcd for $C_{12}H_{12}N_3O$: 214.0975, found: 214.0983.

5-(2,5-Dimethylphenoxy)-2,2-dimethylpentanenitrile (**4ab**)[11]

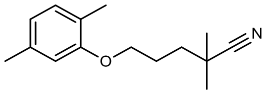

19.7 mg, 85%; colorless oil; $R_f$ = 0.58 (EtOAc/Hx = 1:9); $^1$H NMR (400 MHz, $CDCl_3$) δ 7.01 (d, $J$ = 8.1 Hz, 1H), 6.68 (d, $J$ = 7.5 Hz, 1H), 6.62 (s, 1H), 3.99 (t, $J$ = 6.0 Hz, 2H), 2.32 (s, 3H), 2.18 (s, 3H), 2.04 – 1.95 (m, 2H), 1.79 – 1.72 (m, 2H), 1.40 (s, 6H); $^{13}$C NMR (101 MHz, $CDCl_3$) δ 156.9, 136.7, 130.5, 125.1, 123.7, 121.1, 112.1, 67.4, 38.0, 32.4, 26.8, 25.7, 21.5, 15.9.

(*S*)-2-(4-Isobutylphenyl)propanenitrile (**4ac**)

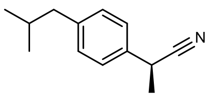

15.3 mg, 82%; colorless oil; $R_f$ = 0.6 (EtOAc/Hx = 1:9); $^1$H NMR (500 MHz, $CDCl_3$) δ 7.25 (d, $J$ = 8.2 Hz, 2H), 7.16 (d, $J$ = 8.3 Hz, 2H), 3.87 (q, $J$ = 7.3 Hz, 1H), 2.47 (d, $J$ = 7.2 Hz, 2H), 1.91 – 1.79 (m, 1H), 1.63 (d, $J$ = 7.3 Hz, 3H), 0.90 (d, $J$ = 6.6 Hz, 6H). $^{13}$C NMR (126 MHz, $CDCl_3$) δ 141.8, 134.4, 129.9, 126.6, 122.0, 45.1, 31.0, 30.3, 22.4, 21.6.

2-[1-(4-Chlorobenzoyl)-5-methoxy-2-methyl-1*H*-indol-3-yl]acetonitrile (**4ad**)[13]

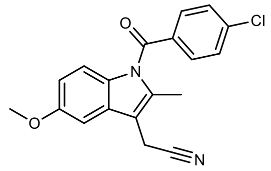

25.9 mg, 76%; off-white solid; $R_f$ = 0.17 (acetone/Hx = 1:9); $^1$H NMR (500 MHz, $CDCl_3$) δ 7.67 (d, $J$ = 8.6 Hz, 2H), 7.49 (d, $J$ = 8.5 Hz, 2H), 6.98 (d, $J$ = 2.5 Hz, 1H), 6.82 (d, $J$ = 9.0 Hz, 1H), 6.71 (dd, $J$ = 9.1, 2.5 Hz, 1H), 3.86 (s, 3H), 3.73 (s, 2H), 2.43 (s, 3H). $^{13}$C NMR (126 MHz, $CDCl_3$) δ 168.3, 156.3, 139.9, 136.1, 133.4, 131.4, 130.8, 129.4, 129.1, 117.1, 115.3, 112.6, 108.1, 100.5, 55.9, 29.8, 13.2.

4-([1,1'-Biphenyl]-4-yl)-4-oxobutanenitrile (**4ae**)[14]

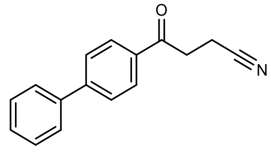

18.6 mg, 79%; yellow solid; $R_f$ = 0.39 (EtOAc/Hx = 1:4); $^1$H NMR (400 MHz, $CDCl_3$) δ 8.03 (d, $J$ = 8.5 Hz, 2H), 7.72 (d, $J$ = 8.4 Hz, 2H), 7.63 (d, $J$ = 7.1 Hz, 2H), 7.49 (t, $J$ = 7.4 Hz, 2H), 7.45 – 7.38 (m, 1H), 3.42 (t, $J$ = 7.2 Hz, 2H), 2.80 (t, $J$ = 7.2 Hz, 2H); $^{13}$C NMR (101 MHz, $CDCl_3$) δ 195.0, 146.8, 139.7, 134.4, 129.2, 128.8, 128.6, 127.6, 127.4, 119.4, 34.5, 12.0.

2-(10-Oxo-10,11-dihydrodibenzo[*b*,*f*]thiepin-3-yl)propanenitrile (**4af**)[15]

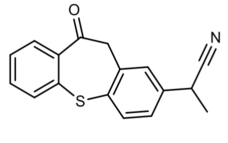

17.6 mg, 63%; colorless oil; $R_f$ = 0.29 (EtOAc/Hx = 1:10); $^1$H NMR (600 MHz, $CDCl_3$) δ 8.20 (d, $J$ = 7.8 Hz, 1H), 7.66 (d, $J$ = 8.0 Hz, 1H), 7.60 (d, $J$ = 7.8 Hz, 1H), 7.47 – 7.40 (m, 2H), 7.33 (t, $J$ = 7.5 Hz, 1H), 7.22 (d, $J$ = 7.6 Hz, 1H), 4.38 (dd, $J$ = 15.6, 11.8 Hz, 1H), 3.90 (q, $J$ = 7.2 Hz, 1H), 1.62 (d, $J$ = 7.3 Hz, 3H); $^{13}$C NMR (151 MHz, $CDCl_3$) δ 191.1, 139.9, 139.2, 138.8, 136.2, 134.7, 132.8, 132.2, 131.7, 131.1, 127.8, 127.2, 125.7, 121.1, 51.1, 31.0, 21.4.

(*R*)-5-(1,2-Dithiolan-3-yl)pentanenitrile (**4ag**) (0.2 mmol scale)

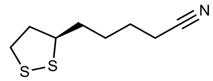


24.9 mg, 67%; yellow oil; $R_f$ = 0.47 (EtOAc/Hx = 1:3); IR (diamond) 2924, 2854, 2245, 1458, 1423, 1312, 1261, 732 cm$^{-1}$; $^1$H NMR (500 MHz, $CDCl_3$) δ 3.57 (dddd, *J* = 8.5, 6.3, 6.3, 6.3 Hz, 1H), 3.19 (ddd, *J* = 11.2, 7.1, 5.4 Hz, 1H), 3.12 (dt, *J* = 11.1, 6.9 Hz, 1H), 2.48 (dddd, *J* = 12.5, 12.5, 6.7, 6.7 Hz, 1H), 2.36 (t, *J* = 6.9 Hz, 2H), 1.92 (dddd, *J* = 13.4, 6.8, 6.8, 6.8 Hz, 1H), 1.77 – 1.52 (m, 6H). $^{13}$C NMR (126 MHz, $CDCl_3$) δ 119.6, 56.1, 40.3, 38.7, 34.2, 28.4, 25.3, 17.2; HRMS (ESI) m/z: $[M+Na]^+$ Calcd for $C_8H_{13}NS_2Na$: 210.0381, found: 210.0391.

5-[(3a*S*,4*S*,6a*R*)-2-oxohexahydro-1*H*-thieno[3,4-d]imidazol-4-yl]pentanenitrile (**4ah**)

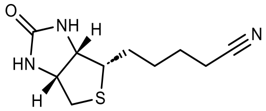


7.6 mg, 34%; yellow solid; $R_f$ = 0.10 (acetone/$CH_2Cl_2$ = 1:4); m.p. 61 – 63 °C; IR (diamond) 3237, 2928, 2862, 2245, 1694, 1238, 1007, 760 cm$^{-1}$; $^1$H NMR (500 MHz, acetone-$d_6$) δ 5.83 (br. s, 1H), 5.69 (br. s, 1H), 4.51 (t, *J* = 7.0 Hz, 1H), 4.36 (t, *J* = 6.3 Hz, 1H), 3.25 (dd, *J* = 7.1, 6.7 Hz, 1H), 2.95 (dd, *J* = 12.8, 4.7 Hz, 1H), 2.72 (d, *J* = 12.5 Hz, 1H), 2.48 (t, *J* = 7.0 Hz, 2H), 2.02 – 1.94 (m, 1H), 1.88 – 1.77 (m, 1H), 1.75 – 1.51 (m, 4H); $^{13}$C NMR (126 MHz, acetone-$d_6$) δ 163.6, 120.6, 62.4, 60.8, 56.3, 41.1, 28.9, 28.8, 26.3, 17.0; HRMS (ESI) m/z: $[M+H]^+$ Calcd for $C_{10}H_{16}N_3OS$: 226.1009, found: 226.1015.

## V-2. Scale-up experiment using ibuprofen derivative substrate

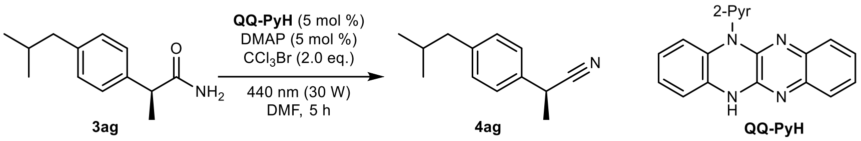


**3ag** 1.03 g, 5.00 mmol

**4ag** 789 mg, 84%

**QQ-PyH**

To a 50 mL round bottom flask equipped with a stir bar were added **3ac** (1.03 g, 5.00 mmol), *N,N*-dimethylaminopyridine (30.5 mg, 0.250 mmol), **QQ-PyH** (77.8 mg, 0.250 mmol), $CCl_3Br$ (1.98 g, 10.0 mmol) and DMF (15 mL) in a glove box. The mixture was stirred for 5 h with irradiation of blue LED (440 nm, 30 W) by using Kessil lamp. The reaction mixture was diluted with pentane (50 mL) and water (250 mL) and the aqueous layer was extracted with pentane (50 mL x 2). The combined organic layer was dried over $MgSO_4$, concentrated under reduced pressure, and the residue was purified by $SiO_2$ column chromatography to obtain the product (789 mg, 84%).

## VI. Mechanistic studies

### VI-1. Stern-Volmer quenching experiment

**QQ-PyH** photocatalyst (1.2 mg, 0.004 mmol) was dissolved in 20 mL of DMF, followed by 10-fold dilution to prepare 20 μM solution. The solution was mixed thoroughly to ensure homogeneity. A 3 mL aliquot of the sample was placed in a cuvette and the fluorescence was observed using 350 nm excitation light over the wavelength range of 400 nm to 650 nm. To the solution were then sequentially added increasing equivalents (1.0, 5.0, 10.0, 15.0, 20.0, and 25.0 equiv) of the quencher. At each quencher concentration, the emission spectrum was recorded, and the fluorescence intensity at the emission maximum ($\lambda_{em,max}$ = 484 nm) was measured. The relative fluorescence intensities ($I_0/I$, where $I_0$ is the intensity in the absence of quencher) were then plotted against the quencher concentration to construct the Stern-Volmer plot. The Stern-Volmer constants were then obtained as the slope, using following equation: $\frac{I_0}{I} = 1 + K_{SV}[Q]$.

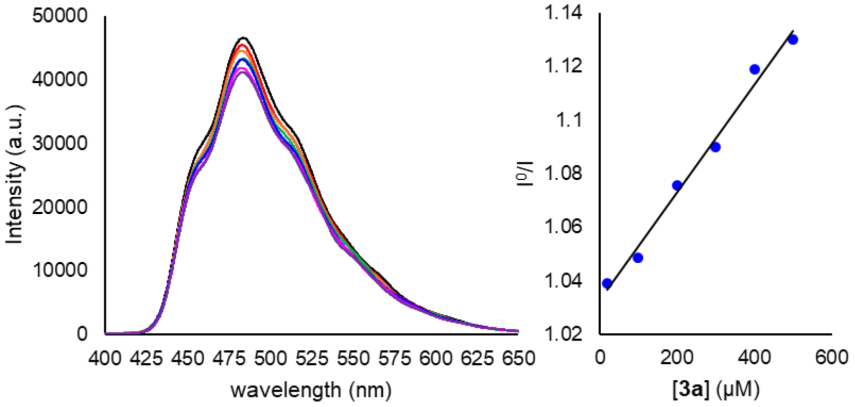


| **3a** (eq.) | [**3a**] (μM) | $I_0/I$ |
|---|---|---|
| 0 | 0 | 1 |
| 1 | 20 | 1.038839 |
| 5 | 100 | 1.048413 |
| 10 | 200 | 1.075391 |
| 15 | 300 | 1.089702 |
| 20 | 400 | 1.118904 |
| $K_{SV}$ = 200.8 $M^{-1}$ | | |

**Figure S38.** Stern-Volmer quenching plot of **QQ-PyH** using **3a** as quencher.

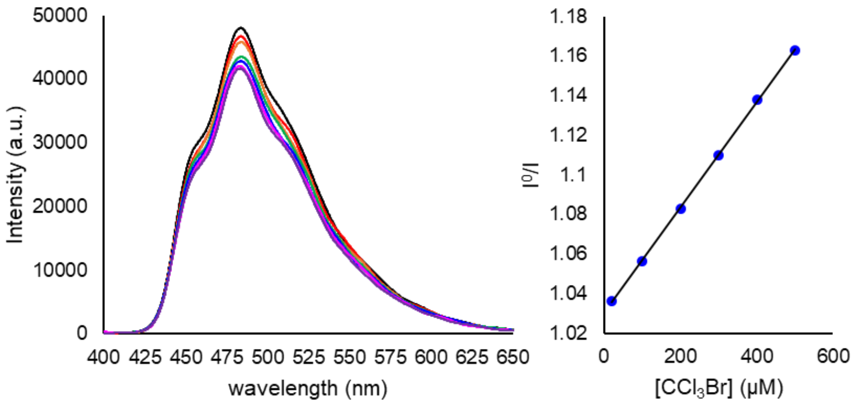


| $CCl_3Br$ (eq.) | [$CCl_3Br$] (μM) | $I_0/I$ |
|---|---|---|
| 0 | 0 | 1 |
| 1 | 20 | 1.036043 |
| 5 | 100 | 1.056305 |
| 10 | 200 | 1.082759 |
| 15 | 300 | 1.109727 |
| 20 | 400 | 1.137696 |
| $K_{SV}$ = 266.6 $M^{-1}$ | | |

**Figure S39.** Stern-Volmer quenching plot of **QQ-PyH** using $CCl_3Br$ as quencher.

### VI-2. NMR spectroscopy for detection of H-bonding complex

### VI-2-1) Chemical shift deviation by catalyst-substrate interaction

To three solutions of **QQ-Mes** (0.70 mg, 0.0020 mmol) in $CD_2Cl_2$ (400 μL) was added $CD_2Cl_2$ (100 μL), a solution of $CCl_3Br$ (2.0 mg, 0.010 mmol) in $CD_2Cl_2$ (100 μL), and a solution of **3a** (1.5 mg, 0.010 mmol) in $CD_2Cl_2$, respectively, to form 4.0 mM photocatalyst solutions. The $^1$H NMR of each sample was measured in a 400 MHz NMR spectrometer with relaxation delay (d1) = 1 sec and scan number = 64 at 300 K.

| Sample | Concentration |
|---|---|
| **A** | **QQ-Mes** (4.0 mM) only |
| **B** | **QQ-Mes** (4.0 mM) + $CCl_3Br$ (20 mM) |
| **C** | **QQ-Mes** (4.0 mM) + **3a** (20 mM) |

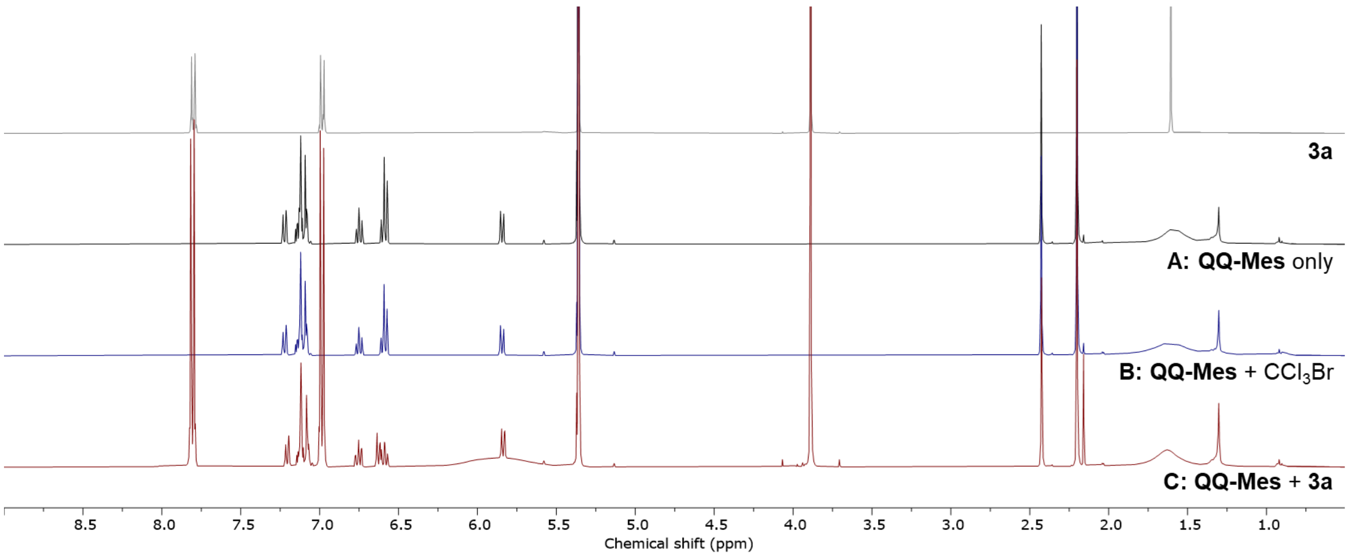


**Figure S40.** Stacked full NMR spectra of photocatalyst (**QQ-Mes**) samples with and without substrates

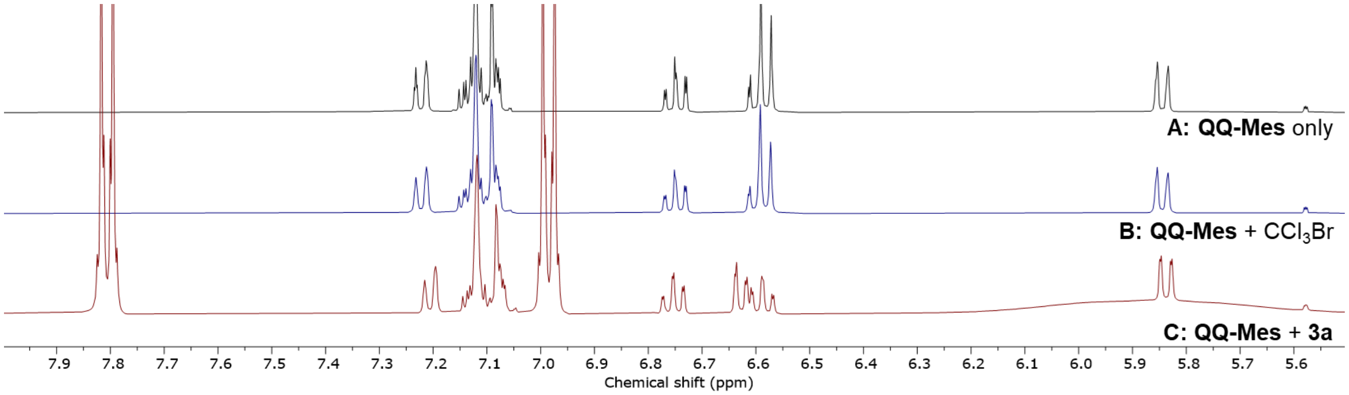


**Figure S41.** Stacked NMR spectra (expanded) of photocatalyst (**QQ-Mes**) samples with and without substrates

**VI-2-2) Nuclear Overhauser effect by catalyst-substrate interaction**

To a solution of QQ-Mes (0.70 mg, 0.0020 mmol) in $CD_2Cl_2$ (400 μL) was added a solution of **3ai** (1.4 mg, 0.010 mmol) in $CD_2Cl_2$ (100 μL) and the solution was thoroughly shaken for 30 sec. The selective NOESY measurement was performed in a 400 MHz NMR spectrometer with following parameters: irradiation frequency = 6.623 ppm; relaxation delay (d1) = 1 sec; mixing time (d8) = 2 sec; scan number = 1000; temperature = 300 K.

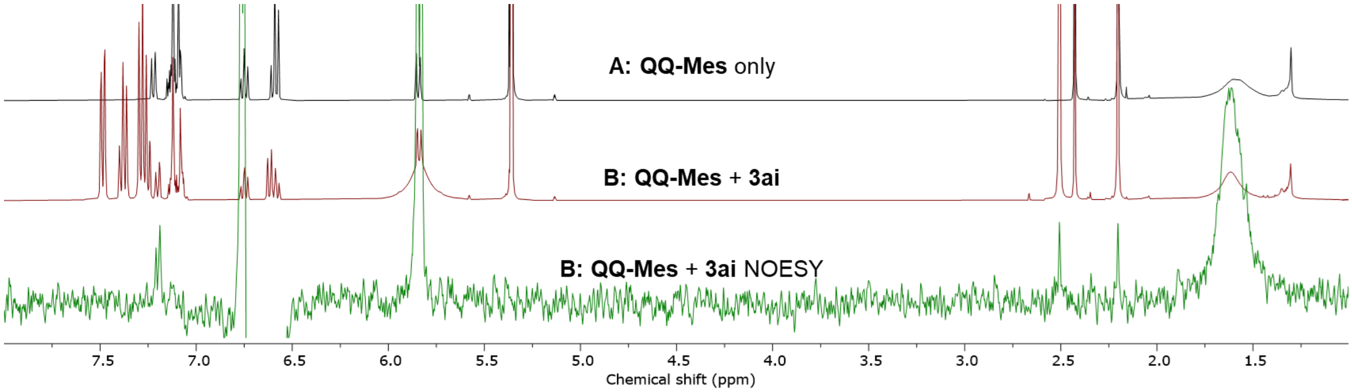


**Figure S42.** Selective $^1$H NOE spectrum of **QQ-Mes** + **3ai** solution.

### VI-3. Catalytic fluorescence quenching of intermediate species

**QQ-PyH** (1.2 mg, 0.004 mmol) was dissolved in 20 mL of DMF, followed by 10-fold dilution to form 20 μM solution. The solution was mixed thoroughly to ensure homogeneity. A 3 mL aliquot of the sample was placed in a 4-way cuvette and a solution of $CCl_3Br$ (2000 uM, 30 μL, 60 nmol) was added. The solution was then irradiated with 440 nm Kessil lamp (30 W) for 10 min then the fluorescence was observed using 350 nm excitation light over the wavelength range of 400 nm to 650 nm. To the solution were then sequentially added increasing equivalents (0.1, 0.2, and 0.3 equiv. to **QQ-PyH**) of **3a**. The emission spectrum was recorded at each **3a** concentration.

| Sample | Concentration |
|---|---|
| **A** | **QQ-PyH** (20 μM) |
| **B** | **QQ-PyH** (20 μM) + $CCl_3Br$ (x μM), 10 min irradiation with 440 nm lamp (30 W) |
| **C** | **B** + **3a** (2.0 μM) |
| **D** | **B** + **3a** (4.0 μM) |
| **E** | **B** + **3a** (6.0 μM) |

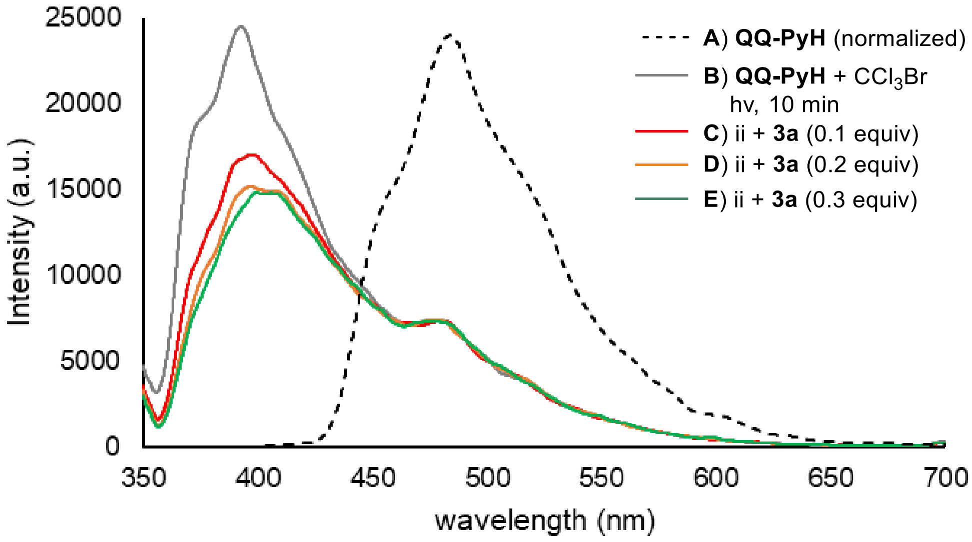


**Figure S43.** Catalytic fluorescence quenching of intermediate by amide **3a**.

### VI-4. EPR spectroscopy

Three samples were prepared in a vial containing **QQ-PyH** (0.9 mg, 0.003 mmol), *N*-*tert*-butylnitrone (PBN) (5.32 mg, 0.03 mmol), and 0.4 mL of anhydrous DMF: (A) without additive, (B) with $CCl_3Br$ (5.9 µL, 0.06 mmol) and (C) with **3a** (4.5 mg, 0.03 mmol). Each sample was transferred to an EPR tube. Sample B and C were irradiated with a blue LED (440 nm) for 10 min using a Kessil lamp (30 W). All samples were then frozen in liquid nitrogen. X-band EPR spectra of the frozen samples were obtained under following conditions: MW frequency = 9.38 GHz, temperature = 100 K, MW power = 2.52 mW, modulation amplitude = 10 G, modulation frequench = 100 kHz, and time constant = 0.01 ms. The high-spin region did not show any noticeable signal.

| Sample | Solution |
|---|---|
| **A** | **QQ-PyH** (0.003 mmol) + PBN (0.030 mmol) in DMF |
| **B** | **QQ-PyH** (0.003 mmol) + PBN (0.030 mmol) + $CCl_3Br$ (0.060 mmol) in DMF |
| **C** | **QQ-PyH** (0.003 mmol) + PBN (0.030 mmol) + **3a** (0.060 mmol) in DMF |

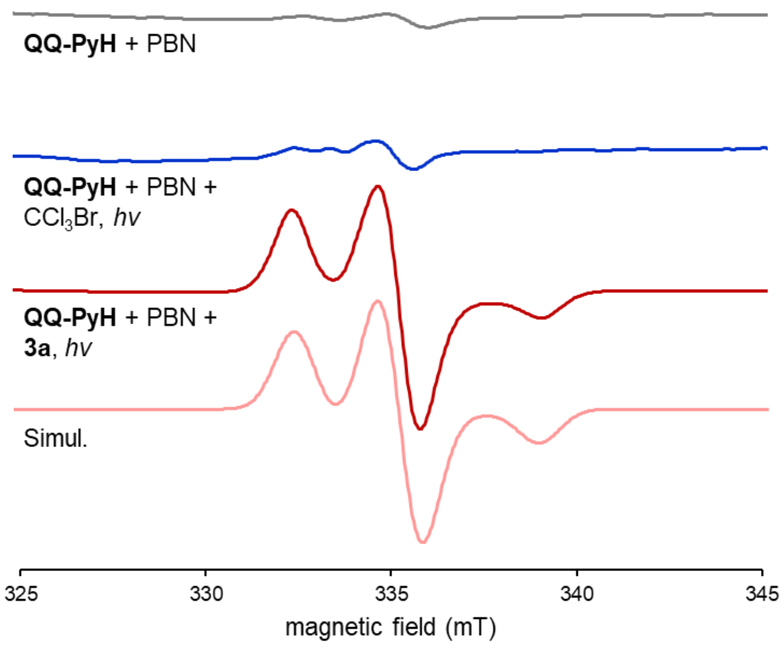


**Figure S44.** Experimental and simulated (pink) X-band EPR spectrum of each solution. Frequency = 9.38 GHz. Simulation parameters: $\boldsymbol{g}$ = [2.0066 2.0055 2.0011], $\boldsymbol{A}(^{14}N)$ = [11.07 11.44 90.14].

## VII. Computational analysis for catalytic cycle

### VII-1. General computational methods

DFT calculations for catalytic cycle investigation were carried out with Gaussian 16 quantum chemical package.[1] Geometry optimizations were performed with uB3LYP[2-6] level of theory with Grimme's D3 correction[7] and def2-SVP basis set. Vibrational frequency calculations were carried out at the same calculation theory level as the geometry optimization calculations, wherein thermochemistry correction energy ($G$–$E$) was acquired. No imaginary frequencies were found for all optimized intermediates, and the transition states were confirmed by the presence of a single imaginary frequency. The single-point energy calculations of the optimized geometries were performed with uB3LYP-D3/def2-TZVP level of theory. Implicit solvation energies were considered during the geometric optimization using Self-Consistent Reaction Field (SCRF)[16-18] calculations with radii and non-electrostatic terms disclosed by Truhalr and coworkers,[19] employing the dielectric constants of $\varepsilon_{ps}$ = 37.219 and $\varepsilon_{ps}$(inf) = 2.04633 for *N,N*-dimethylformamide. For transition structure **IV**, broken symmetry calculation was performed during both geometry optimization and single point energy calculation. Final solution phase Gibbs free energies ($G_{sol}$) were calculated as follows:

$$G_{sol,TZ} = E_{sol,TZ} + (G_{sol,DZ} - E_{sol,DZ})$$

$$\Delta G_{sol,TZ} = \Sigma G_{sol,TZ} \text{ for products} - \Sigma G_{sol,TZ} \text{ for reactants}$$

Graphical structures are visualized with ChemCraft. Spin density diagram was obtained by subtracting total beta spin density from the total alpha spin density.

## VII-2. Potential energy data of optimized structures

| Species | **I (QQ-Py)** | $CCl_3Br$ | $PhCONH_2$ | $Br^-$ | $CCl_3H$ | DMF | $[DMF-H]^+$ |
|---|---|---|---|---|---|---|---|
| Charge | 0 | 0 | 0 | -1 | 0 | 0 | 1 |
| Multiplicity | 1 | 1 | 1 | 1 | 1 | 1 | 1 |
| $\nu_{Im}$ | - | - | - | - | - | - | - |
| *E* (Hartree) | -1006.061440 | -3992.963281 | -400.450634 | -2574.364590 | -1419.401634 | -248.632410 | -249.067086 |
| *G-E* (Hartree) | 0.240306 | -0.023466 | 0.080769 | -0.016176 | -0.009661 | 0.072946 | 0.086310 |
| *G* (Hartree) | -1005.821134 | -3992.986747 | -400.369865 | -2574.380766 | -1419.411295 | -248.559464 | -248.980776 |
| Δ*G* (kcal/mol) | 0.0 | | | | | | |

| Species | $H_2O$ | PhCN | $[DMF-Br]^+$ | **II** | **III** | **IV** | **V** |
|---|---|---|---|---|---|---|---|
| Charge | 0 | 1 | 1 | 0 | 1 | 1 | 0 |
| Multiplicity | 1 | 1 | 1 | 1 | 1 | 1 | 1 |
| $\nu_{Im}$ | - | - | - | - | - | -1782.80 | - |
| *E* (Hartree) | -76.470921 | -324.632400 | -2956.431733 | -1407.218338 | -2825.810250 | -2825.762555 | -3980.756284 |
| *G-E* (Hartree) | 0.002903 | 0.069063 | 0.127875 | 0.355538 | 0.363085 | 0.350261 | 0.342324 |
| *G* (Hartree) | -76.468018 | -324.563337 | -2956.303858 | -1406.862800 | -2825.447165 | -2825.412294 | -3980.413960 |
| Δ*G* (kcal/mol) | | | | 2.38 | 13.56 | 35.44 | 15.24 |

| Species | **VI** | **VII** | **VIII** |
|---|---|---|---|
| Charge | 1 | 1 | 1 |
| Multiplicity | 1 | 1 | 1 |
| $\nu_{Im}$ | -184.40 | - | -72.20 |
| *E* (Hartree) | -4229.798145 | -3580.049203 | -3962.485039 |
| *G-E* (Hartree) | 0.442493 | 0.239194 | 0.388769 |
| *G* (Hartree) | -4229.355652 | -3579.810009 | -3962.096270 |
| Δ*G* (kcal/mol) | 39.77 | 11.42 | 19.48 |

**Table S8.** Potential energy data of optimized structures.

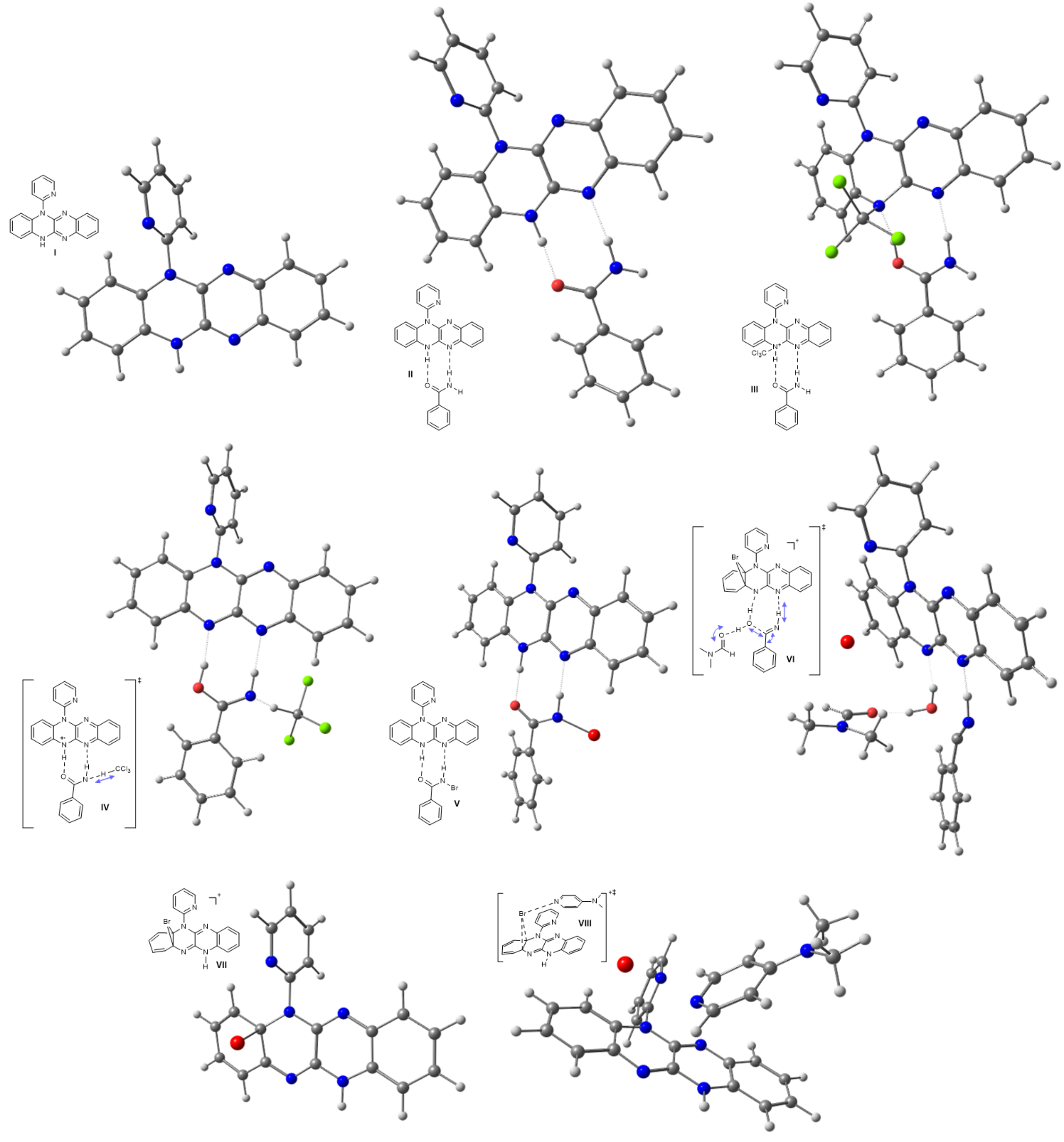


**Figure S45.** Optimized structures listed in Figure 4.

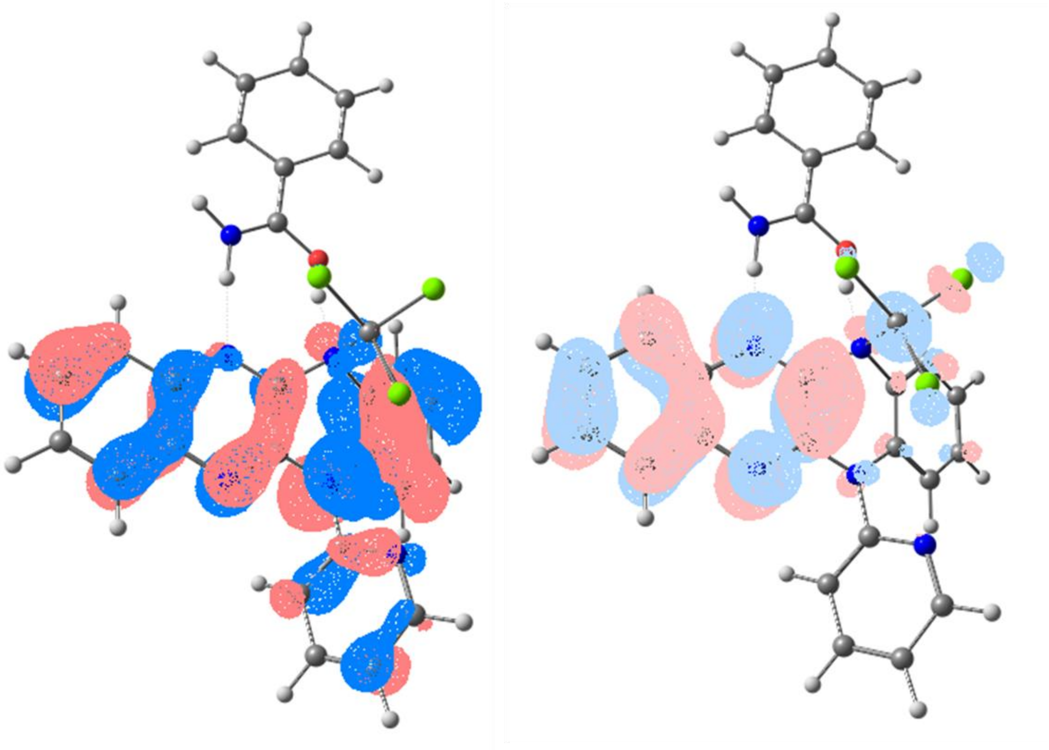

**Figure S46.** HOMO (left) and LUMO (right) diagram of intermediate **III**.

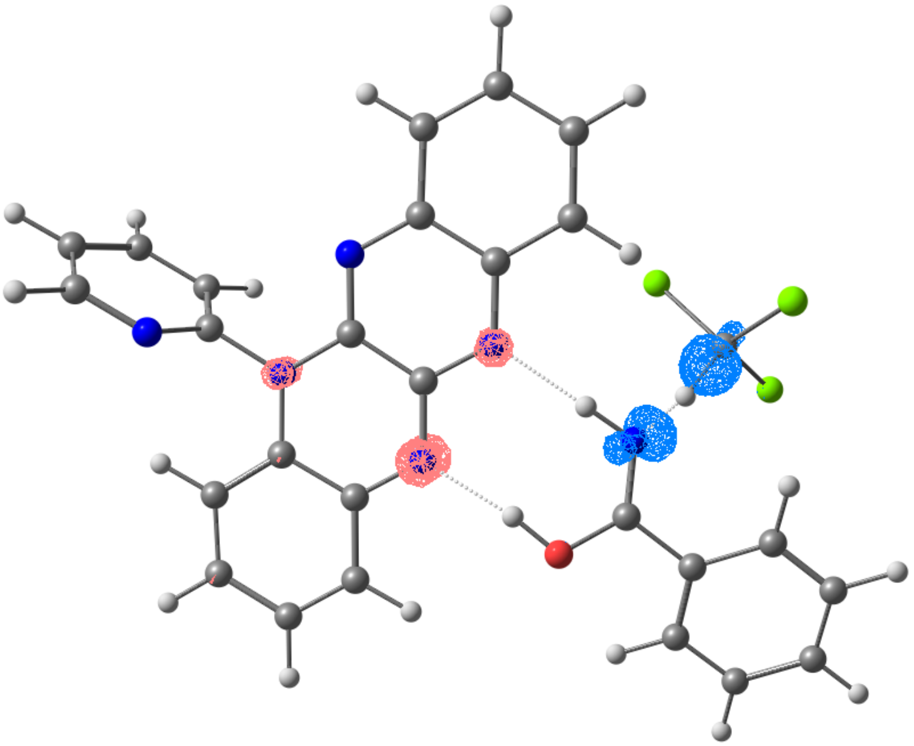

**Figure S47.** Spin density diagram of transition structure **IV**.

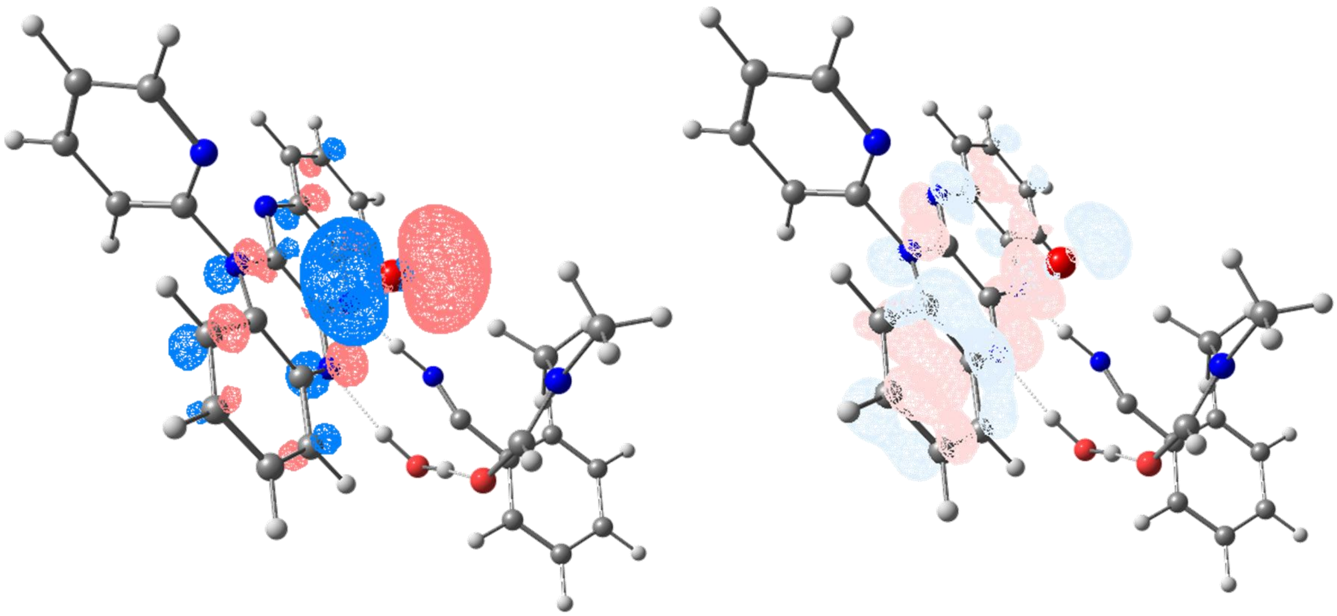

**Figure S48.** HOMO and LUMO diagram of transition structure **VI**.

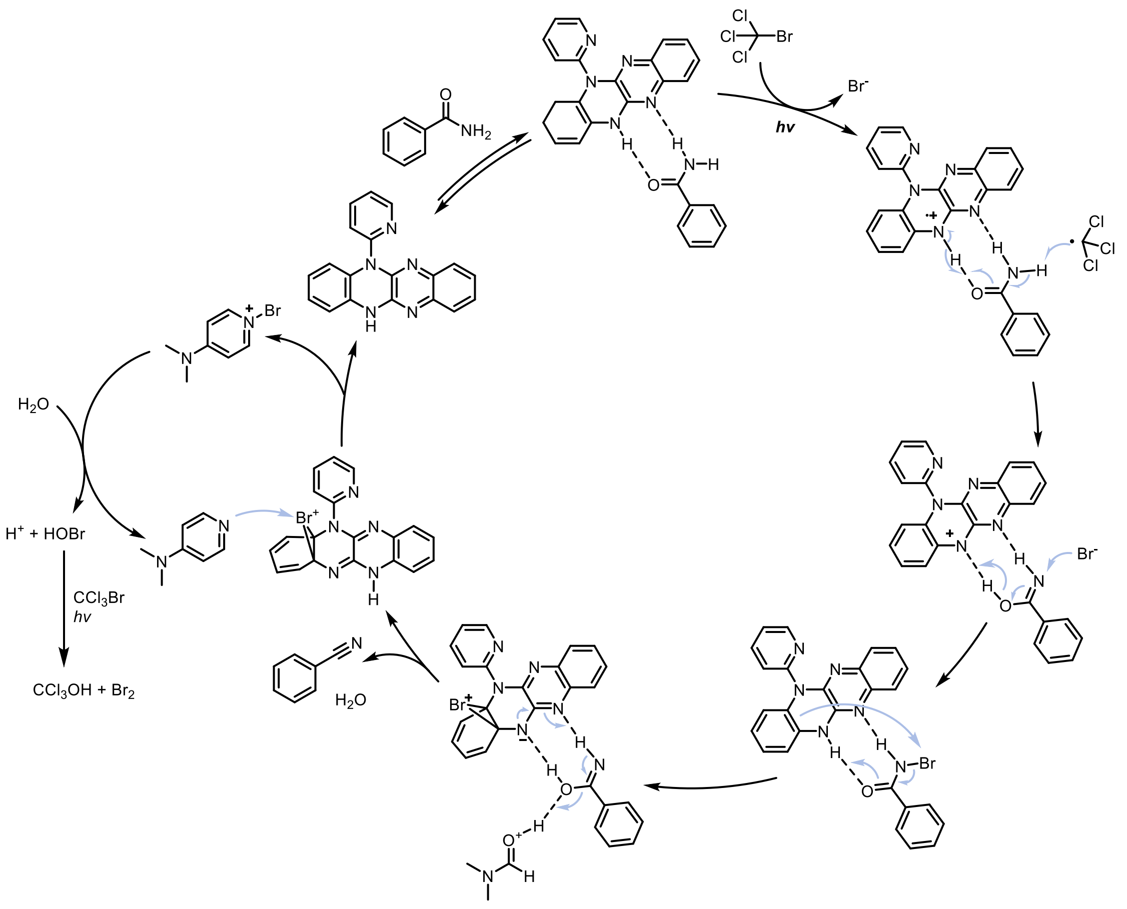


**Figure S49.** Proposed catalytic cycle of amide nitrilization in this study.

## References


(1) Frisch, M. J. T., G. W.; Schlegel, H. B.; Scuseria, G. E.; Robb, M. A.; Cheeseman, J. R.; Scalmani, G.; Barone, V.;; Mennucci, B. P., G. A.; Nakatsuji, H.; Caricato, M.; Li, X.; Hratchian, H. P.; Izmaylov, A. F.; Bloino, J.; Zheng,; G.; Sonnenberg, J. L. H., M.; Ehara, M.; Toyota, K.; Fukuda, R.; Hasegawa, J.; Ishida, M.; Nakajima, T.; Honda, Y.;; Kitao, O. N., H.; Vreven, T.; Montgomery, J. A., Jr.; Peralta, J. E.; Ogliaro, F.; Bearpark, M.; Heyd, J. J.; Brothers, E.;; Kudin, K. N. S., V. N.; Kobayashi, R.; Normand, J.; Raghavachari, K.; Rendell, A.; Burant, J. C.; Iyengar, S. S.;; Tomasi, J. C., M.; Rega, N.; Millam, J. M.; Klene, M.; Knox, J. E.; Cross, J. B.; Bakken, V.; Adamo, C.; Jaramillo, J.;; Gomperts, R. S., R. E.; Yazyev, O.; Austin, A. J.; Cammi, R.; Pomelli, C.; Ochterski, J. W.; Martin, R. L.;; Morokuma, K. Z., V. G.; Voth, G. A.; Salvador, P.; Dannenberg, J. J.; Dapprich, S.; Daniels, A. D.; Farkas, Ö.;; Foresman, J. B. O., J. V.; Cioslowski, J.; Fox, D. J.; et al. . Gaussian 09, revision A.1; Gaussian, Inc.: Wallingford, CT, 2009.

(2) Becke, A. D. Density-functional exchange-energy approximation with correct asymptotic behavior. *Phys. Rev. A* **1988**, *38* (6), 3098-3100; DOI: 10.1103/PhysRevA.38.3098.

(3) Becke, A. D. Density-functional thermochemistry. III. The role of exact exchange. *J. Chem. Phys.* **1993**, *98* (7), 5648-5652; DOI: 10.1063/1.464913.

(4) Lee, C.; Yang, W.; Parr, R. G. Development of the Colle-Salvetti correlation-energy formula into a functional of the electron density. *Phys. Rev. B* **1988**, *37* (2), 785-789; DOI: 10.1103/PhysRevB.37.785.

(5) Slater, J. C. *Quantum Theory of Molecules and Solids Vol. 4: The Self-Consistent Field for Molecules and Solids*; McGraw-Hill, New York, 1974.

(6) Vosko, S. H.; Wilk, L.; Nusair, M. Accurate spin-dependent electron liquid correlation energies for local spin density calculations: a critical analysis. *Can. J. Phys.* **1980**, *58* (8), 1200-1211; DOI: 10.1139/p80-159.

(7) Grimme, S.; Antony, J.; Ehrlich, S.; Krieg, H. A consistent and accurate ab initio parametrization of density functional dispersion correction (DFT-D) for the 94 elements H-Pu. *J. Chem. Phys.* **2010**, *132* (15), 154104; DOI: 10.1063/1.3382344.

(8) Romero, N. A.; Nicewicz, D. A. Organic Photoredox Catalysis. *Chem. Rev.* **2016**, *116* (17), 10075-10166; DOI: 10.1021/acs.chemrev.6b00057.

(9) Wang, Z.; Wang, X.; Ura, Y.; Nishihara, Y. Nickel-Catalyzed Decarbonylative Cyanation of Acyl Chlorides. *Org. Lett.* **2019**, *21* (17), 6779-6784; DOI: 10.1021/acs.orglett.9b02398.

(10) Li, J.; Lear, M. J.; Hayashi, Y. Sterically Demanding Oxidative Amidation of α-Substituted Malononitriles with Amines Using O2. *Angew. Chem., Int. Ed.* **2016**, *55* (31), 9060-9064; DOI: https://doi.org/10.1002/anie.201603399.

(11) Feng, B.; Guo, H.; Wang, X.; Hu, X.; Li, C.; Guo, Y.; Su, J.; Xuan, Q.; Song, Q. Difluorocarbene-Enabled Dehydration of Primary Amides To Access Nitriles. *Org. Lett.* **2025**, *27* (12), 2992-2996; DOI: 10.1021/acs.orglett.5c00629.

(12) Xu, L.; Hu, Y.; Zhu, X.; He, L.; Wu, Q.; Li, C.; Xia, C.; Liu, C. Momentary click nitrile synthesis enabled by an aminoazanium reagent. *Org. Chem. Front.* **2022**, *9* (13), 3420-3427; DOI: 10.1039/D2QO00560C.

(13) Ali, S. Z.; Manno, N. A.; Shen, J.; Schenker, A.; Lipshultz, J. M.; White, N. A.; Radosevich, A. T. Nitrilation of carboxylic acids by PIII/PV-catalysis. *Chem. Sci.* **2025**, *16* (35), 16145-16150; DOI: 10.1039/D5SC05216E.

(14) Liang, Y.; Pan, W.; Wang, M.; Wan, K.; Cui, A.; Huang, X.; Kong, L.; Zhang, L.; Peng, B. [5,5]-Sigmatropic Rearrangement of Aryl Sulfoxides with α,β-Unsaturated Nitriles. *Organometallics* **2026**, *45* (2), 219-223; DOI: 10.1021/acs.organomet.5c00470.

(15) Li, Z.; Zhang, G. a.; Song, Y.; Li, M.; Li, Z.; Ding, W.; Wu, J. Copper-Catalyzed Enantioselective Decarboxylative Cyanation of Benzylic Acids Promoted by Hypervalent Iodine(III) Reagents. *Org. Lett.* **2023**, *25* (17), 3023-3028; DOI: 10.1021/acs.orglett.3c00816.

(16) Marten, B.; Kim, K.; Cortis, C.; Friesner, R. A.; Murphy, R. B.; Ringnalda, M. N.; Sitkoff, D.; Honig, B. New Model for Calculation of Solvation Free Energies: Correction of Self-Consistent Reaction Field Continuum Dielectric Theory for Short-Range Hydrogen-Bonding Effects. *J. Phys. Chem.* **1996**, *100* (28), 11775-11788; DOI: 10.1021/jp953087x.

(17) Edinger, S. R.; Cortis, C.; Shenkin, P. S.; Friesner, R. A. Solvation Free Energies of Peptides: Comparison of Approximate Continuum Solvation Models with Accurate Solution of the Poisson−Boltzmann Equation. *J. Phys. Chem. B* **1997**, *101* (7), 1190-1197; DOI: 10.1021/jp962156k.

(18) Friedrichs, M.; Zhou, R.; Edinger, S. R.; Friesner, R. A. Poisson−Boltzmann Analytical Gradients for Molecular Modeling Calculations. *J. Phys. Chem. B* **1999**, *103* (16), 3057-3061; DOI: 10.1021/jp982513m.

(19) Marenich, A. V.; Cramer, C. J.; Truhlar, D. G. Universal Solvation Model Based on Solute Electron Density and on a Continuum Model of the Solvent Defined by the Bulk Dielectric Constant and Atomic Surface Tensions. *J. Phys. Chem. B* **2009**, *113* (18), 6378-6396; DOI: 10.1021/jp810292n.

# *Appendix I.*
# *Crystallographic data*

**AI-I. Crystallographic data of QQ-Mes**

Table 1. Crystal data and structure refinement for **QQ-Mes**.

| | | |
|---|---|---|
| Empirical formula | $C_{23} H_{20} N_4$ | |
| Formula weight | 352.43 | |
| Temperature | 173(2) K | |
| Wavelength | 0.71073 Å | |
| Crystal system | Triclinic | |
| Space group | *P*-1 | |
| Unit cell dimensions | a = 10.9721(11) Å | α = 86.726(3)° |
| | b = 11.3455(11) Å | β = 87.154(3)° |
| | c = 17.3743(17) Å | γ = 68.917(3)° |
| Volume | 2013.8(3) $Å^3$ | |
| Z | 4 | |
| Density (calculated) | 1.162 $Mg/m^3$ | |
| Absorption coefficient | 0.071 $mm^{-1}$ | |
| F(000) | 744 | |
| Crystal size | 0.122 x 0.025 x 0.021 $mm^3$ | |
| Theta range for data collection | 2.577 to 28.005°. | |
| Index ranges | –14<=h<=14, –14<=k<=14, –22<=l<=22 | |
| Reflections collected | 94994 | |
| Independent reflections | 9690 [R(int) = 0.0793] | |
| Completeness to theta = 25.242° | 99.8 % | |
| Absorption correction | Semi-empirical from equivalents | |
| Max. and min. transmission | 0.7404 and 0.7155 | |
| Refinement method | Full-matrix least-squares on $F^2$ | |
| Data / restraints / parameters | 9690 / 0 / 499 | |
| Goodness-of-fit on $F^2$ | 1.029 | |
| Final R indices [I>2sigma(I)] | R1 = 0.0584, wR2 = 0.1260 | |
| R indices (all data) | R1 = 0.0881, wR2 = 0.1430 | |
| Largest diff. peak and hole | 0.292 and –0.258 e·$Å^{-3}$ | |

Table 2. Atomic coordinates ( x $10^4$ ) and equivalent isotropic displacement parameters ( $Å^2$ x $10^3$ ) for **QQ-Mes**. U(eq) is defined as one third of the trace of the orthogonalized $U^{ij}$ tensor.

| | x | y | z | U(eq) |
|---|---|---|---|---|
| C(1) | 6848(2) | 2043(2) | 7400(1) | 27(1) |
| N(2) | 7169(1) | 825(1) | 7317(1) | 31(1) |
| C(3) | 6277(2) | 445(2) | 6952(1) | 33(1) |
| C(4) | 6546(2) | -842(2) | 6851(1) | 48(1) |
| C(5) | 5684(2) | -1221(2) | 6471(2) | 56(1) |
| C(6) | 4546(2) | -332(2) | 6175(1) | 53(1) |
| C(7) | 4263(2) | 932(2) | 6272(1) | 43(1) |
| C(8) | 5109(2) | 1345(2) | 6668(1) | 32(1) |
| N(9) | 4788(1) | 2618(1) | 6769(1) | 31(1) |
| C(10) | 5606(2) | 2951(2) | 7141(1) | 28(1) |
| N(11) | 5296(1) | 4186(1) | 7298(1) | 33(1) |
| C(12) | 6117(2) | 4637(2) | 7675(1) | 32(1) |
| C(13) | 5741(2) | 5914(2) | 7821(1) | 41(1) |
| C(14) | 6564(2) | 6352(2) | 8200(1) | 48(1) |
| C(15) | 7771(2) | 5521(2) | 8428(1) | 48(1) |
| C(16) | 8163(2) | 4246(2) | 8271(1) | 39(1) |
| C(17) | 7345(2) | 3793(2) | 7897(1) | 30(1) |
| N(18) | 7706(1) | 2499(1) | 7735(1) | 29(1) |
| C(19) | 9030(2) | 1653(2) | 7878(1) | 27(1) |
| C(20) | 9377(2) | 1205(2) | 8627(1) | 33(1) |
| C(21) | 10674(2) | 430(2) | 8747(1) | 40(1) |
| C(22) | 11597(2) | 105(2) | 8148(1) | 40(1) |
| C(23) | 11217(2) | 589(2) | 7410(1) | 37(1) |
| C(24) | 9936(2) | 1369(2) | 7260(1) | 31(1) |
| C(25) | 8411(2) | 1548(2) | 9300(1) | 44(1) |
| C(26) | 12991(2) | -771(2) | 8278(2) | 61(1) |
| C(27) | 9558(2) | 1852(2) | 6451(1) | 47(1) |
| C(31) | 1664(2) | 6785(2) | 7468(1) | 27(1) |
| N(32) | 2718(1) | 6267(1) | 7035(1) | 29(1) |
| C(33) | 2792(2) | 6852(2) | 6318(1) | 31(1) |
| C(34) | 3906(2) | 6363(2) | 5834(1) | 43(1) |

| | | | | |
|---|---|---|---|---|
| C(35) | 3996(2) | 6950(2) | 5128(1) | 45(1) |
| C(36) | 2970(2) | 8039(2) | 4887(1) | 42(1) |
| C(37) | 1869(2) | 8529(2) | 5348(1) | 37(1) |
| C(38) | 1758(2) | 7947(2) | 6070(1) | 30(1) |
| N(39) | 652(1) | 8472(1) | 6532(1) | 32(1) |
| C(40) | 616(2) | 7926(2) | 7210(1) | 27(1) |
| N(41) | -434(1) | 8436(1) | 7696(1) | 32(1) |
| C(42) | -541(2) | 7933(2) | 8434(1) | 27(1) |
| C(43) | -1630(2) | 8518(2) | 8908(1) | 34(1) |
| C(44) | -1733(2) | 8023(2) | 9645(1) | 38(1) |
| C(45) | -756(2) | 6936(2) | 9912(1) | 37(1) |
| C(46) | 323(2) | 6338(2) | 9434(1) | 33(1) |
| C(47) | 443(2) | 6824(2) | 8694(1) | 27(1) |
| N(48) | 1523(1) | 6236(1) | 8193(1) | 27(1) |
| C(49) | 2415(2) | 4979(2) | 8400(1) | 26(1) |
| C(50) | 3342(2) | 4817(2) | 8959(1) | 31(1) |
| C(51) | 4137(2) | 3583(2) | 9171(1) | 35(1) |
| C(52) | 4032(2) | 2541(2) | 8845(1) | 33(1) |
| C(53) | 3094(2) | 2738(2) | 8293(1) | 32(1) |
| C(54) | 2283(2) | 3945(2) | 8058(1) | 28(1) |
| C(55) | 3489(2) | 5909(2) | 9348(1) | 42(1) |
| C(56) | 4924(2) | 1215(2) | 9077(1) | 47(1) |
| C(57) | 1332(2) | 4116(2) | 7430(1) | 38(1) |

Table 3. Bond lengths [Å] and angles [°] for **QQ-Mes**.

| | |
|---|---|
| C(1)-N(2) | 1.313(2) |
| C(1)-N(18) | 1.390(2) |
| C(1)-C(10) | 1.455(2) |
| N(2)-C(3) | 1.393(2) |
| C(3)-C(4) | 1.402(3) |
| C(3)-C(8) | 1.412(2) |
| C(4)-C(5) | 1.379(3) |
| C(4)-H(4) | 0.9500 |
| C(5)-C(6) | 1.393(3) |
| C(5)-H(5) | 0.9500 |
| C(6)-C(7) | 1.373(3) |
| C(6)-H(6) | 0.9500 |
| C(7)-C(8) | 1.402(3) |
| C(7)-H(7) | 0.9500 |
| C(8)-N(9) | 1.378(2) |
| N(9)-C(10) | 1.301(2) |
| C(10)-N(11) | 1.358(2) |
| N(11)-C(12) | 1.390(2) |
| N(11)-H(11) | 0.92(2) |
| C(12)-C(13) | 1.391(3) |
| C(12)-C(17) | 1.401(2) |
| C(13)-C(14) | 1.384(3) |
| C(13)-H(13) | 0.9500 |
| C(14)-C(15) | 1.383(3) |
| C(14)-H(14) | 0.9500 |
| C(15)-C(16) | 1.392(3) |
| C(15)-H(15) | 0.9500 |
| C(16)-C(17) | 1.385(3) |
| C(16)-H(16) | 0.9500 |
| C(17)-N(18) | 1.418(2) |
| N(18)-C(19) | 1.449(2) |
| C(19)-C(20) | 1.390(2) |
| C(19)-C(24) | 1.396(2) |
| C(20)-C(21) | 1.396(3) |

| | |
|---|---|
| C(20)-C(25) | 1.508(3) |
| C(21)-C(22) | 1.383(3) |
| C(21)-H(21) | 0.9500 |
| C(22)-C(23) | 1.389(3) |
| C(22)-C(26) | 1.514(3) |
| C(23)-C(24) | 1.393(2) |
| C(23)-H(23) | 0.9500 |
| C(24)-C(27) | 1.501(3) |
| C(25)-H(25A) | 0.9800 |
| C(25)-H(25B) | 0.9800 |
| C(25)-H(25C) | 0.9800 |
| C(26)-H(26A) | 0.9800 |
| C(26)-H(26B) | 0.9800 |
| C(26)-H(26C) | 0.9800 |
| C(27)-H(27A) | 0.9800 |
| C(27)-H(27B) | 0.9800 |
| C(27)-H(27C) | 0.9800 |
| C(31)-N(32) | 1.313(2) |
| C(31)-N(48) | 1.399(2) |
| C(31)-C(40) | 1.453(2) |
| N(32)-C(33) | 1.391(2) |
| C(33)-C(34) | 1.404(3) |
| C(33)-C(38) | 1.410(2) |
| C(34)-C(35) | 1.380(3) |
| C(34)-H(34) | 0.9500 |
| C(35)-C(36) | 1.398(3) |
| C(35)-H(35) | 0.9500 |
| C(36)-C(37) | 1.370(3) |
| C(36)-H(36) | 0.9500 |
| C(37)-C(38) | 1.405(2) |
| C(37)-H(37) | 0.9500 |
| C(38)-N(39) | 1.382(2) |
| N(39)-C(40) | 1.304(2) |
| C(40)-N(41) | 1.363(2) |
| N(41)-C(42) | 1.389(2) |
| N(41)-H(41) | 0.96(2) |

| | |
|---|---|
| C(42)-C(43) | 1.393(2) |
| C(42)-C(47) | 1.400(2) |
| C(43)-C(44) | 1.385(3) |
| C(43)-H(43) | 0.9500 |
| C(44)-C(45) | 1.385(3) |
| C(44)-H(44) | 0.9500 |
| C(45)-C(46) | 1.392(3) |
| C(45)-H(45) | 0.9500 |
| C(46)-C(47) | 1.389(2) |
| C(46)-H(46) | 0.9500 |
| C(47)-N(48) | 1.415(2) |
| N(48)-C(49) | 1.447(2) |
| C(49)-C(50) | 1.398(2) |
| C(49)-C(54) | 1.400(2) |
| C(50)-C(51) | 1.397(2) |
| C(50)-C(55) | 1.505(3) |
| C(51)-C(52) | 1.383(3) |
| C(51)-H(51) | 0.9500 |
| C(52)-C(53) | 1.392(3) |
| C(52)-C(56) | 1.514(2) |
| C(53)-C(54) | 1.389(2) |
| C(53)-H(53) | 0.9500 |
| C(54)-C(57) | 1.503(2) |
| C(55)-H(55A) | 0.9800 |
| C(55)-H(55B) | 0.9800 |
| C(55)-H(55C) | 0.9800 |
| C(56)-H(56A) | 0.9800 |
| C(56)-H(56B) | 0.9800 |
| C(56)-H(56C) | 0.9800 |
| C(57)-H(57A) | 0.9800 |
| C(57)-H(57B) | 0.9800 |
| C(57)-H(57C) | 0.9800 |
| | |
| N(2)-C(1)-N(18) | 120.14(15) |
| N(2)-C(1)-C(10) | 121.66(15) |
| N(18)-C(1)-C(10) | 118.20(15) |

| | |
|---|---|
| C(1)-N(2)-C(3) | 116.74(15) |
| N(2)-C(3)-C(4) | 119.99(17) |
| N(2)-C(3)-C(8) | 120.80(16) |
| C(4)-C(3)-C(8) | 119.21(17) |
| C(5)-C(4)-C(3) | 120.2(2) |
| C(5)-C(4)-H(4) | 119.9 |
| C(3)-C(4)-H(4) | 119.9 |
| C(4)-C(5)-C(6) | 120.6(2) |
| C(4)-C(5)-H(5) | 119.7 |
| C(6)-C(5)-H(5) | 119.7 |
| C(7)-C(6)-C(5) | 120.00(19) |
| C(7)-C(6)-H(6) | 120.0 |
| C(5)-C(6)-H(6) | 120.0 |
| C(6)-C(7)-C(8) | 120.74(19) |
| C(6)-C(7)-H(7) | 119.6 |
| C(8)-C(7)-H(7) | 119.6 |
| N(9)-C(8)-C(7) | 119.52(17) |
| N(9)-C(8)-C(3) | 121.26(16) |
| C(7)-C(8)-C(3) | 119.22(18) |
| C(10)-N(9)-C(8) | 117.09(15) |
| N(9)-C(10)-N(11) | 119.32(15) |
| N(9)-C(10)-C(1) | 122.28(16) |
| N(11)-C(10)-C(1) | 118.39(15) |
| C(10)-N(11)-C(12) | 123.53(15) |
| C(10)-N(11)-H(11) | 117.9(14) |
| C(12)-N(11)-H(11) | 118.1(14) |
| N(11)-C(12)-C(13) | 120.76(16) |
| N(11)-C(12)-C(17) | 119.25(16) |
| C(13)-C(12)-C(17) | 119.98(17) |
| C(14)-C(13)-C(12) | 120.30(18) |
| C(14)-C(13)-H(13) | 119.9 |
| C(12)-C(13)-H(13) | 119.9 |
| C(15)-C(14)-C(13) | 119.94(18) |
| C(15)-C(14)-H(14) | 120.0 |
| C(13)-C(14)-H(14) | 120.0 |
| C(14)-C(15)-C(16) | 120.05(19) |

| | |
|---|---|
| C(14)-C(15)-H(15) | 120.0 |
| C(16)-C(15)-H(15) | 120.0 |
| C(17)-C(16)-C(15) | 120.56(18) |
| C(17)-C(16)-H(16) | 119.7 |
| C(15)-C(16)-H(16) | 119.7 |
| C(16)-C(17)-C(12) | 119.16(16) |
| C(16)-C(17)-N(18) | 122.14(16) |
| C(12)-C(17)-N(18) | 118.70(15) |
| C(1)-N(18)-C(17) | 121.64(14) |
| C(1)-N(18)-C(19) | 119.44(14) |
| C(17)-N(18)-C(19) | 118.80(13) |
| C(20)-C(19)-C(24) | 122.03(16) |
| C(20)-C(19)-N(18) | 119.36(15) |
| C(24)-C(19)-N(18) | 118.53(15) |
| C(19)-C(20)-C(21) | 117.76(17) |
| C(19)-C(20)-C(25) | 122.15(17) |
| C(21)-C(20)-C(25) | 120.08(17) |
| C(22)-C(21)-C(20) | 122.02(18) |
| C(22)-C(21)-H(21) | 119.0 |
| C(20)-C(21)-H(21) | 119.0 |
| C(21)-C(22)-C(23) | 118.49(17) |
| C(21)-C(22)-C(26) | 121.7(2) |
| C(23)-C(22)-C(26) | 119.8(2) |
| C(22)-C(23)-C(24) | 121.73(18) |
| C(22)-C(23)-H(23) | 119.1 |
| C(24)-C(23)-H(23) | 119.1 |
| C(23)-C(24)-C(19) | 117.95(17) |
| C(23)-C(24)-C(27) | 120.11(17) |
| C(19)-C(24)-C(27) | 121.92(16) |
| C(20)-C(25)-H(25A) | 109.5 |
| C(20)-C(25)-H(25B) | 109.5 |
| H(25A)-C(25)-H(25B) | 109.5 |
| C(20)-C(25)-H(25C) | 109.5 |
| H(25A)-C(25)-H(25C) | 109.5 |
| H(25B)-C(25)-H(25C) | 109.5 |
| C(22)-C(26)-H(26A) | 109.5 |

| | |
|---|---|
| C(22)-C(26)-H(26B) | 109.5 |
| H(26A)-C(26)-H(26B) | 109.5 |
| C(22)-C(26)-H(26C) | 109.5 |
| H(26A)-C(26)-H(26C) | 109.5 |
| H(26B)-C(26)-H(26C) | 109.5 |
| C(24)-C(27)-H(27A) | 109.5 |
| C(24)-C(27)-H(27B) | 109.5 |
| H(27A)-C(27)-H(27B) | 109.5 |
| C(24)-C(27)-H(27C) | 109.5 |
| H(27A)-C(27)-H(27C) | 109.5 |
| H(27B)-C(27)-H(27C) | 109.5 |
| N(32)-C(31)-N(48) | 120.19(14) |
| N(32)-C(31)-C(40) | 121.41(15) |
| N(48)-C(31)-C(40) | 118.40(14) |
| C(31)-N(32)-C(33) | 117.36(14) |
| N(32)-C(33)-C(34) | 120.21(16) |
| N(32)-C(33)-C(38) | 120.54(15) |
| C(34)-C(33)-C(38) | 119.25(16) |
| C(35)-C(34)-C(33) | 120.51(18) |
| C(35)-C(34)-H(34) | 119.7 |
| C(33)-C(34)-H(34) | 119.7 |
| C(34)-C(35)-C(36) | 119.96(18) |
| C(34)-C(35)-H(35) | 120.0 |
| C(36)-C(35)-H(35) | 120.0 |
| C(37)-C(36)-C(35) | 120.53(17) |
| C(37)-C(36)-H(36) | 119.7 |
| C(35)-C(36)-H(36) | 119.7 |
| C(36)-C(37)-C(38) | 120.48(18) |
| C(36)-C(37)-H(37) | 119.8 |
| C(38)-C(37)-H(37) | 119.8 |
| N(39)-C(38)-C(37) | 119.52(16) |
| N(39)-C(38)-C(33) | 121.20(15) |
| C(37)-C(38)-C(33) | 119.27(16) |
| C(40)-N(39)-C(38) | 117.21(15) |
| N(39)-C(40)-N(41) | 119.32(15) |
| N(39)-C(40)-C(31) | 122.24(15) |

| | |
|---|---|
| N(41)-C(40)-C(31) | 118.44(15) |
| C(40)-N(41)-C(42) | 123.22(14) |
| C(40)-N(41)-H(41) | 119.1(13) |
| C(42)-N(41)-H(41) | 117.3(13) |
| N(41)-C(42)-C(43) | 120.28(15) |
| N(41)-C(42)-C(47) | 119.63(15) |
| C(43)-C(42)-C(47) | 120.08(16) |
| C(44)-C(43)-C(42) | 120.36(17) |
| C(44)-C(43)-H(43) | 119.8 |
| C(42)-C(43)-H(43) | 119.8 |
| C(43)-C(44)-C(45) | 119.97(17) |
| C(43)-C(44)-H(44) | 120.0 |
| C(45)-C(44)-H(44) | 120.0 |
| C(44)-C(45)-C(46) | 119.78(17) |
| C(44)-C(45)-H(45) | 120.1 |
| C(46)-C(45)-H(45) | 120.1 |
| C(47)-C(46)-C(45) | 120.95(17) |
| C(47)-C(46)-H(46) | 119.5 |
| C(45)-C(46)-H(46) | 119.5 |
| C(46)-C(47)-C(42) | 118.85(15) |
| C(46)-C(47)-N(48) | 122.38(15) |
| C(42)-C(47)-N(48) | 118.77(15) |
| C(31)-N(48)-C(47) | 121.35(13) |
| C(31)-N(48)-C(49) | 119.40(13) |
| C(47)-N(48)-C(49) | 118.89(13) |
| C(50)-C(49)-C(54) | 121.56(15) |
| C(50)-C(49)-N(48) | 119.72(15) |
| C(54)-C(49)-N(48) | 118.66(14) |
| C(51)-C(50)-C(49) | 117.78(16) |
| C(51)-C(50)-C(55) | 119.45(16) |
| C(49)-C(50)-C(55) | 122.75(16) |
| C(52)-C(51)-C(50) | 122.13(17) |
| C(52)-C(51)-H(51) | 118.9 |
| C(50)-C(51)-H(51) | 118.9 |
| C(51)-C(52)-C(53) | 118.49(16) |
| C(51)-C(52)-C(56) | 120.85(18) |

| | |
|---|---|
| C(53)-C(52)-C(56) | 120.66(18) |
| C(54)-C(53)-C(52) | 121.76(17) |
| C(54)-C(53)-H(53) | 119.1 |
| C(52)-C(53)-H(53) | 119.1 |
| C(53)-C(54)-C(49) | 118.28(16) |
| C(53)-C(54)-C(57) | 119.95(16) |
| C(49)-C(54)-C(57) | 121.73(15) |
| C(50)-C(55)-H(55A) | 109.5 |
| C(50)-C(55)-H(55B) | 109.5 |
| H(55A)-C(55)-H(55B) | 109.5 |
| C(50)-C(55)-H(55C) | 109.5 |
| H(55A)-C(55)-H(55C) | 109.5 |
| H(55B)-C(55)-H(55C) | 109.5 |
| C(52)-C(56)-H(56A) | 109.5 |
| C(52)-C(56)-H(56B) | 109.5 |
| H(56A)-C(56)-H(56B) | 109.5 |
| C(52)-C(56)-H(56C) | 109.5 |
| H(56A)-C(56)-H(56C) | 109.5 |
| H(56B)-C(56)-H(56C) | 109.5 |
| C(54)-C(57)-H(57A) | 109.5 |
| C(54)-C(57)-H(57B) | 109.5 |
| H(57A)-C(57)-H(57B) | 109.5 |
| C(54)-C(57)-H(57C) | 109.5 |
| H(57A)-C(57)-H(57C) | 109.5 |
| H(57B)-C(57)-H(57C) | 109.5 |

Symmetry transformations used to generate equivalent atoms:

Table 4. Anisotropic displacement parameters ( $Å^2 \times 10^3$ ) for **QQ-Mes**. The anisotropic displacement factor exponent takes the form: $-2\pi^2[ h^2 a^{*2} U^{11} + ... + 2 h k a^* b^* U^{12} ]$

| | $U^{11}$ | $U^{22}$ | $U^{33}$ | $U^{23}$ | $U^{13}$ | $U^{12}$ |
|---|---|---|---|---|---|---|
| C(1) | 24(1) | 28(1) | 26(1) | 1(1) | 1(1) | -7(1) |
| N(2) | 28(1) | 27(1) | 35(1) | 2(1) | -4(1) | -9(1) |
| C(3) | 31(1) | 34(1) | 33(1) | 0(1) | -2(1) | -14(1) |
| C(4) | 41(1) | 37(1) | 67(1) | -3(1) | -10(1) | -13(1) |
| C(5) | 55(1) | 41(1) | 80(2) | -14(1) | -10(1) | -22(1) |
| C(6) | 44(1) | 59(1) | 65(1) | -18(1) | -7(1) | -26(1) |
| C(7) | 31(1) | 50(1) | 49(1) | -9(1) | -5(1) | -13(1) |
| C(8) | 27(1) | 38(1) | 30(1) | -3(1) | 2(1) | -11(1) |
| N(9) | 25(1) | 36(1) | 32(1) | -2(1) | -2(1) | -9(1) |
| C(10) | 24(1) | 30(1) | 27(1) | 1(1) | 1(1) | -7(1) |
| N(11) | 24(1) | 27(1) | 42(1) | -2(1) | -5(1) | -4(1) |
| C(12) | 26(1) | 28(1) | 38(1) | -2(1) | 2(1) | -7(1) |
| C(13) | 31(1) | 28(1) | 59(1) | -4(1) | -2(1) | -4(1) |
| C(14) | 42(1) | 28(1) | 74(2) | -11(1) | 0(1) | -12(1) |
| C(15) | 39(1) | 40(1) | 68(1) | -10(1) | -5(1) | -18(1) |
| C(16) | 30(1) | 33(1) | 52(1) | -2(1) | -6(1) | -10(1) |
| C(17) | 27(1) | 25(1) | 35(1) | 0(1) | 1(1) | -7(1) |
| N(18) | 23(1) | 25(1) | 34(1) | 0(1) | -4(1) | -4(1) |
| C(19) | 23(1) | 22(1) | 35(1) | 0(1) | -6(1) | -5(1) |
| C(20) | 36(1) | 29(1) | 33(1) | 1(1) | -6(1) | -11(1) |
| C(21) | 43(1) | 32(1) | 45(1) | 7(1) | -19(1) | -11(1) |
| C(22) | 28(1) | 26(1) | 66(1) | 0(1) | -15(1) | -6(1) |
| C(23) | 28(1) | 30(1) | 51(1) | -7(1) | 2(1) | -10(1) |
| C(24) | 29(1) | 30(1) | 36(1) | -1(1) | -4(1) | -12(1) |
| C(25) | 55(1) | 45(1) | 33(1) | 0(1) | -1(1) | -18(1) |
| C(26) | 32(1) | 39(1) | 105(2) | 3(1) | -23(1) | -3(1) |
| C(27) | 43(1) | 58(1) | 36(1) | 5(1) | 0(1) | -15(1) |
| C(31) | 26(1) | 23(1) | 31(1) | -2(1) | -4(1) | -8(1) |
| N(32) | 26(1) | 26(1) | 32(1) | 0(1) | -1(1) | -5(1) |
| C(33) | 31(1) | 31(1) | 30(1) | -1(1) | -2(1) | -9(1) |
| C(34) | 38(1) | 41(1) | 40(1) | 1(1) | 1(1) | -4(1) |

| | | | | | | |
|---|---|---|---|---|---|---|
| C(35) | 40(1) | 53(1) | 34(1) | -4(1) | 6(1) | -9(1) |
| C(36) | 46(1) | 53(1) | 27(1) | 4(1) | -1(1) | -17(1) |
| C(37) | 37(1) | 42(1) | 30(1) | 4(1) | -6(1) | -11(1) |
| C(38) | 30(1) | 31(1) | 29(1) | -1(1) | -5(1) | -11(1) |
| N(39) | 31(1) | 33(1) | 29(1) | 2(1) | -2(1) | -9(1) |
| C(40) | 25(1) | 24(1) | 31(1) | -2(1) | -2(1) | -7(1) |
| N(41) | 25(1) | 29(1) | 34(1) | -1(1) | 4(1) | -2(1) |
| C(42) | 25(1) | 23(1) | 34(1) | -3(1) | 1(1) | -7(1) |
| C(43) | 28(1) | 26(1) | 43(1) | -2(1) | 5(1) | -4(1) |
| C(44) | 36(1) | 35(1) | 41(1) | -7(1) | 12(1) | -11(1) |
| C(45) | 43(1) | 36(1) | 33(1) | 1(1) | 4(1) | -17(1) |
| C(46) | 32(1) | 26(1) | 38(1) | -2(1) | -3(1) | -9(1) |
| C(47) | 26(1) | 24(1) | 32(1) | -4(1) | 0(1) | -9(1) |
| N(48) | 23(1) | 23(1) | 31(1) | 1(1) | -2(1) | -2(1) |
| C(49) | 24(1) | 22(1) | 29(1) | 1(1) | -1(1) | -4(1) |
| C(50) | 29(1) | 30(1) | 32(1) | -1(1) | -3(1) | -8(1) |
| C(51) | 29(1) | 36(1) | 33(1) | 4(1) | -6(1) | -5(1) |
| C(52) | 28(1) | 27(1) | 37(1) | 7(1) | 5(1) | -3(1) |
| C(53) | 32(1) | 25(1) | 37(1) | -1(1) | 6(1) | -9(1) |
| C(54) | 26(1) | 28(1) | 30(1) | -1(1) | 2(1) | -9(1) |
| C(55) | 40(1) | 39(1) | 46(1) | -5(1) | -11(1) | -12(1) |
| C(56) | 37(1) | 32(1) | 60(1) | 13(1) | 3(1) | 0(1) |
| C(57) | 38(1) | 34(1) | 43(1) | -3(1) | -9(1) | -15(1) |

Table 5. Hydrogen coordinates ( x $10^4$ ) and isotropic displacement parameters ( $Å^2$ x $10^3$ ) for **QQ-Mes**.

| | x | y | z | U(eq) |
|---|---|---|---|---|
| H(4) | 7325 | -1456 | 7045 | 58 |
| H(5) | 5869 | -2096 | 6410 | 68 |
| H(6) | 3965 | -601 | 5906 | 64 |
| H(7) | 3485 | 1534 | 6069 | 52 |
| H(11) | 4460(20) | 4730(20) | 7206(13) | 49 |
| H(13) | 4916 | 6487 | 7659 | 49 |
| H(14) | 6299 | 7223 | 8304 | 58 |
| H(15) | 8333 | 5820 | 8692 | 57 |
| H(16) | 8999 | 3681 | 8422 | 46 |
| H(21) | 10931 | 117 | 9255 | 48 |
| H(23) | 11846 | 383 | 6996 | 44 |
| H(25A) | 8763 | 1895 | 9702 | 67 |
| H(25B) | 8257 | 790 | 9508 | 67 |
| H(25C) | 7586 | 2181 | 9127 | 67 |
| H(26A) | 13593 | -440 | 7985 | 92 |
| H(26B) | 13111 | -1614 | 8104 | 92 |
| H(26C) | 13170 | -828 | 8828 | 92 |
| H(27A) | 10345 | 1781 | 6136 | 70 |
| H(27B) | 8977 | 2740 | 6457 | 70 |
| H(27C) | 9105 | 1351 | 6233 | 70 |
| H(34) | 4604 | 5622 | 5995 | 51 |
| H(35) | 4754 | 6615 | 4804 | 54 |
| H(36) | 3036 | 8443 | 4401 | 51 |
| H(37) | 1177 | 9267 | 5177 | 45 |
| H(41) | -1100(20) | 9230(20) | 7551(13) | 47 |
| H(43) | -2304 | 9260 | 8726 | 41 |
| H(44) | -2475 | 8428 | 9968 | 46 |
| H(45) | -821 | 6599 | 10419 | 44 |
| H(46) | 986 | 5587 | 9617 | 39 |
| H(51) | 4771 | 3455 | 9551 | 42 |
| H(53) | 3005 | 2027 | 8071 | 38 |

| | | | | |
|---|---|---|---|---|
| H(55A) | 4381 | 5898 | 9256 | 63 |
| H(55B) | 3314 | 5834 | 9904 | 63 |
| H(55C) | 2867 | 6705 | 9136 | 63 |
| H(56A) | 4405 | 676 | 9180 | 71 |
| H(56B) | 5380 | 1240 | 9544 | 71 |
| H(56C) | 5566 | 873 | 8658 | 71 |
| H(57A) | 1158 | 3333 | 7390 | 56 |
| H(57B) | 1705 | 4319 | 6938 | 56 |
| H(57C) | 514 | 4808 | 7552 | 56 |

Table 6. Hydrogen bonds for **QQ-Mes** [Å and °].

| D-H...A | d(D-H) | d(H...A) | d(D...A) | <(DHA) |
|---|---|---|---|---|
| N(11)-H(11)...N(32) | 0.92(2) | 2.10(2) | 2.998(2) | 168(2) |
| N(41)-H(41)...N(2)#1 | 0.96(2) | 2.14(2) | 3.088(2) | 168.6(19) |

Symmetry transformations used to generate equivalent atoms:

#1 x-1,y+1,z

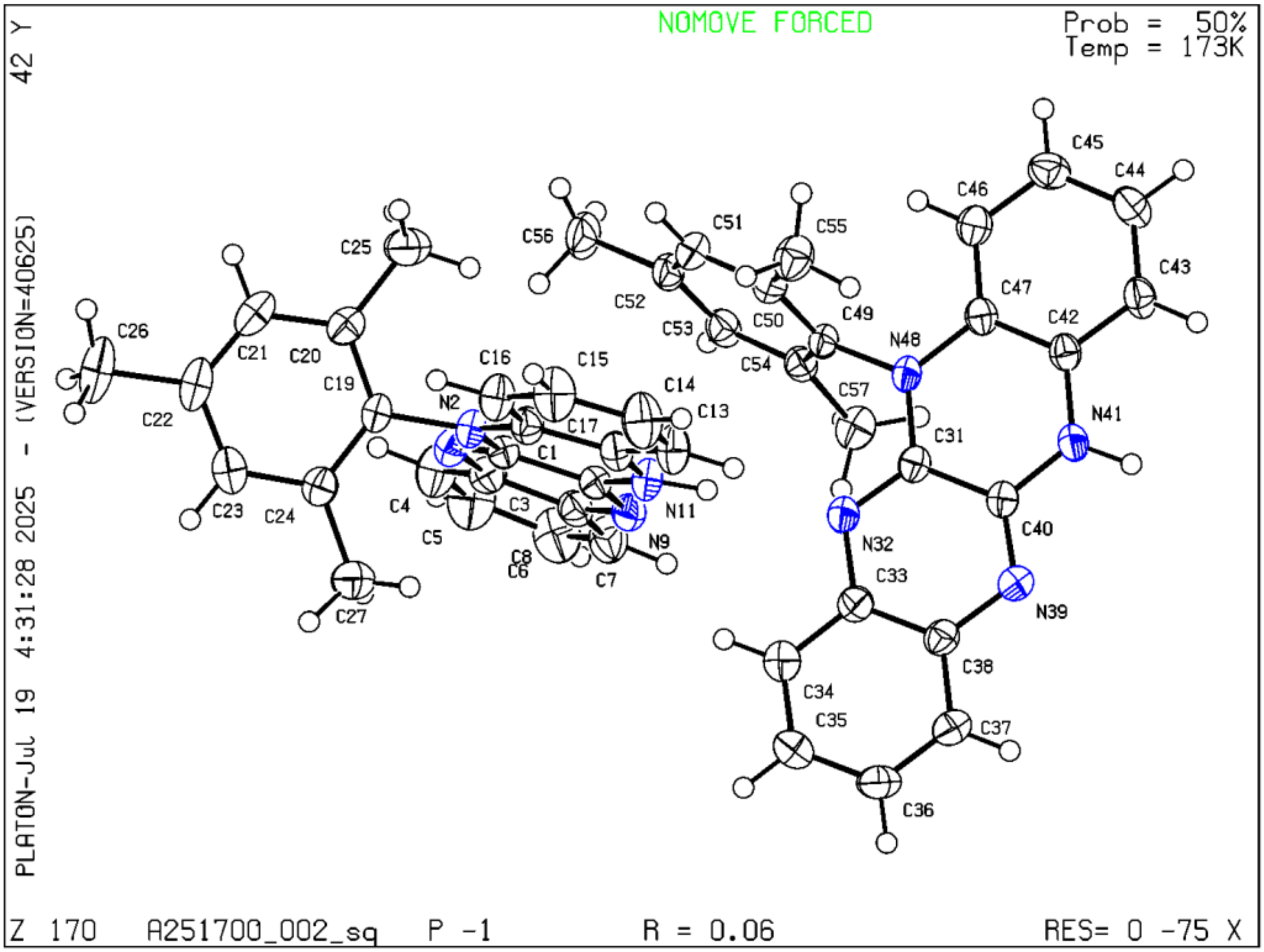


**Figure S50.** ORTEP diagram of **QQ-Mes** with 50% ellipsoid probability.

## AI-II. Crystallographic data of QQ-PyH-HOAc

Table 1. Crystal data and structure refinement for **QQ-PyH-HOAc**.

| | | |
|---|---|---|
| Empirical formula | $C_{21} H_{17} N_5 O_2$ | |
| Formula weight | 371.40 | |
| Temperature | 173(2) K | |
| Wavelength | 0.71073 Å | |
| Crystal system | Monoclinic | |
| Space group | $P2_1/c$ | |
| Unit cell dimensions | a = 17.5287(5) Å | α = 90° |
| | b = 4.8349(2) Å | β = 91.2351(14)° |
| | c = 20.6487(6) Å | γ = 90° |
| Volume | 1749.56(10) $Å^3$ | |
| Z | 4 | |
| Density (calculated) | 1.410 $Mg/m^3$ | |
| Absorption coefficient | 0.095 $mm^{-1}$ | |
| F(000) | 776 | |
| Crystal size | 0.188 x 0.026 x 0.023 $mm^3$ | |
| Theta range for data collection | 3.017 to 26.017°. | |
| Index ranges | –21<=h<=21, –5<=k<=5, –25<=l<=25 | |
| Reflections collected | 16918 | |
| Independent reflections | 3402 [R(int) = 0.0935] | |
| Completeness to theta = 25.242° | 99.5 % | |
| Absorption correction | Semi-empirical from equivalents | |
| Max. and min. transmission | 0.7453 and 0.6773 | |
| Refinement method | Full-matrix least-squares on $F^2$ | |
| Data / restraints / parameters | 3402 / 0 / 260 | |
| Goodness-of-fit on $F^2$ | 1.069 | |
| Final R indices [I>2sigma(I)] | R1 = 0.0512, wR2 = 0.1048 | |
| R indices (all data) | R1 = 0.0891, wR2 = 0.1198 | |
| Largest diff. peak and hole | 0.208 and –0.243 e·$Å^{-3}$ | |

Table 2. Atomic coordinates ( x $10^4$ ) and equivalent isotropic displacement parameters ( $Å^2$ x $10^3$ ) for **QQ-PyH-HOAc**. U(eq) is defined as one third of the trace of the orthogonalized $U^{ij}$ tensor.

| | x | y | z | U(eq) |
|---|---|---|---|---|
| C(1) | 7084(1) | 7171(5) | 2926(1) | 24(1) |
| N(2) | 6437(1) | 5986(4) | 2782(1) | 27(1) |
| C(3) | 6158(1) | 4115(5) | 3230(1) | 26(1) |
| C(4) | 5450(1) | 2874(5) | 3118(1) | 32(1) |
| C(5) | 5171(1) | 964(5) | 3546(1) | 36(1) |
| C(6) | 5602(1) | 224(5) | 4099(1) | 35(1) |
| C(7) | 6301(1) | 1453(5) | 4222(1) | 32(1) |
| C(8) | 6581(1) | 3429(5) | 3795(1) | 26(1) |
| N(9) | 7271(1) | 4723(4) | 3934(1) | 26(1) |
| C(10) | 7506(1) | 6606(5) | 3528(1) | 25(1) |
| N(11) | 8154(1) | 8000(4) | 3662(1) | 29(1) |
| C(12) | 8453(1) | 9970(5) | 3247(1) | 27(1) |
| C(13) | 9110(1) | 11429(5) | 3415(1) | 31(1) |
| C(14) | 9391(1) | 13431(5) | 3001(1) | 34(1) |
| C(15) | 9008(1) | 13974(5) | 2423(1) | 34(1) |
| C(16) | 8355(1) | 12520(5) | 2251(1) | 30(1) |
| C(17) | 8077(1) | 10481(5) | 2656(1) | 26(1) |
| N(18) | 7410(1) | 8983(4) | 2495(1) | 28(1) |
| C(19) | 7123(1) | 9014(5) | 1831(1) | 26(1) |
| C(20) | 6507(1) | 10573(6) | 1656(1) | 44(1) |
| C(21) | 6268(1) | 10516(7) | 1012(1) | 49(1) |
| C(22) | 6657(1) | 8922(5) | 583(1) | 34(1) |
| C(23) | 7277(2) | 7467(6) | 807(1) | 44(1) |
| N(24) | 7520(1) | 7496(5) | 1425(1) | 43(1) |
| O(25) | 8142(1) | 2470(4) | 4873(1) | 37(1) |
| O(26) | 8926(1) | 6071(4) | 4803(1) | 43(1) |
| C(27) | 8749(1) | 3896(5) | 5051(1) | 33(1) |
| C(28) | 9204(1) | 2577(7) | 5589(1) | 51(1) |

Table 3. Bond lengths [Å] and angles [°] for **QQ-PyH-HOAc**.

| | |
|---|---|
| C(1)-N(2) | 1.299(3) |
| C(1)-N(18) | 1.381(3) |
| C(1)-C(10) | 1.460(3) |
| N(2)-C(3) | 1.389(3) |
| C(3)-C(4) | 1.394(3) |
| C(3)-C(8) | 1.409(3) |
| C(4)-C(5) | 1.376(3) |
| C(4)-H(4) | 0.9500 |
| C(5)-C(6) | 1.401(3) |
| C(5)-H(5) | 0.9500 |
| C(6)-C(7) | 1.381(3) |
| C(6)-H(6) | 0.9500 |
| C(7)-C(8) | 1.396(3) |
| C(7)-H(7) | 0.9500 |
| C(8)-N(9) | 1.386(3) |
| N(9)-C(10) | 1.310(3) |
| C(10)-N(11) | 1.345(3) |
| N(11)-C(12) | 1.390(3) |
| N(11)-H(11) | 0.86(3) |
| C(12)-C(13) | 1.389(3) |
| C(12)-C(17) | 1.396(3) |
| C(13)-C(14) | 1.389(3) |
| C(13)-H(13) | 0.9500 |
| C(14)-C(15) | 1.381(3) |
| C(14)-H(14) | 0.9500 |
| C(15)-C(16) | 1.384(3) |
| C(15)-H(15) | 0.9500 |
| C(16)-C(17) | 1.388(3) |
| C(16)-H(16) | 0.9500 |
| C(17)-N(18) | 1.409(3) |
| N(18)-C(19) | 1.450(2) |
| C(19)-N(24) | 1.322(3) |
| C(19)-C(20) | 1.358(3) |
| C(20)-C(21) | 1.386(3) |

| | |
|---|---|
| C(20)-H(20) | 0.9500 |
| C(21)-C(22) | 1.368(3) |
| C(21)-H(21) | 0.9500 |
| C(22)-C(23) | 1.367(3) |
| C(22)-H(22) | 0.9500 |
| C(23)-N(24) | 1.338(3) |
| C(23)-H(23) | 0.9500 |
| O(25)-C(27) | 1.313(3) |
| O(25)-H(25) | 0.99(3) |
| O(26)-C(27) | 1.213(3) |
| C(27)-C(28) | 1.496(3) |
| C(28)-H(28A) | 0.9800 |
| C(28)-H(28B) | 0.9800 |
| C(28)-H(28C) | 0.9800 |
| | |
| N(2)-C(1)-N(18) | 120.29(17) |
| N(2)-C(1)-C(10) | 122.37(18) |
| N(18)-C(1)-C(10) | 117.32(17) |
| C(1)-N(2)-C(3) | 116.97(16) |
| N(2)-C(3)-C(4) | 119.65(17) |
| N(2)-C(3)-C(8) | 121.21(18) |
| C(4)-C(3)-C(8) | 119.1(2) |
| C(5)-C(4)-C(3) | 120.64(19) |
| C(5)-C(4)-H(4) | 119.7 |
| C(3)-C(4)-H(4) | 119.7 |
| C(4)-C(5)-C(6) | 120.2(2) |
| C(4)-C(5)-H(5) | 119.9 |
| C(6)-C(5)-H(5) | 119.9 |
| C(7)-C(6)-C(5) | 120.0(2) |
| C(7)-C(6)-H(6) | 120.0 |
| C(5)-C(6)-H(6) | 120.0 |
| C(6)-C(7)-C(8) | 120.15(19) |
| C(6)-C(7)-H(7) | 119.9 |
| C(8)-C(7)-H(7) | 119.9 |
| N(9)-C(8)-C(7) | 119.70(17) |
| N(9)-C(8)-C(3) | 120.44(19) |

| | |
|---|---|
| C(7)-C(8)-C(3) | 119.86(19) |
| C(10)-N(9)-C(8) | 117.73(16) |
| N(9)-C(10)-N(11) | 119.50(17) |
| N(9)-C(10)-C(1) | 121.03(18) |
| N(11)-C(10)-C(1) | 119.45(18) |
| C(10)-N(11)-C(12) | 122.99(17) |
| C(10)-N(11)-H(11) | 115.7(19) |
| C(12)-N(11)-H(11) | 121.3(19) |
| C(13)-C(12)-N(11) | 121.03(18) |
| C(13)-C(12)-C(17) | 120.1(2) |
| N(11)-C(12)-C(17) | 118.86(19) |
| C(12)-C(13)-C(14) | 120.18(19) |
| C(12)-C(13)-H(13) | 119.9 |
| C(14)-C(13)-H(13) | 119.9 |
| C(15)-C(14)-C(13) | 119.6(2) |
| C(15)-C(14)-H(14) | 120.2 |
| C(13)-C(14)-H(14) | 120.2 |
| C(14)-C(15)-C(16) | 120.6(2) |
| C(14)-C(15)-H(15) | 119.7 |
| C(16)-C(15)-H(15) | 119.7 |
| C(15)-C(16)-C(17) | 120.34(19) |
| C(15)-C(16)-H(16) | 119.8 |
| C(17)-C(16)-H(16) | 119.8 |
| C(16)-C(17)-C(12) | 119.18(19) |
| C(16)-C(17)-N(18) | 121.48(17) |
| C(12)-C(17)-N(18) | 119.30(19) |
| C(1)-N(18)-C(17) | 121.72(16) |
| C(1)-N(18)-C(19) | 118.39(17) |
| C(17)-N(18)-C(19) | 119.21(16) |
| N(24)-C(19)-C(20) | 124.48(18) |
| N(24)-C(19)-N(18) | 114.57(18) |
| C(20)-C(19)-N(18) | 120.92(19) |
| C(19)-C(20)-C(21) | 117.8(2) |
| C(19)-C(20)-H(20) | 121.1 |
| C(21)-C(20)-H(20) | 121.1 |
| C(22)-C(21)-C(20) | 119.2(2) |

| | |
|---|---|
| C(22)-C(21)-H(21) | 120.4 |
| C(20)-C(21)-H(21) | 120.4 |
| C(23)-C(22)-C(21) | 118.34(19) |
| C(23)-C(22)-H(22) | 120.8 |
| C(21)-C(22)-H(22) | 120.8 |
| N(24)-C(23)-C(22) | 123.5(2) |
| N(24)-C(23)-H(23) | 118.3 |
| C(22)-C(23)-H(23) | 118.3 |
| C(19)-N(24)-C(23) | 116.7(2) |
| C(27)-O(25)-H(25) | 113.3(18) |
| O(26)-C(27)-O(25) | 123.38(19) |
| O(26)-C(27)-C(28) | 123.0(2) |
| O(25)-C(27)-C(28) | 113.6(2) |
| C(27)-C(28)-H(28A) | 109.5 |
| C(27)-C(28)-H(28B) | 109.5 |
| H(28A)-C(28)-H(28B) | 109.5 |
| C(27)-C(28)-H(28C) | 109.5 |
| H(28A)-C(28)-H(28C) | 109.5 |
| H(28B)-C(28)-H(28C) | 109.5 |

Symmetry transformations used to generate equivalent atoms:

Table 4. Anisotropic displacement parameters ( $Å^2 \times 10^3$ ) for **QQ-PyH-HOAc**. The anisotropic displacement factor exponent takes the form: $-2\pi^2[ h^2a^{*2}U^{11} + ... + 2 h k a^* b^* U^{12} ]$

| | $U^{11}$ | $U^{22}$ | $U^{33}$ | $U^{23}$ | $U^{13}$ | $U^{12}$ |
|---|---|---|---|---|---|---|
| C(1) | 27(1) | 24(1) | 19(1) | 0(1) | 1(1) | 4(1) |
| N(2) | 30(1) | 29(1) | 23(1) | 2(1) | 1(1) | 1(1) |
| C(3) | 30(1) | 25(1) | 23(1) | -2(1) | 1(1) | 2(1) |
| C(4) | 35(1) | 36(1) | 25(1) | -1(1) | -4(1) | -2(1) |
| C(5) | 36(1) | 37(1) | 35(1) | 1(1) | -2(1) | -12(1) |
| C(6) | 43(1) | 32(1) | 31(1) | 5(1) | 0(1) | -9(1) |
| C(7) | 37(1) | 36(1) | 24(1) | 5(1) | -3(1) | -2(1) |
| C(8) | 30(1) | 24(1) | 22(1) | -3(1) | 0(1) | 1(1) |
| N(9) | 28(1) | 28(1) | 22(1) | 3(1) | -1(1) | 0(1) |
| C(10) | 24(1) | 28(1) | 22(1) | -2(1) | 2(1) | 4(1) |
| N(11) | 31(1) | 32(1) | 23(1) | 5(1) | -4(1) | -1(1) |
| C(12) | 26(1) | 28(1) | 26(1) | 1(1) | 2(1) | 4(1) |
| C(13) | 30(1) | 32(1) | 30(1) | 0(1) | -2(1) | 2(1) |
| C(14) | 30(1) | 30(1) | 42(1) | -2(1) | 4(1) | -2(1) |
| C(15) | 36(1) | 30(1) | 37(1) | 5(1) | 8(1) | 0(1) |
| C(16) | 35(1) | 28(1) | 26(1) | 3(1) | 4(1) | 4(1) |
| C(17) | 26(1) | 28(1) | 25(1) | -1(1) | 3(1) | 3(1) |
| N(18) | 30(1) | 31(1) | 21(1) | 3(1) | -1(1) | -1(1) |
| C(19) | 31(1) | 27(1) | 21(1) | 3(1) | 1(1) | -1(1) |
| C(20) | 41(1) | 64(2) | 28(1) | -12(1) | -4(1) | 20(1) |
| C(21) | 44(1) | 70(2) | 32(1) | -9(1) | -11(1) | 26(1) |
| C(22) | 46(1) | 38(1) | 20(1) | -1(1) | -3(1) | 0(1) |
| C(23) | 62(2) | 46(2) | 26(1) | -5(1) | 2(1) | 18(1) |
| N(24) | 57(1) | 45(1) | 27(1) | 1(1) | -1(1) | 22(1) |
| O(25) | 34(1) | 42(1) | 35(1) | 10(1) | -7(1) | -5(1) |
| O(26) | 41(1) | 44(1) | 42(1) | 10(1) | -11(1) | -9(1) |
| C(27) | 31(1) | 41(2) | 26(1) | 3(1) | 0(1) | 0(1) |
| C(28) | 43(1) | 66(2) | 42(1) | 21(1) | -12(1) | -8(1) |

Table 5. Hydrogen coordinates ( x $10^4$ ) and isotropic displacement parameters ( $Å^2$ x $10^3$ ) for **QQ-PyH-HOAc**.

| | x | y | z | U(eq) |
|---|---|---|---|---|
| H(4) | 5157 | 3352 | 2742 | 38 |
| H(5) | 4686 | 144 | 3467 | 43 |
| H(6) | 5413 | -1124 | 4389 | 42 |
| H(7) | 6592 | 953 | 4597 | 39 |
| H(11) | 8382(14) | 7580(60) | 4023(12) | 43 |
| H(13) | 9369 | 11057 | 3815 | 37 |
| H(14) | 9842 | 14420 | 3114 | 40 |
| H(15) | 9196 | 15358 | 2141 | 41 |
| H(16) | 8095 | 12919 | 1853 | 35 |
| H(20) | 6250 | 11665 | 1965 | 53 |
| H(21) | 5839 | 11572 | 871 | 59 |
| H(22) | 6500 | 8828 | 140 | 41 |
| H(23) | 7551 | 6378 | 507 | 53 |
| H(25) | 7825(16) | 3410(70) | 4535(13) | 56 |
| H(28A) | 9744 | 3056 | 5546 | 76 |
| H(28B) | 9144 | 564 | 5566 | 76 |
| H(28C) | 9023 | 3247 | 6006 | 76 |

Table 6. Hydrogen bonds for **QQ-PyH-HOAc** [Å and °].

| D-H...A | d(D-H) | d(H...A) | d(D...A) | <(DHA) |
|---|---|---|---|---|
| N(11)-H(11)...O(26) | 0.86(3) | 1.99(3) | 2.849(2) | 172(3) |
| O(25)-H(25)...N(9) | 0.99(3) | 1.68(3) | 2.673(2) | 175(3) |

Symmetry transformations used to generate equivalent atoms:

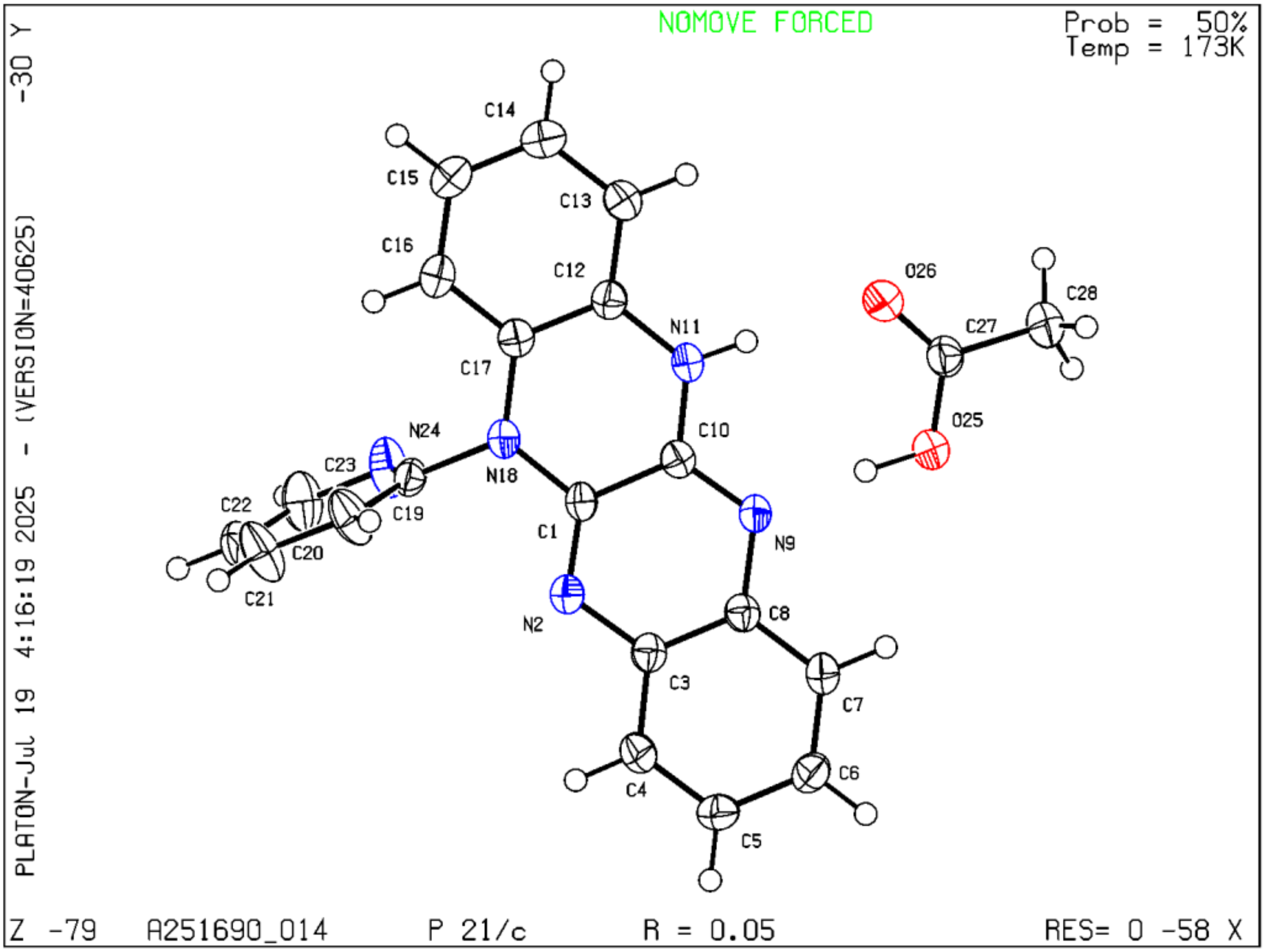


**Figure S51.** ORTEP diagram of **QQ-Py-HOAc** with 50% ellipsoid probability.

# *Appendix II.*
# *Copy of $^{1}H$, $^{13}C\{^{1}H\}$, and $^{19}F$ NMR spectra of obtained chemical species*

$N^1$-Mesitylbenzene-1,2-diamine (**1b**)

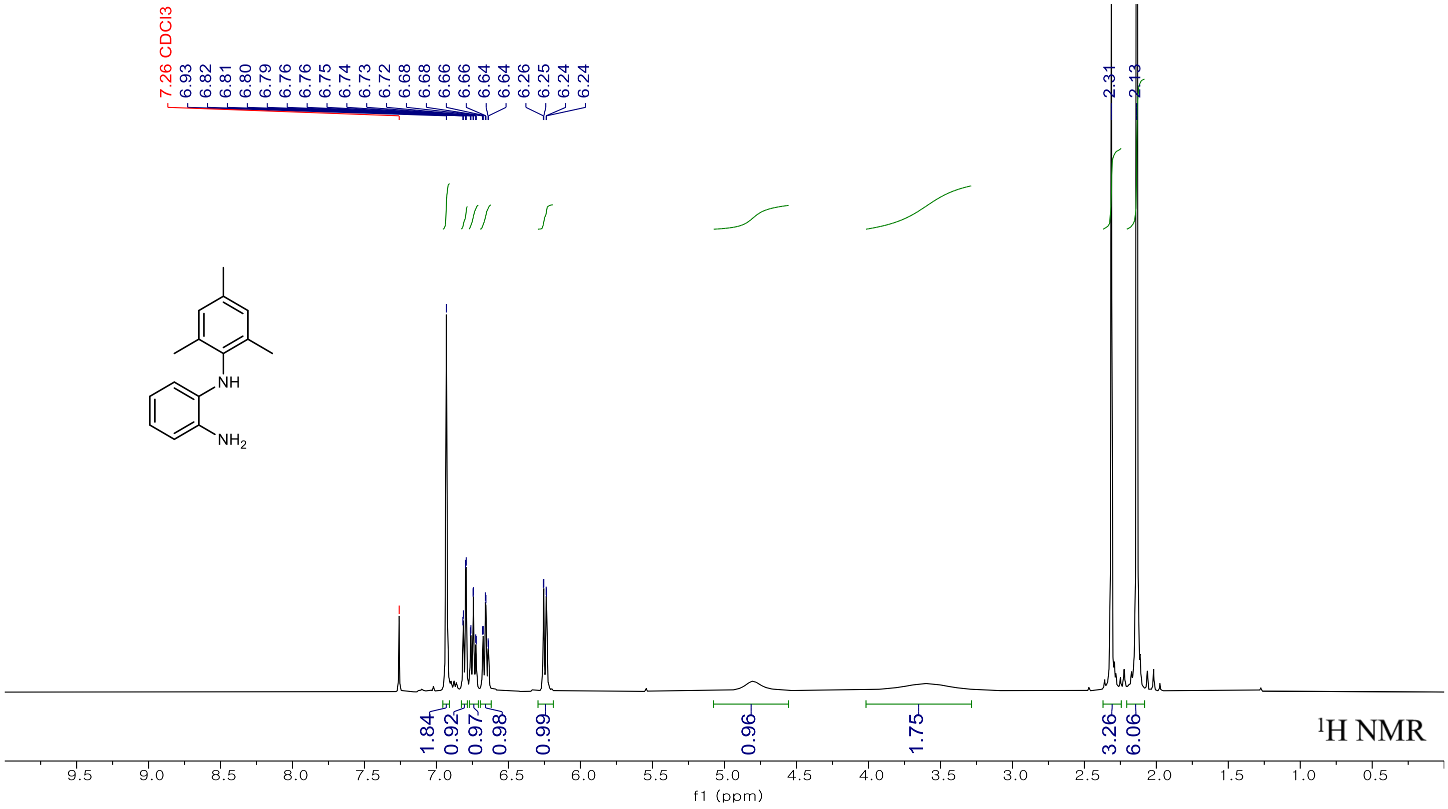


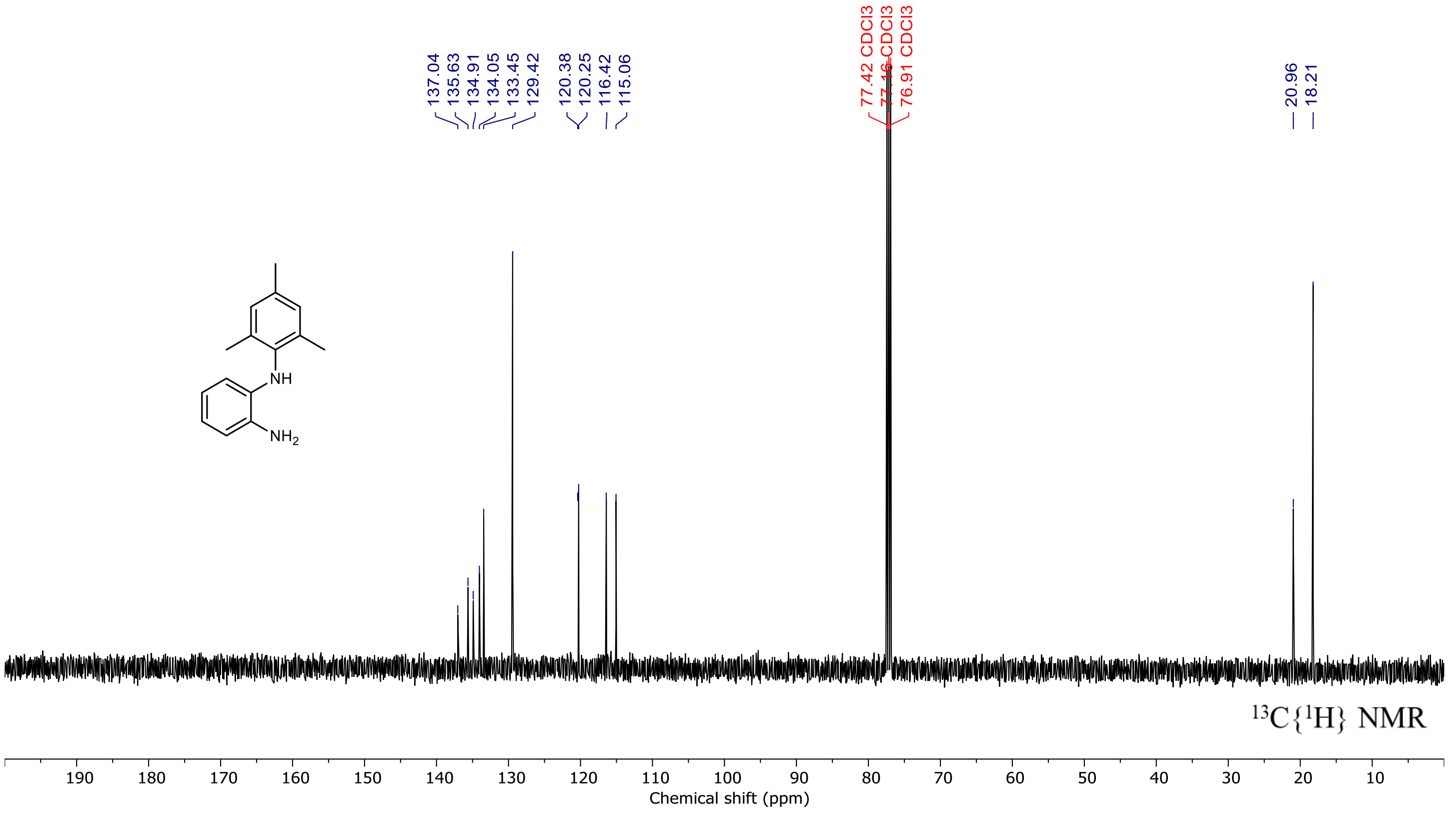

$N^1$-(Pyridin-2-yl)benzene-1,2-diamine (**1c**)

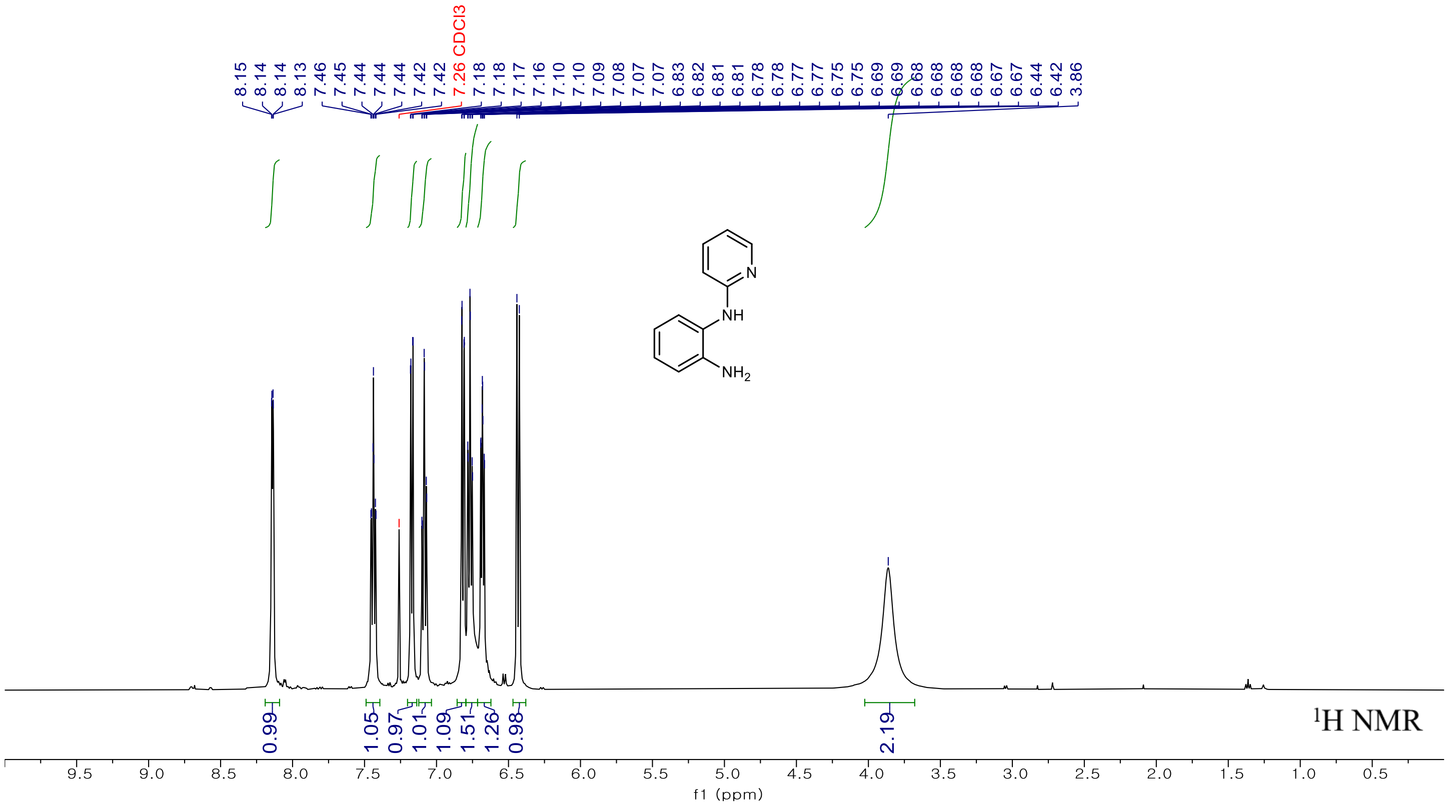


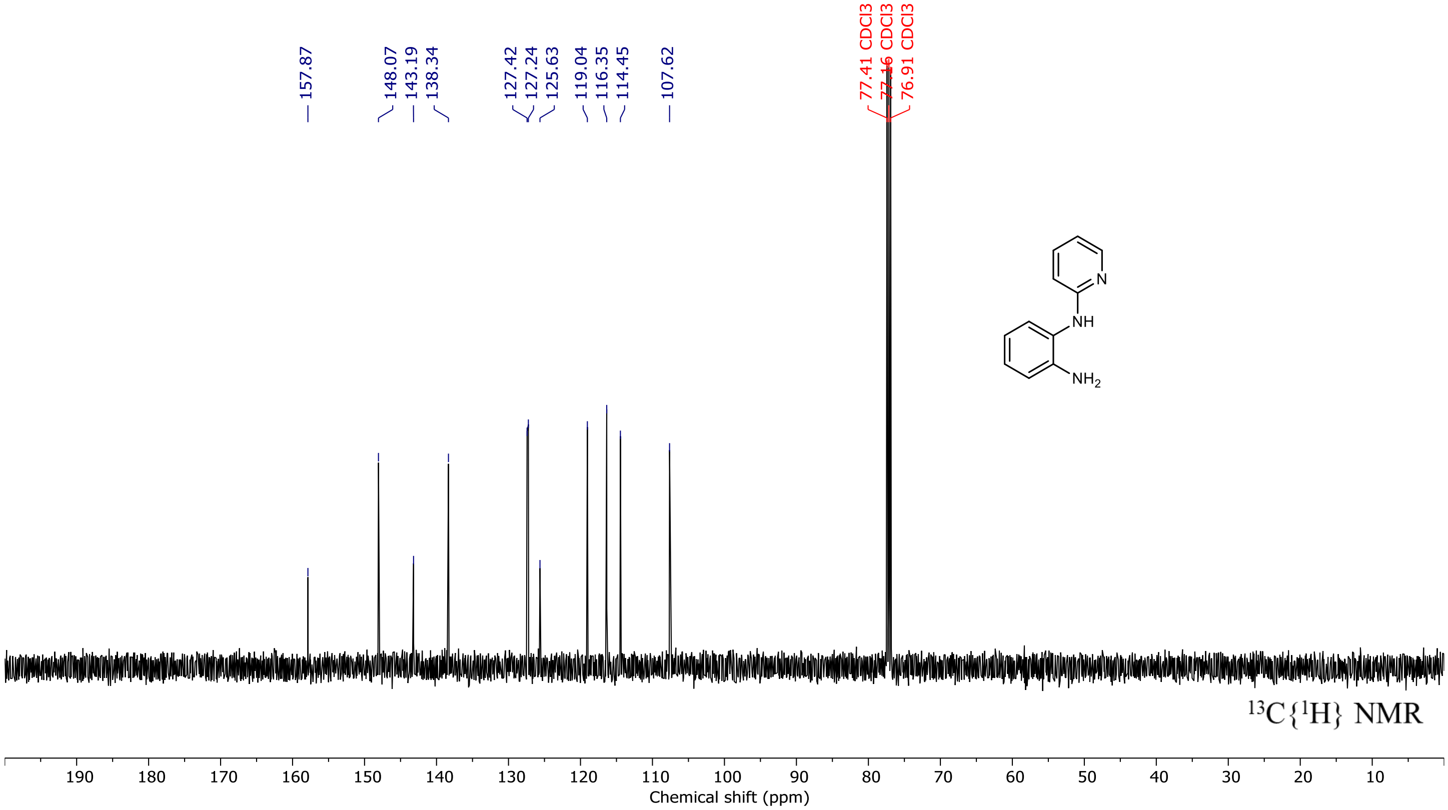

*N*[1]-(5-Methylpyridin-2-yl)benzene-1,2-diamine (**1d**)

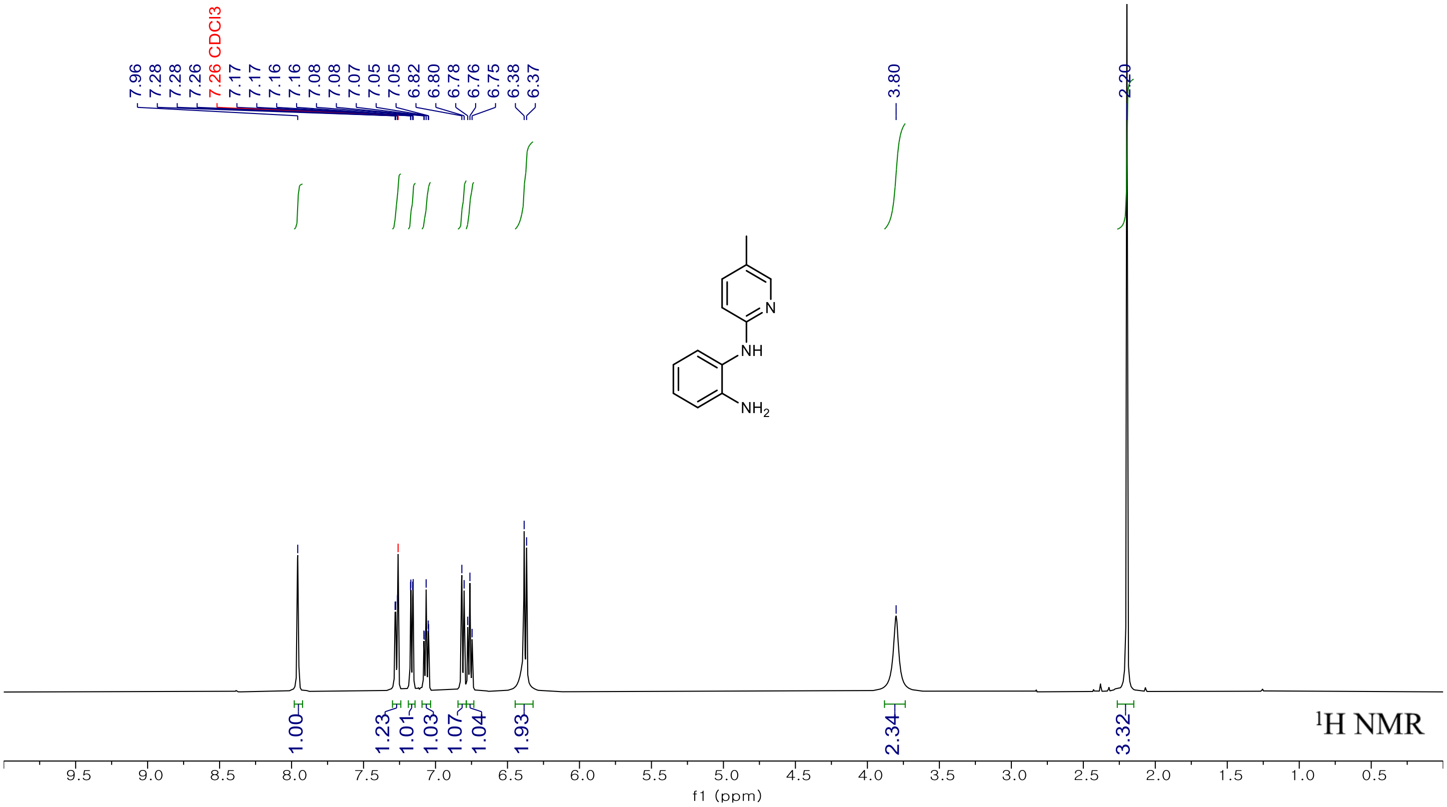


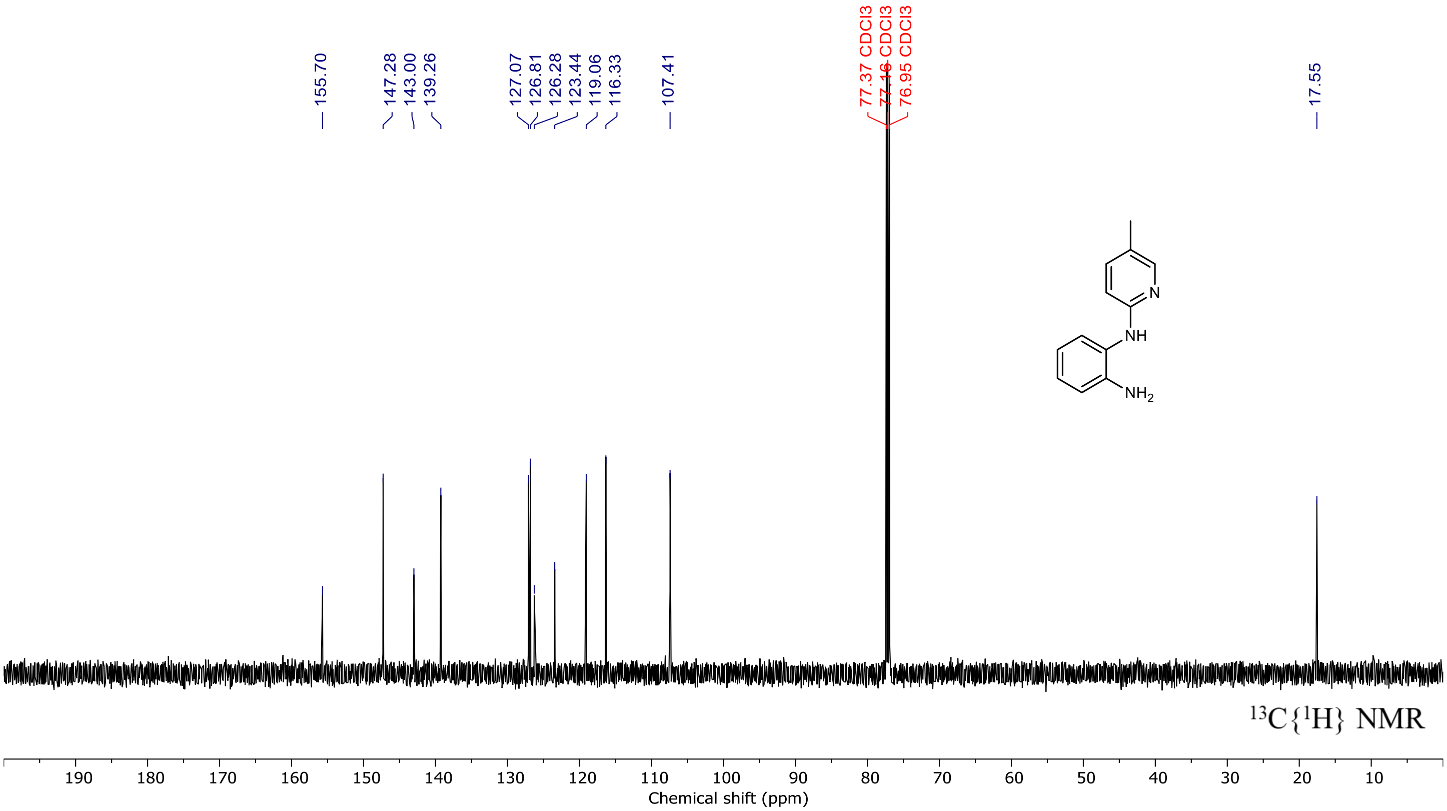

*N*[1]-(5-Chloropyridin-2-yl)benzene-1,2-diamine (**1e**)

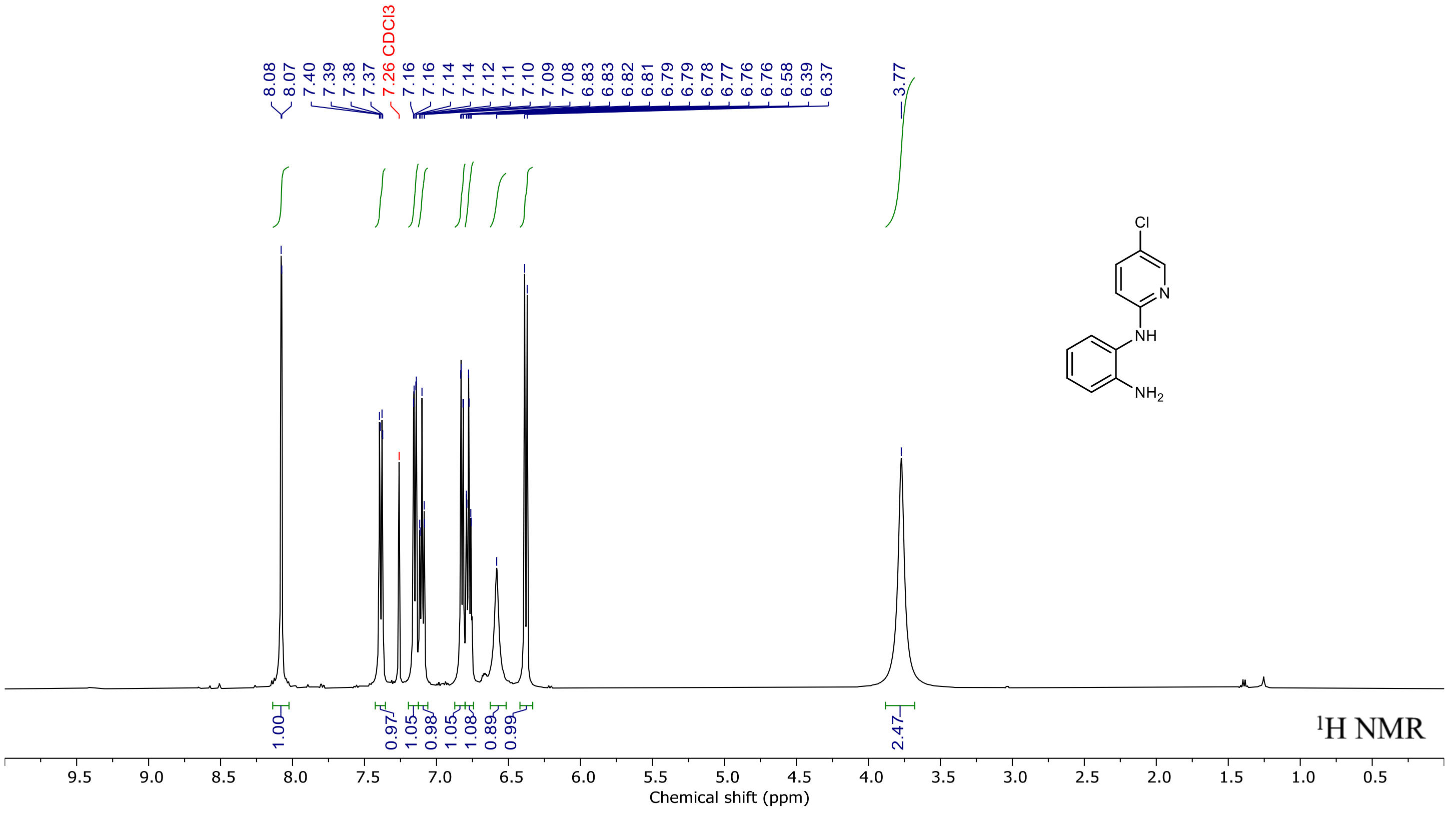


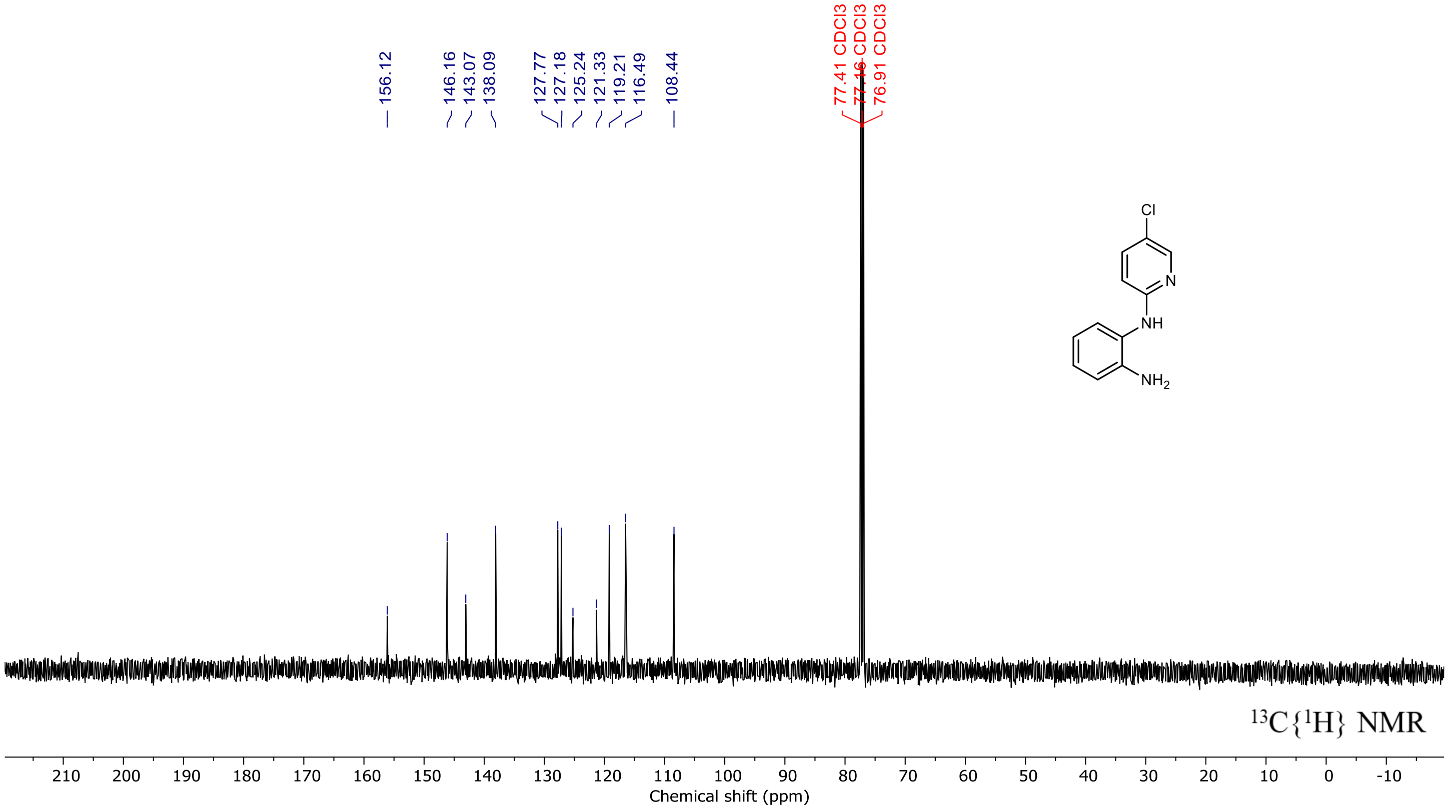

*N*[1]-[5-(Trifluoromethyl)pyridin-2-yl]benzene-1,2-diamine (**1f**)

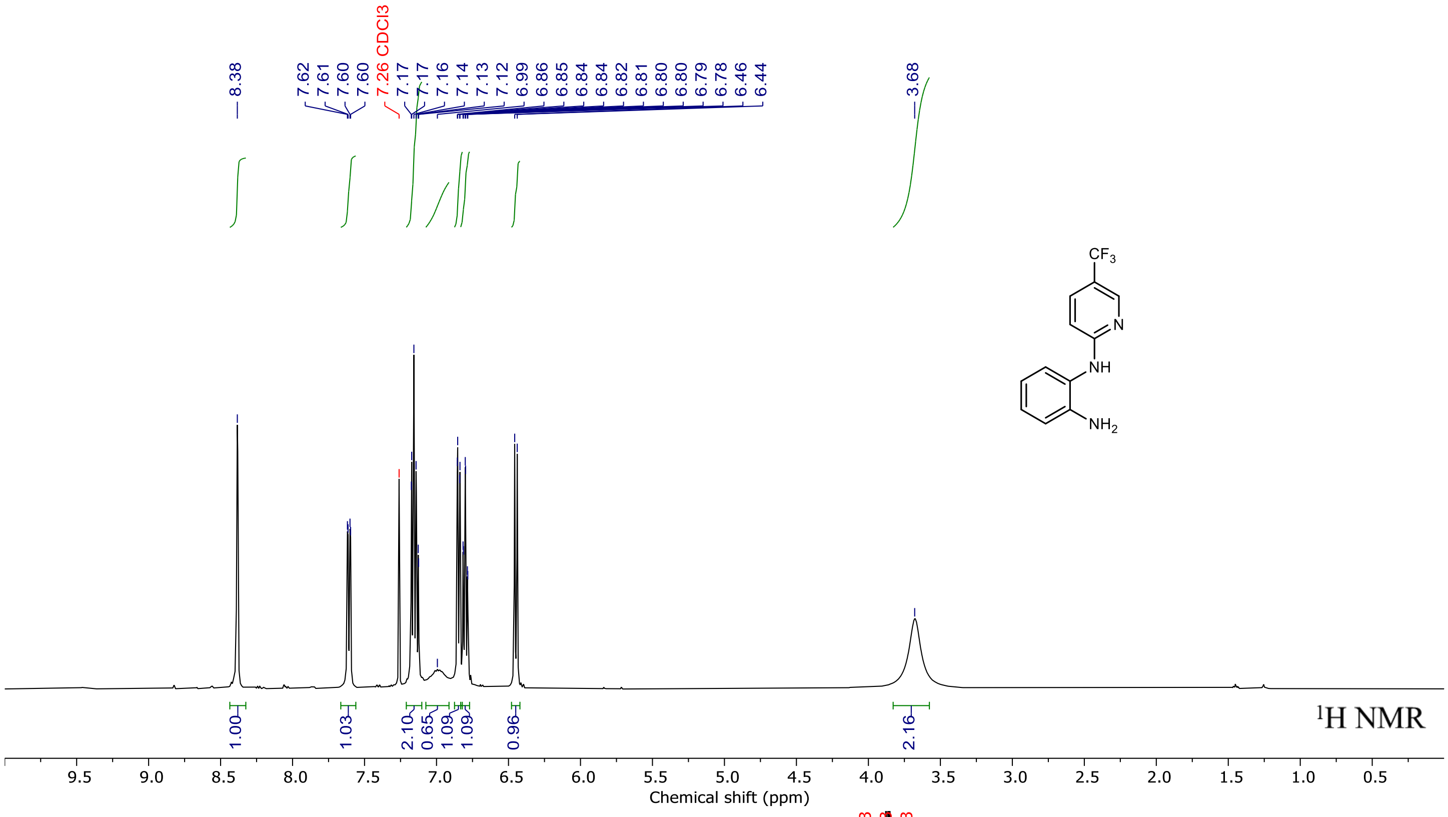


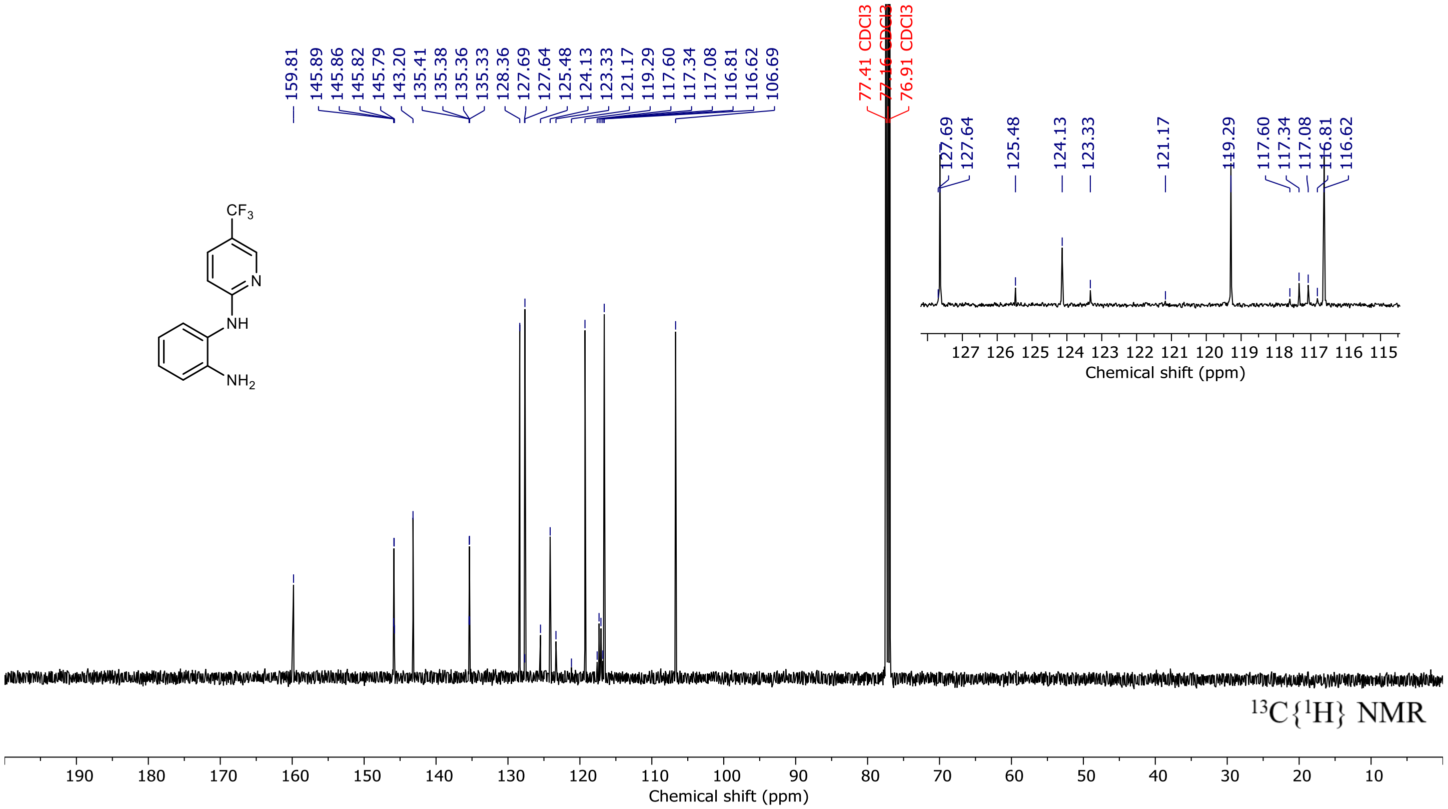

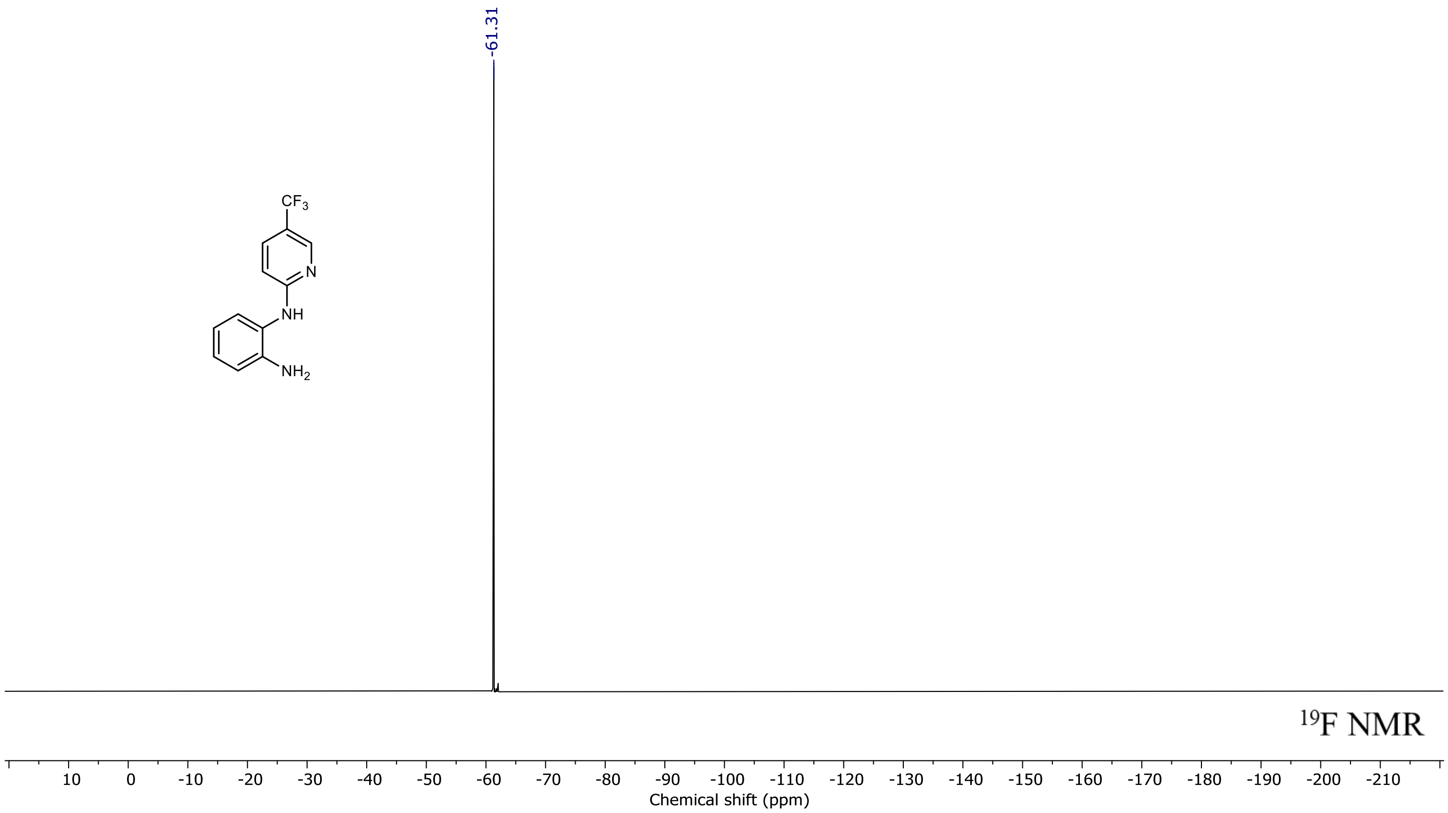
-61.31
CF3
N
NH
NH2
19F NMR
10 0 -10 -20 -30 -40 -50 -60 -70 -80 -90 -100 -110 -120 -130 -140 -150 -160 -170 -180 -190 -200 -210
Chemical shift (ppm)

5-Phenyl-5,12-dihydroquinoxalino[2,3-b]quinoxaline (**QQ-Ph**)

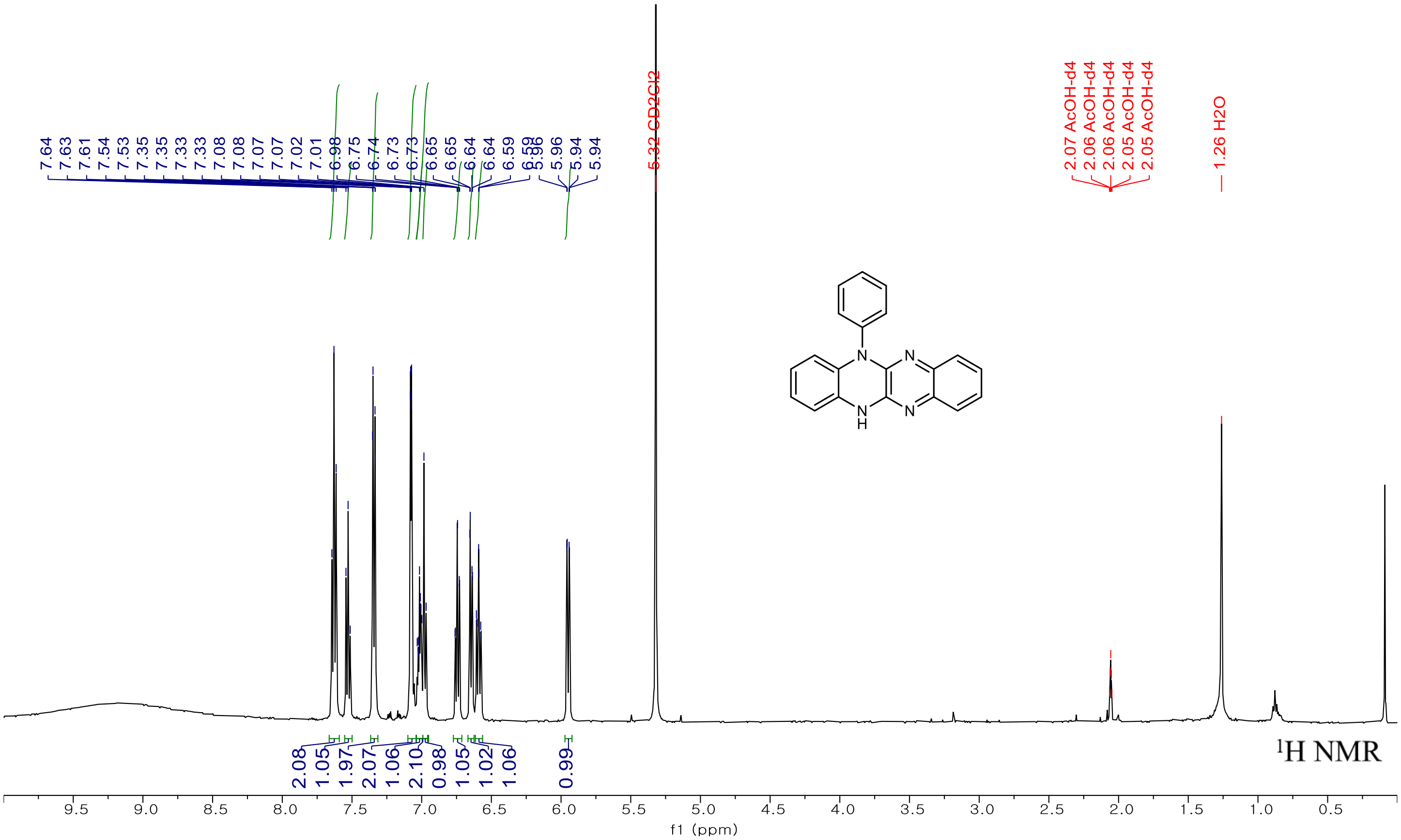


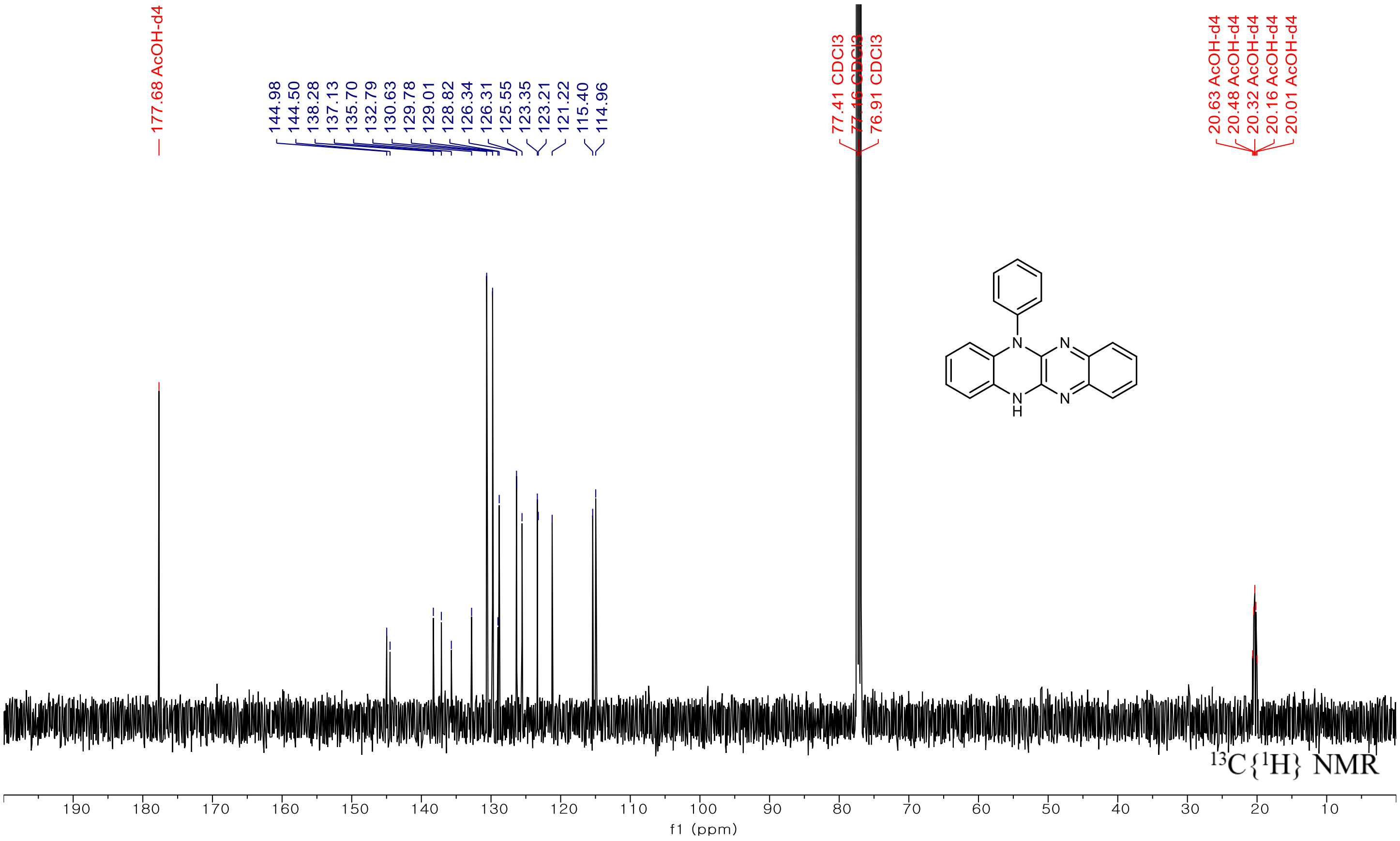

5-Mesityl-5,12-dihydroquinoxalino[2,3-b]quinoxaline (**QQ-Mes**)

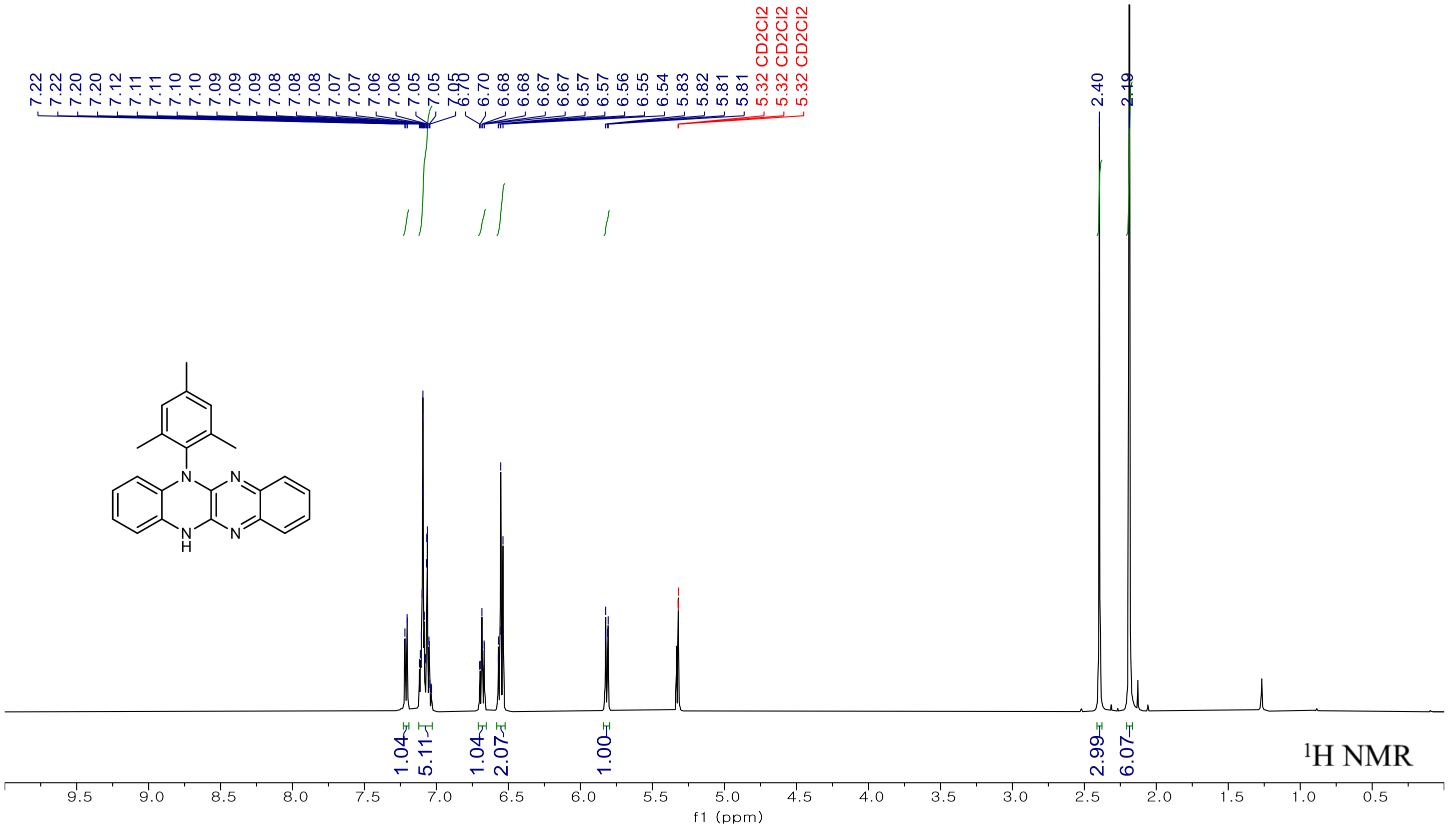


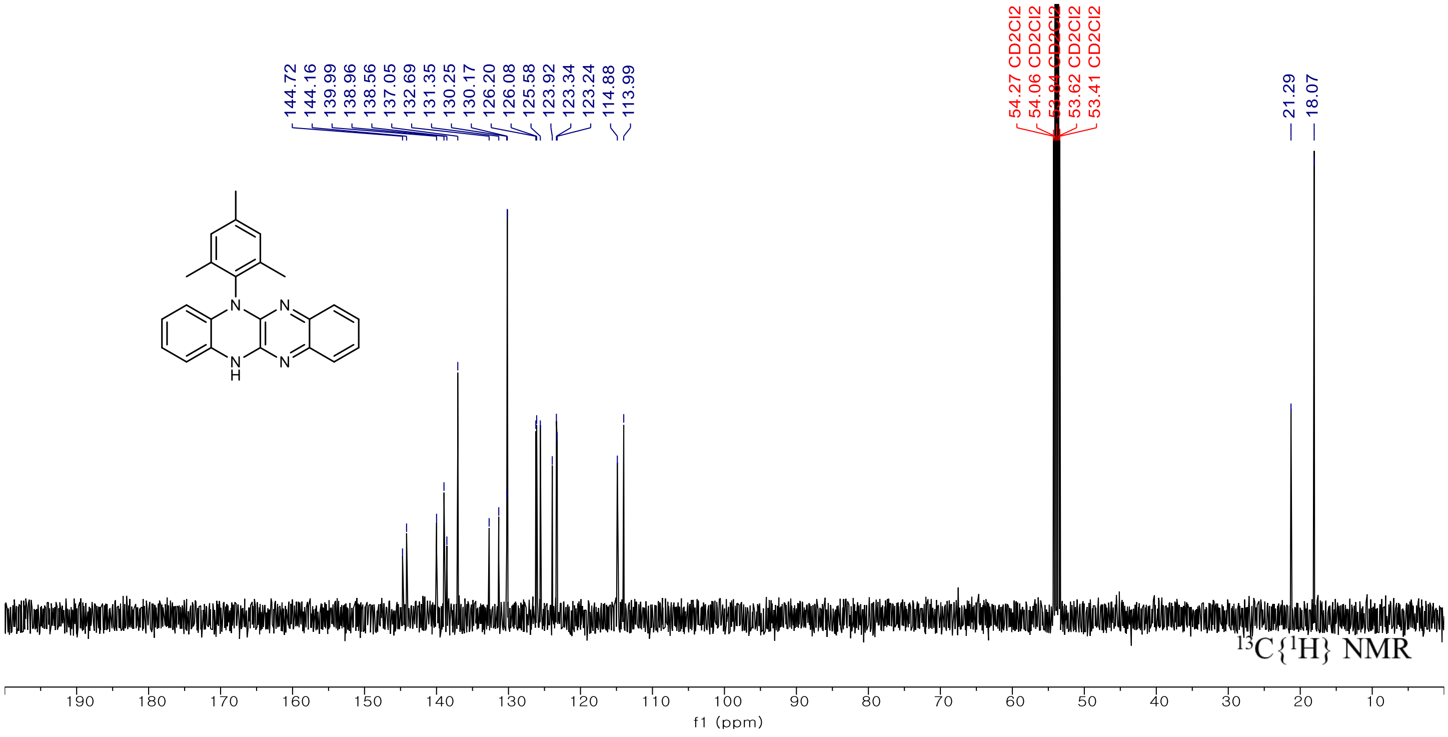

5-(Pyridin-2-yl)-5,12-dihydroquinoxalino[2,3-b]quinoxaline (**QQ-PyH**)

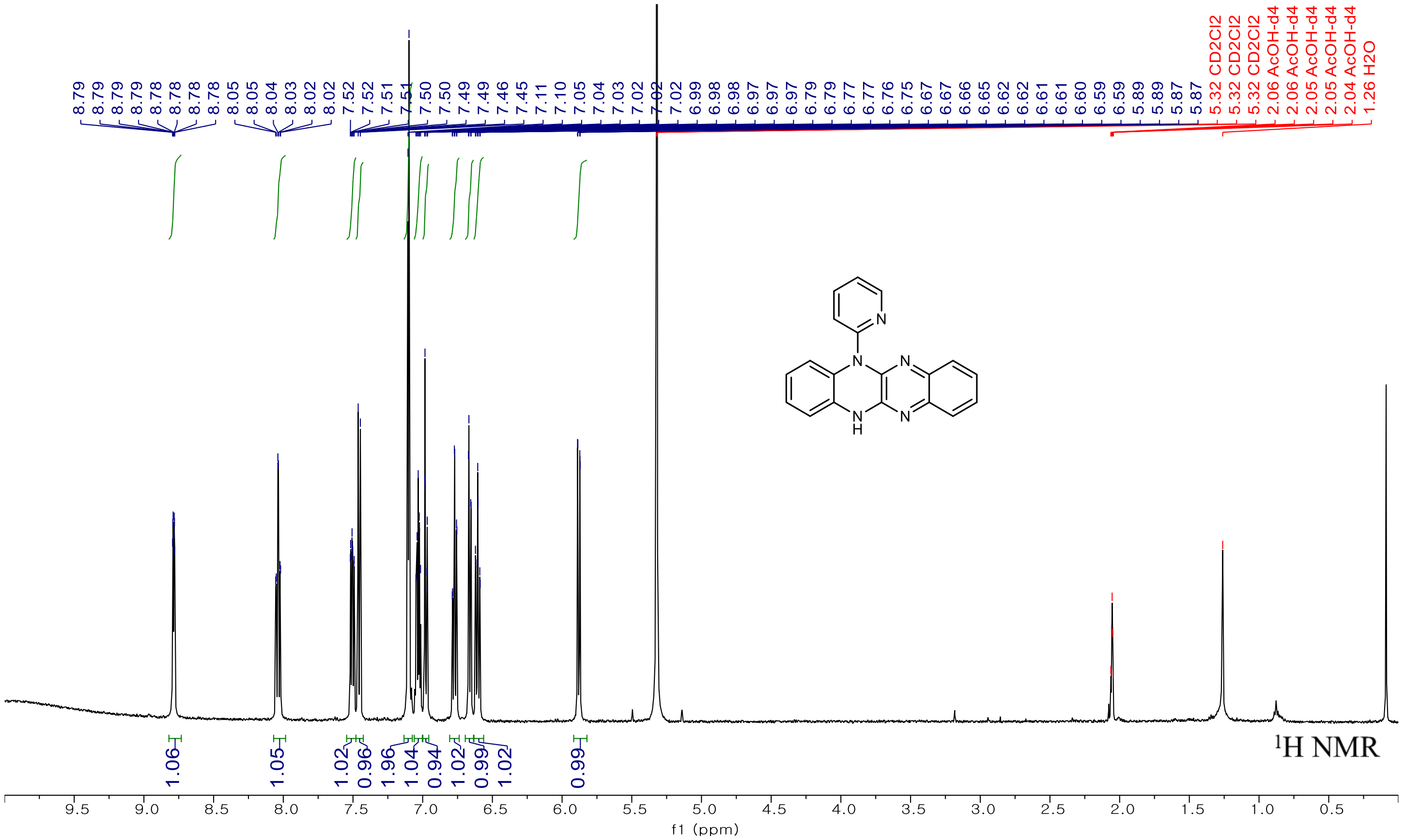


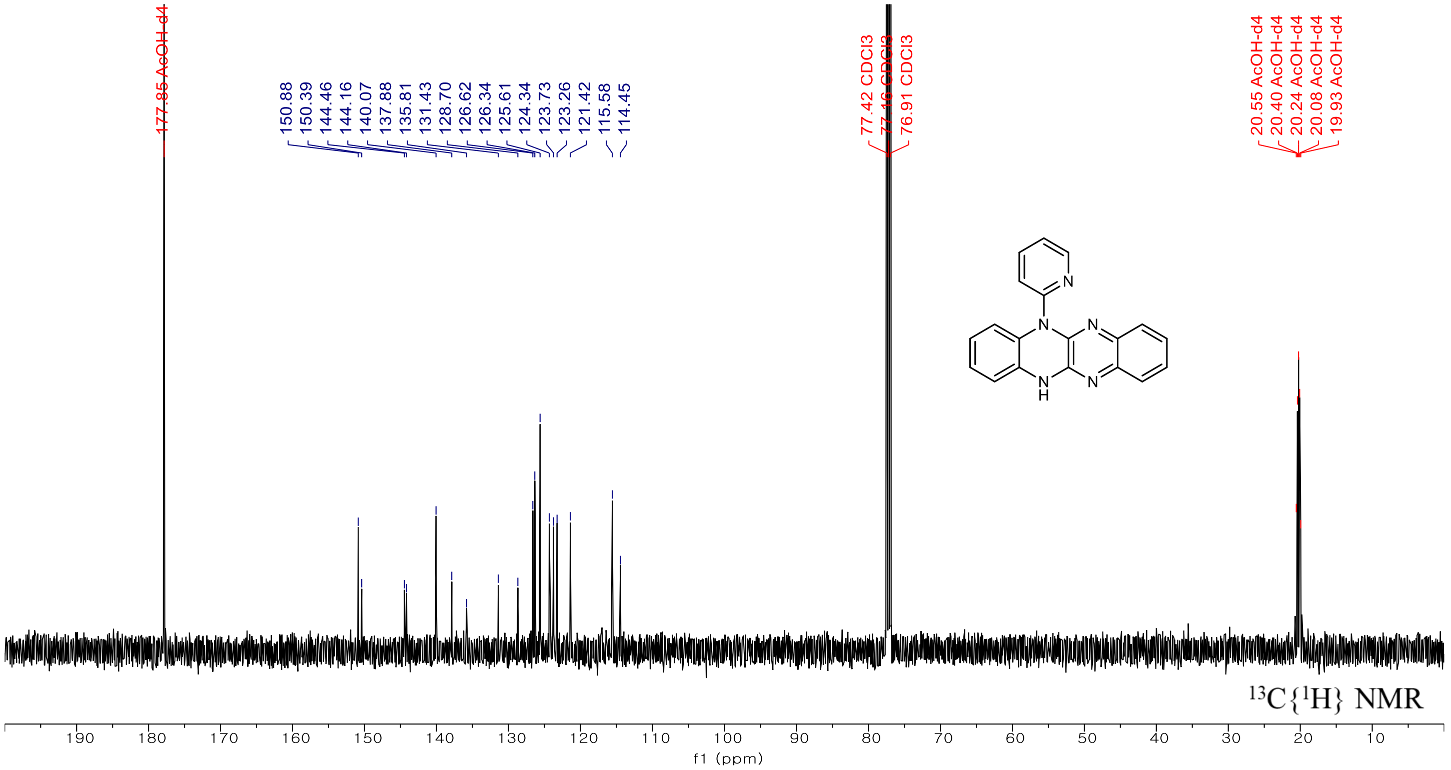

5-(5-Methylpyridin-2-yl)-5,12-dihydroquinoxalino[2,3-b]quinoxaline (**QQ-PyMe**)

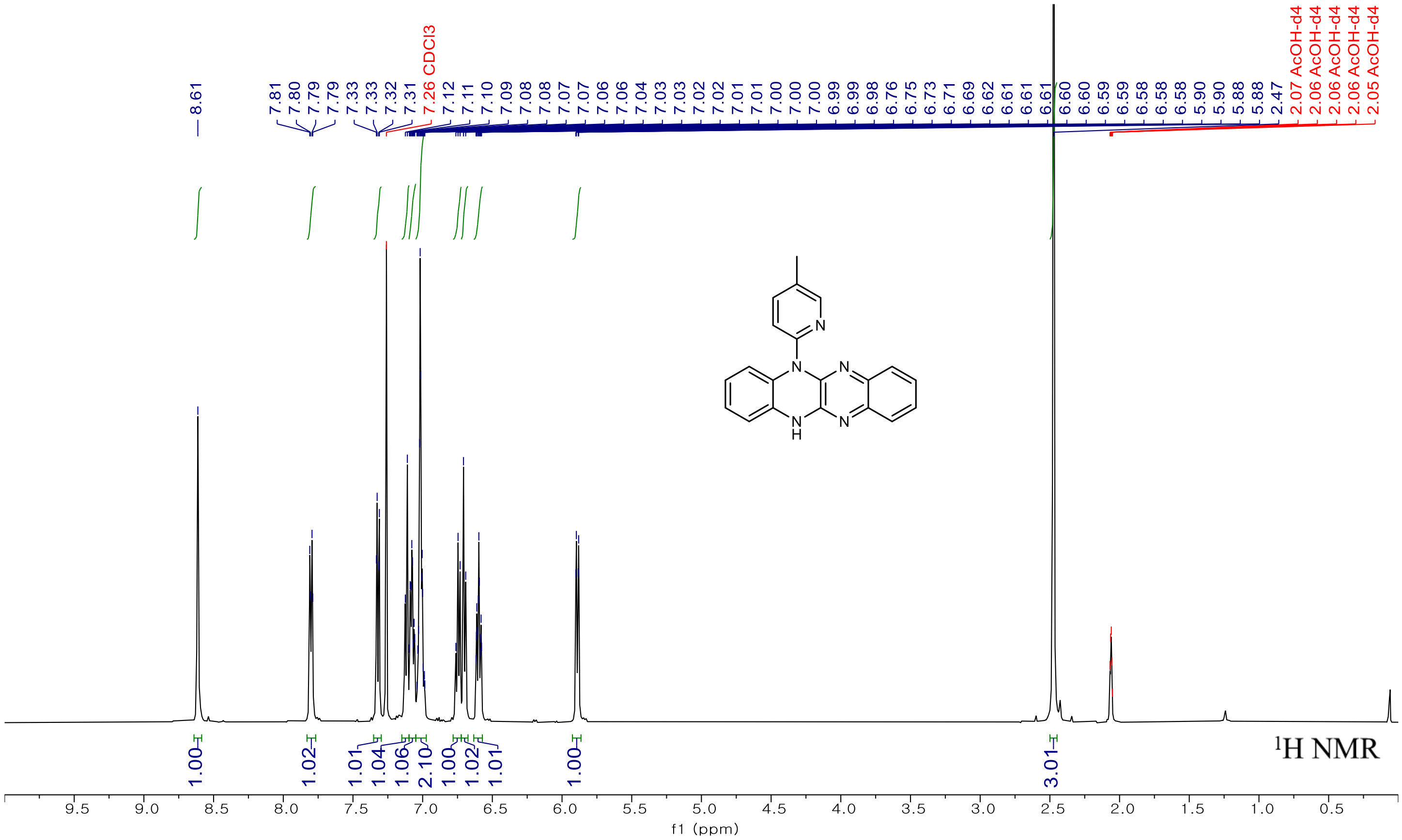


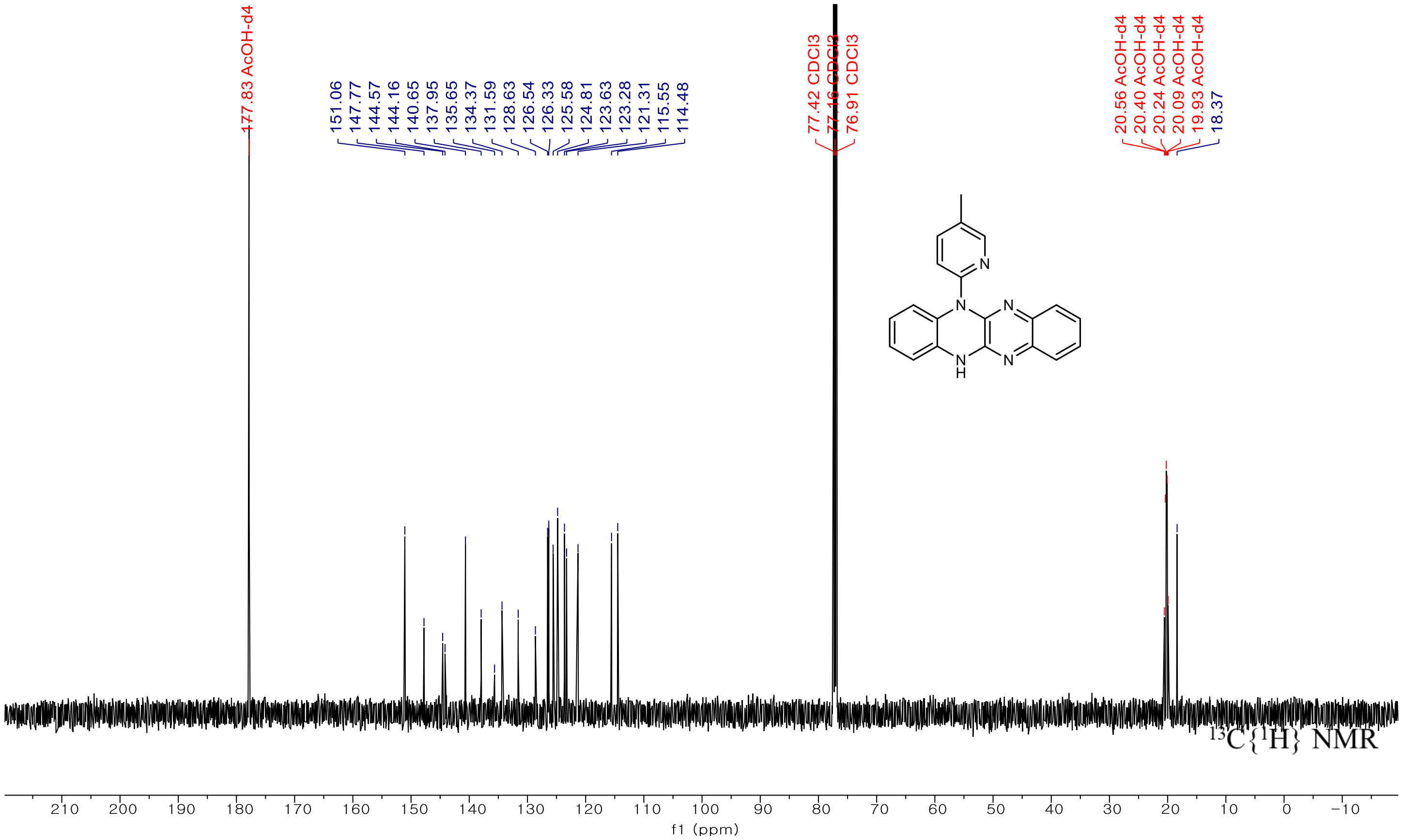

5-(5-Chloropyridin-2-yl)-5,12-dihydroquinoxalino[2,3-b]quinoxaline (**QQ-PyCl**)

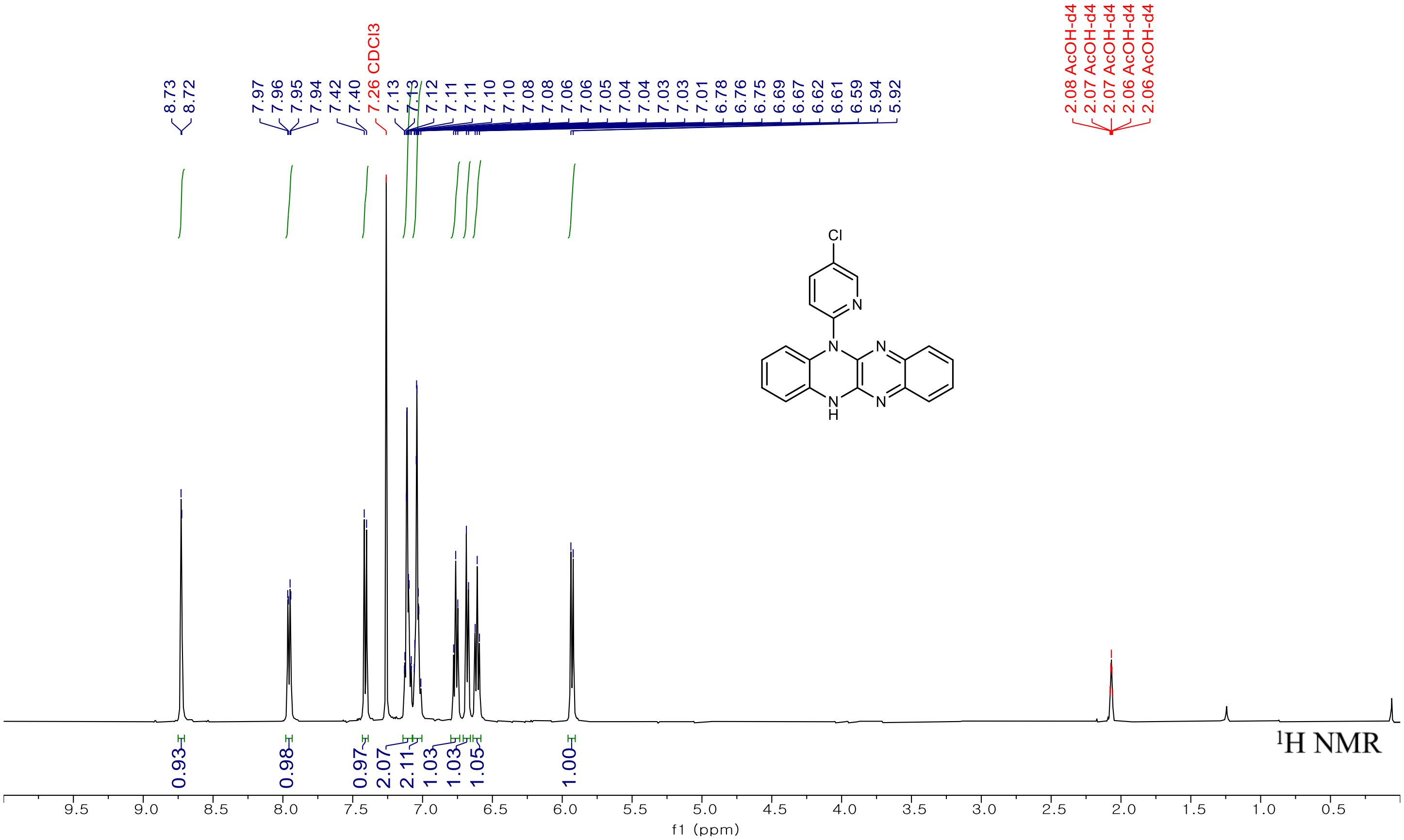


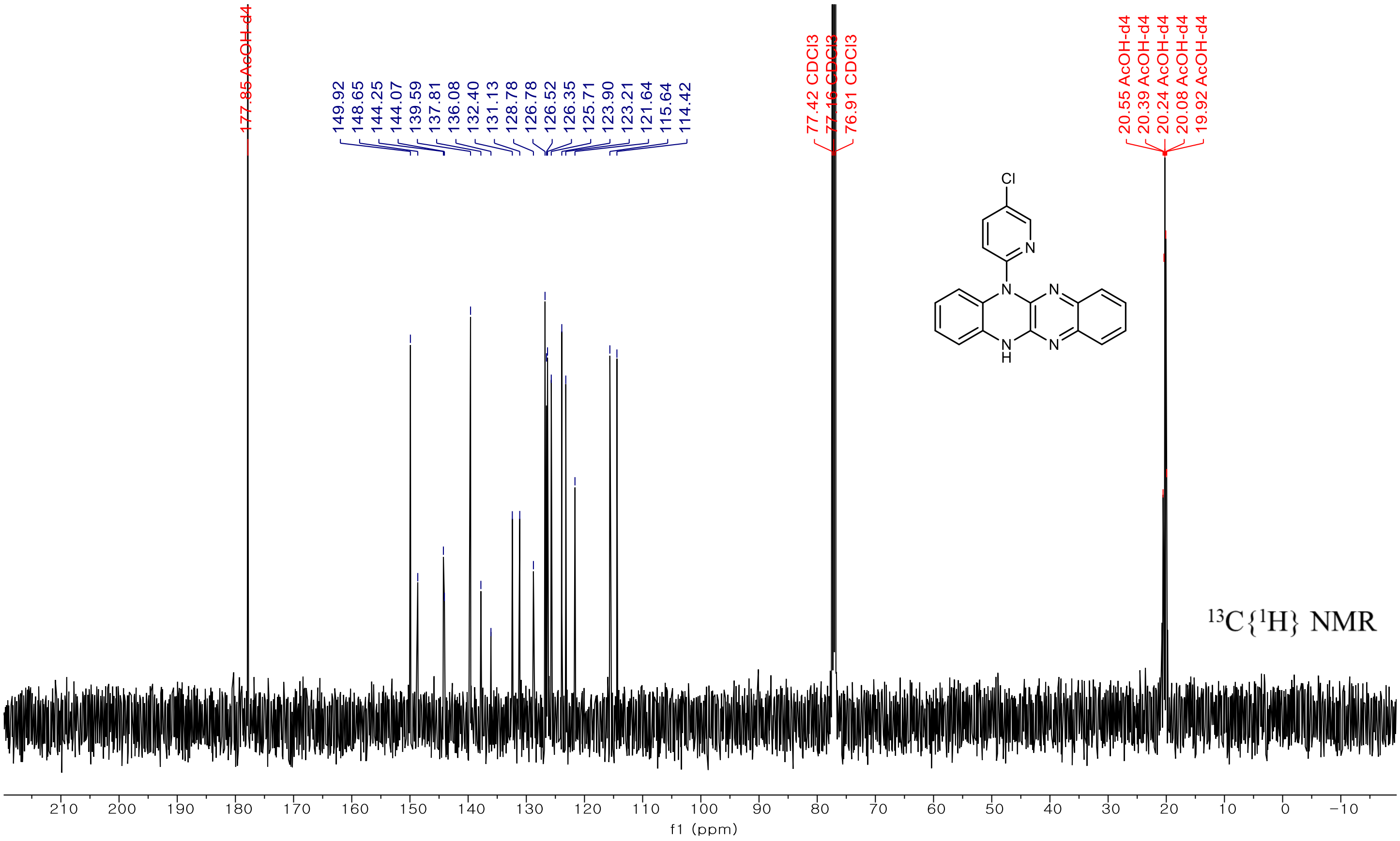

5-[5-(trifluoromethyl)pyridin-2-yl]-5,12-dihydroquinoxalino[2,3-b]quinoxaline (**QQ-PyCF₃**)

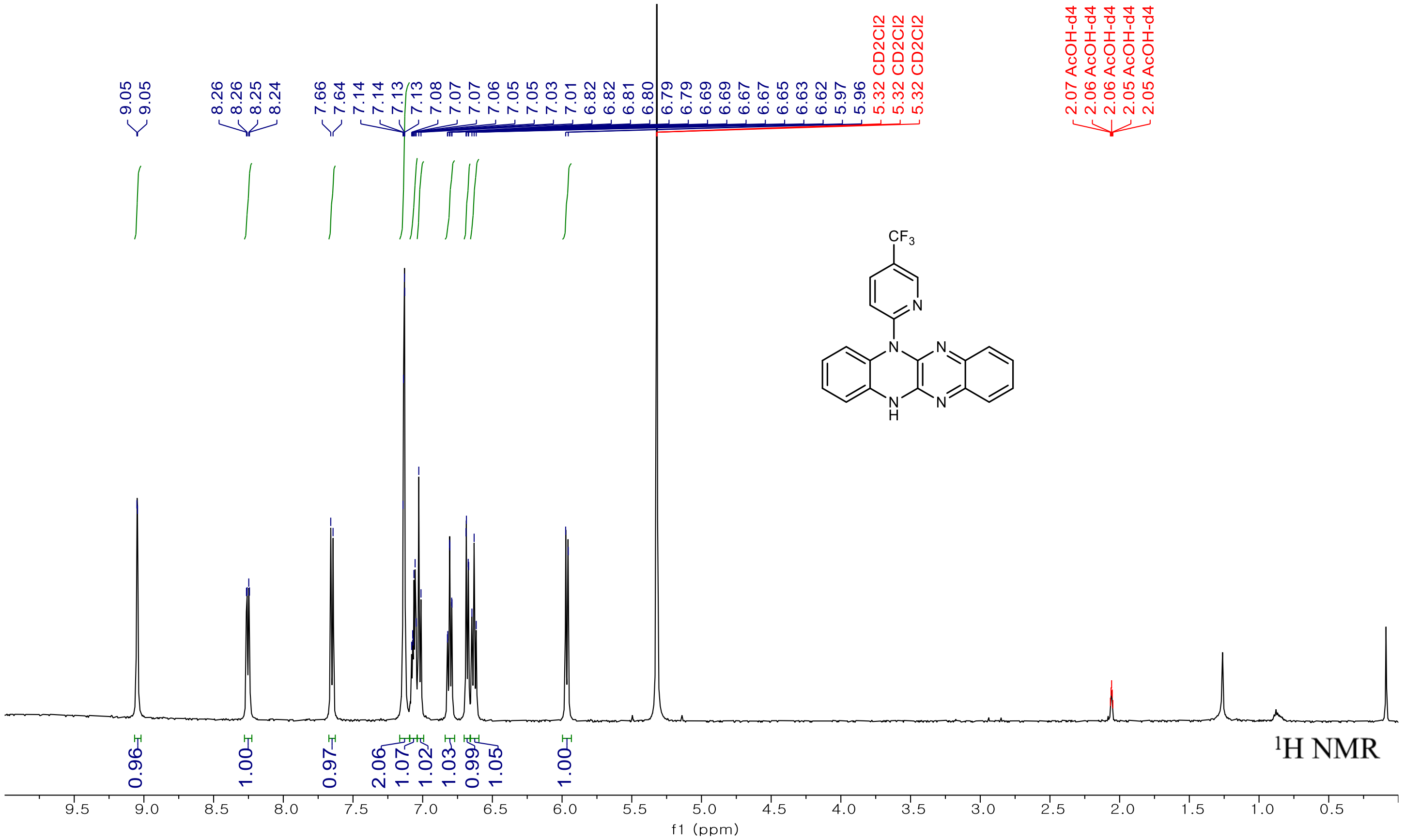


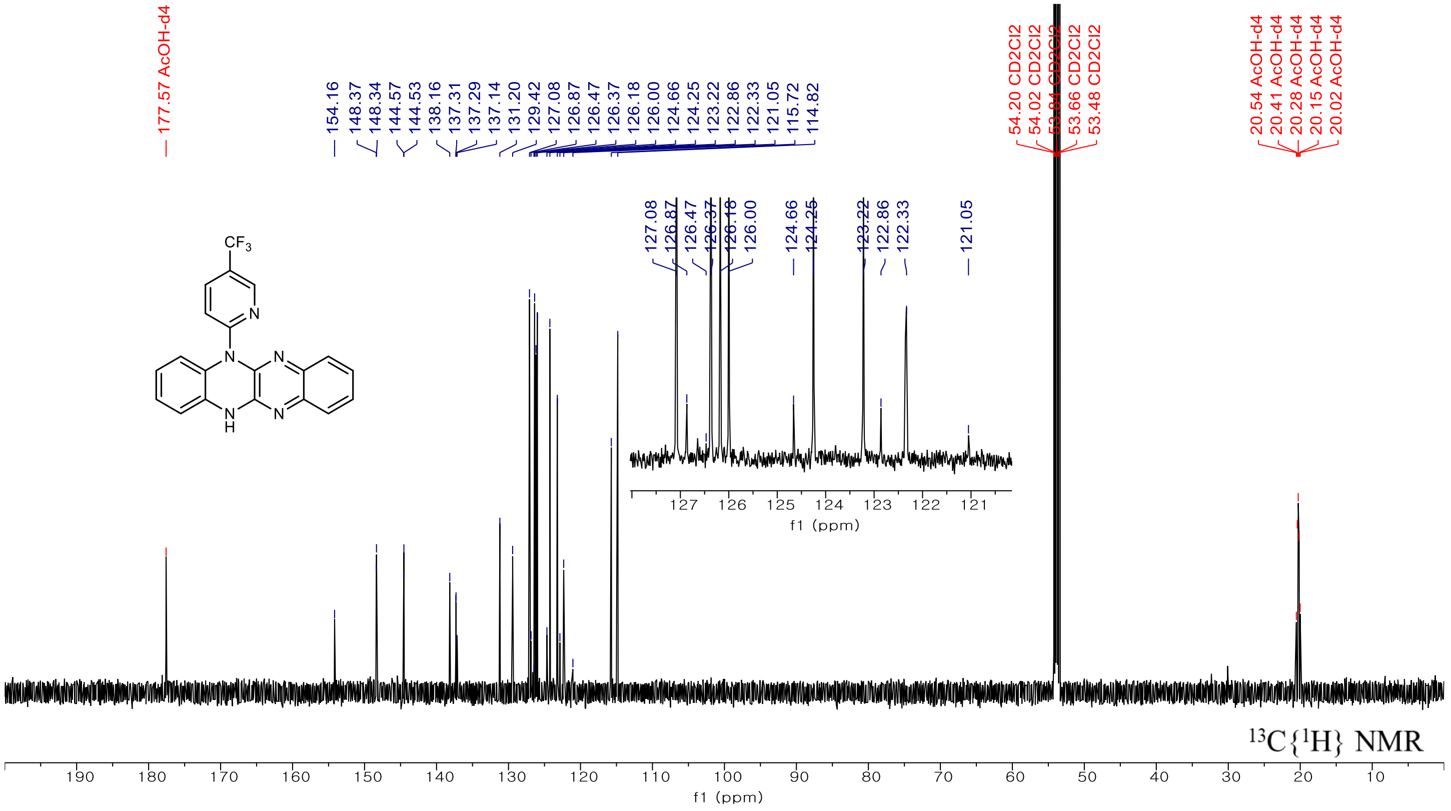

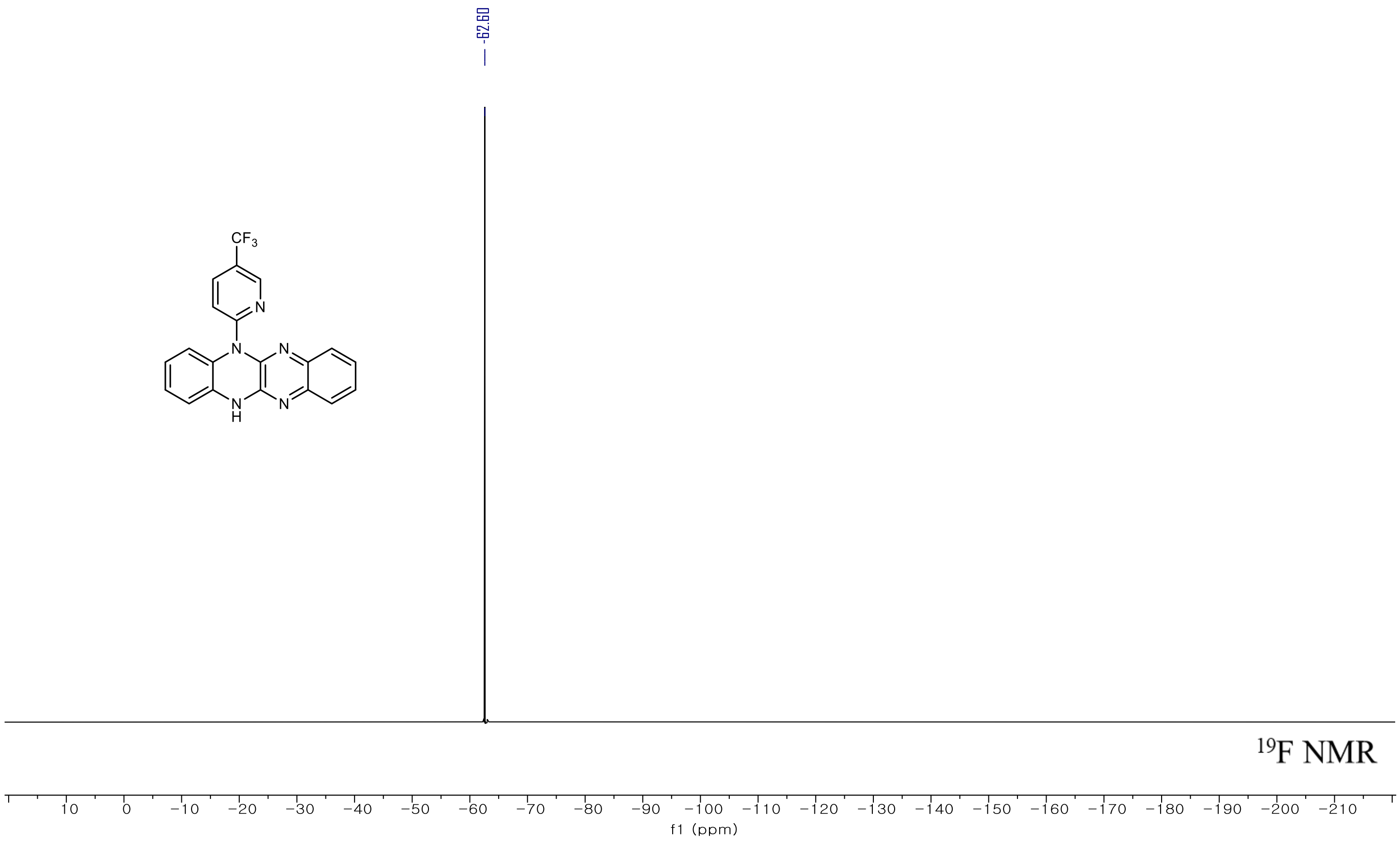
-62.60
CF3
N
N
N
N
H
19F NMR
10 0 −10 −20 −30 −40 −50 −60 −70 −80 −90 −100 −110 −120 −130 −140 −150 −160 −170 −180 −190 −200 −210
f1 (ppm)

4-Methoxybenzonitrile (**4a**)

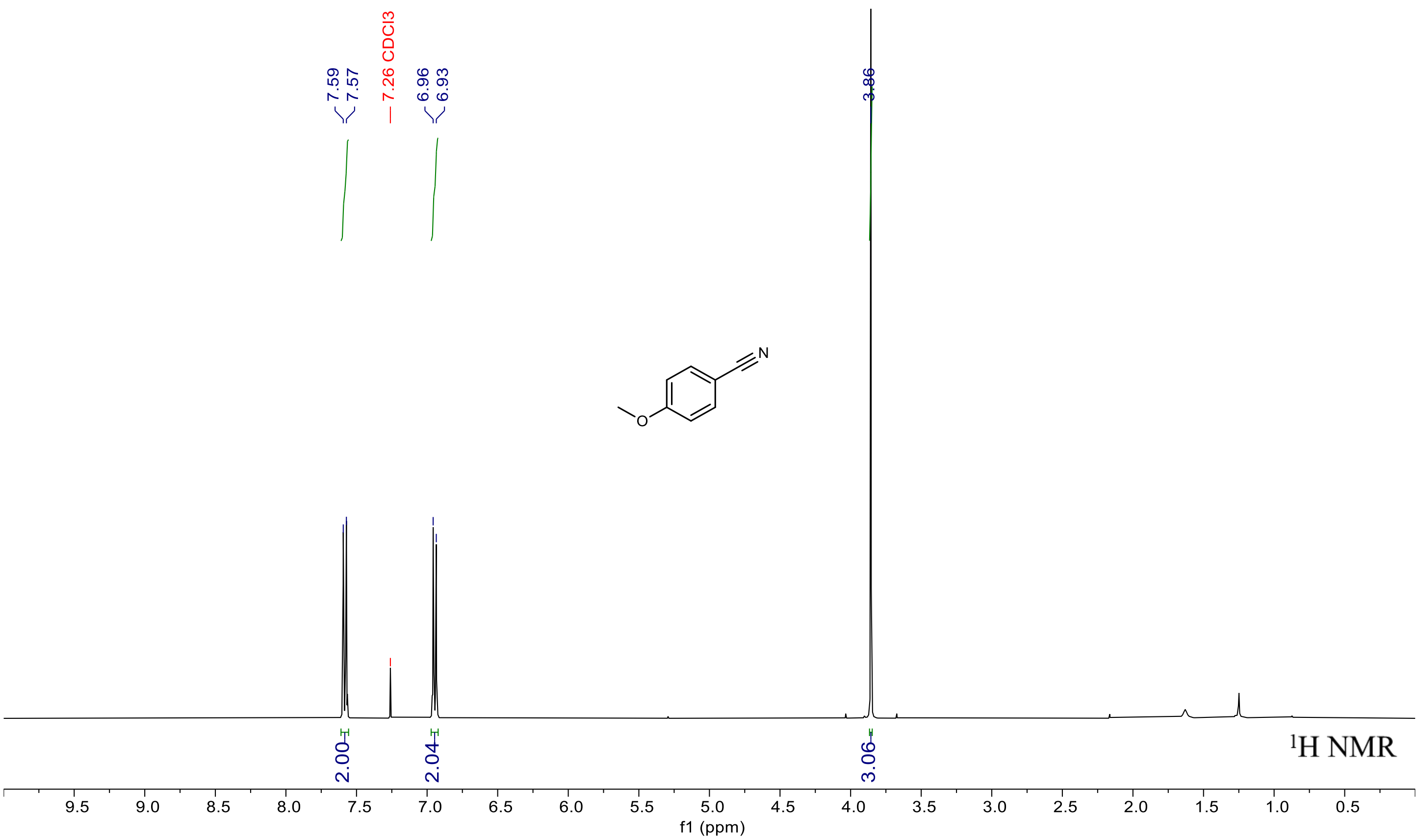


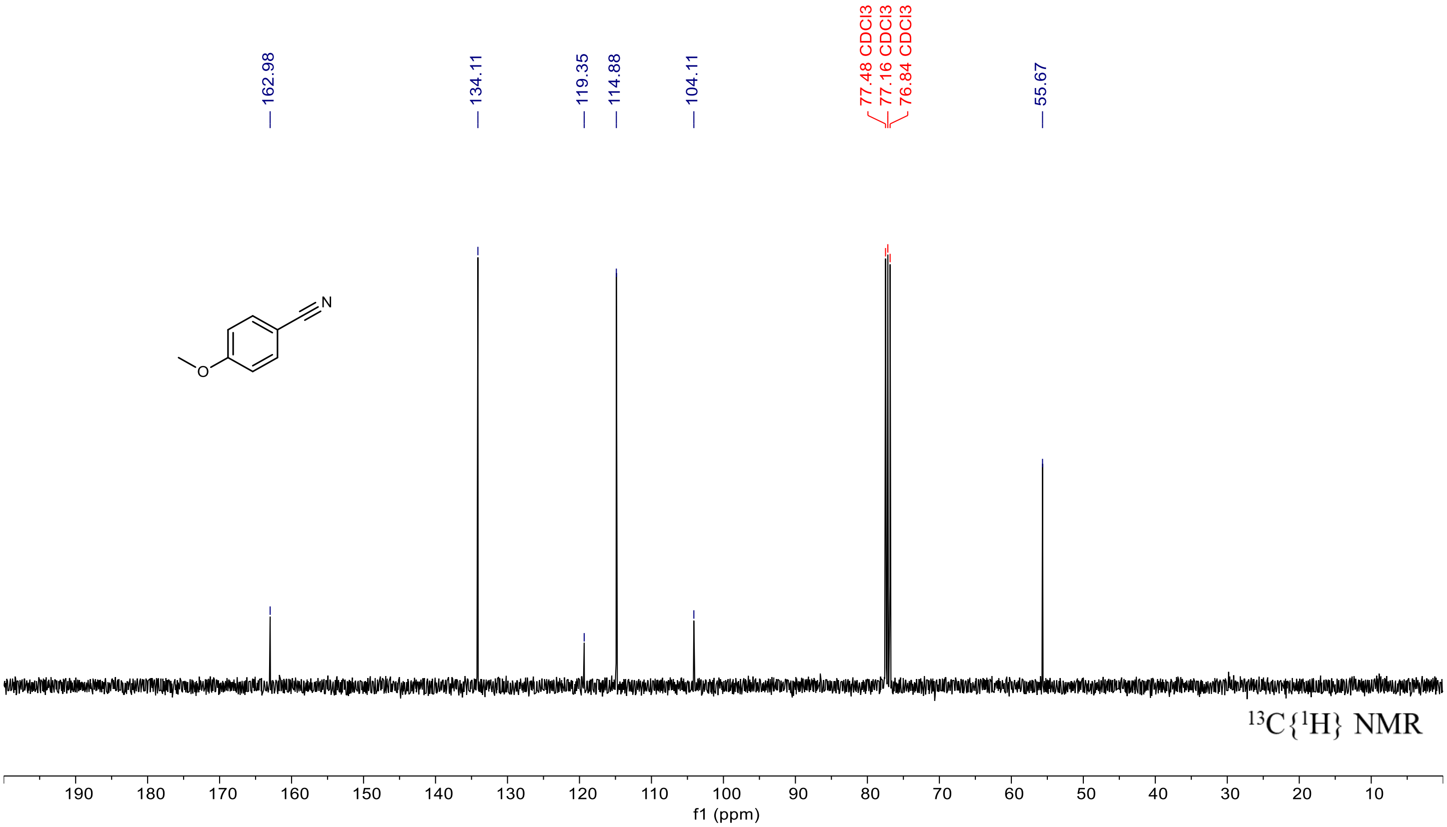

4-Methylbenzonitrile (**4b**)

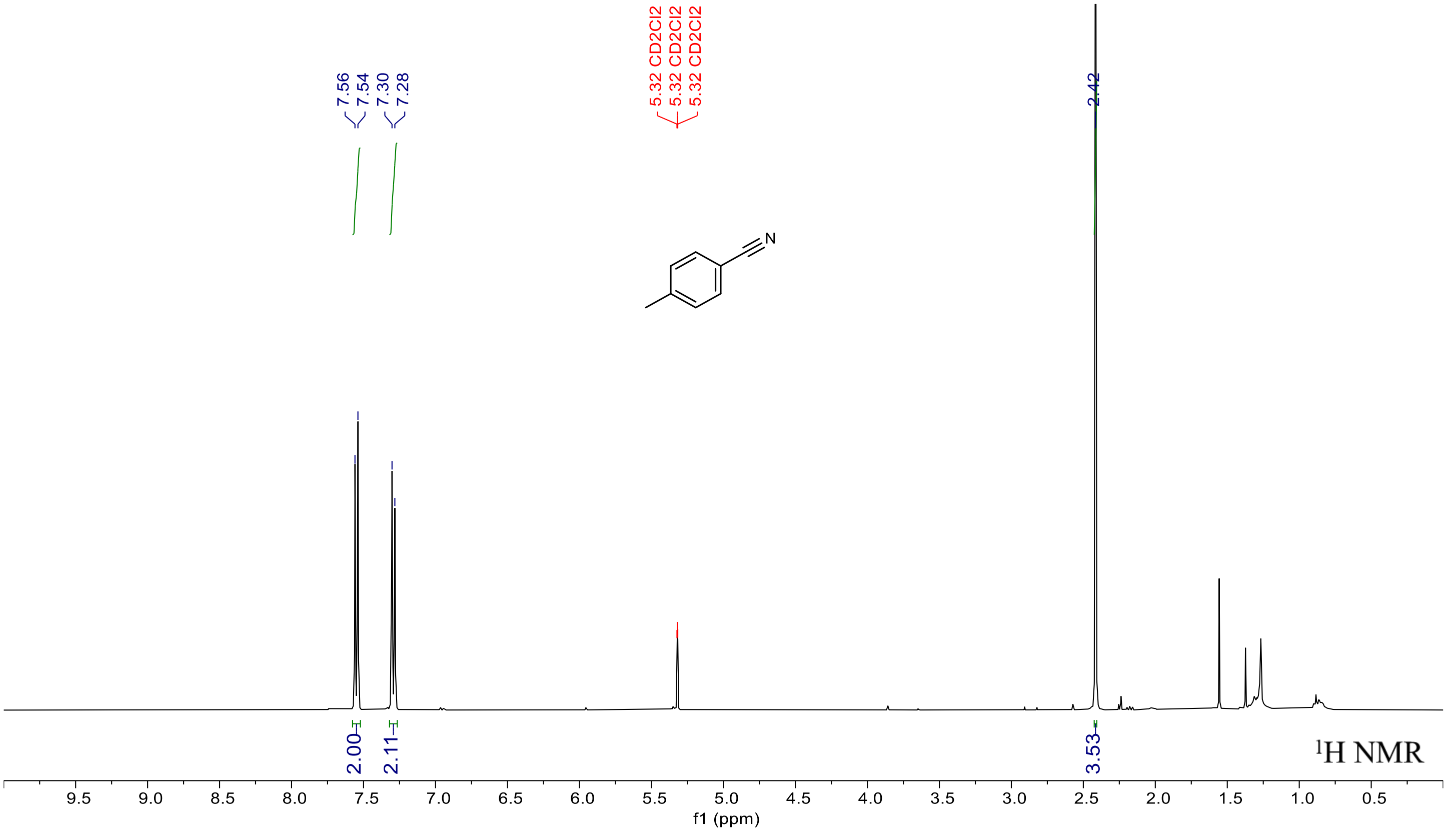


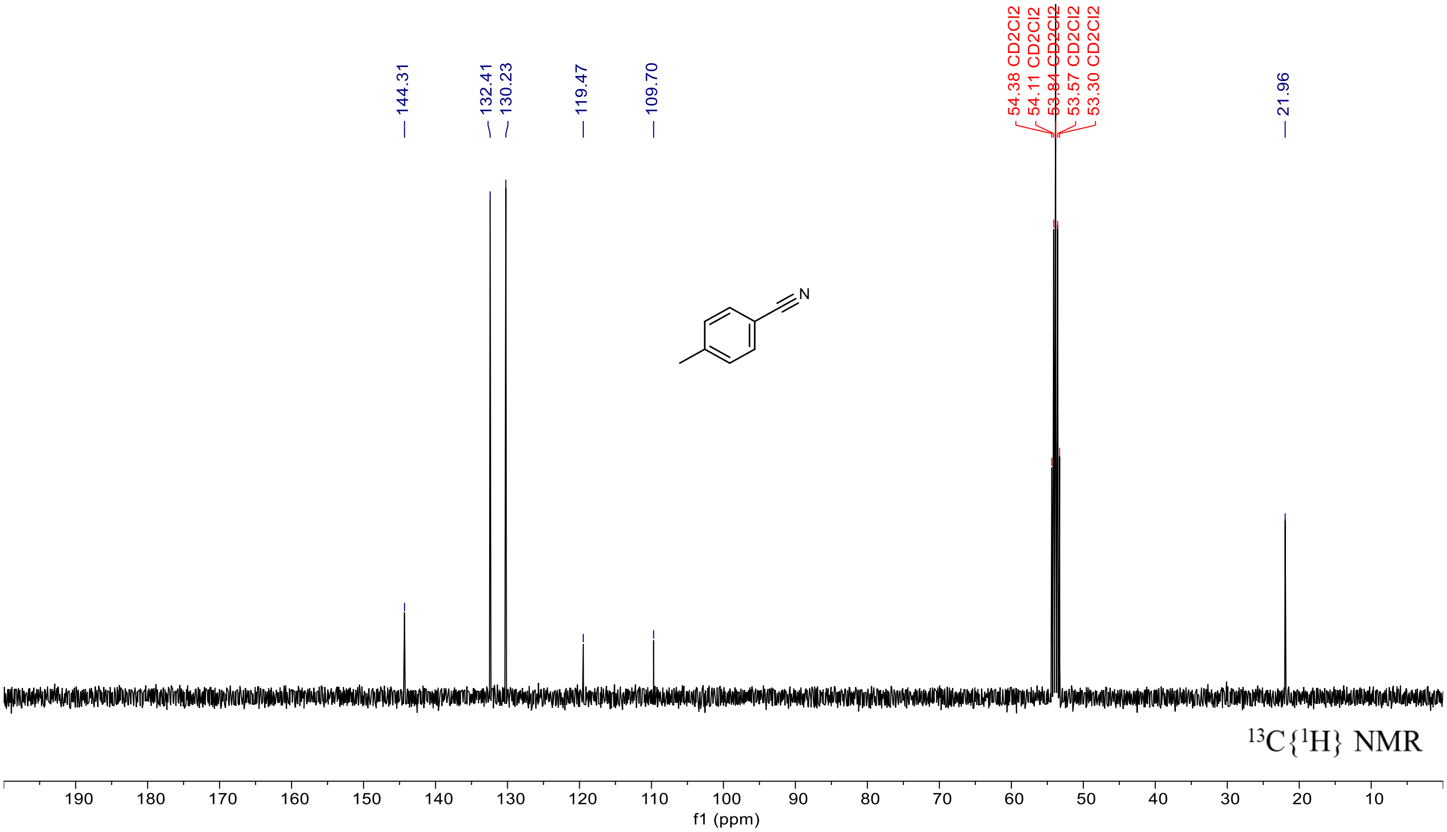

4-Bromobenzonitrile (**4c**)

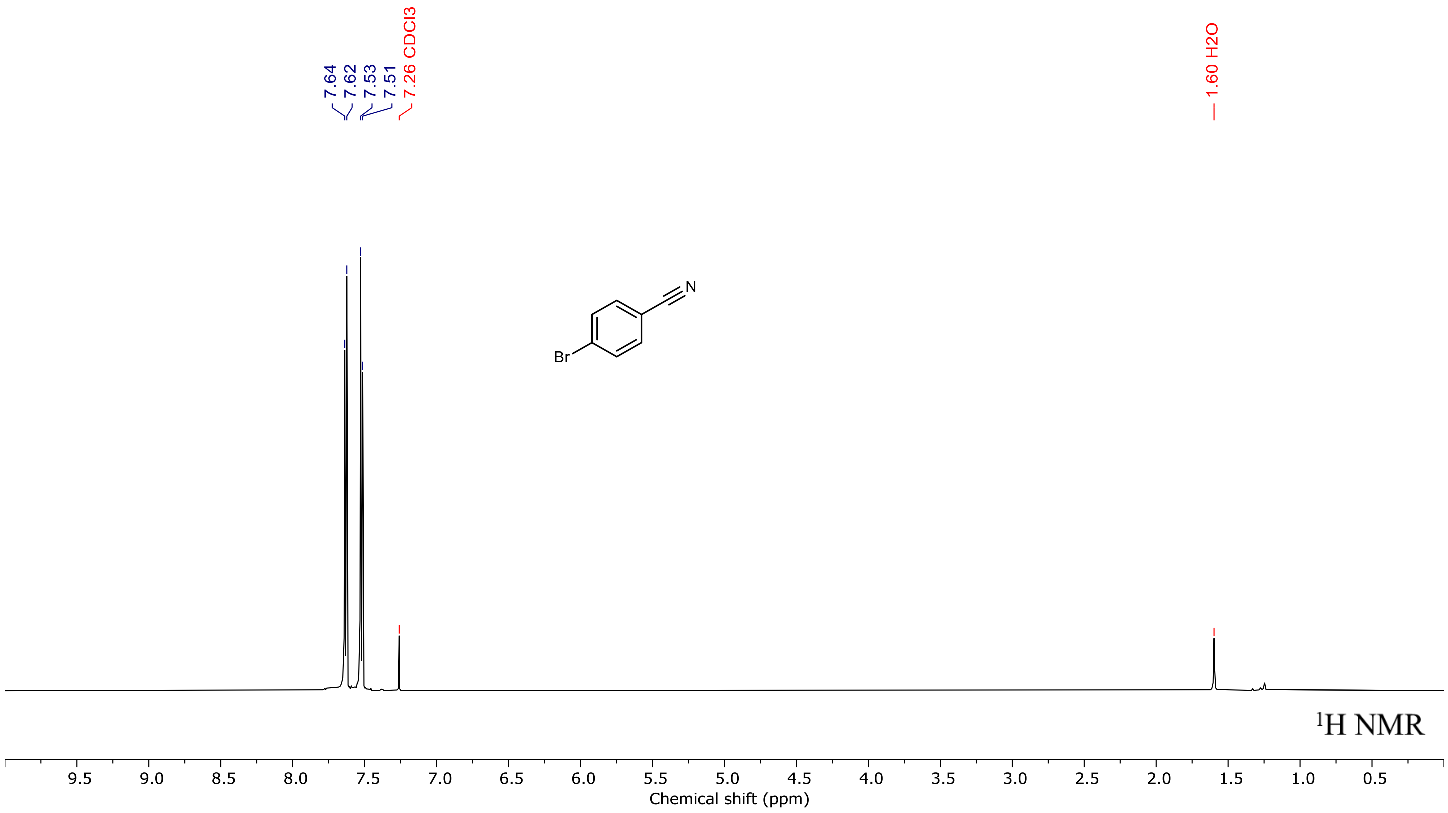


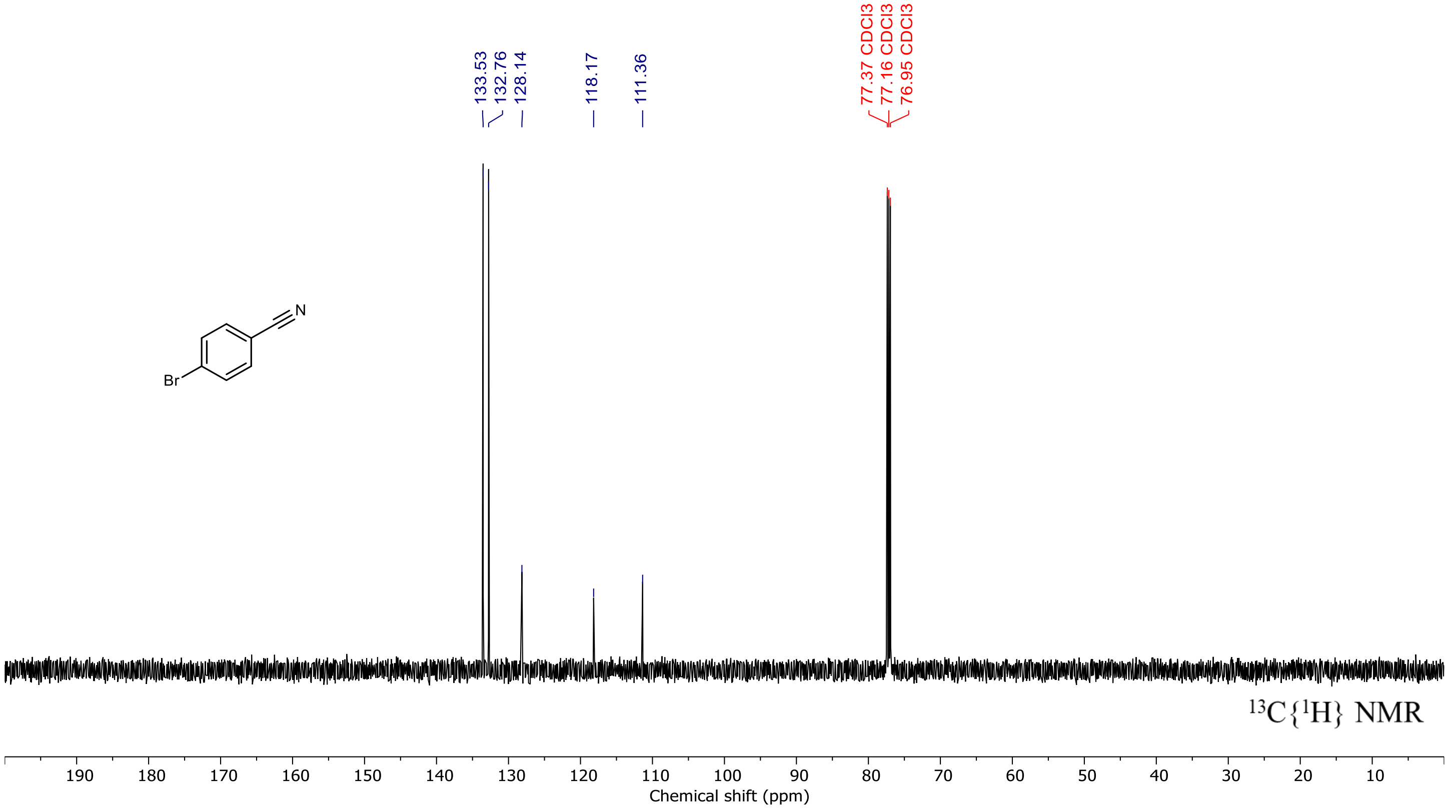

4-Iodobenzonitrile (**4d**)

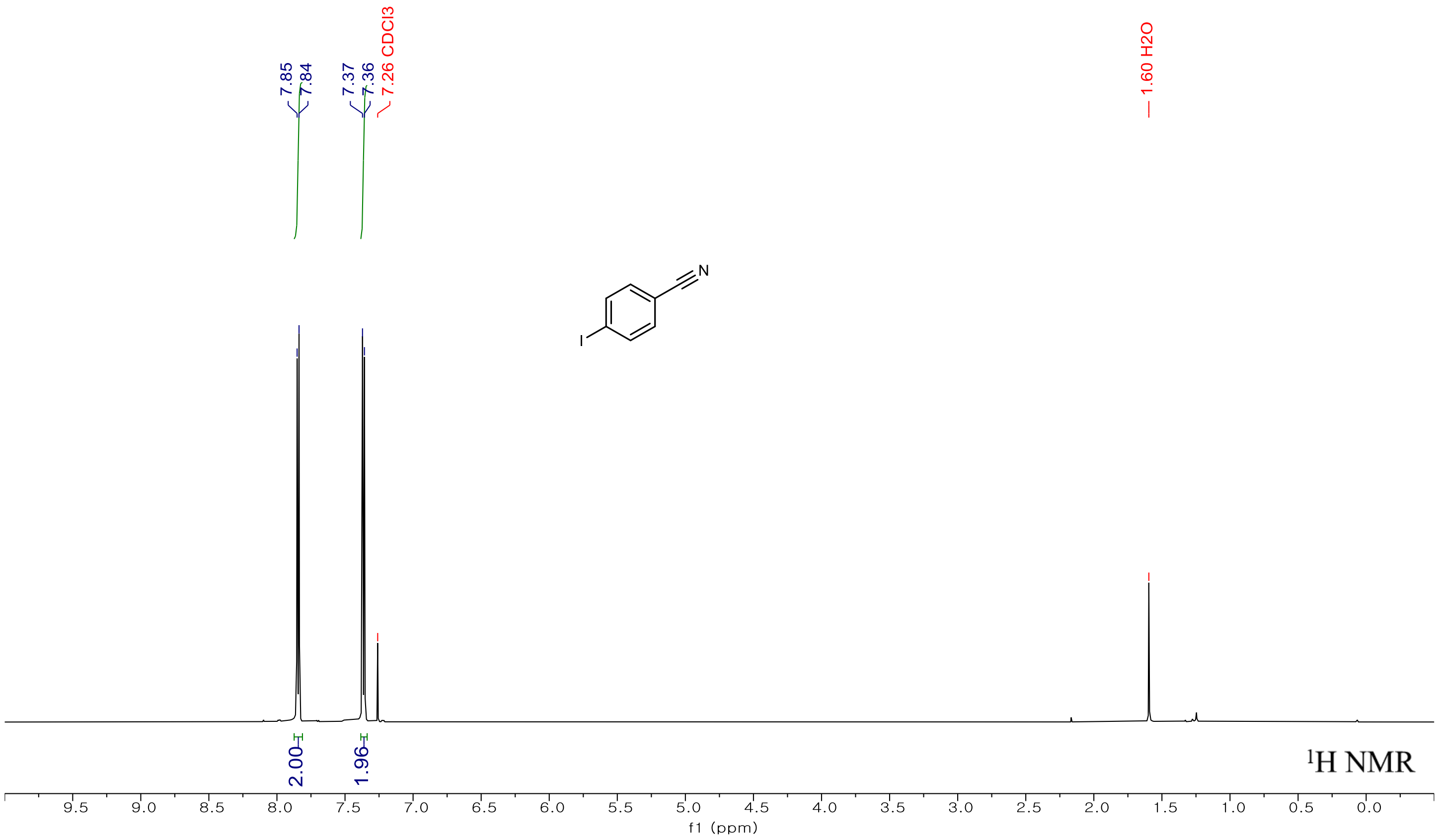


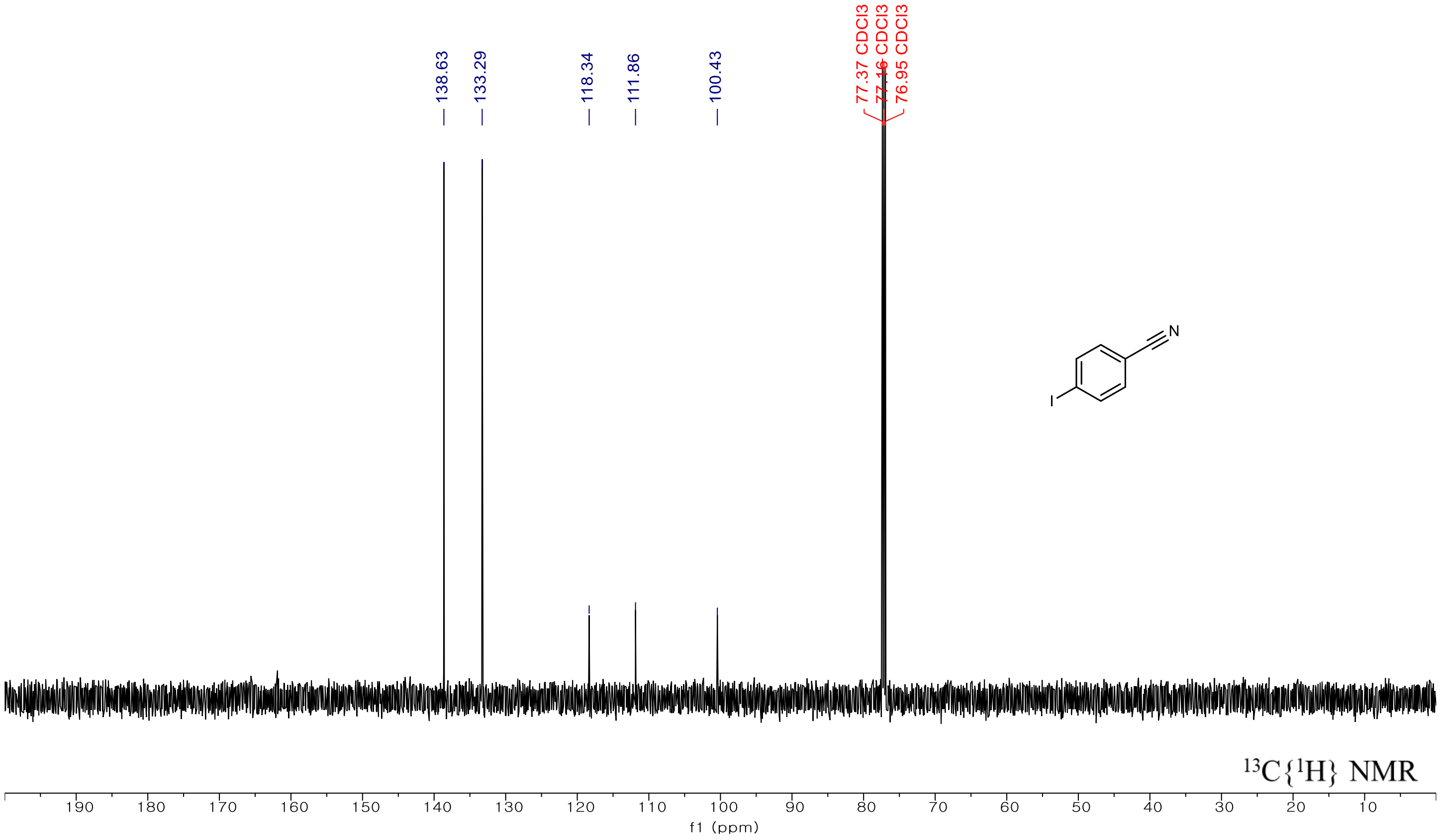

4-(Methylsulfonyl)benzonitrile (**4e**)

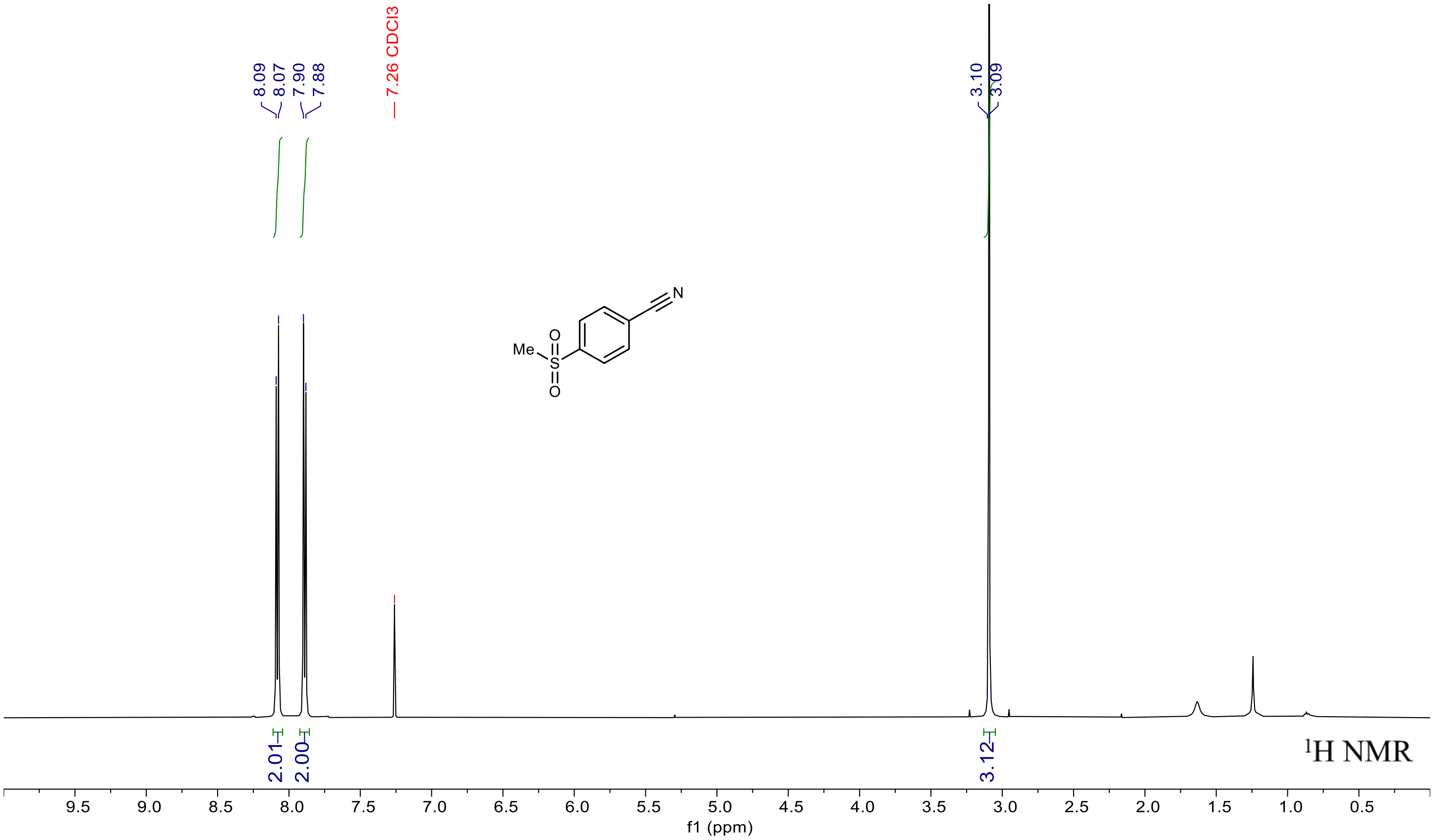


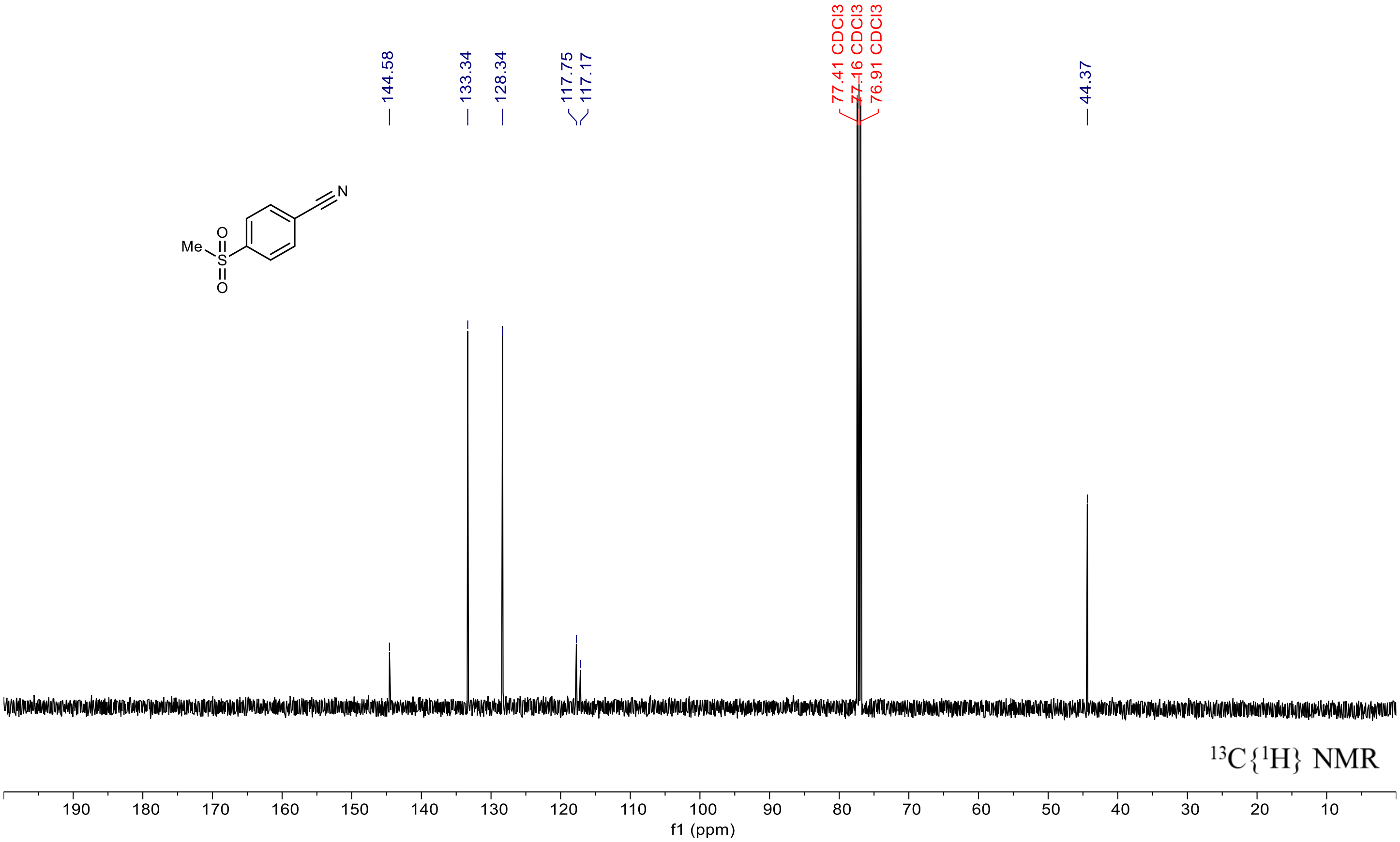

4-Hydroxybenzonitrile (**4f**)

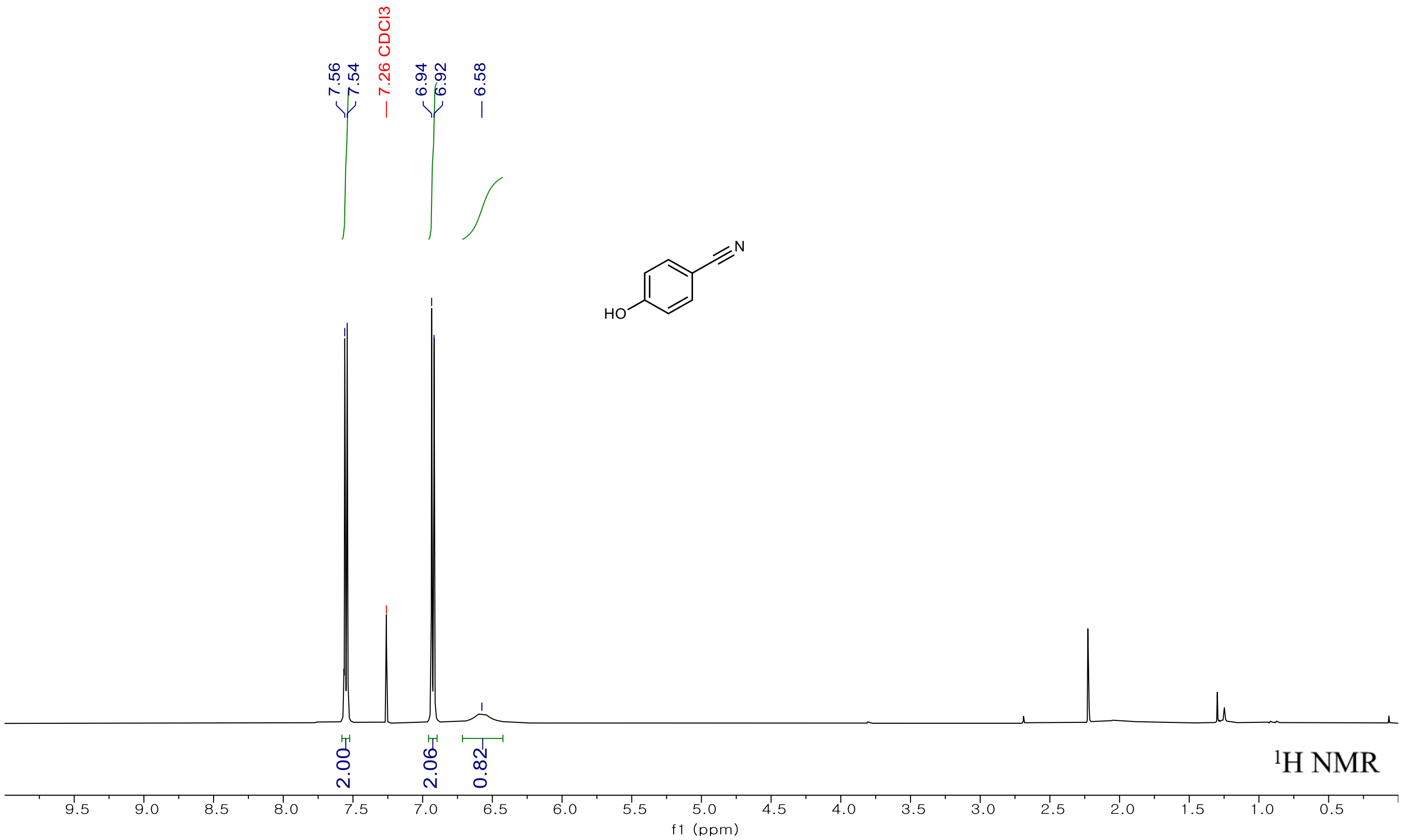


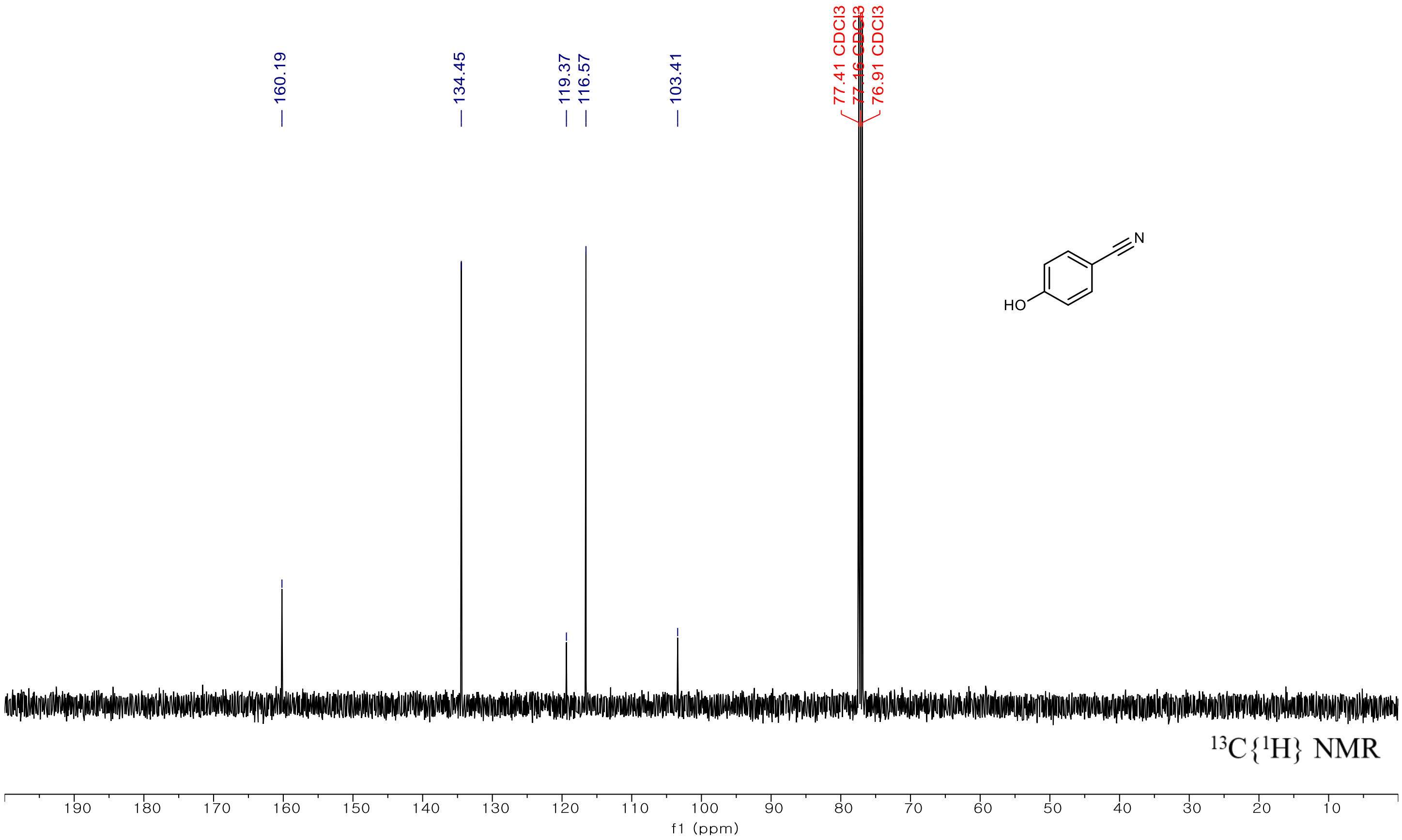

4-Nitrobenzonitrile (**4g**)

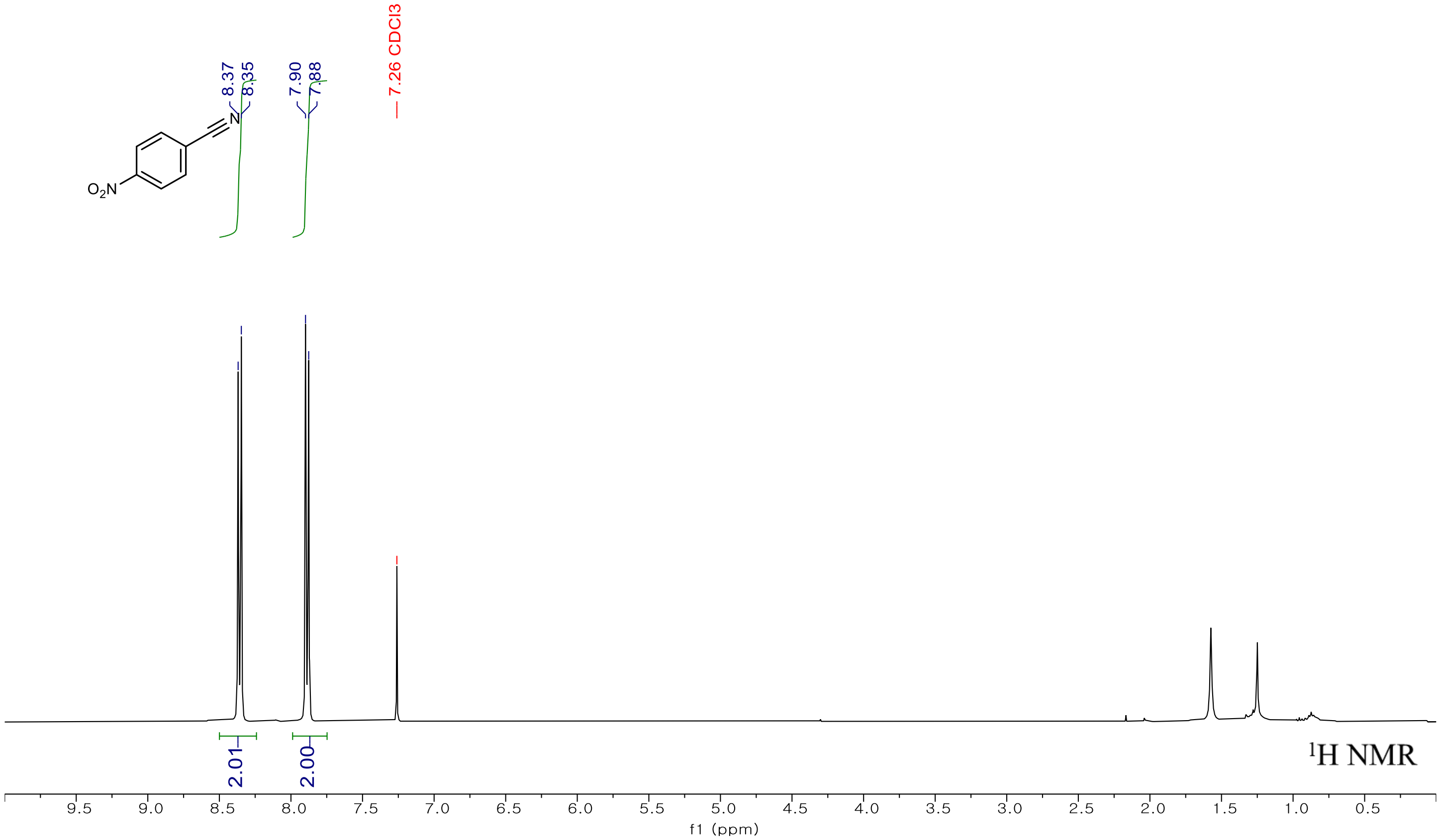


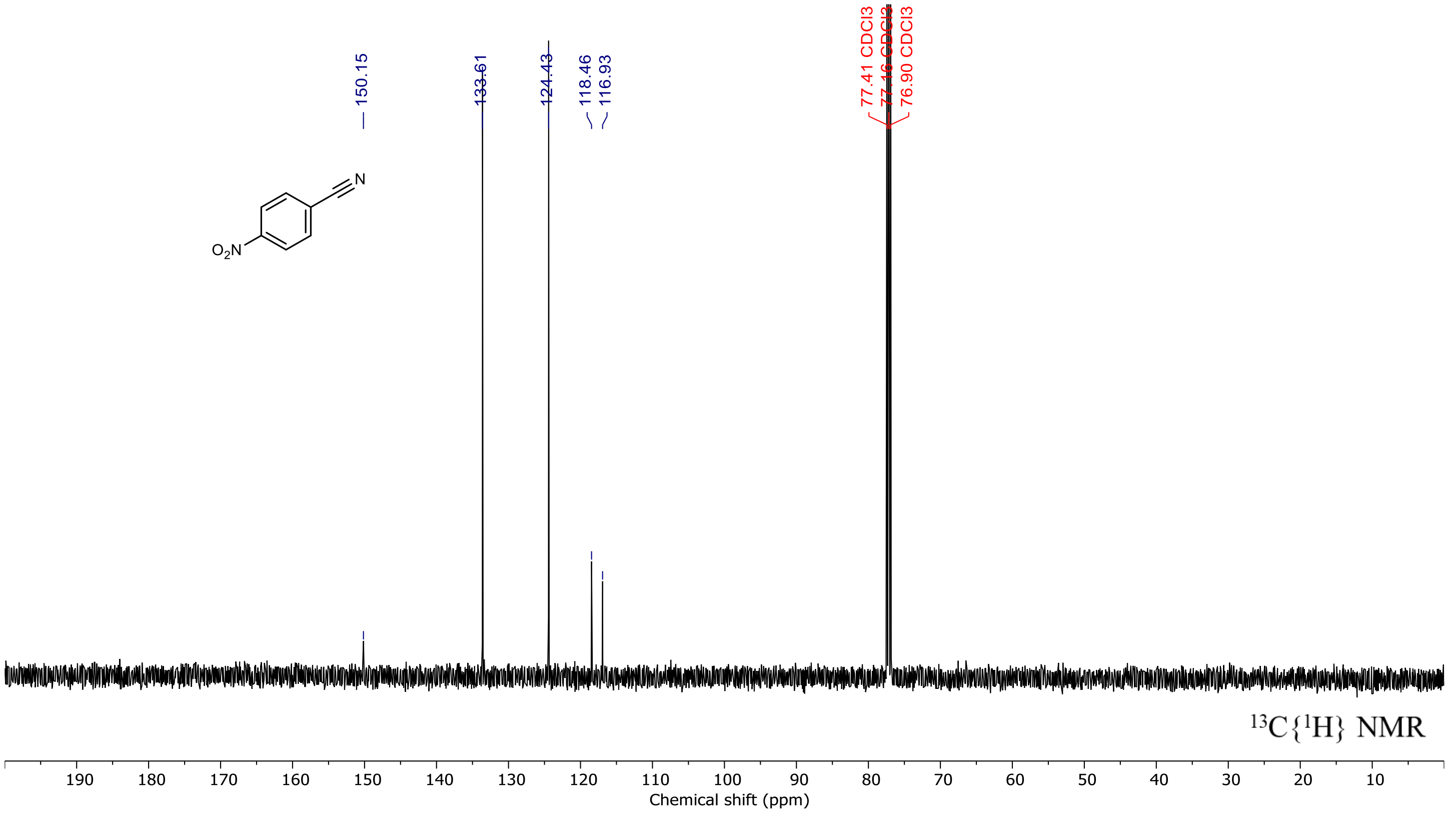

9*H*-Fluorene-1-carbonitrile (**4h**)

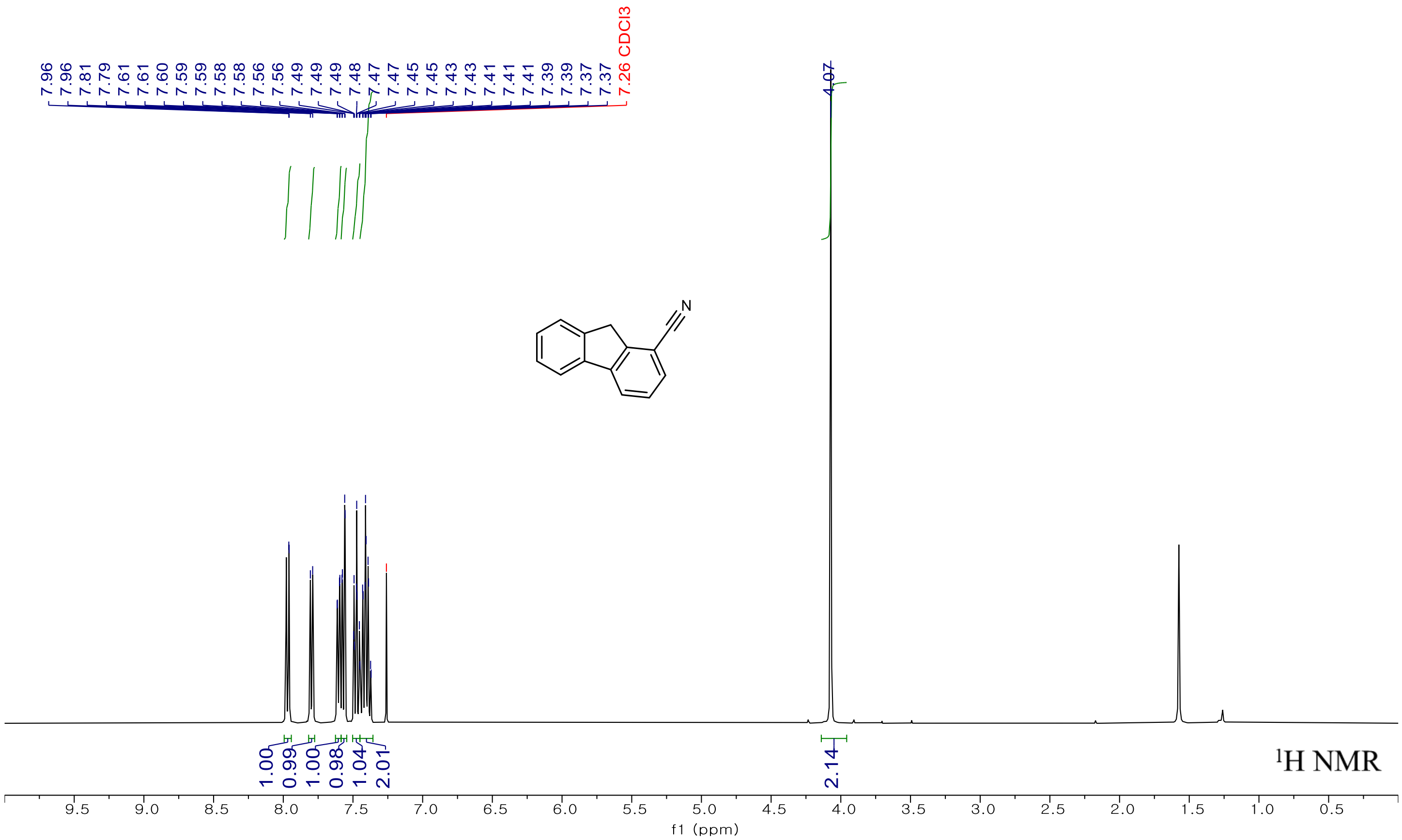


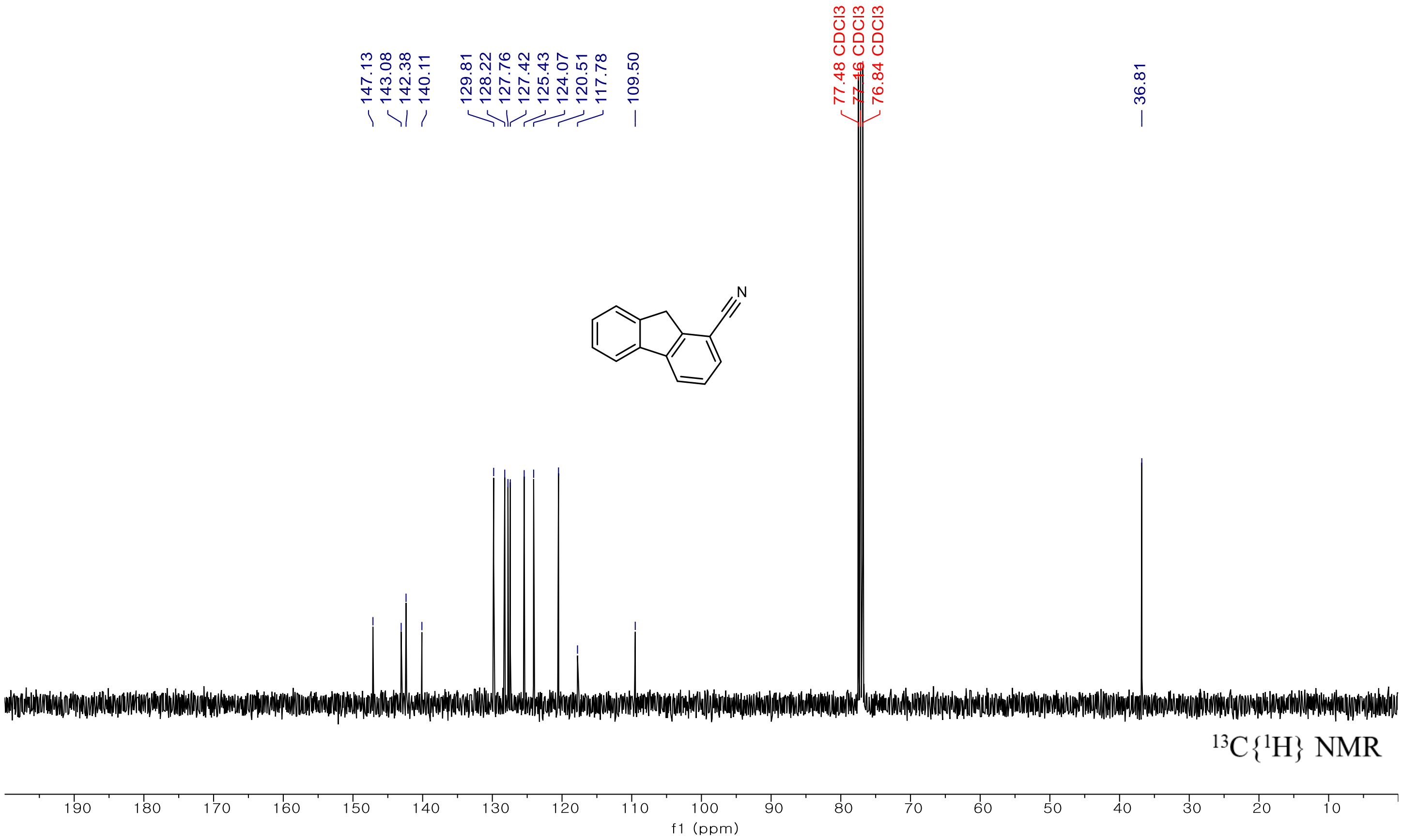

3-(Pyridin-2-yl)benzonitrile (**4i**)

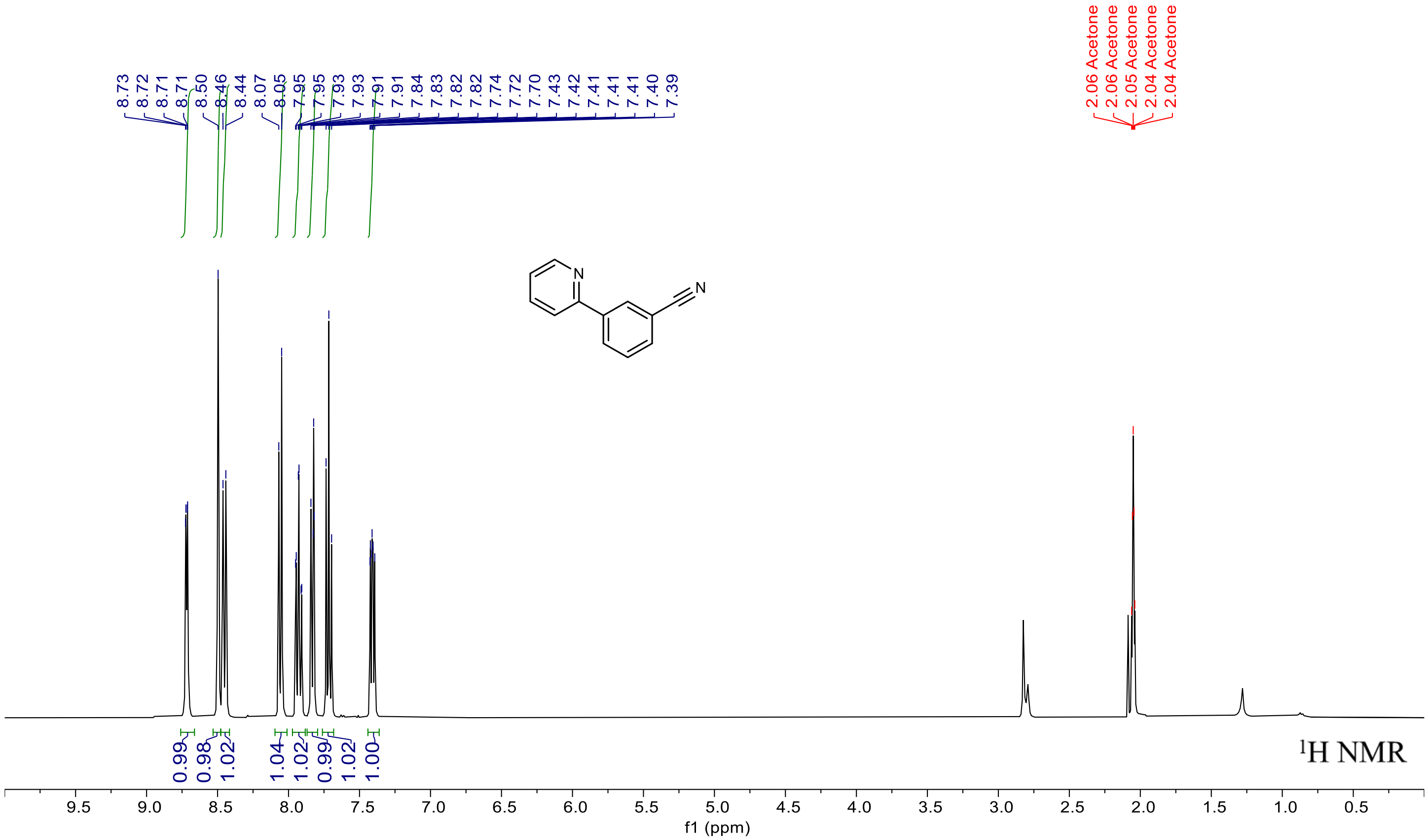


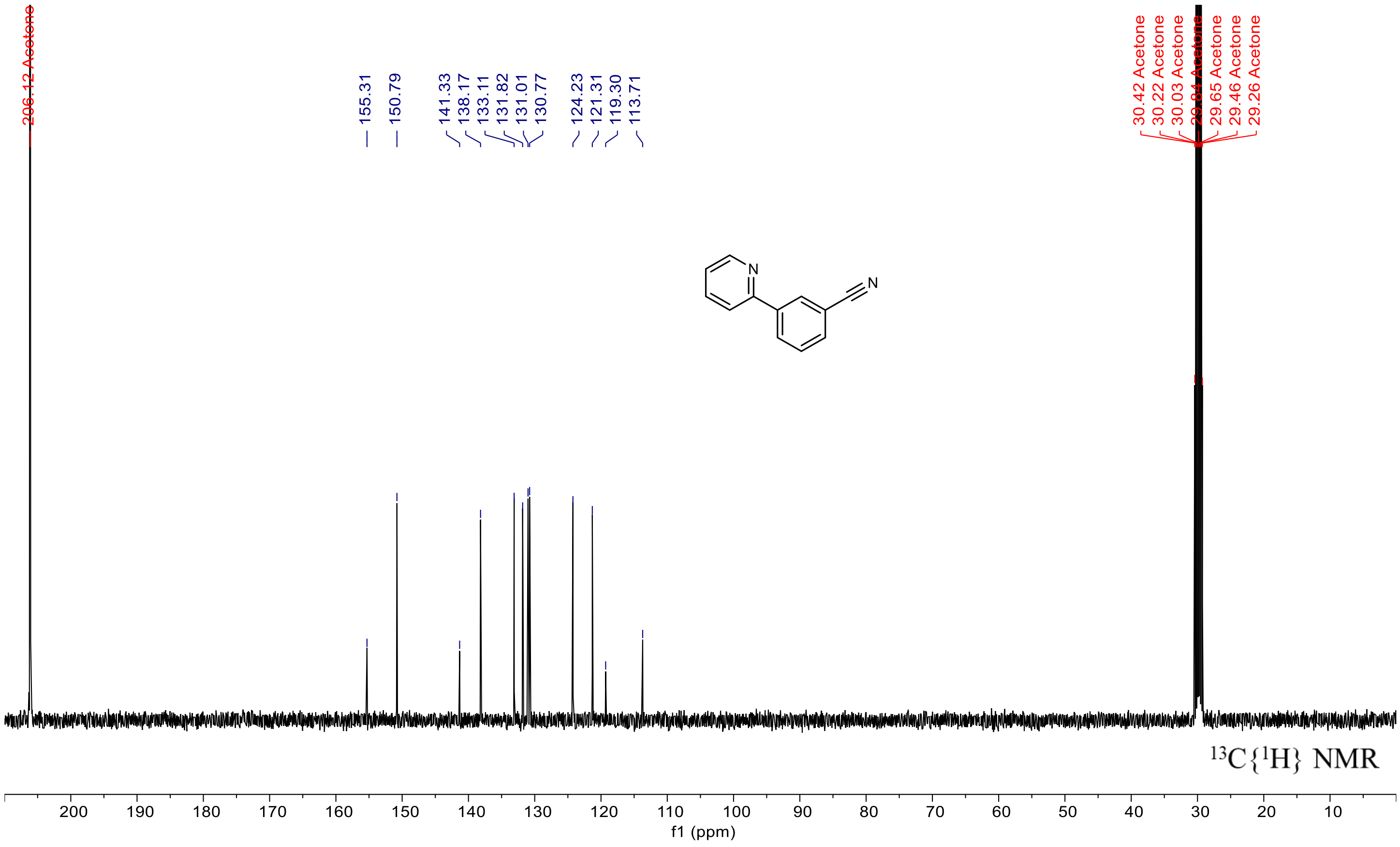

Picolinonitrile (**4j**)

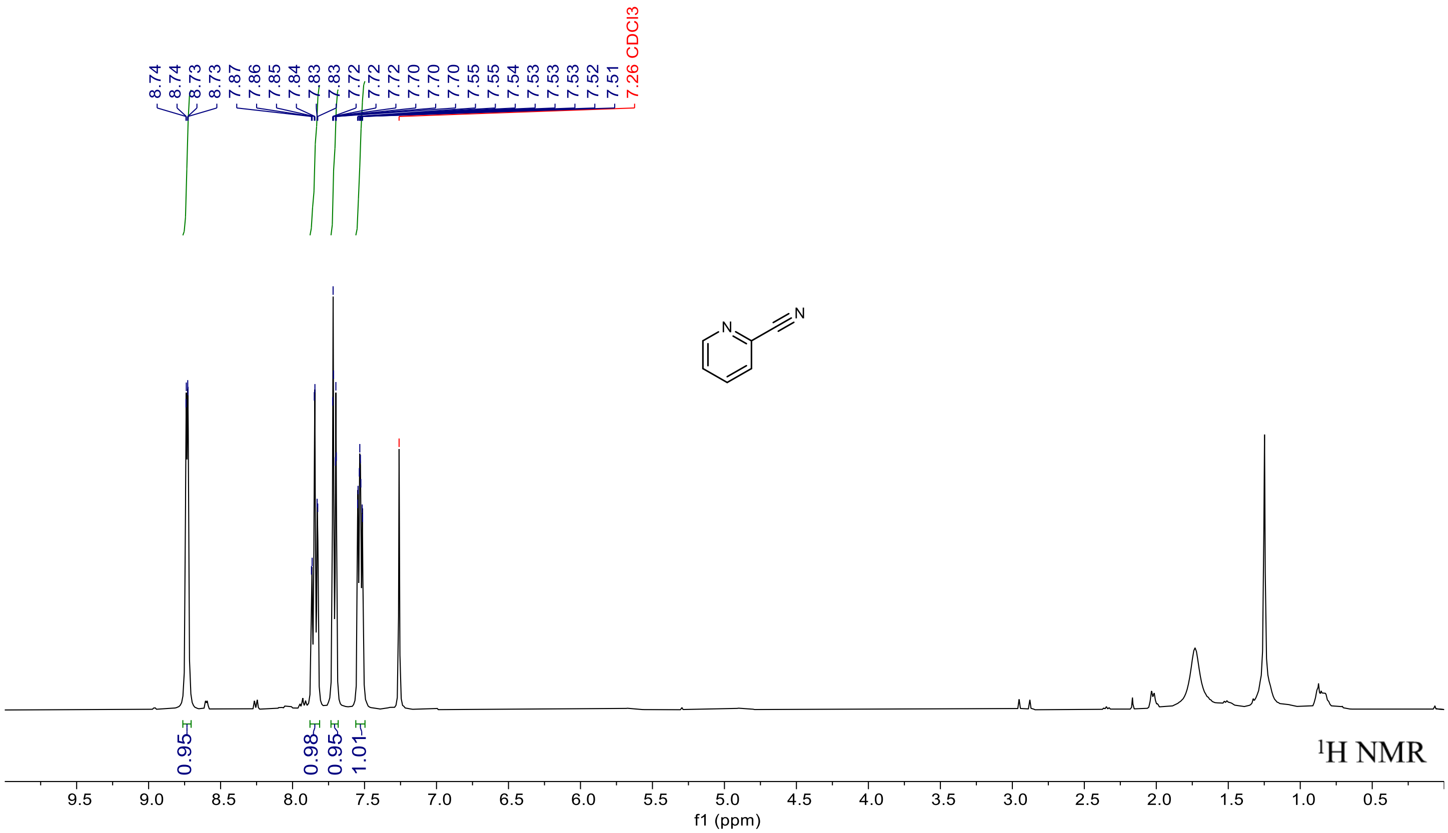


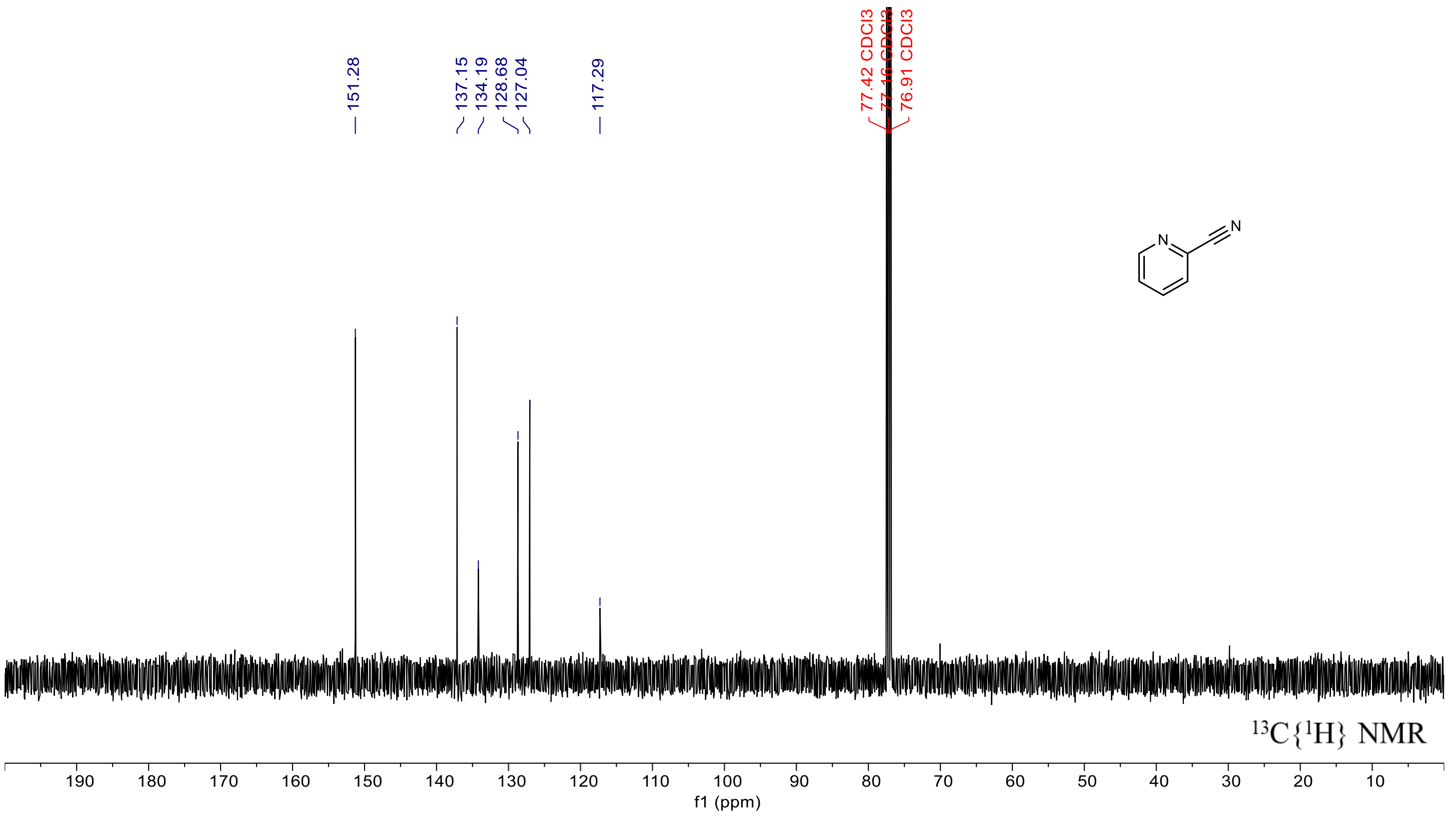

2-Chloronicotinonitrile (**4k**)

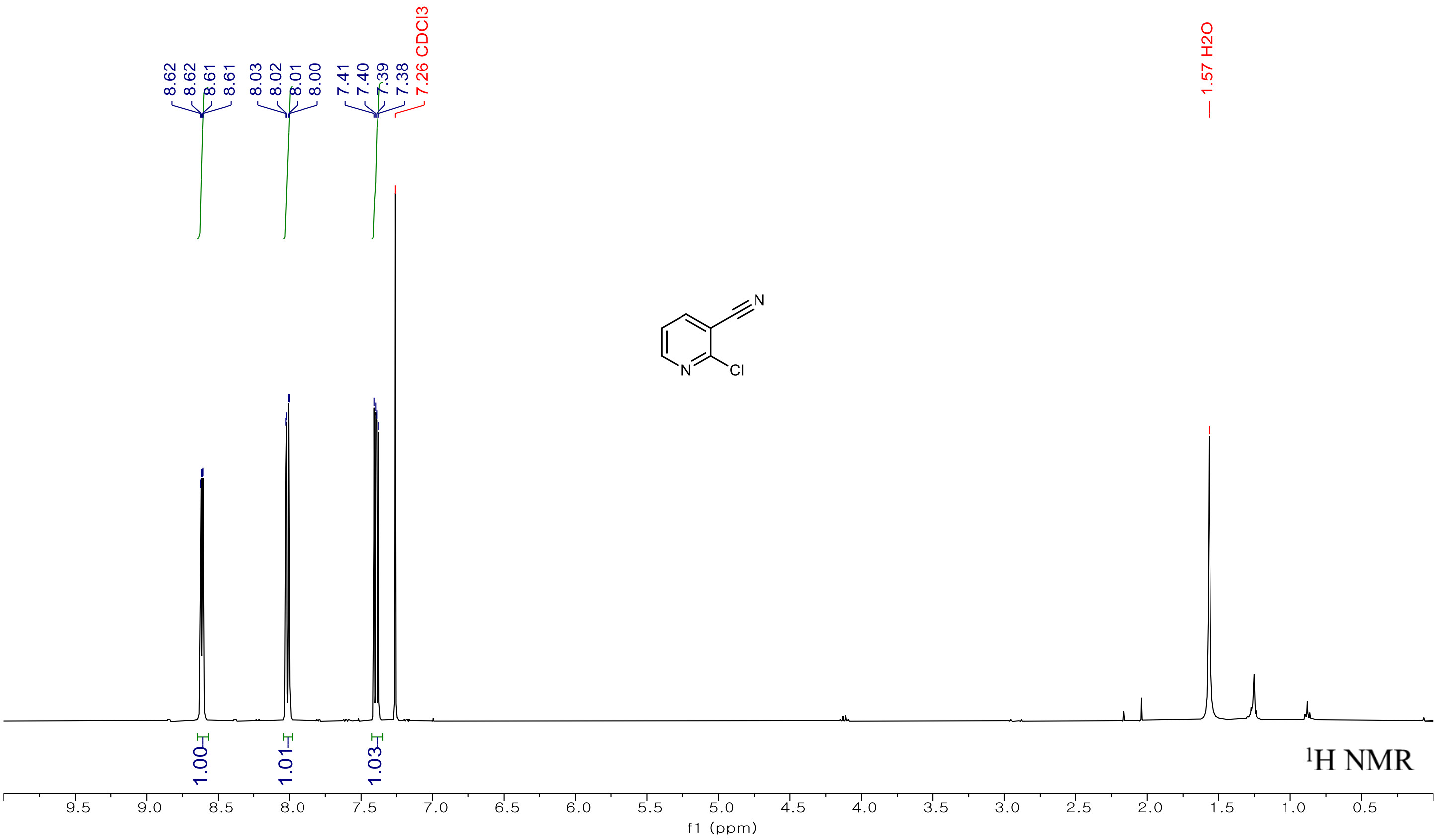


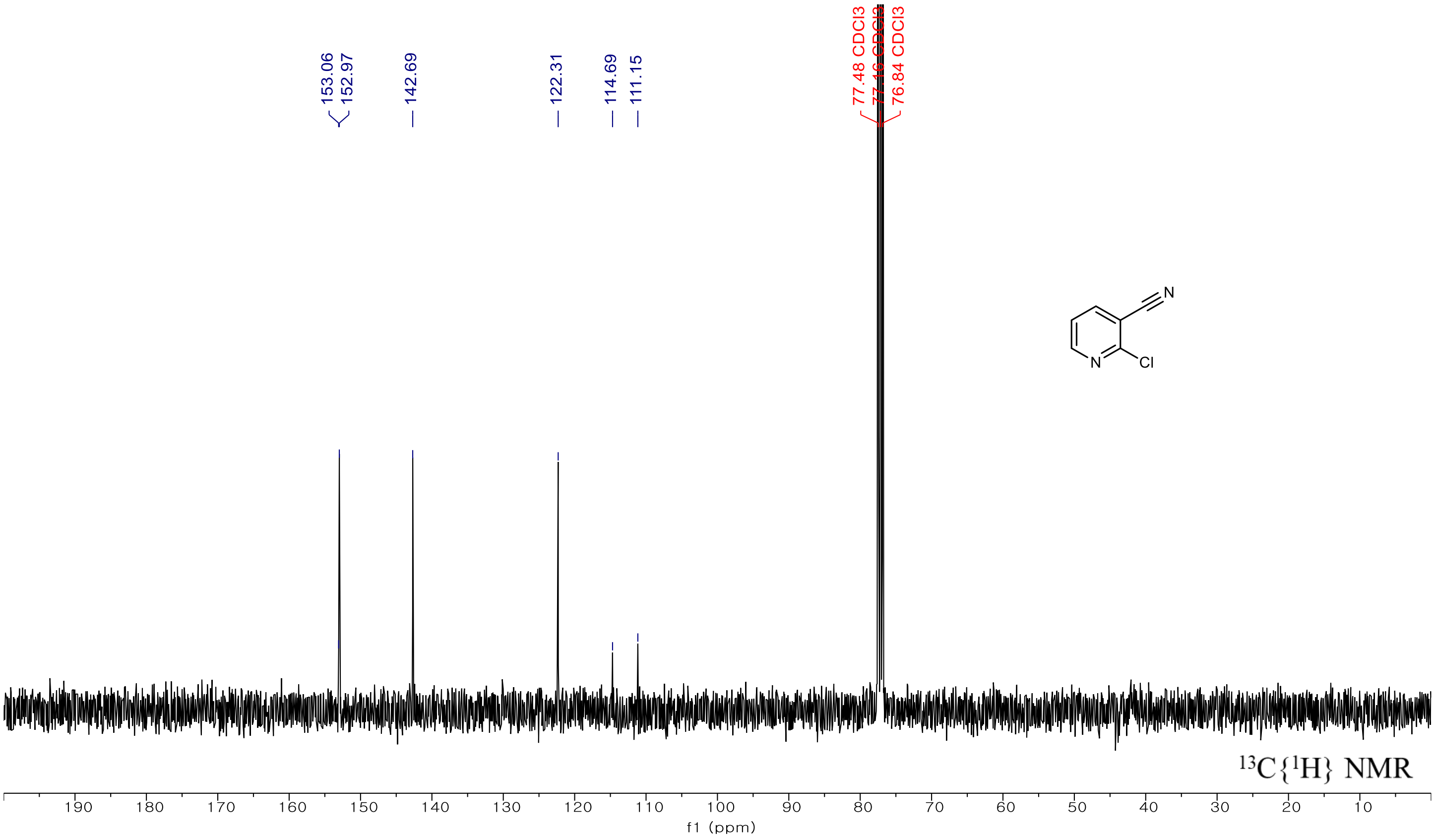

Quinoline-6-carbonitrile (**4l**)

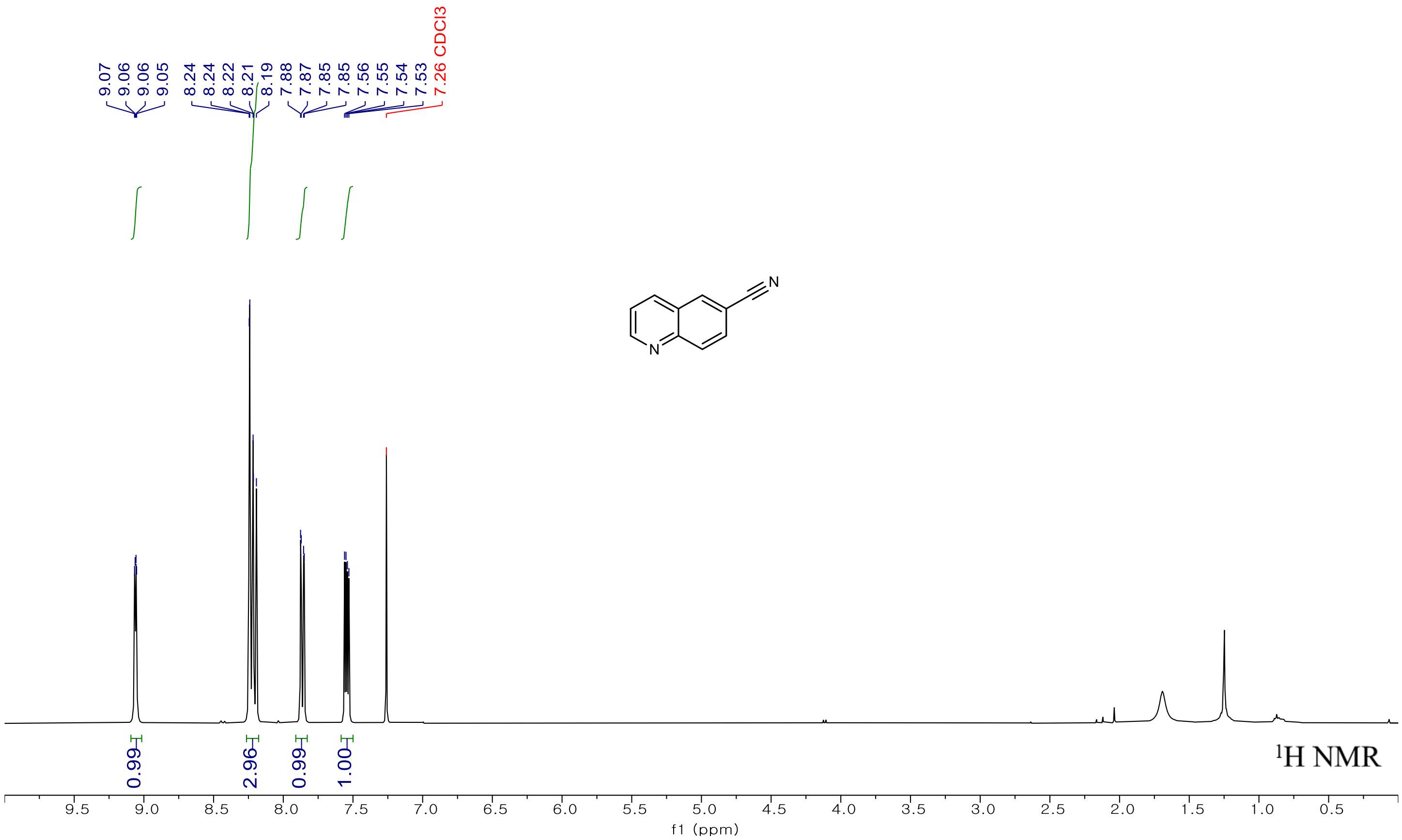


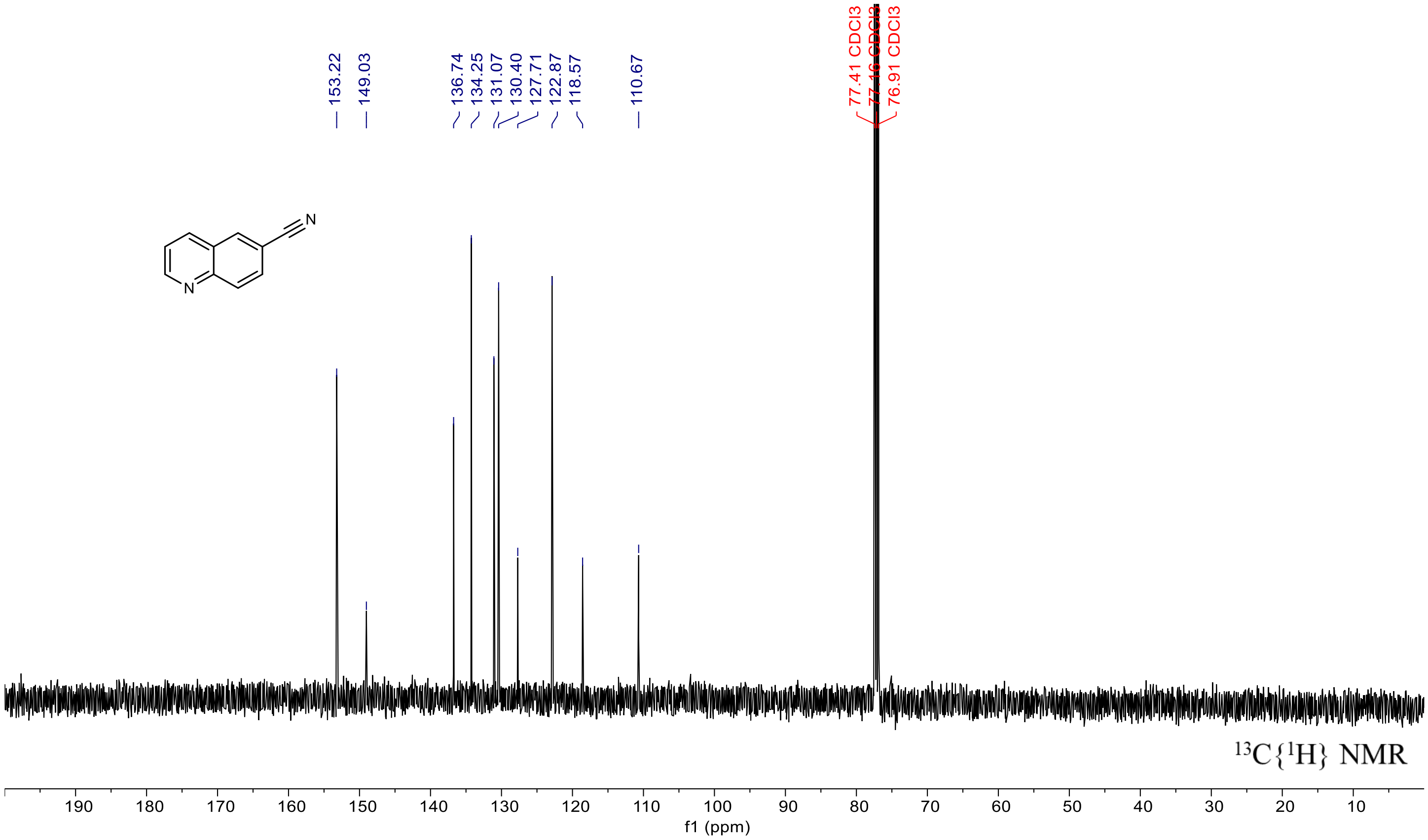

1*H*-Indole-3-carbonitrile (**4m**)

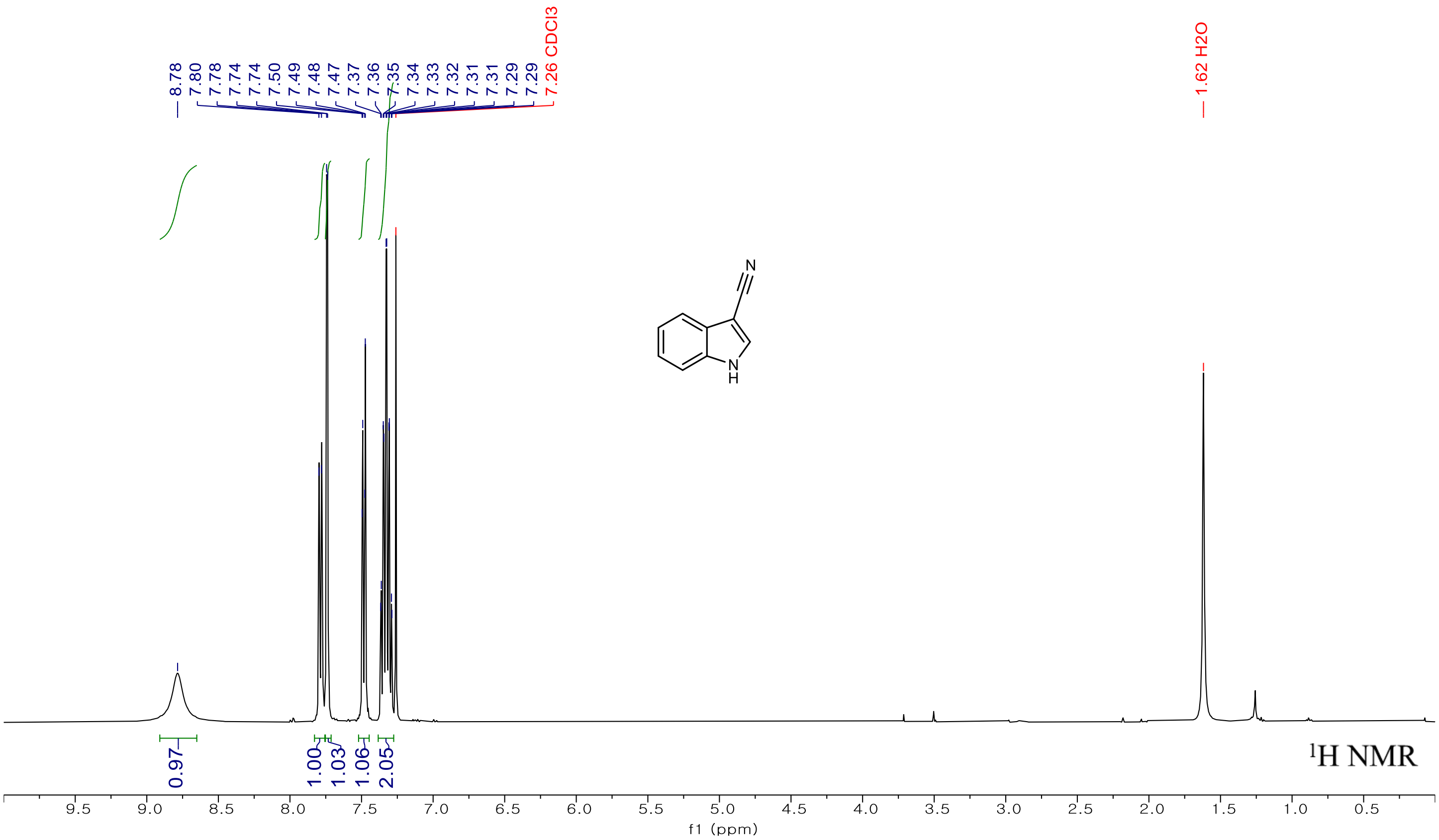


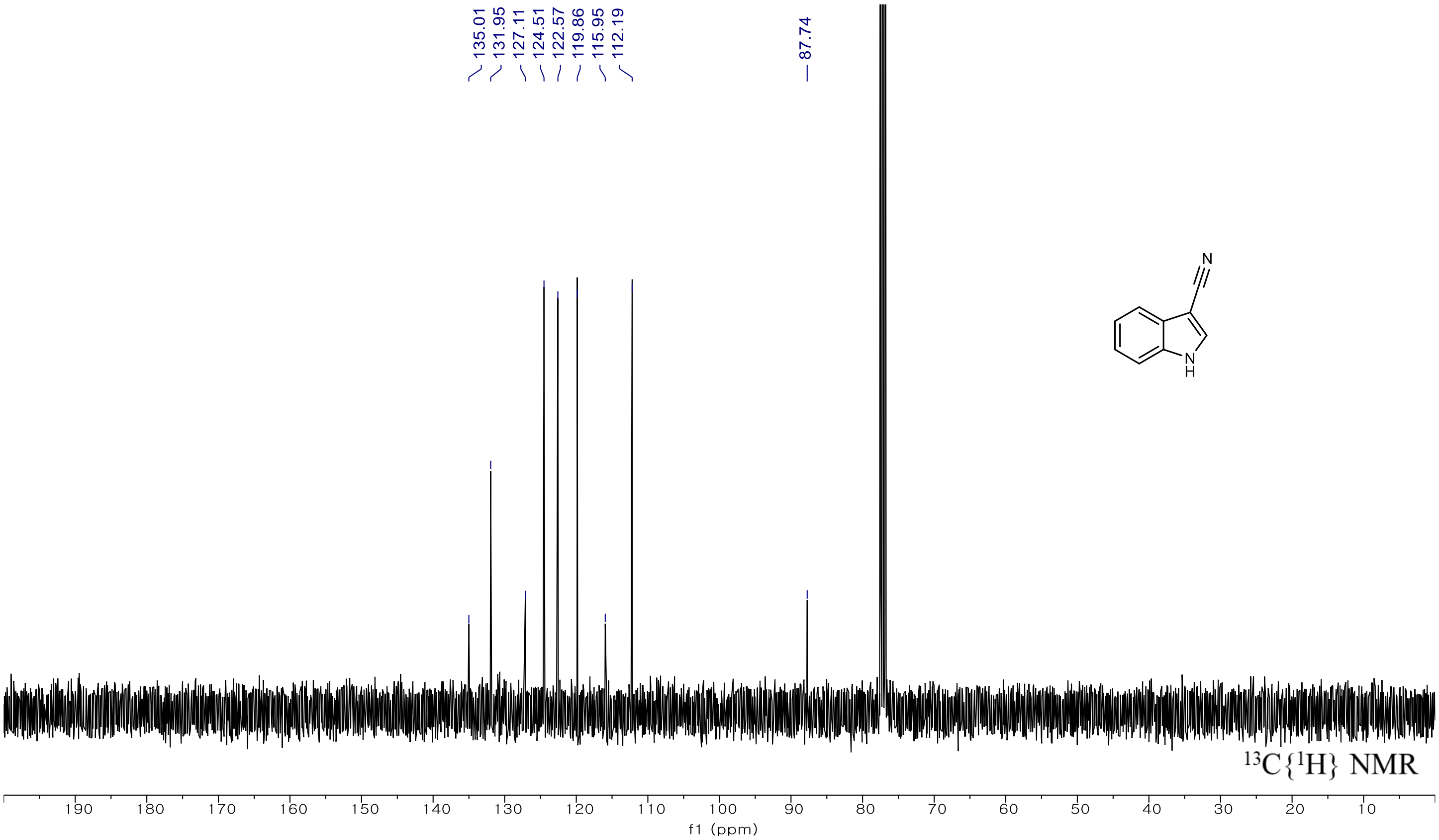

Terephthalonitrile (**4n**)

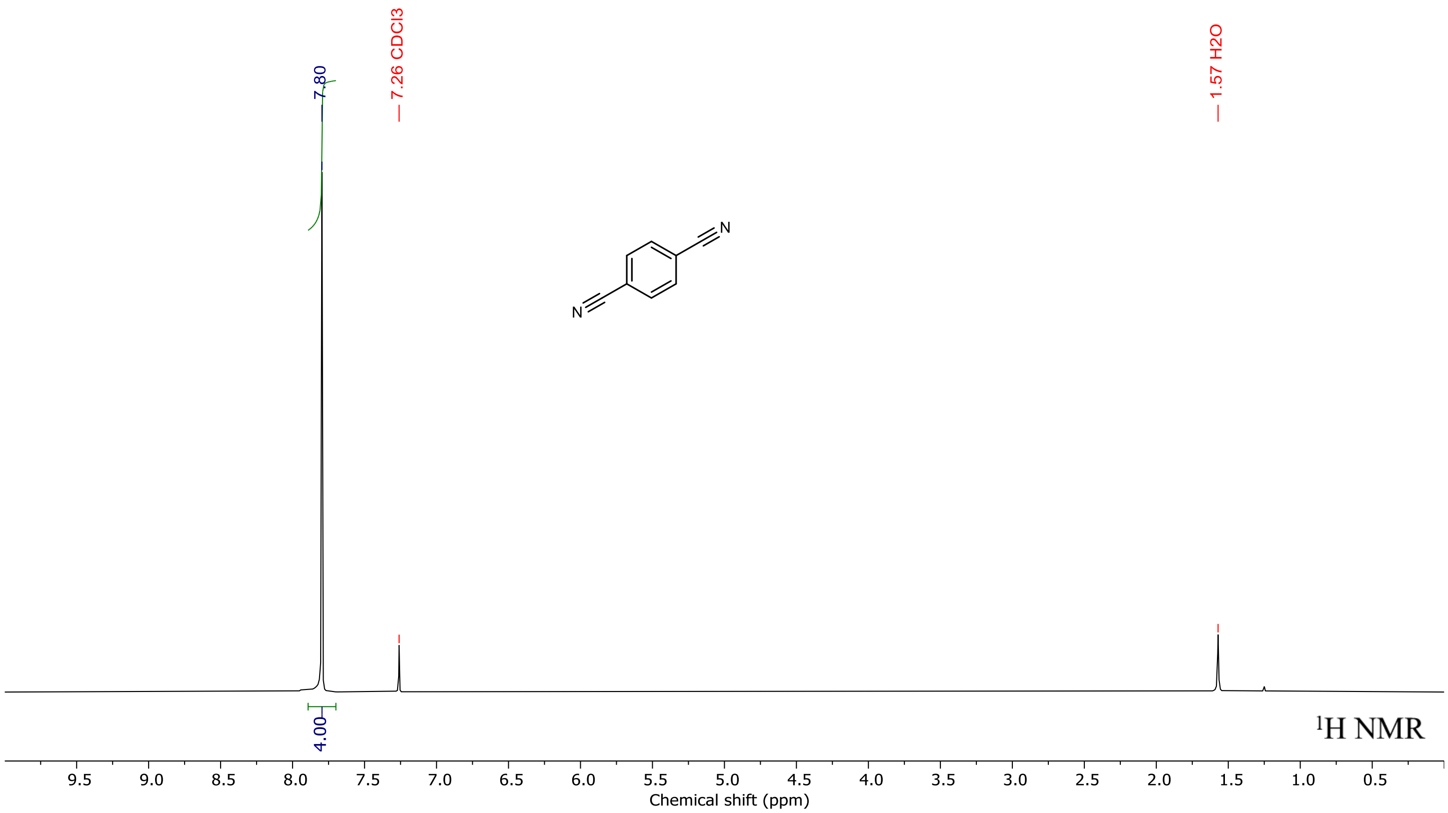


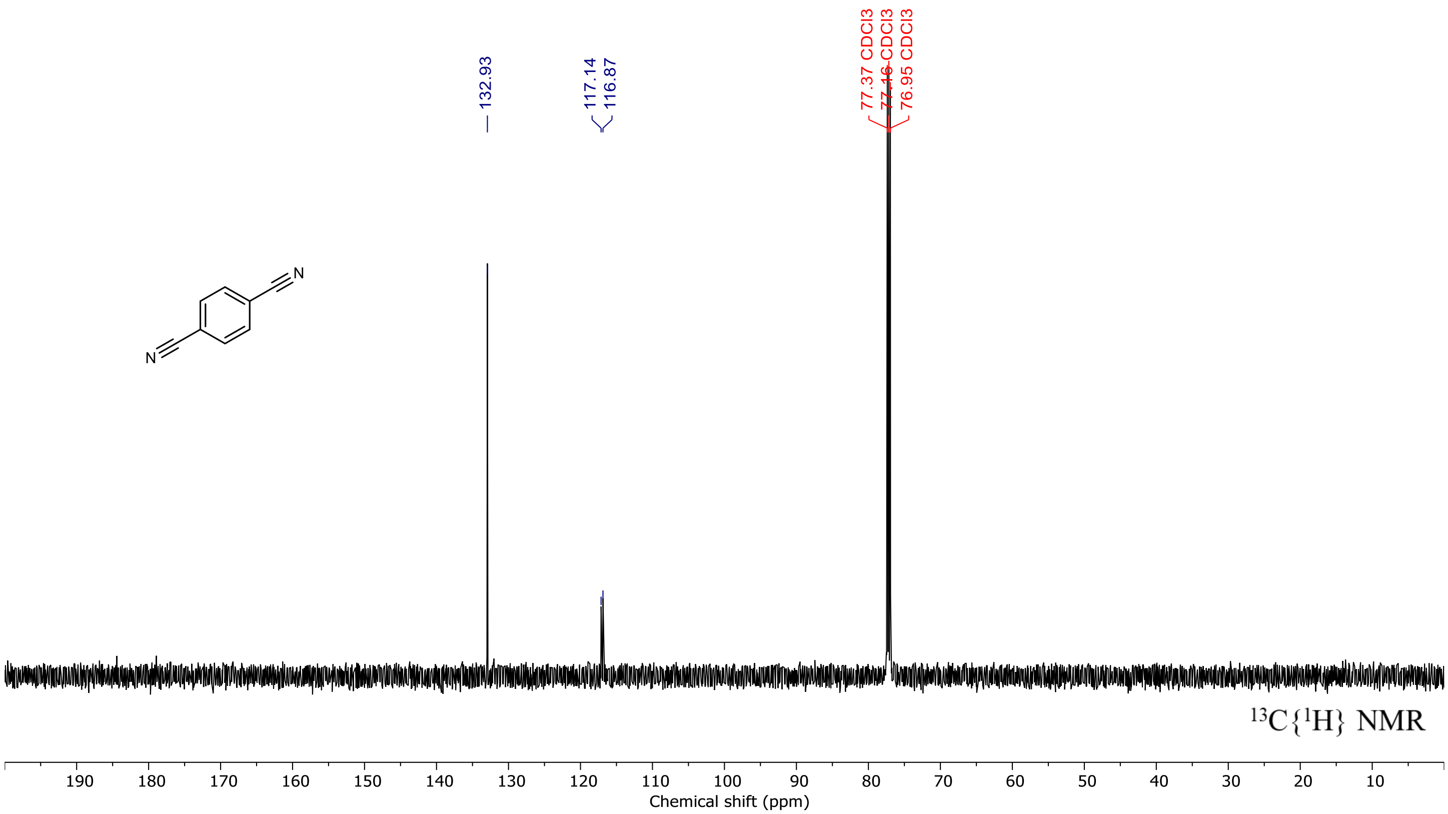

4-Chloropyridine-2,6-dicarbonitrile (**4o**)

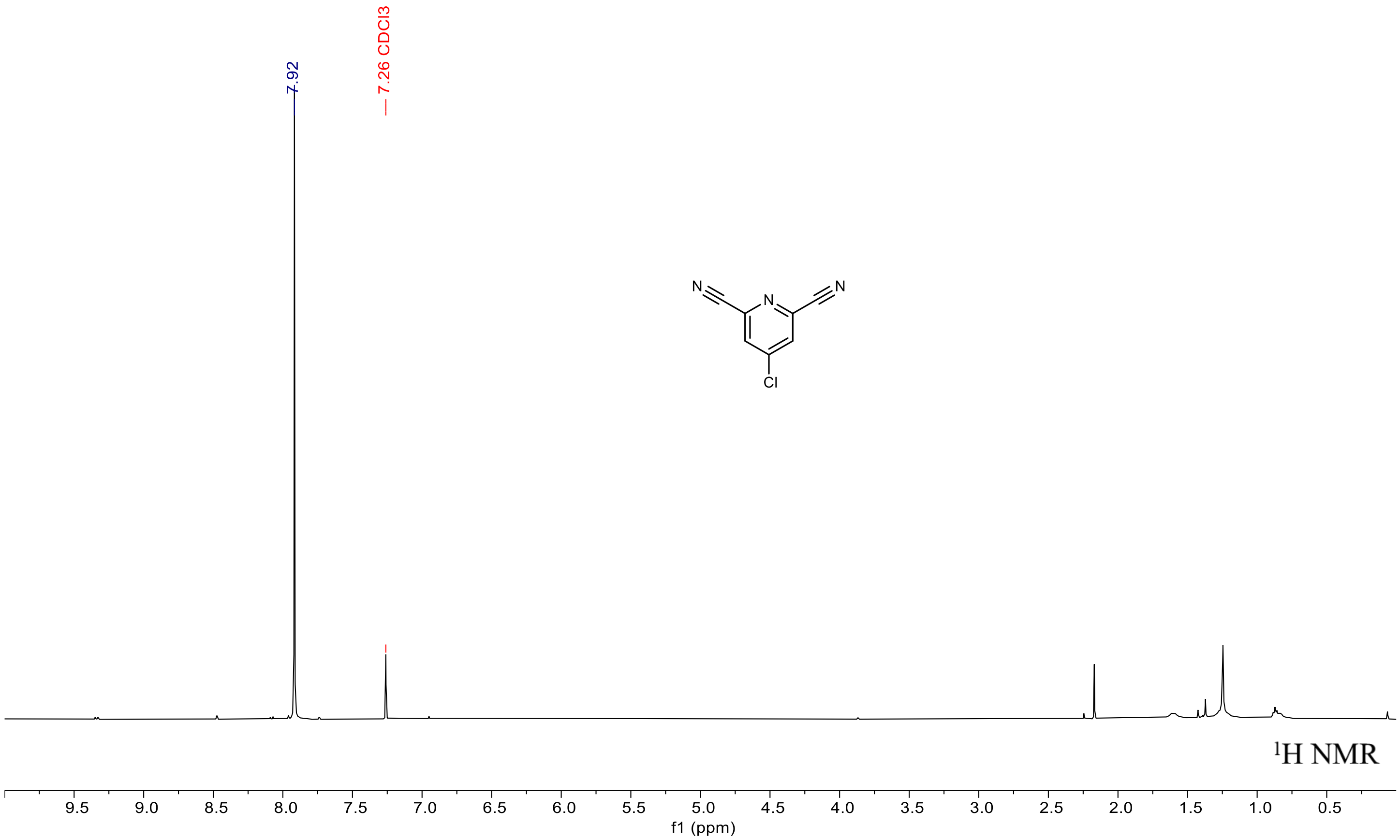


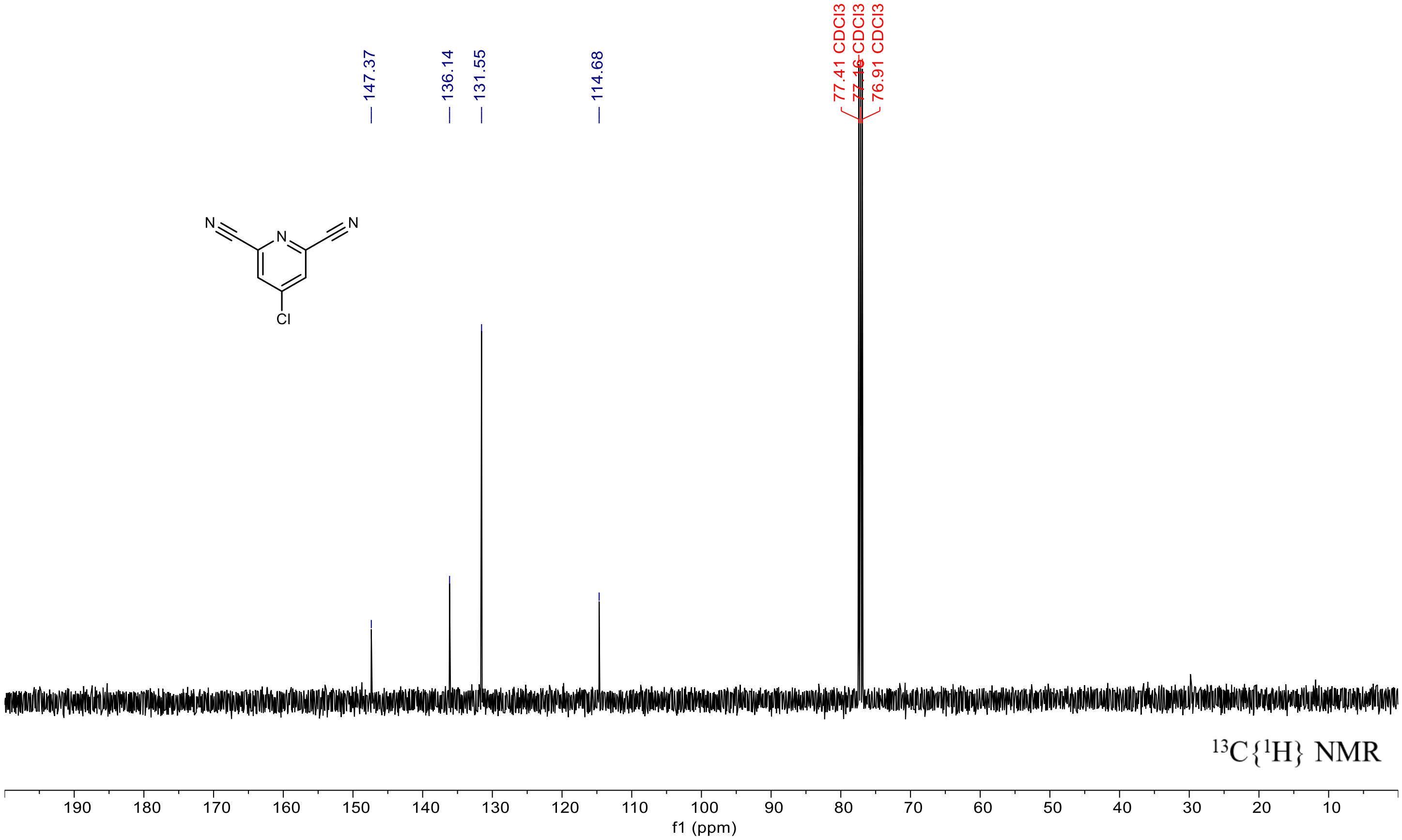

2-Oxo-2*H*-chromene-3-carbonitrile (**4p**)

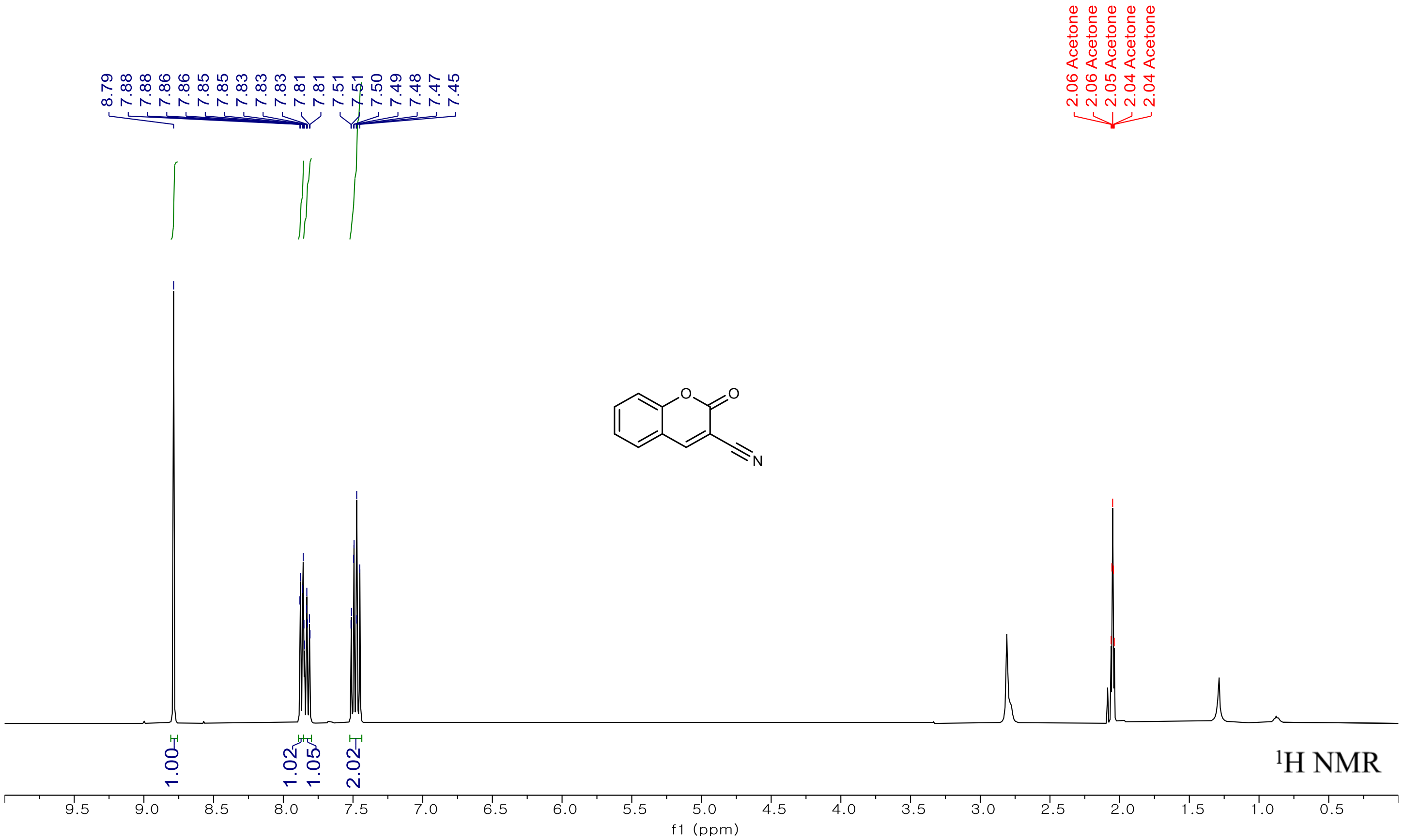


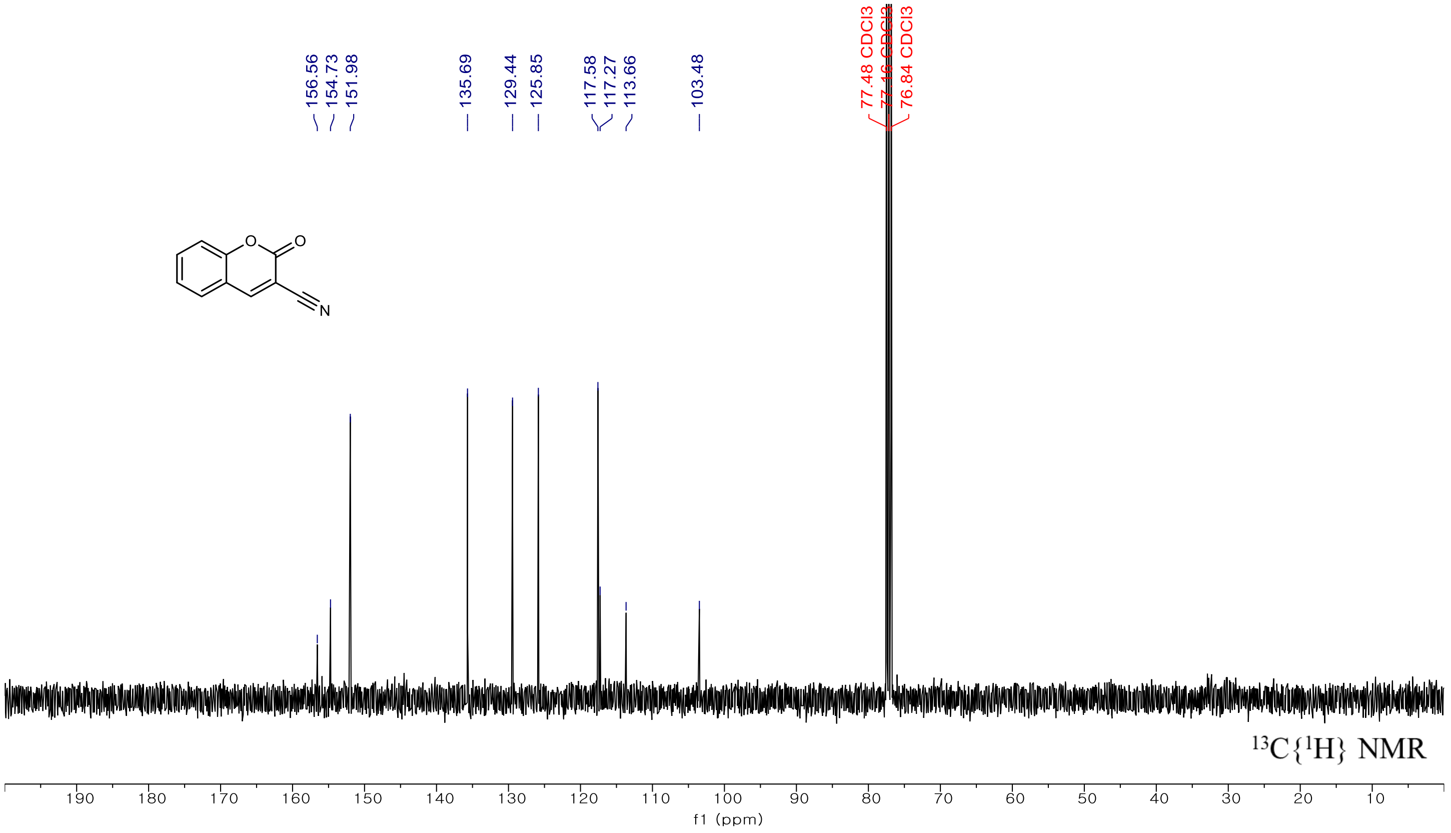

Cinnamonitrile (**4q**)

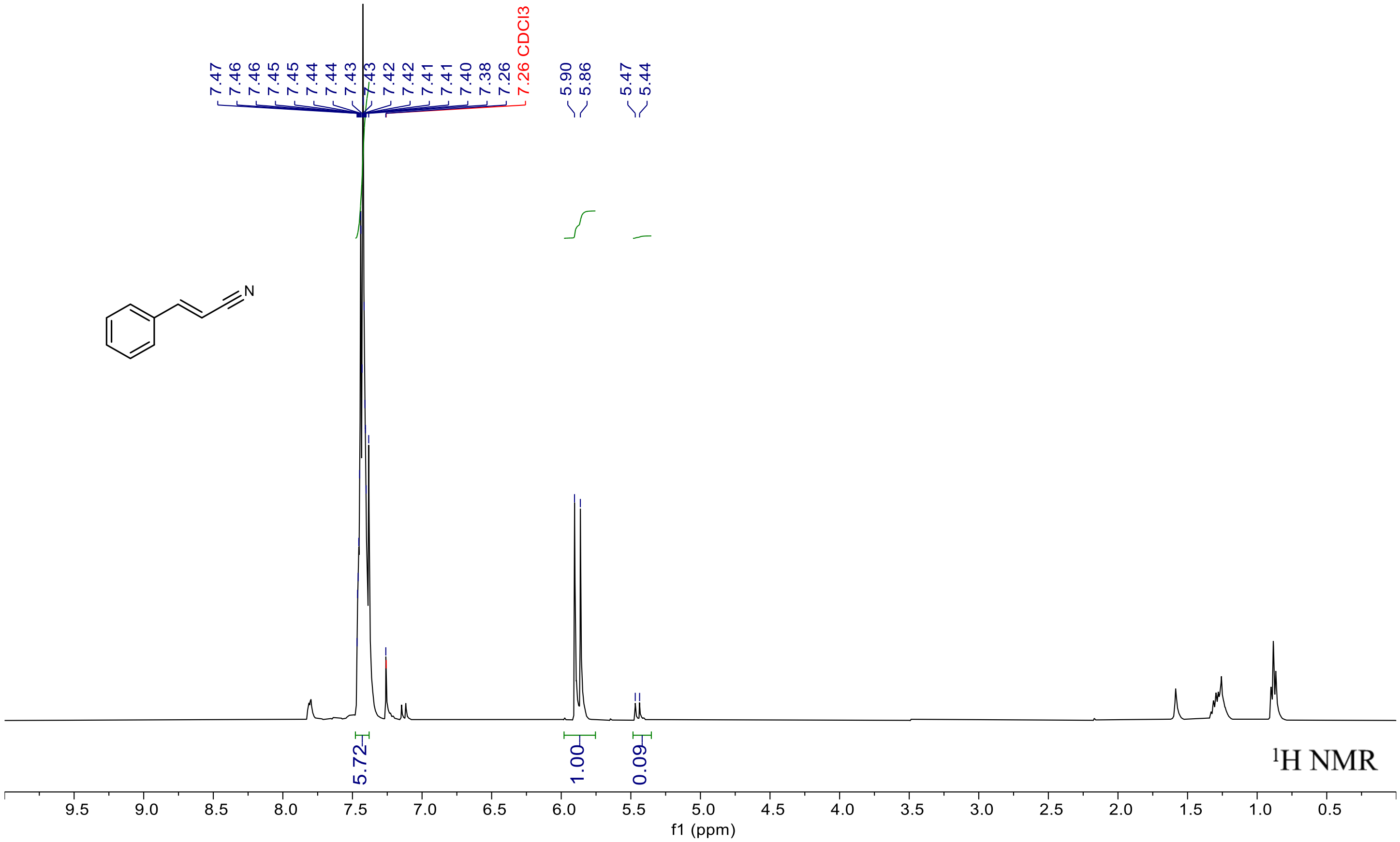


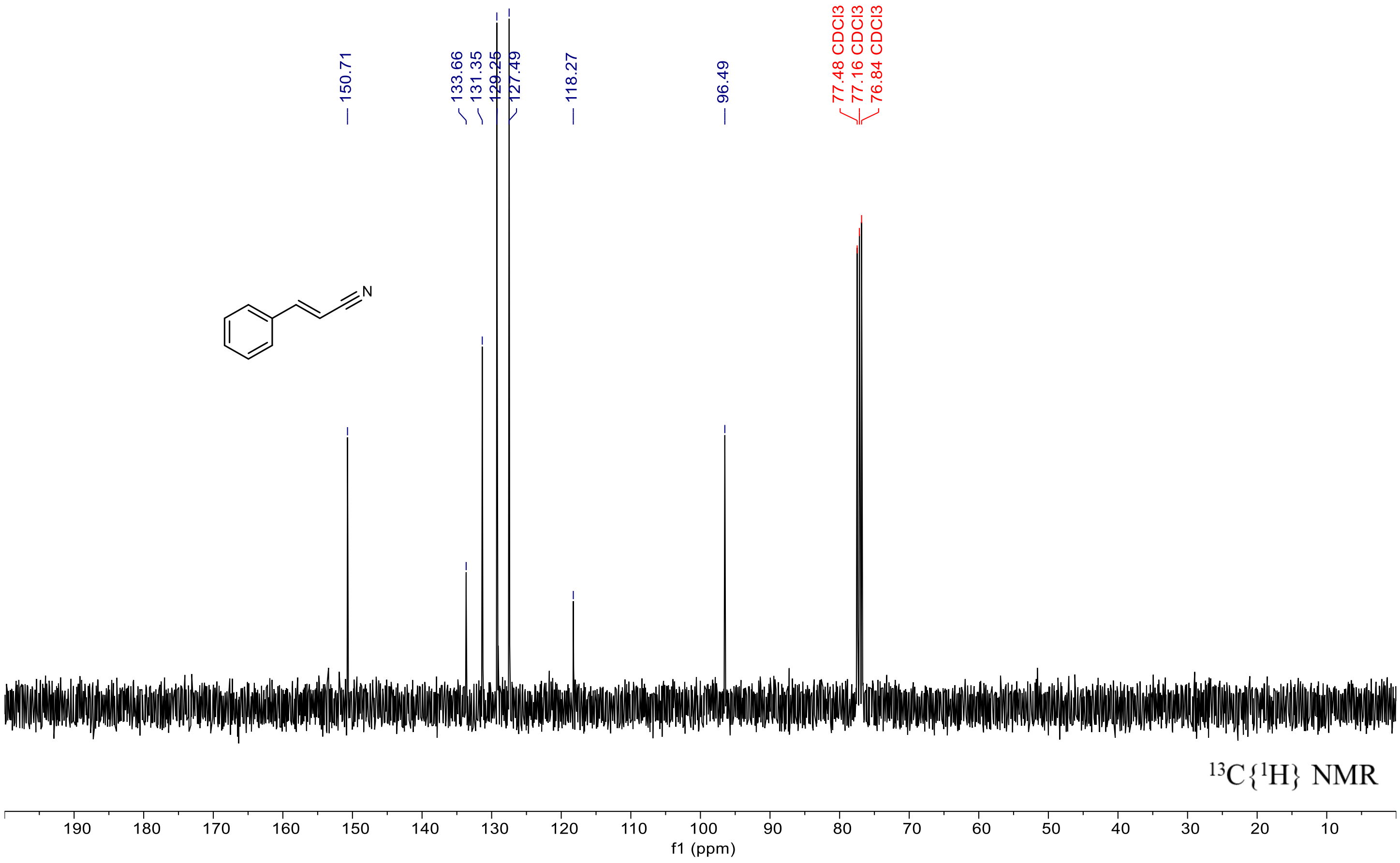

3-(4-Bromophenyl)propanenitrile (**4r**)

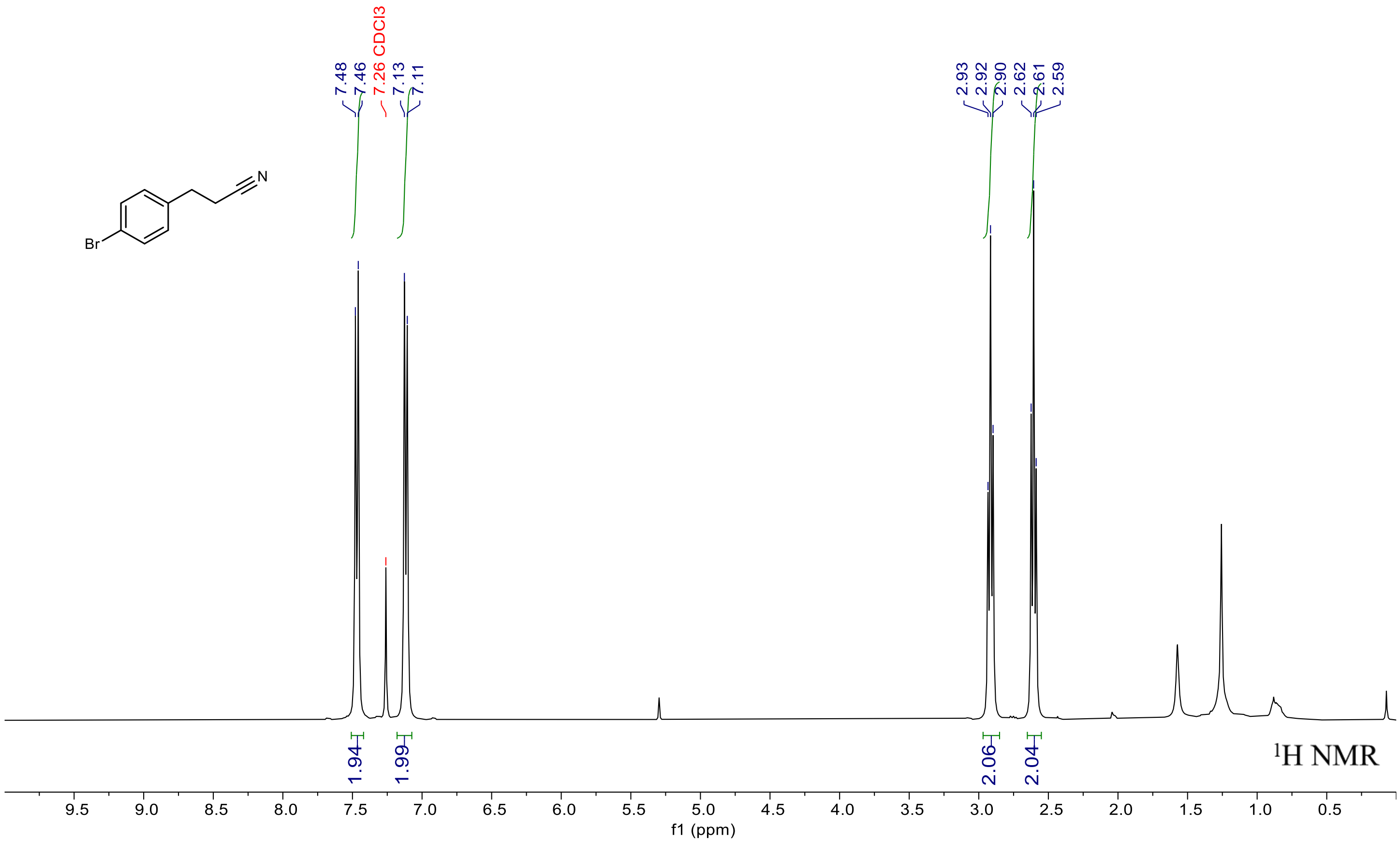


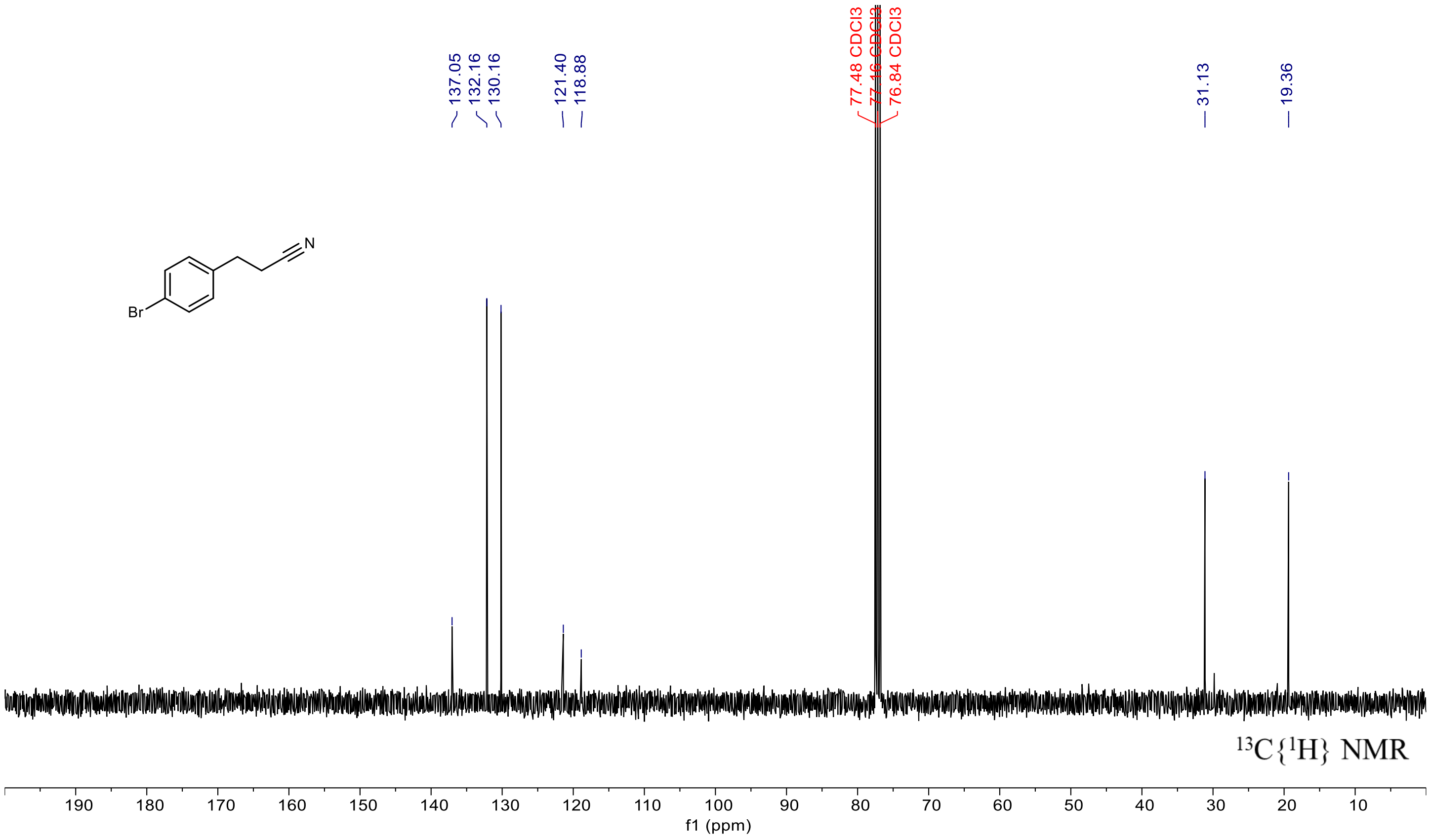

2-(*p*-Tolyloxy)acetonitrile (**4s**)

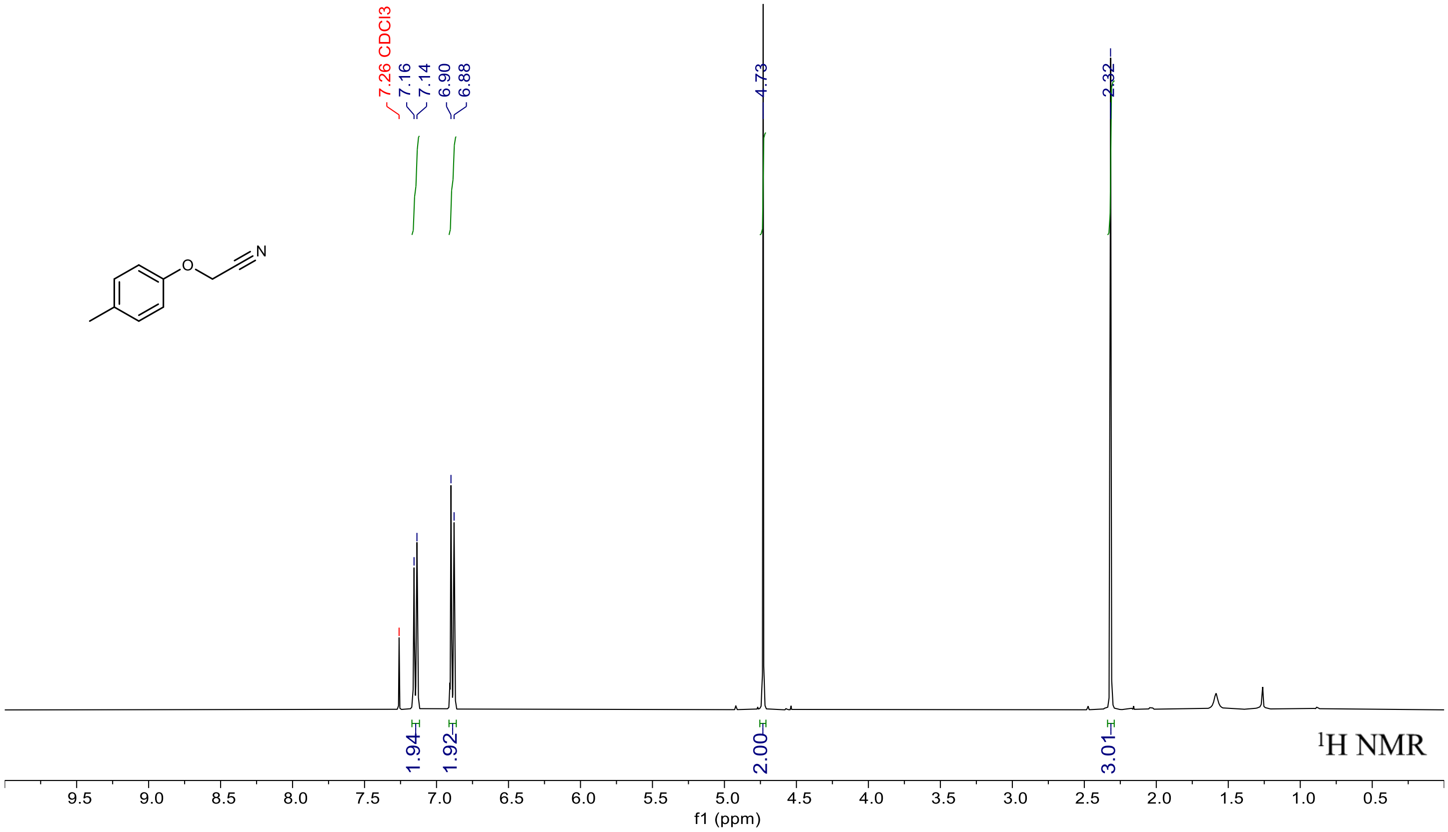


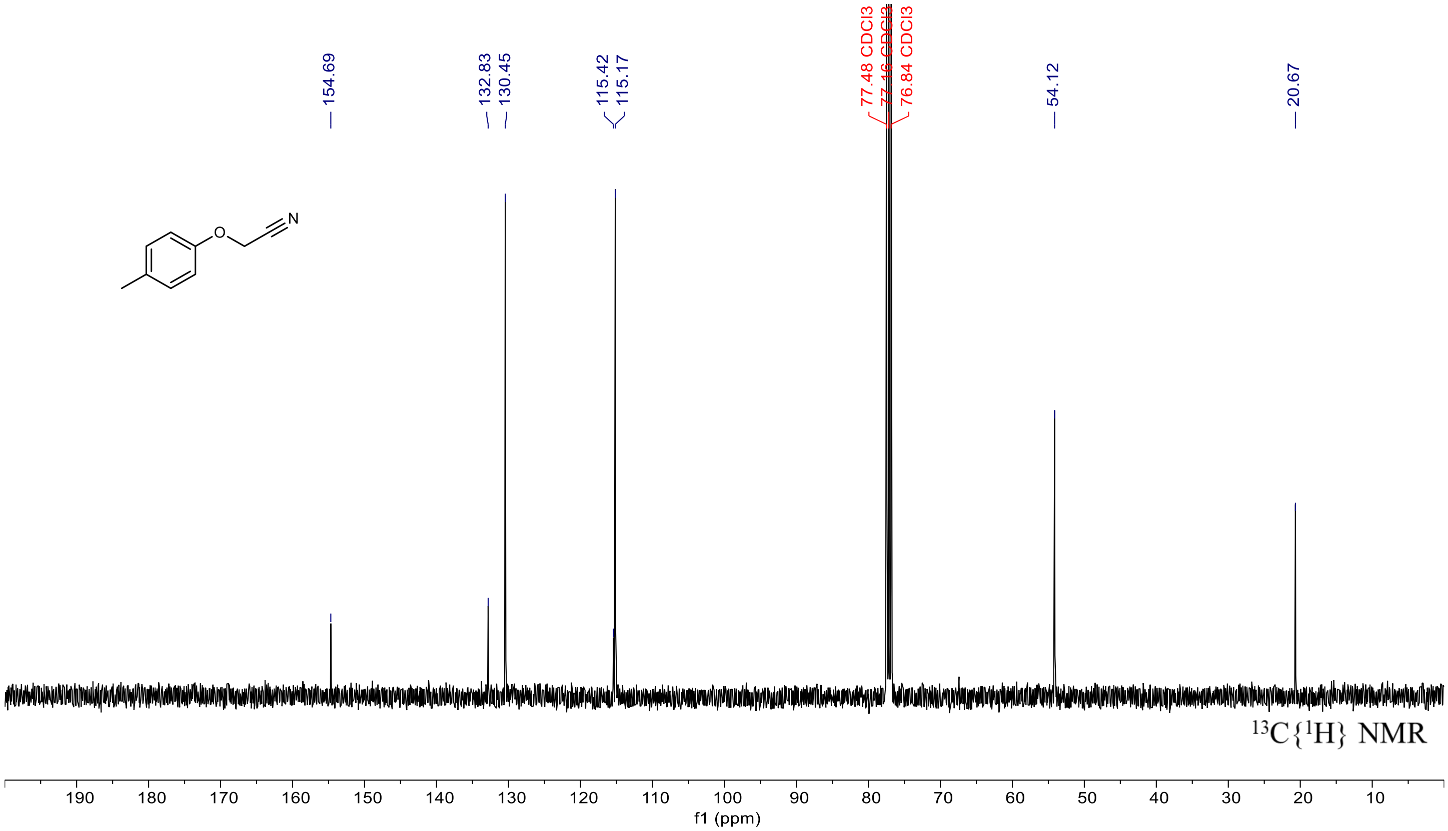

*N*-(Cyanomethyl)-2-phenylacetamide (**4t**)

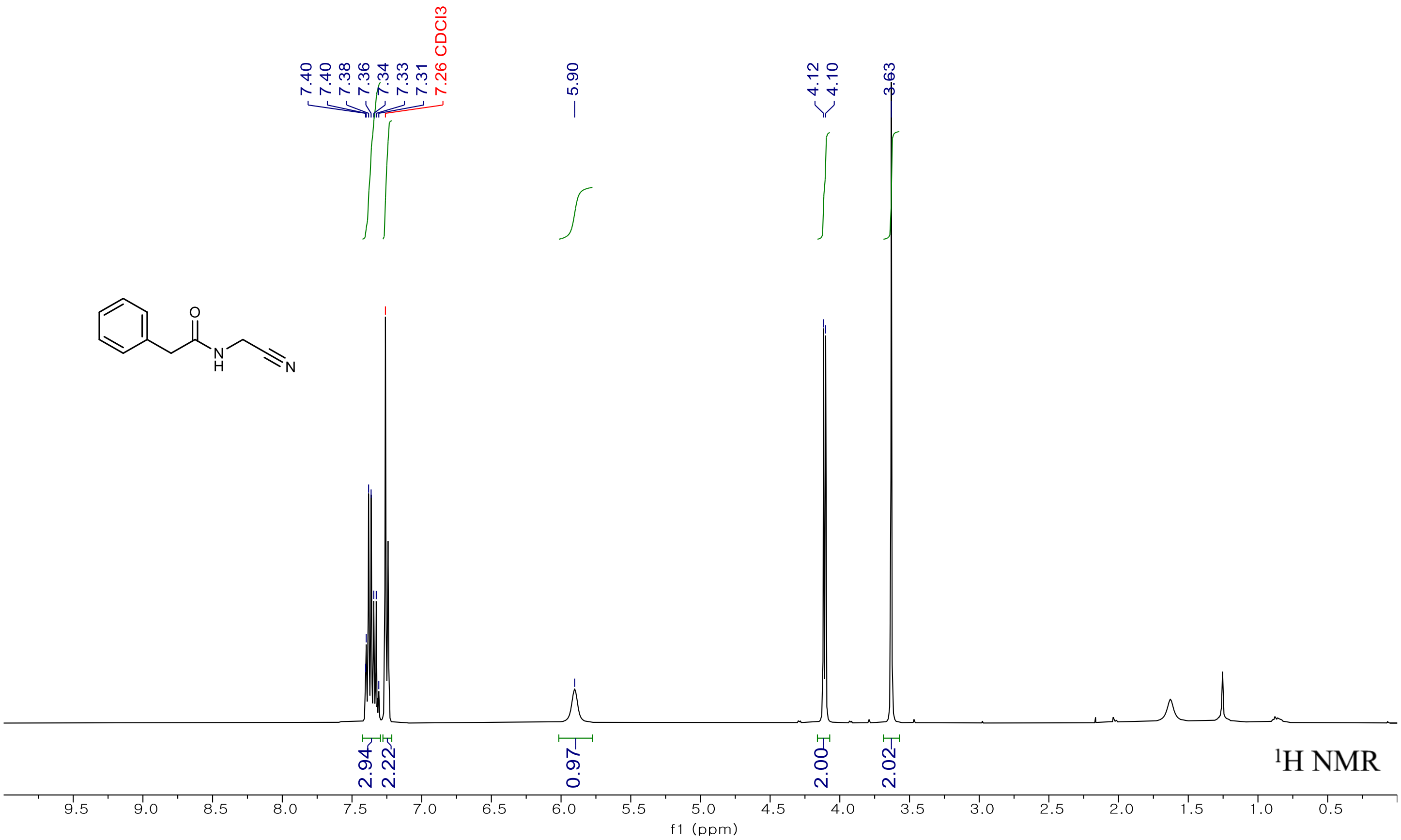


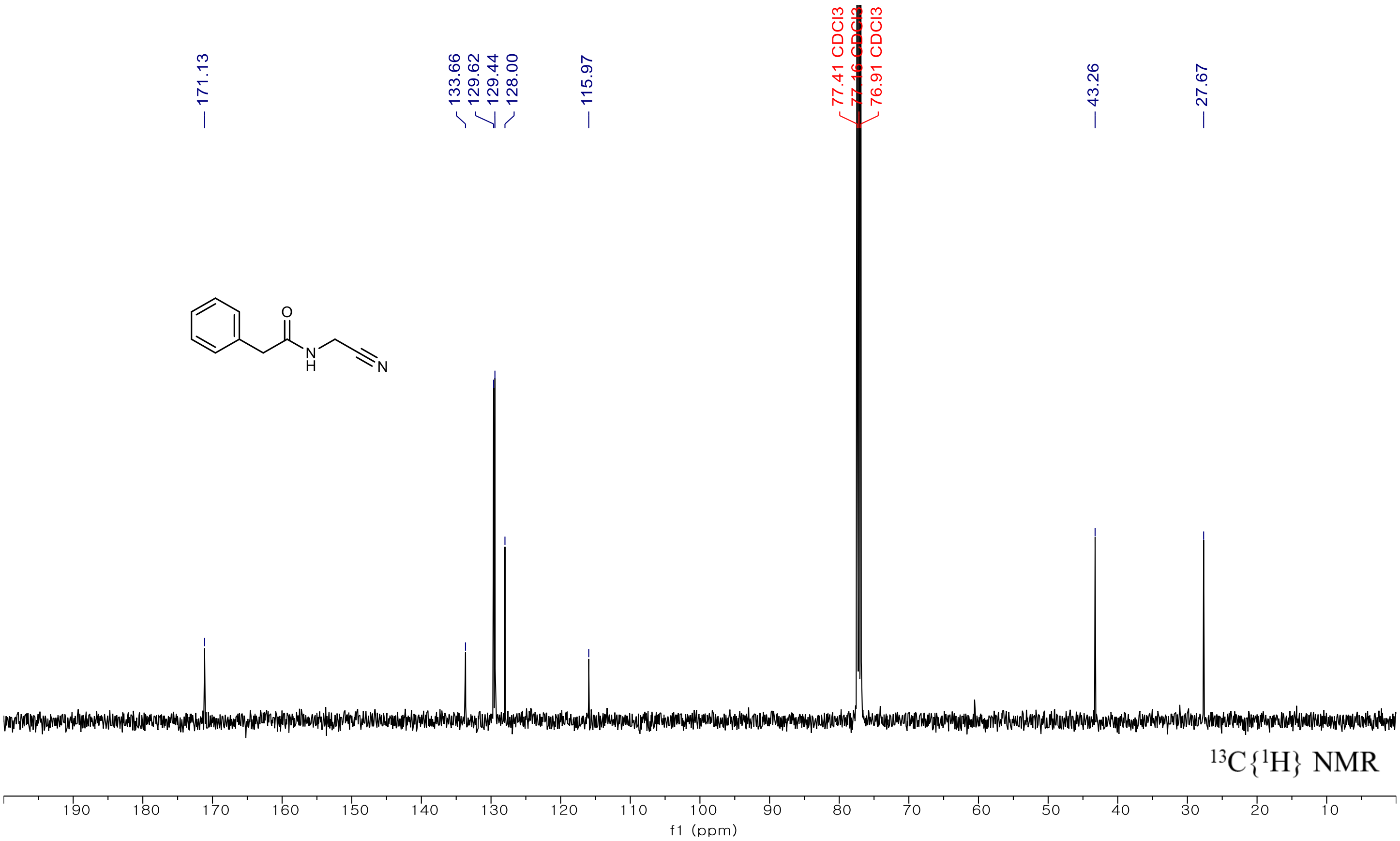

2-(Benzo[*d*][1,3]dioxol-5-yl)acetonitrile (**4u**)

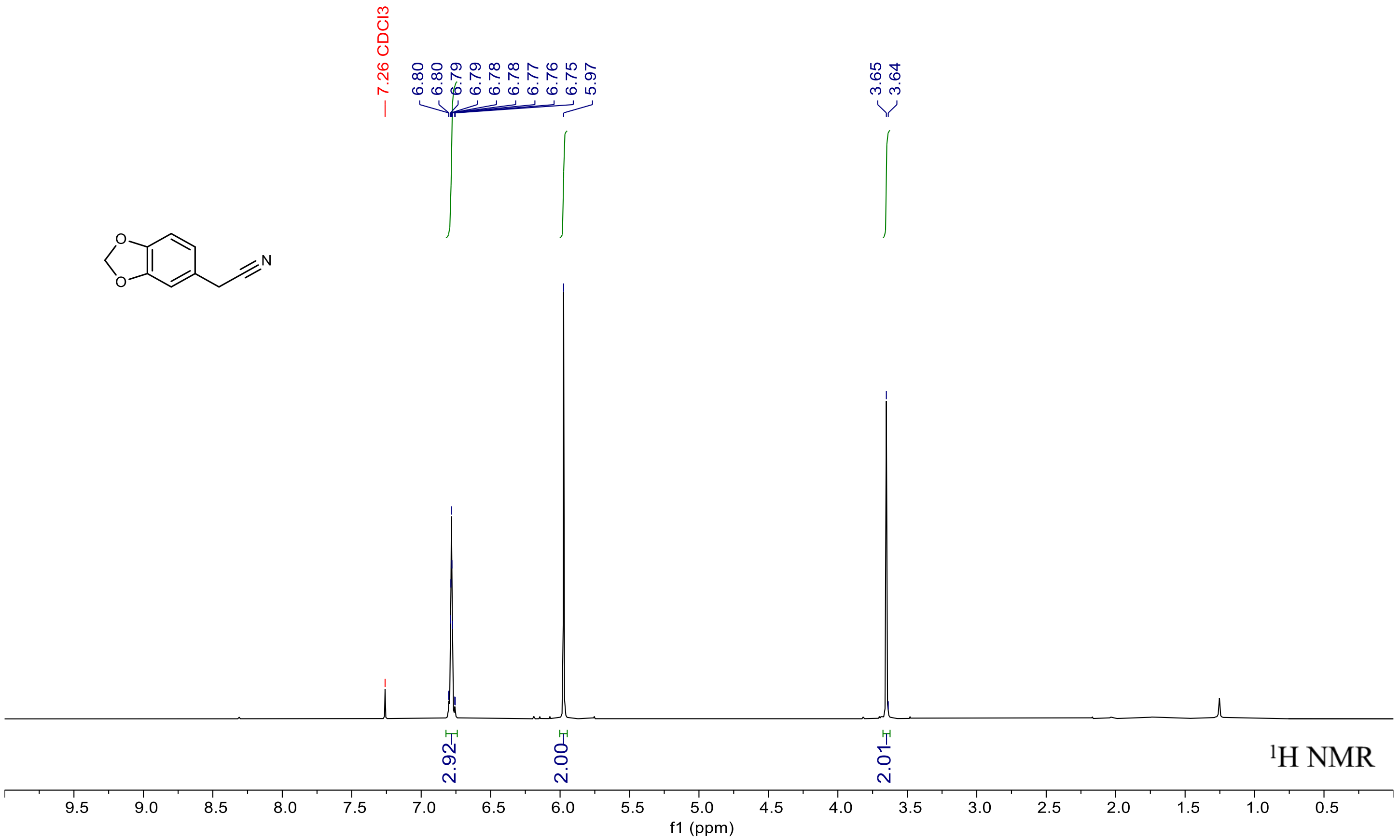


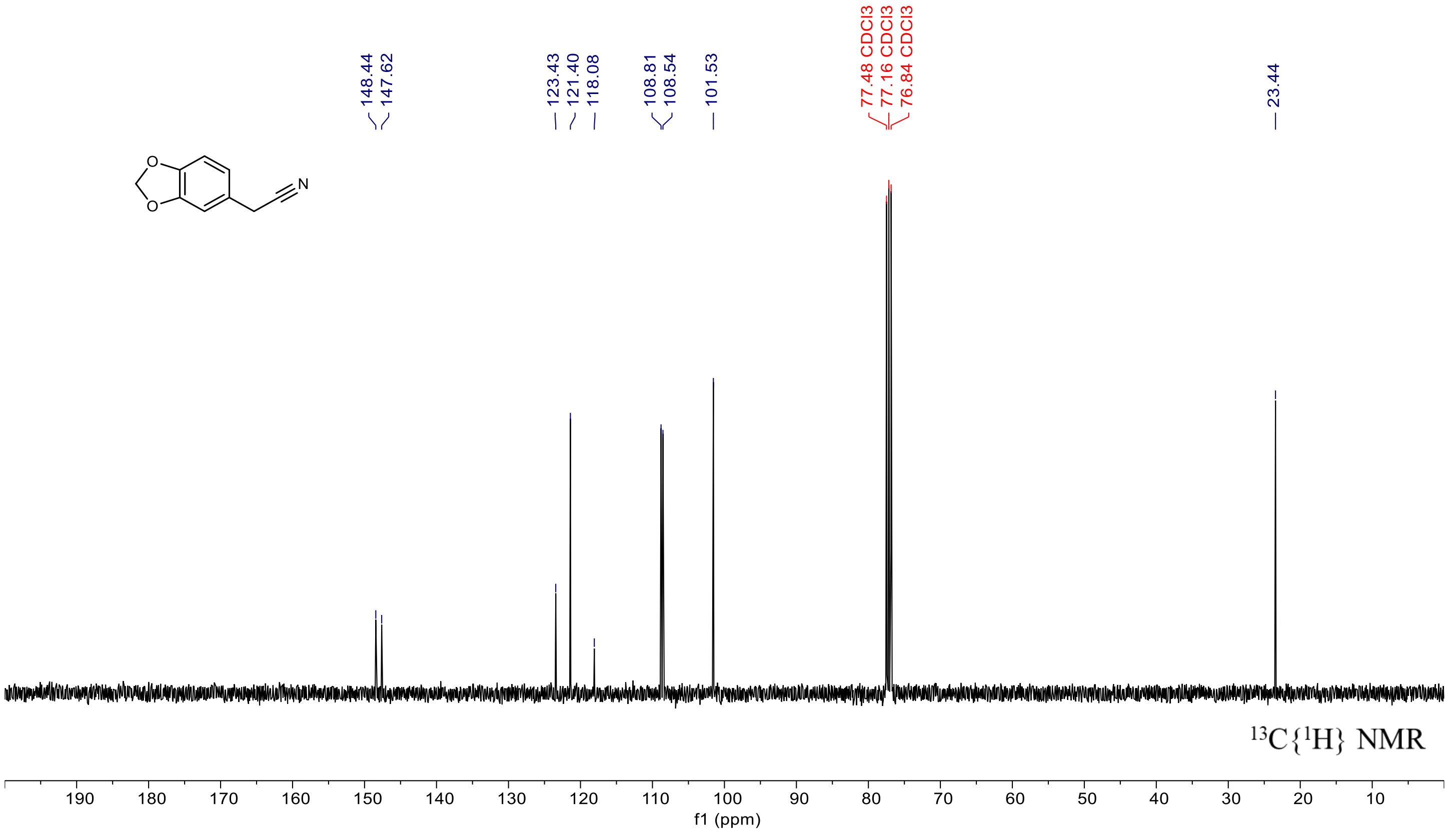

1-Phenylcyclopropane-1-carbonitrile (**4v**)

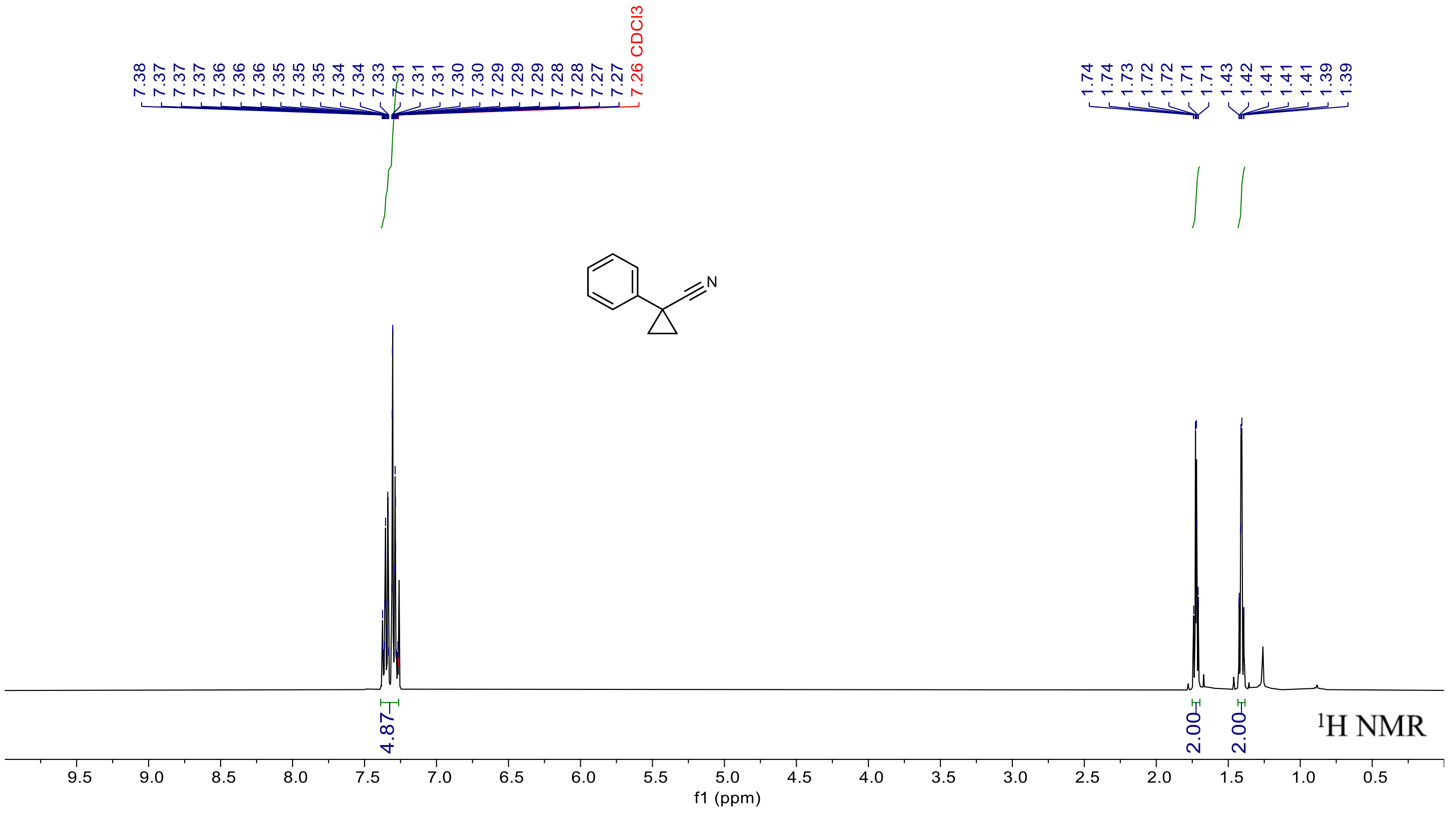


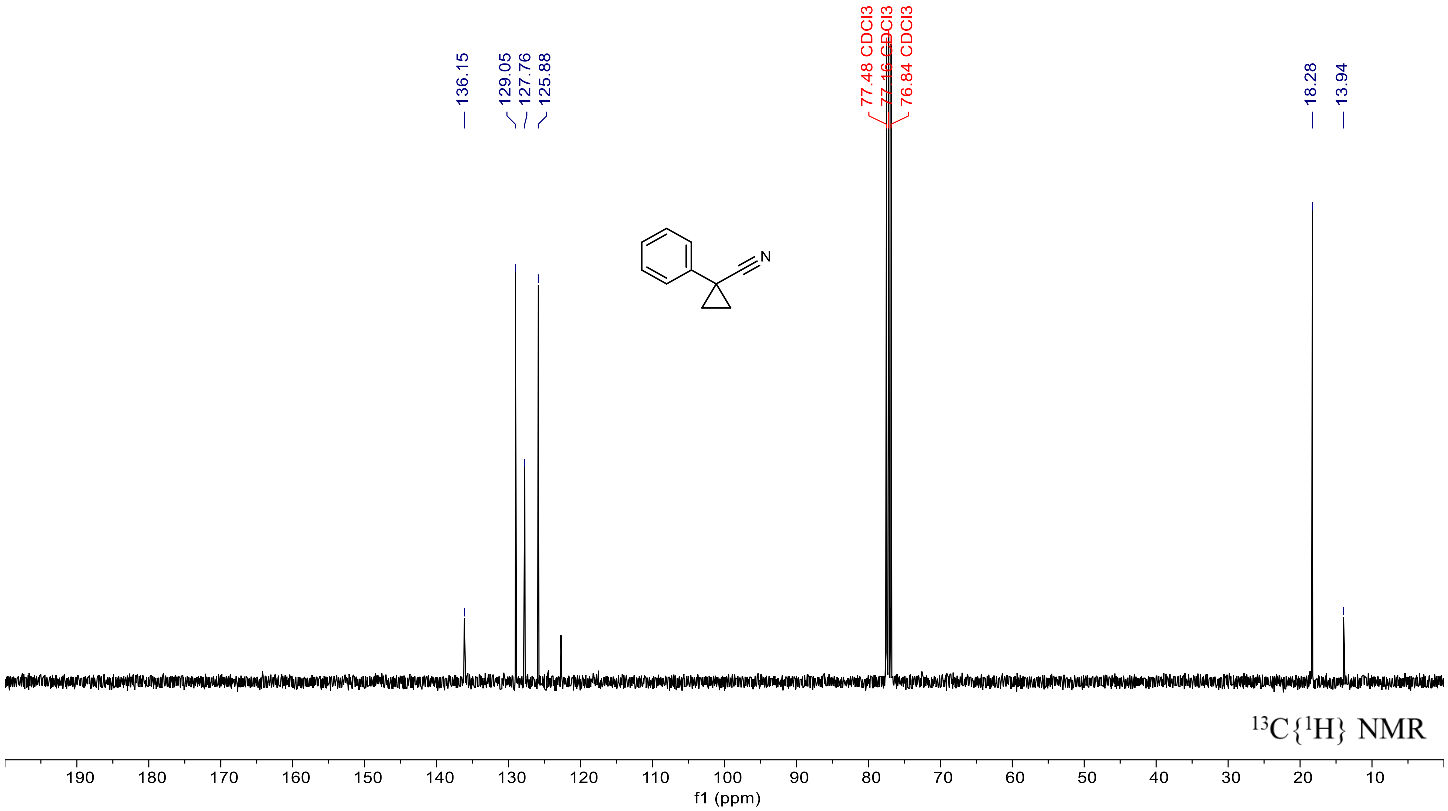

Spiro[3.3]heptane-2-carbonitrile (**4w**)

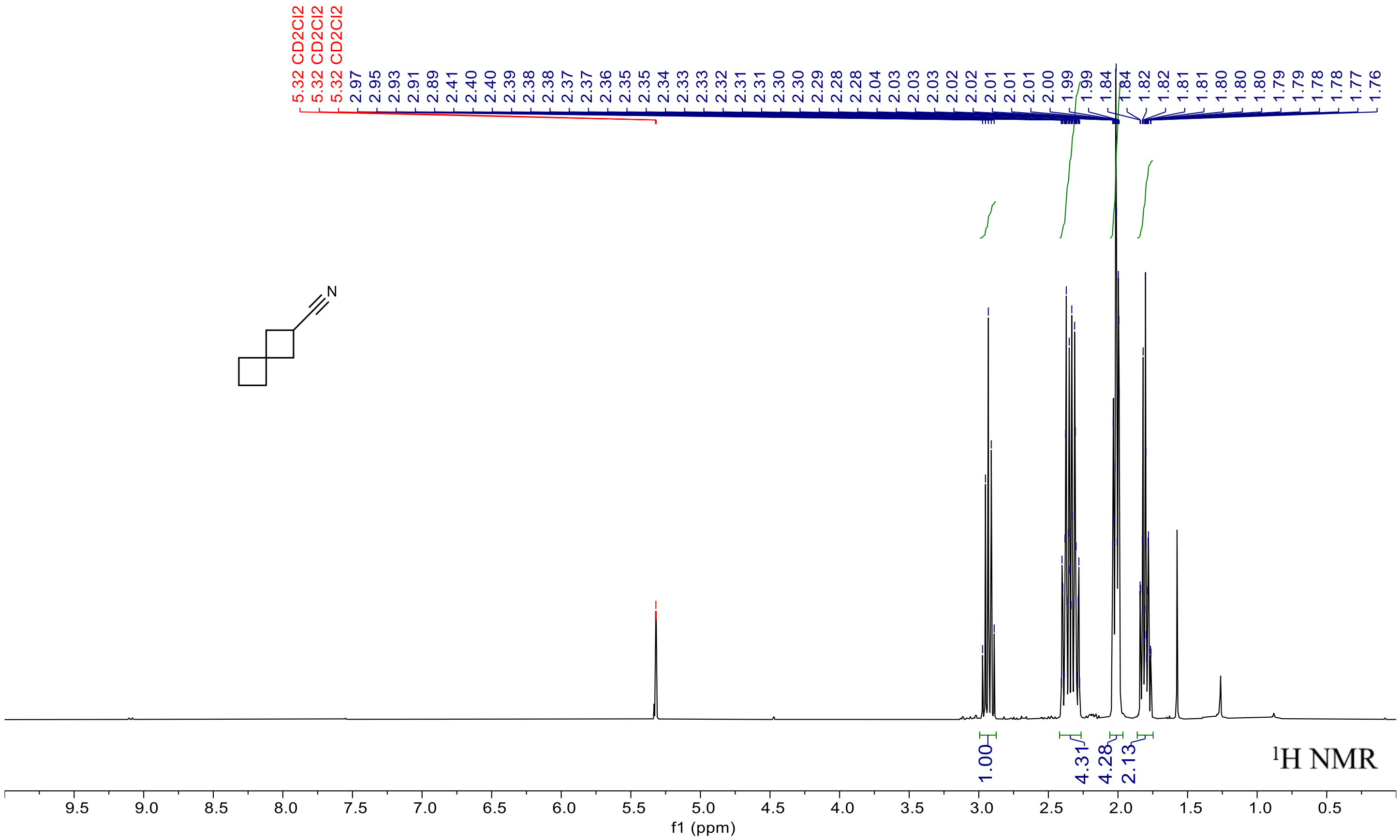


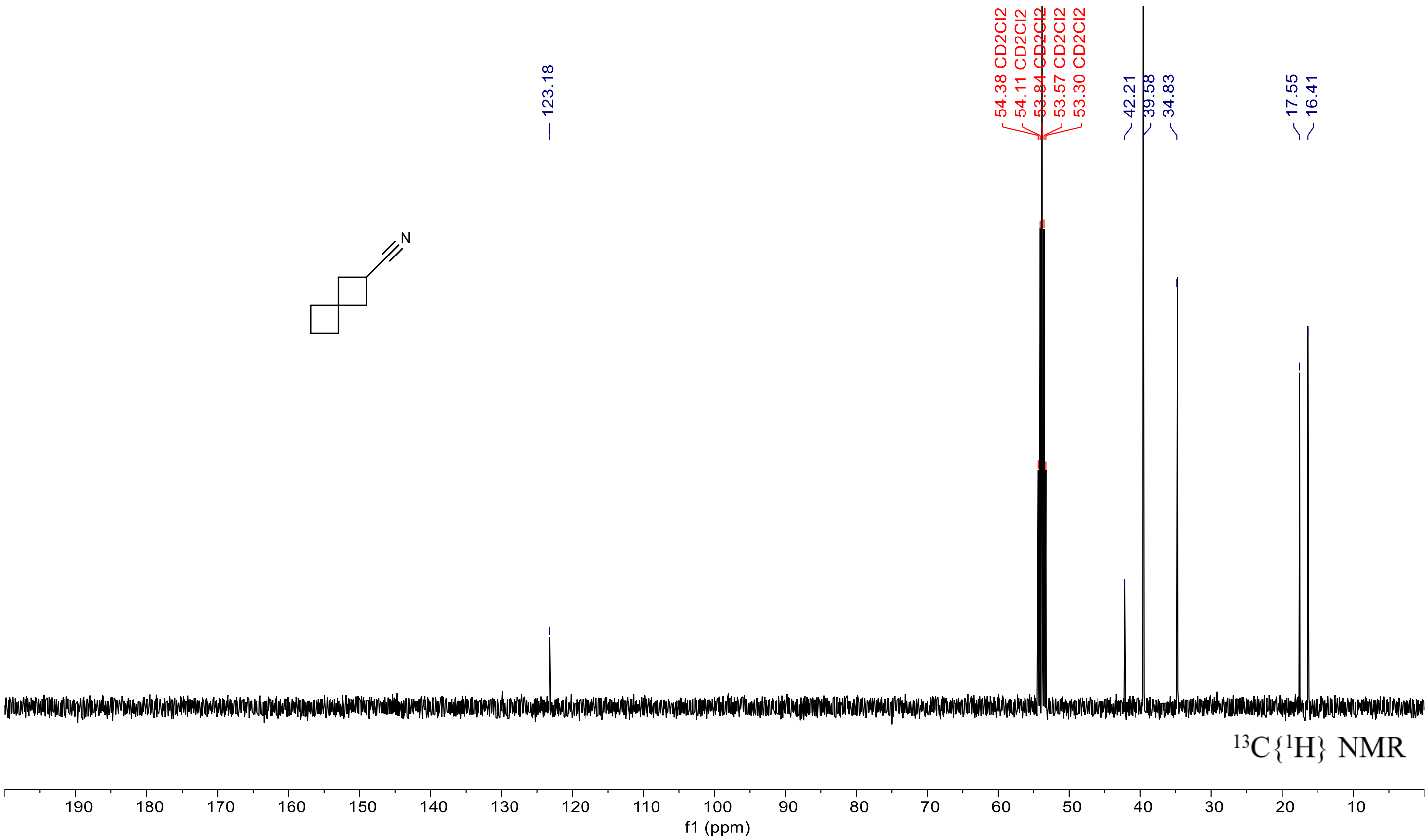

2,2,2-Triphenylacetonitrile (**4x**)

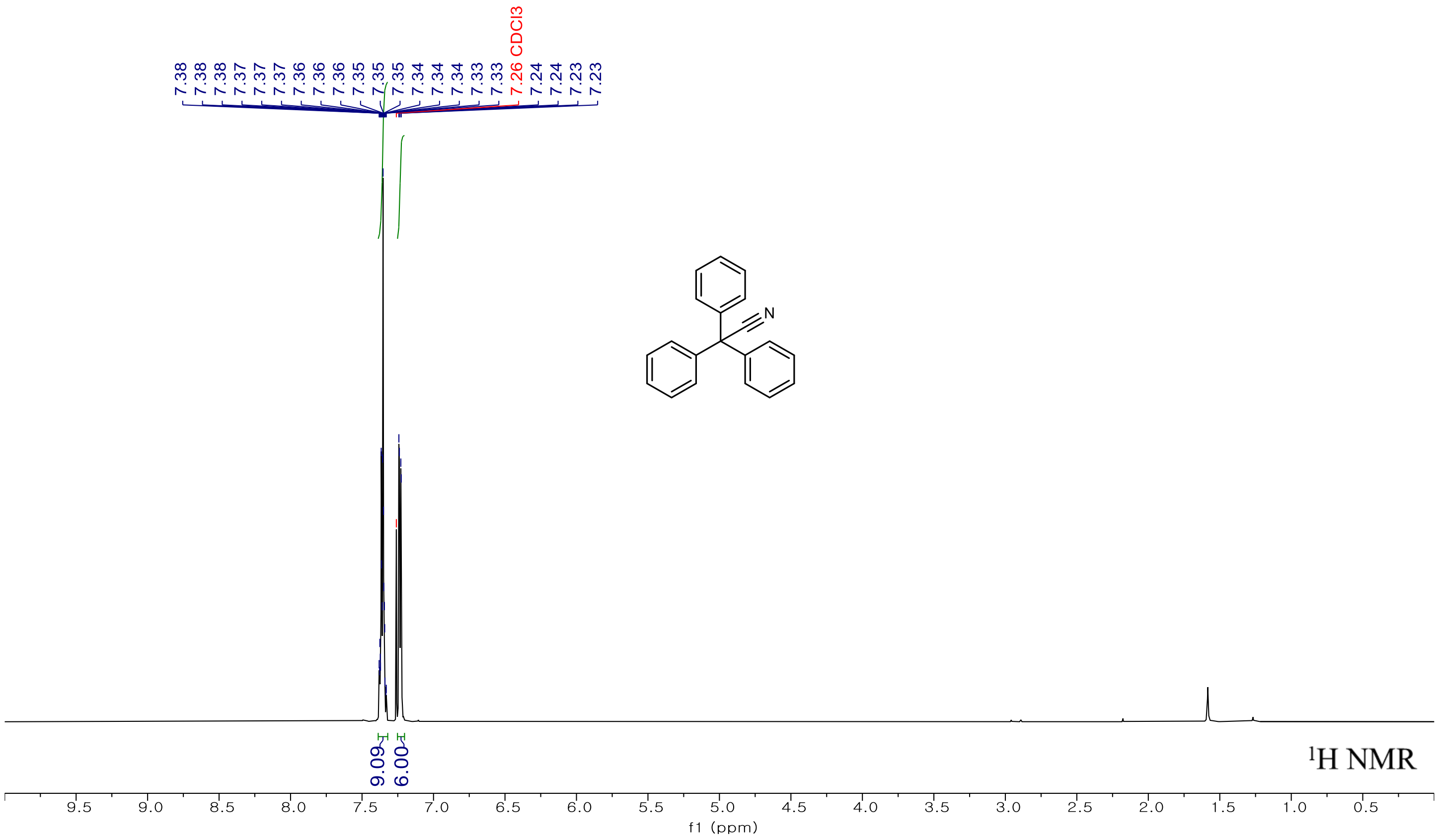


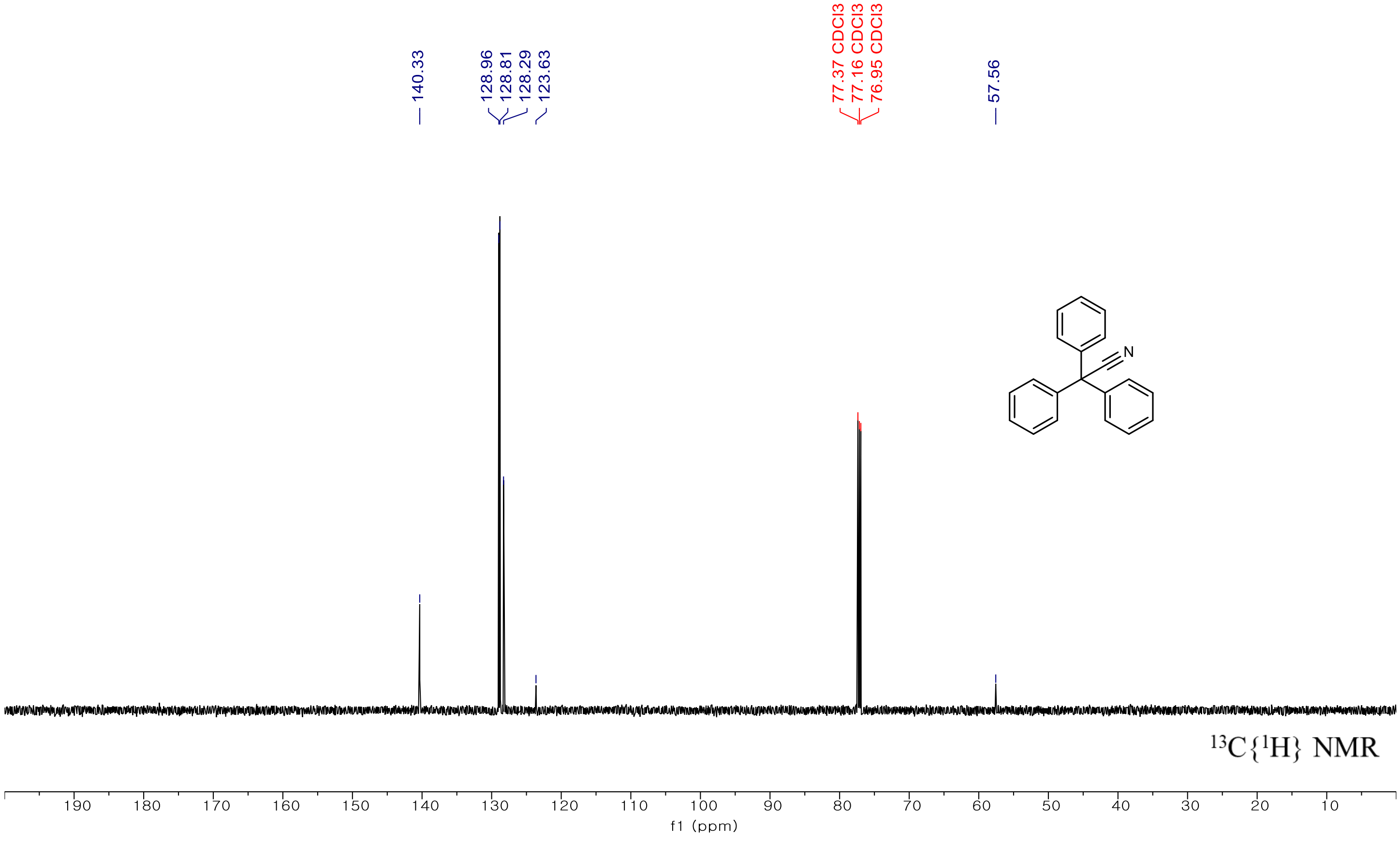

4-Cyano-*N*,*N*-dipropylbenzenesulfonamide (**4y**)

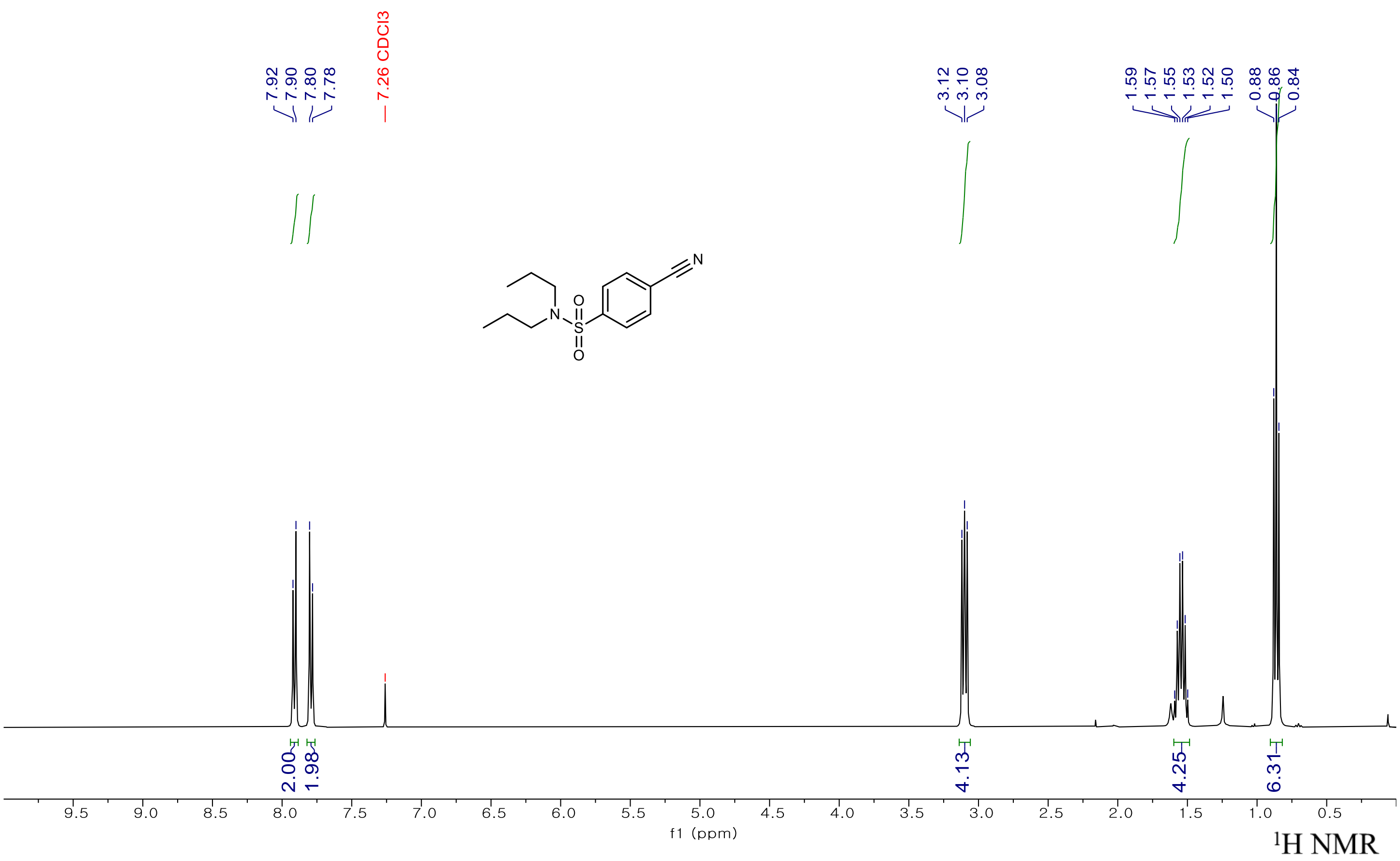


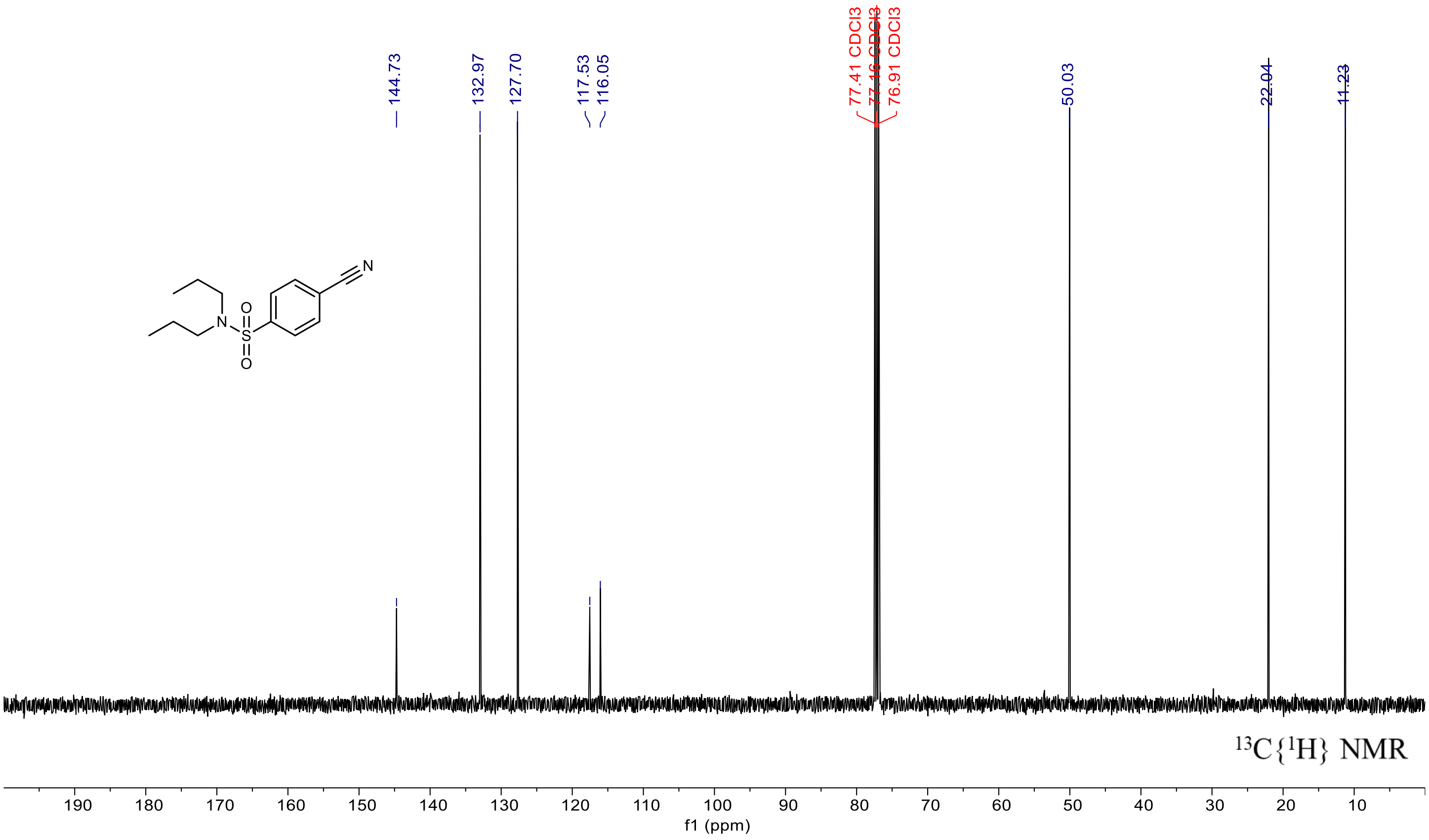

2-(4-Cyano-3-ethoxyphenyl)-*N*-{3-methyl-1-[2-(piperidin-1-yl)phenyl]butyl}ccacetamide (**4z**)

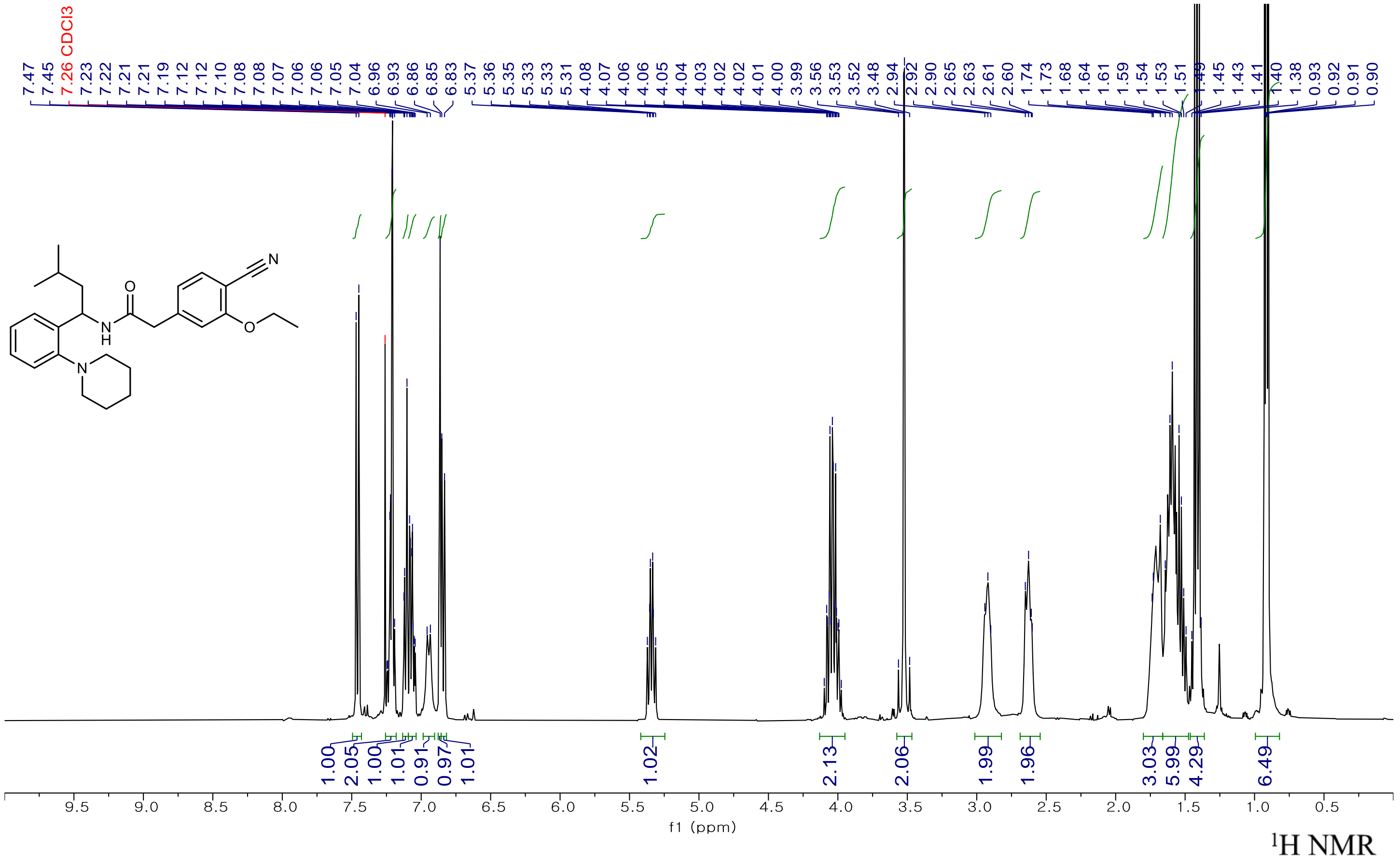

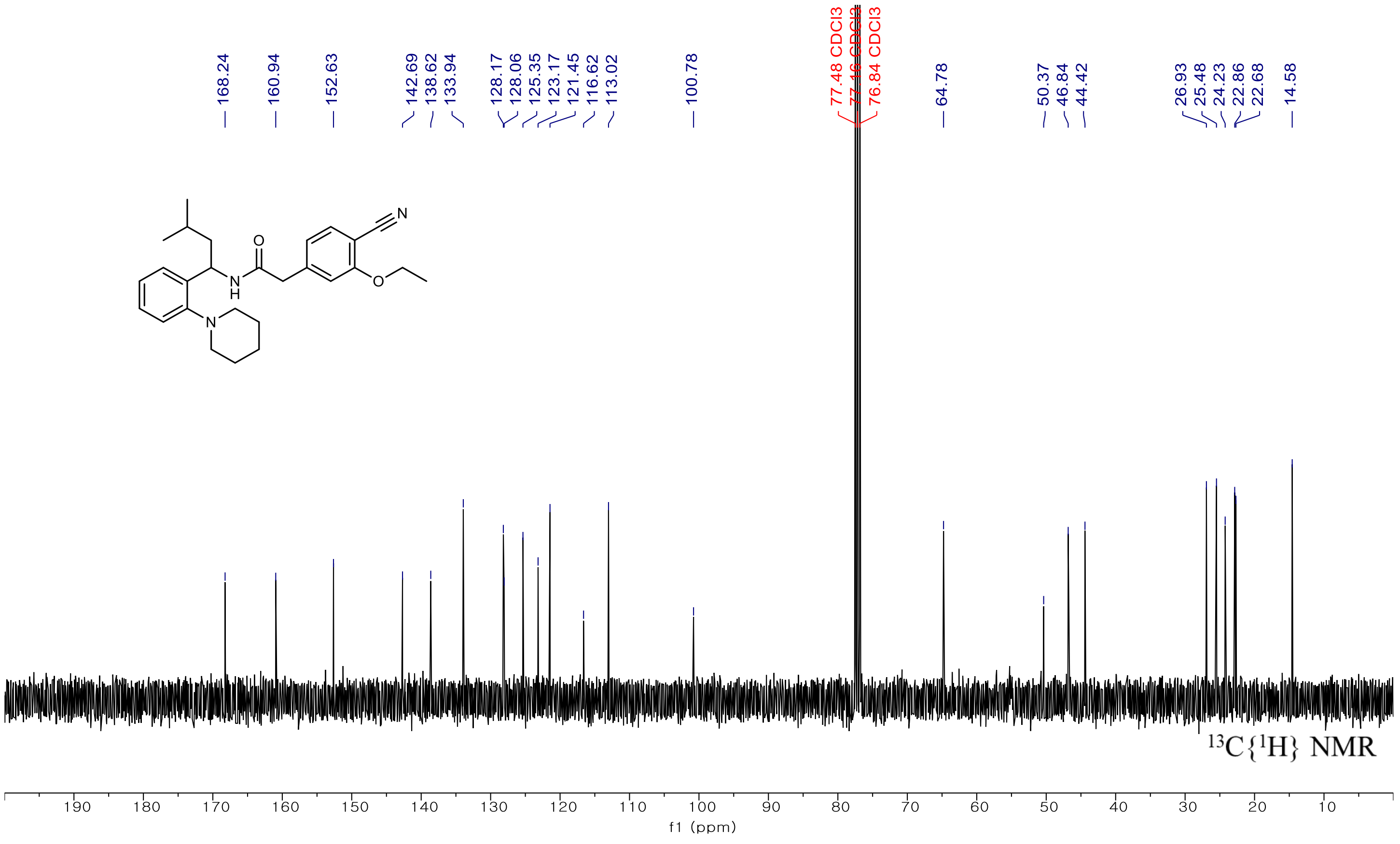

1-Ethyl-7-methyl-4-oxo-1,4-dihydro-1,8-naphthyridine-3-carbonitrile (**4aa**)

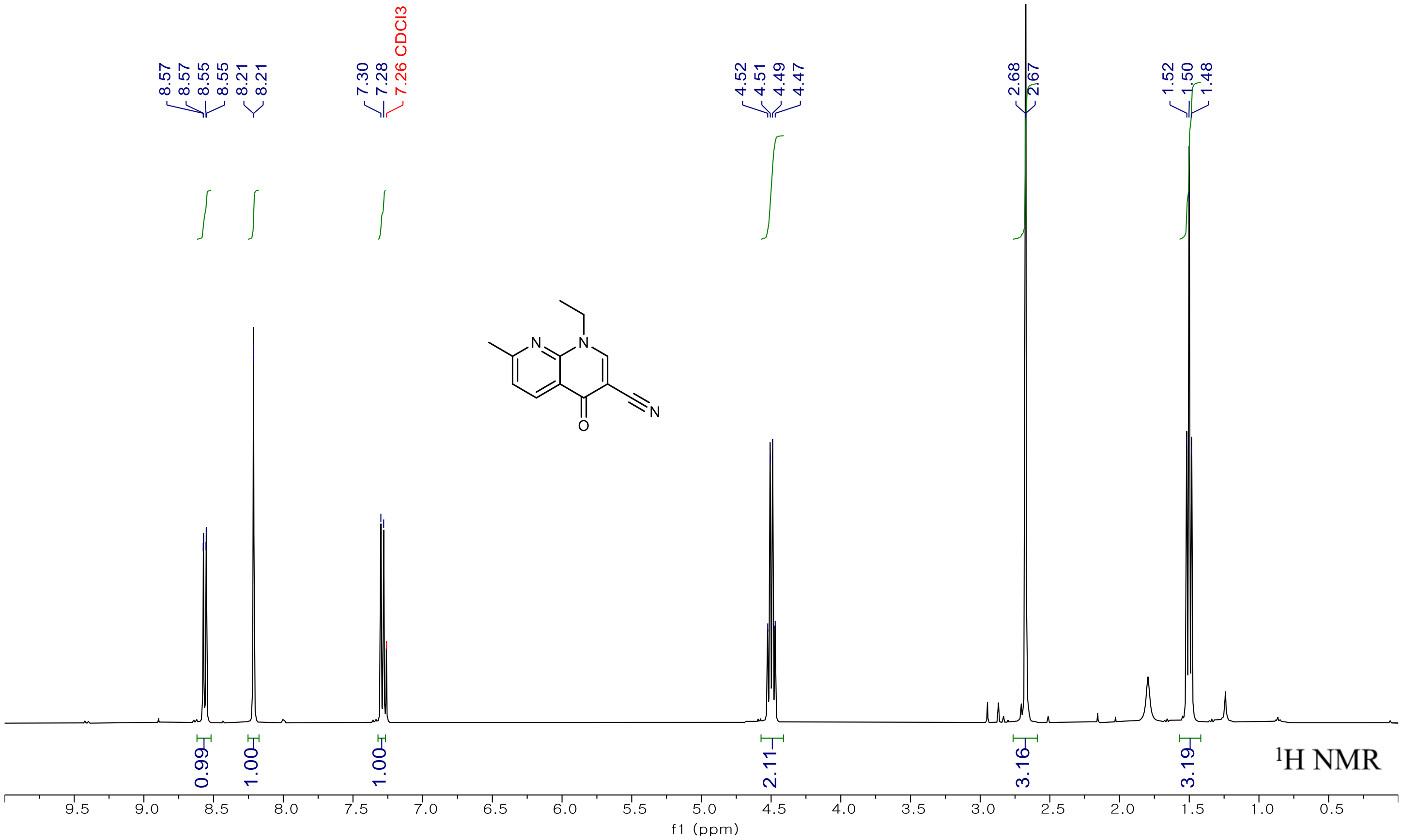


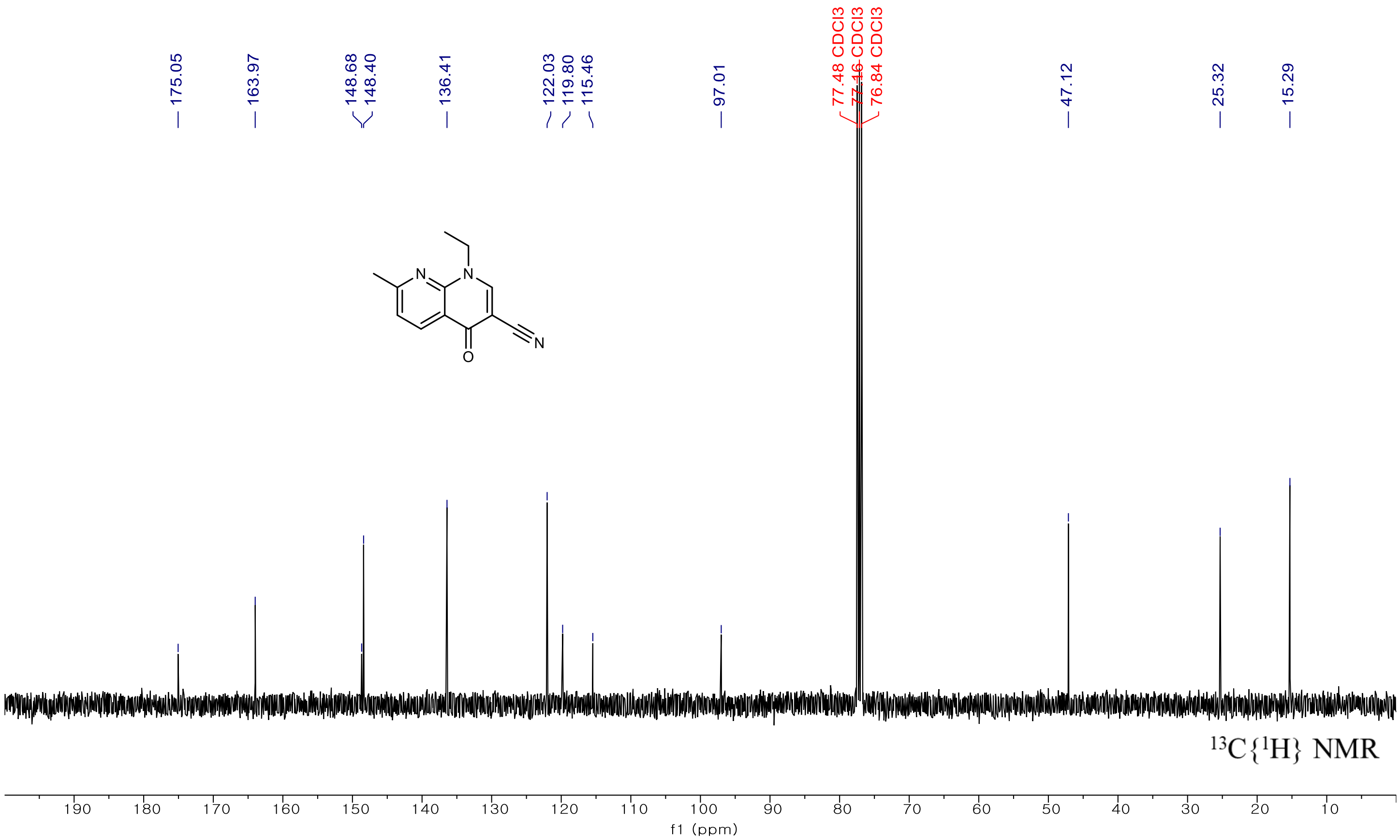

5-(2,5-Dimethylphenoxy)-2,2-dimethylpentanenitrile (**4ab**)

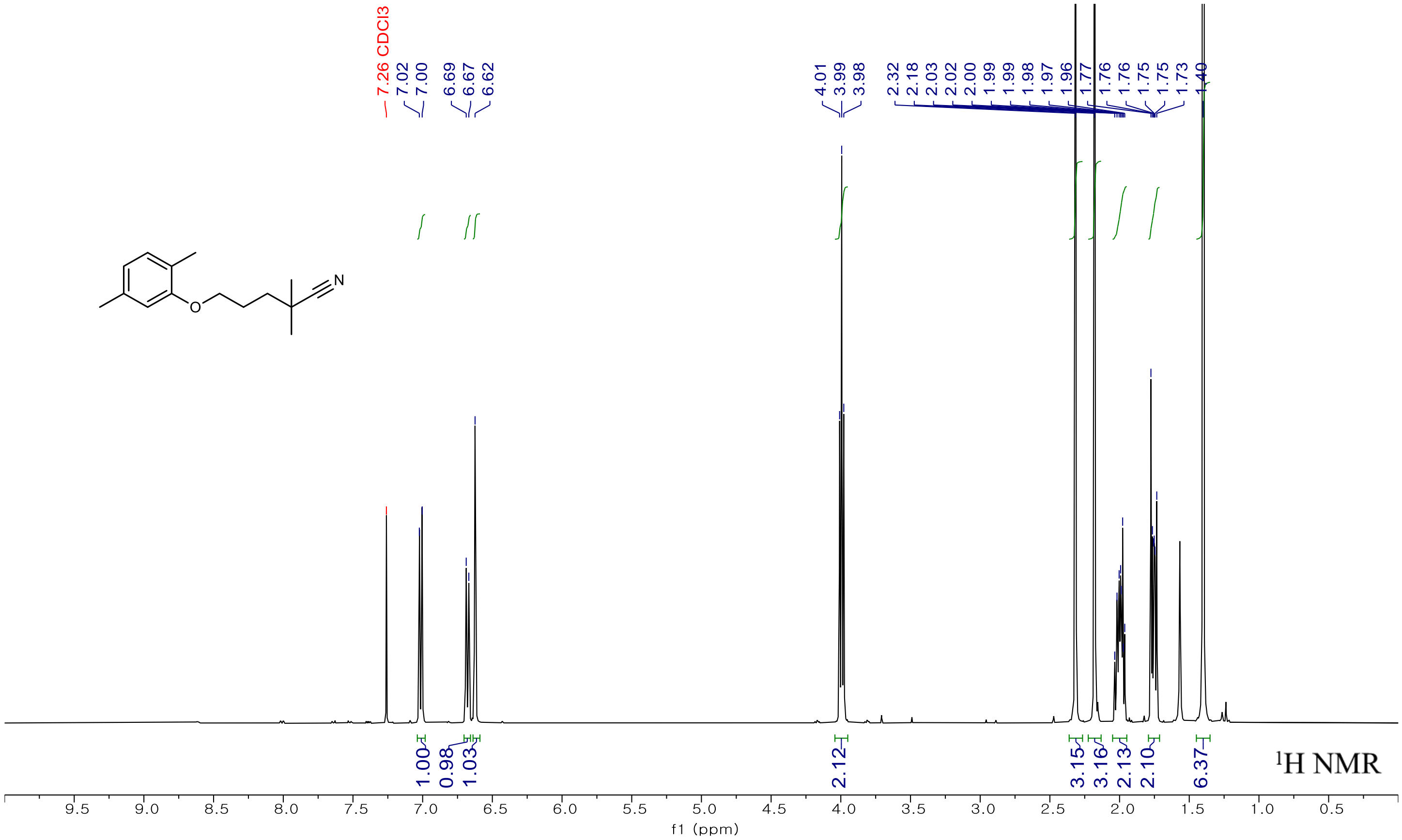

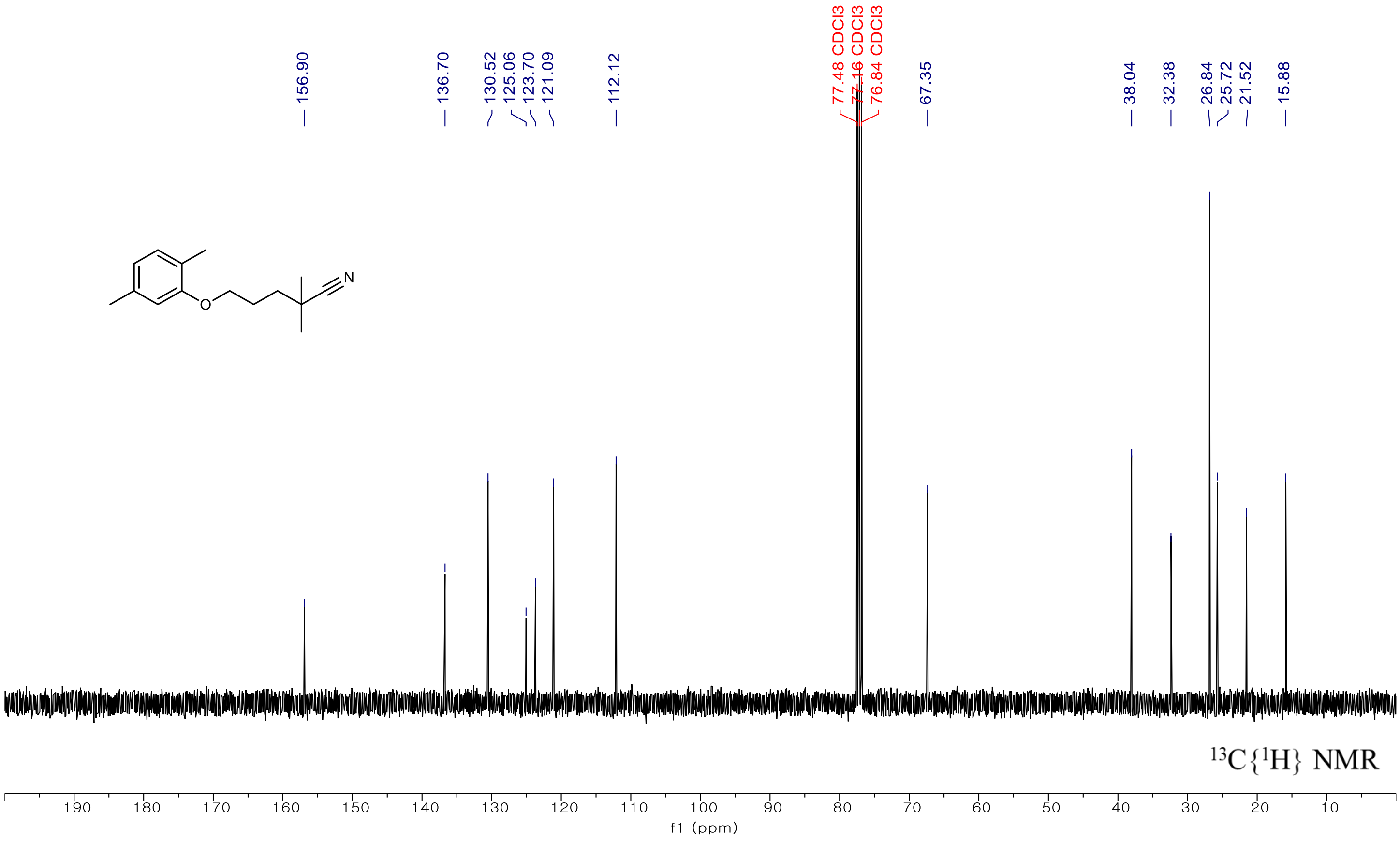

(*S*)-2-(4-Isobutylphenyl)propanenitrile (**4ac**)

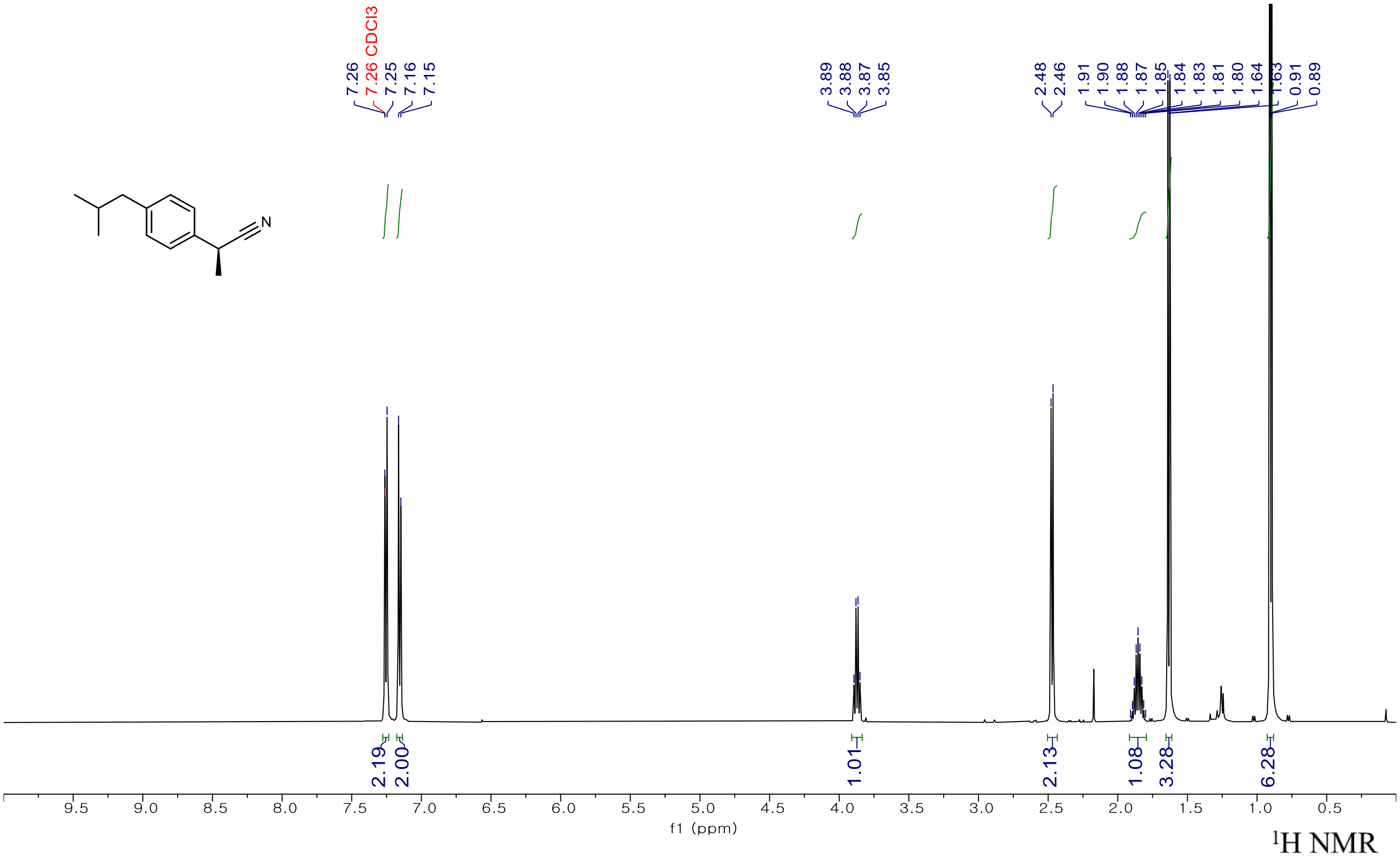


$^{1}$H NMR

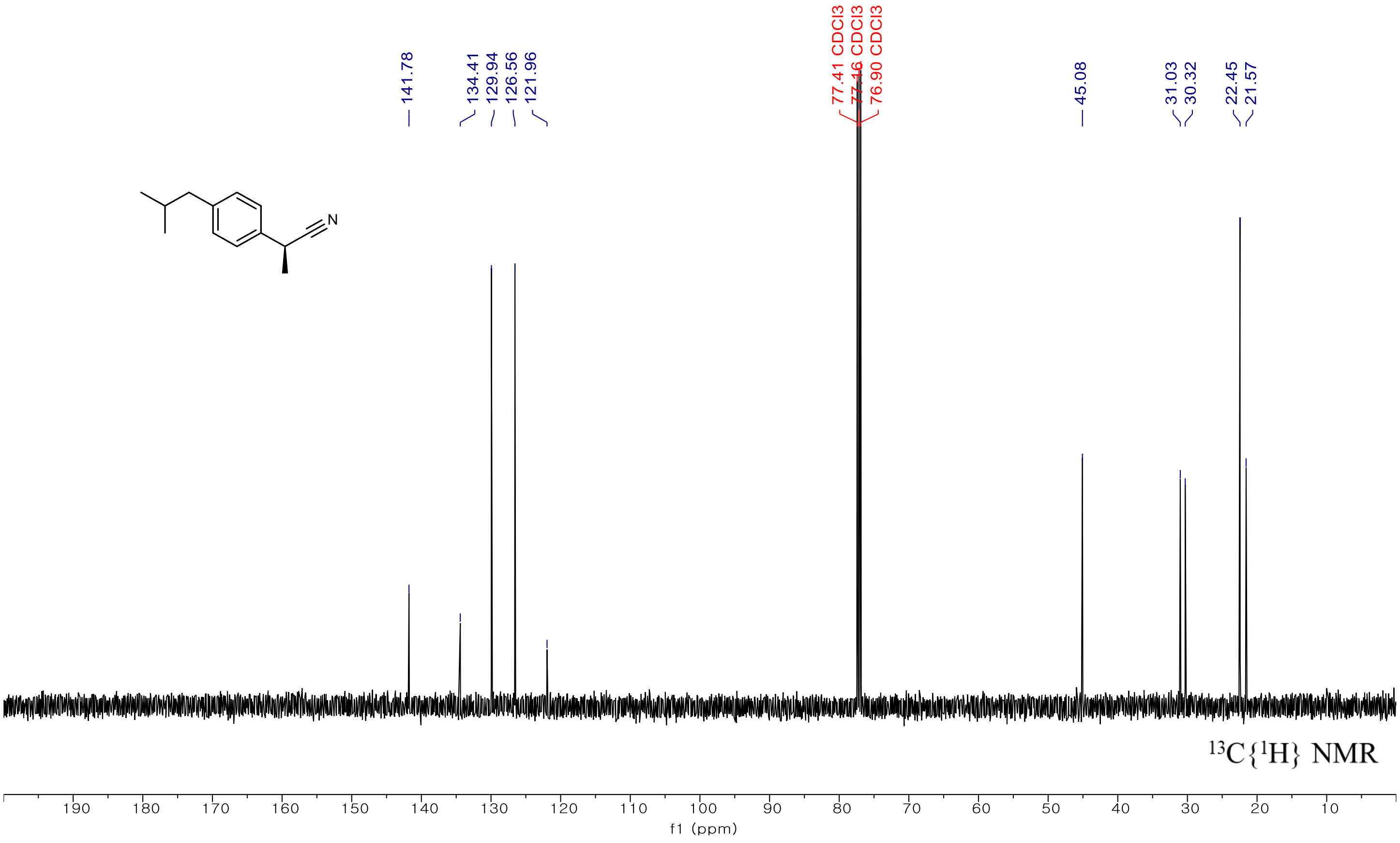


$^{13}$C{$^{1}$H} NMR

2-[1-(4-Chlorobenzoyl)-5-methoxy-2-methyl-1*H*-indol-3-yl]acetonitrile (**4ad**)

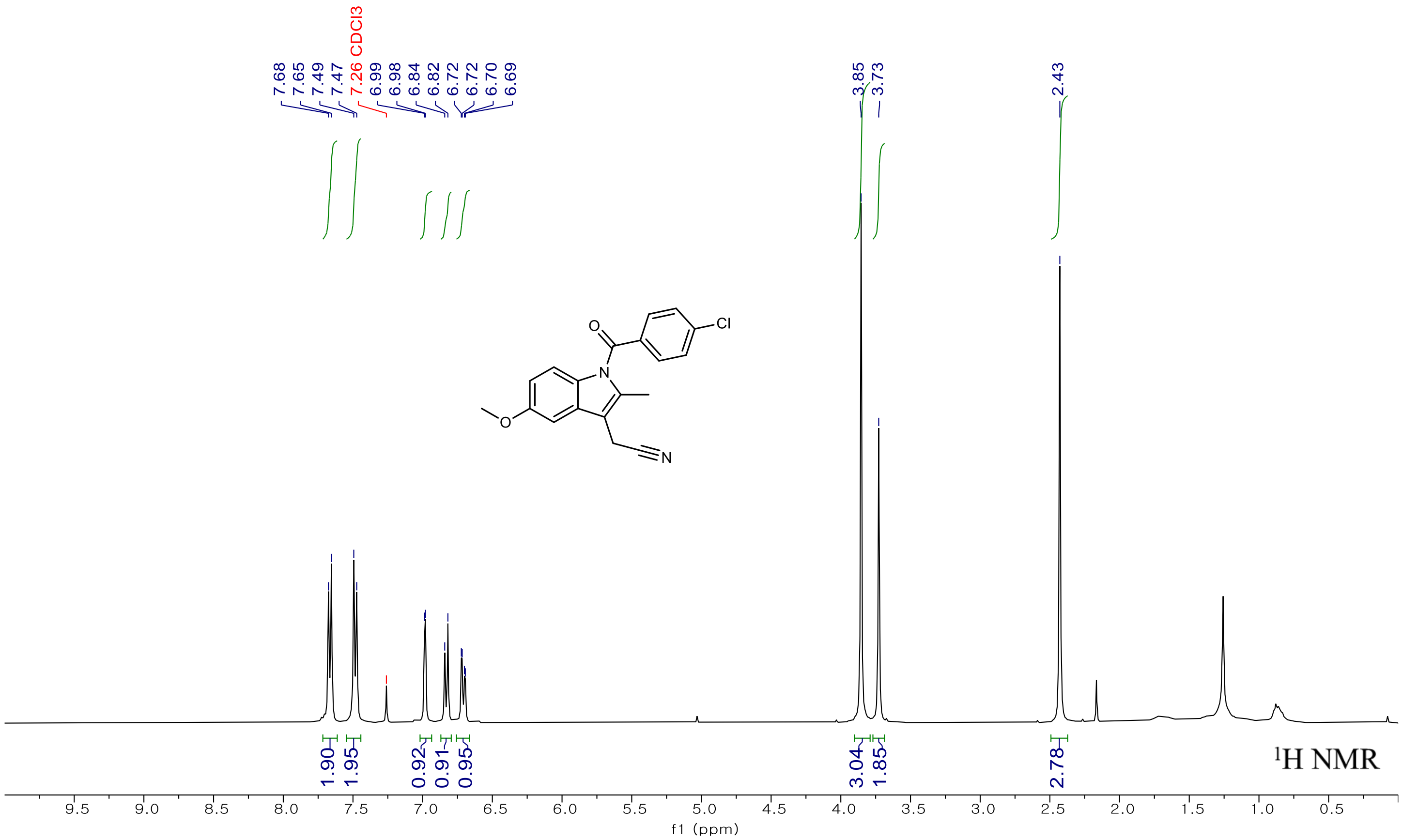


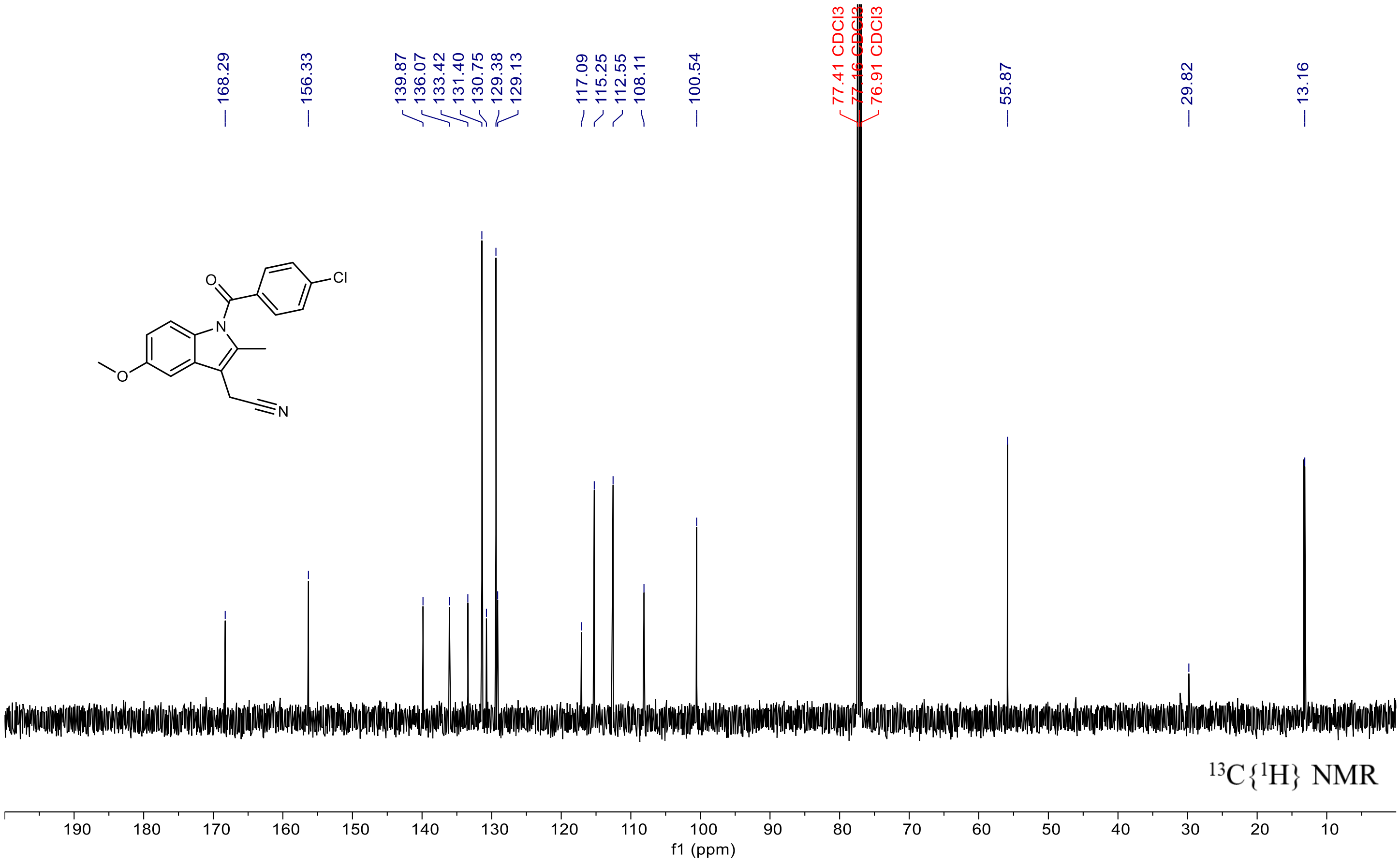

4-([1,1'-Biphenyl]-4-yl)-4-oxobutanenitrile (**4ae**)

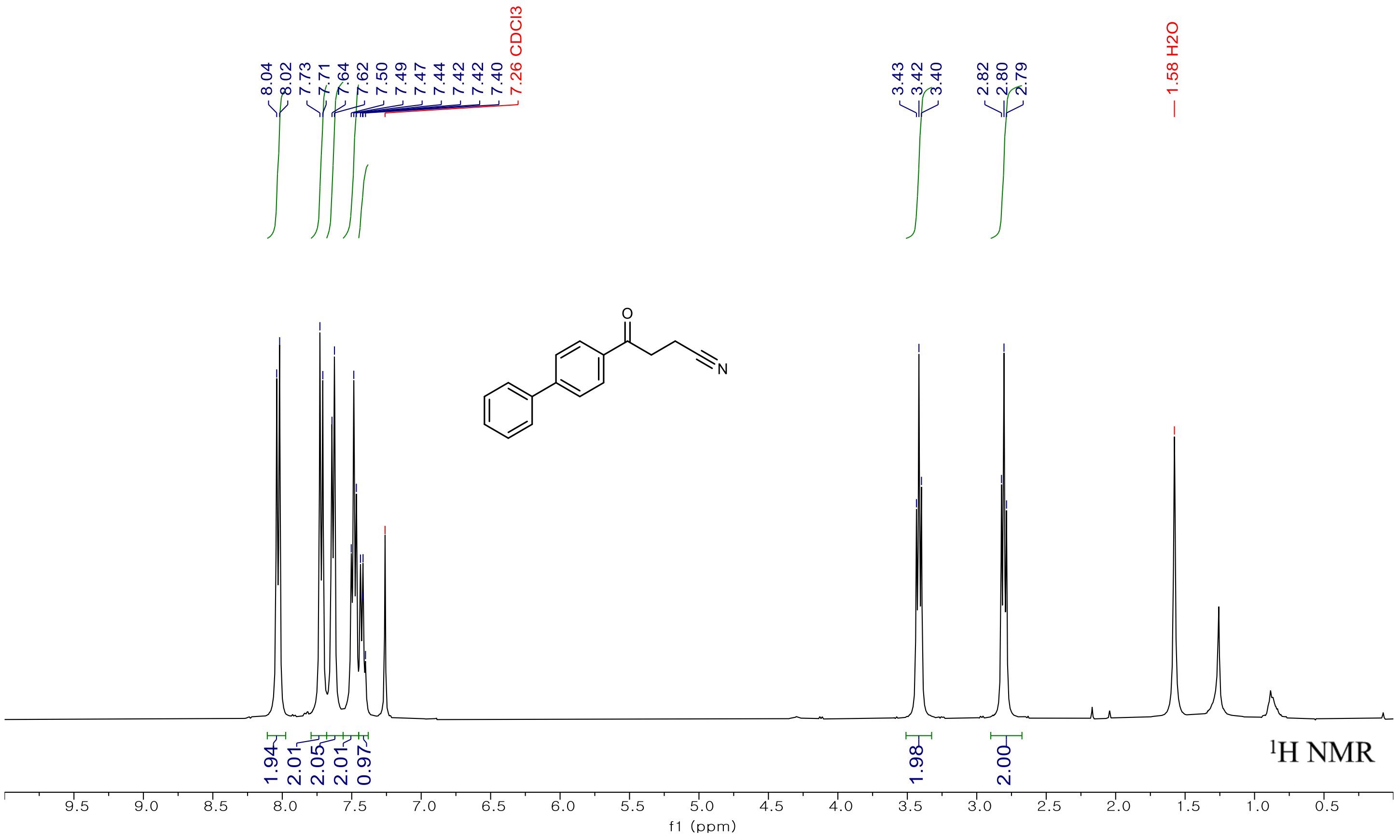


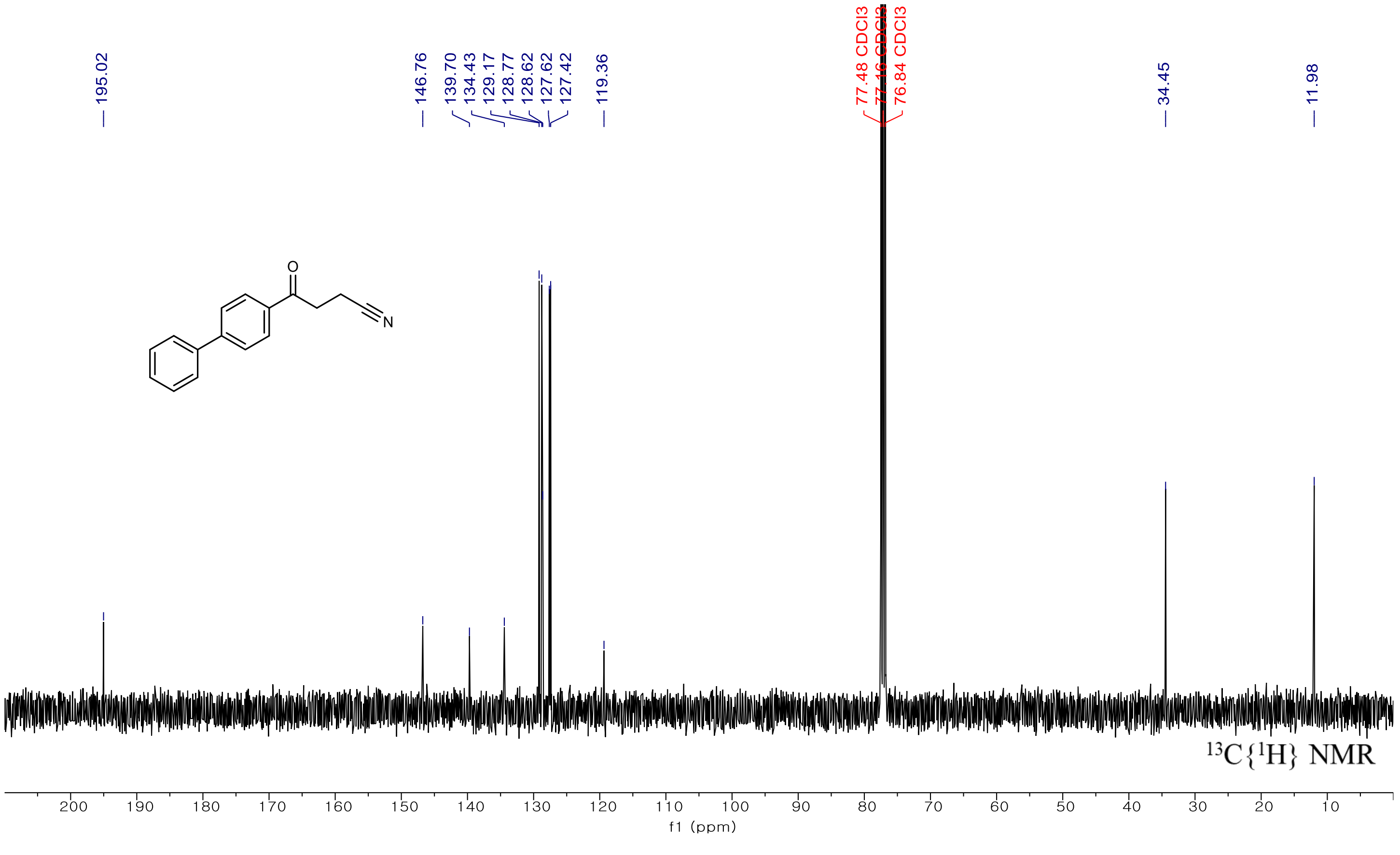

2-(10-Oxo-10,11-dihydrodibenzo[*b*,*f*]thiepin-3-yl)propanenitrile (**4af**)

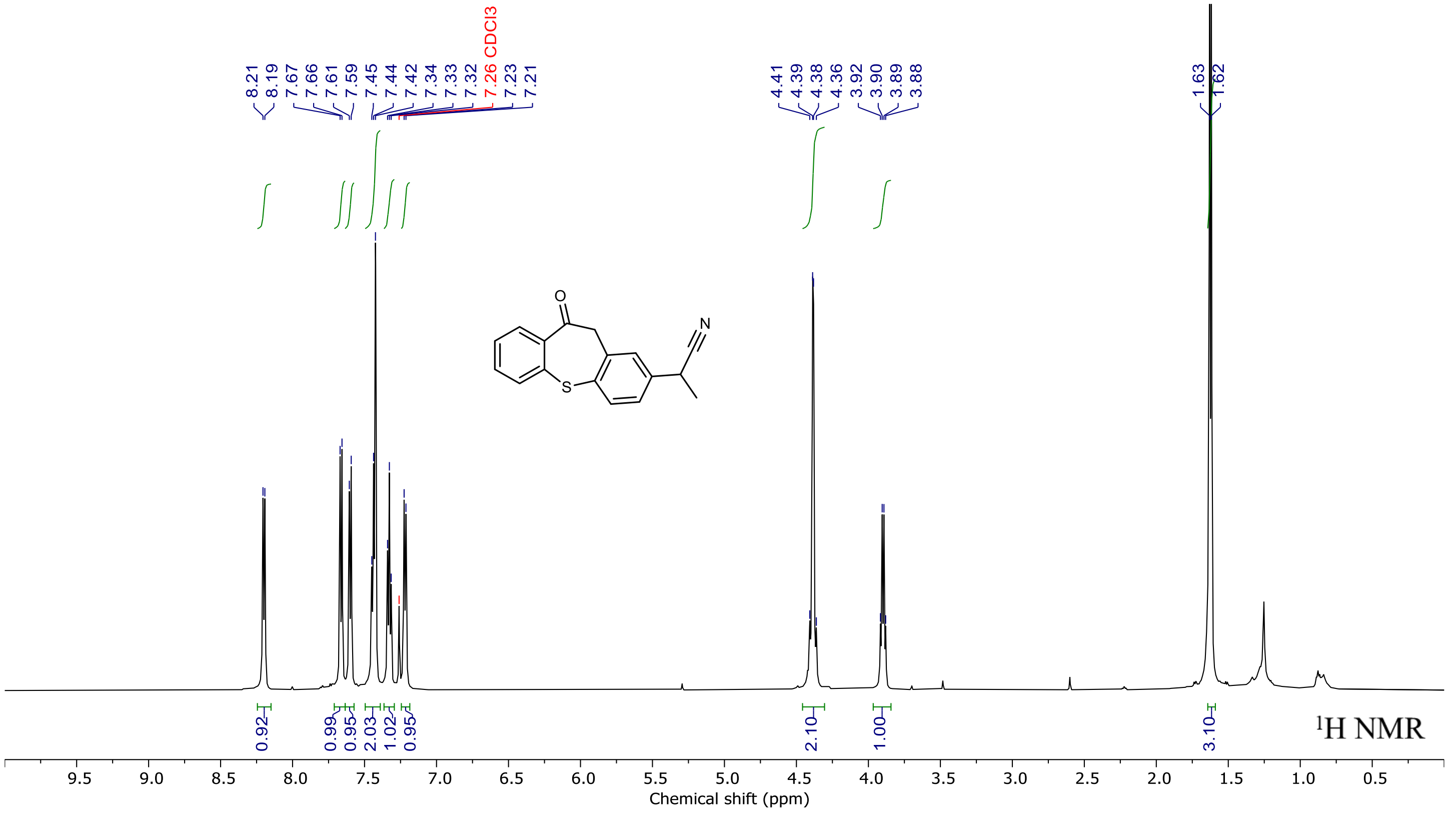


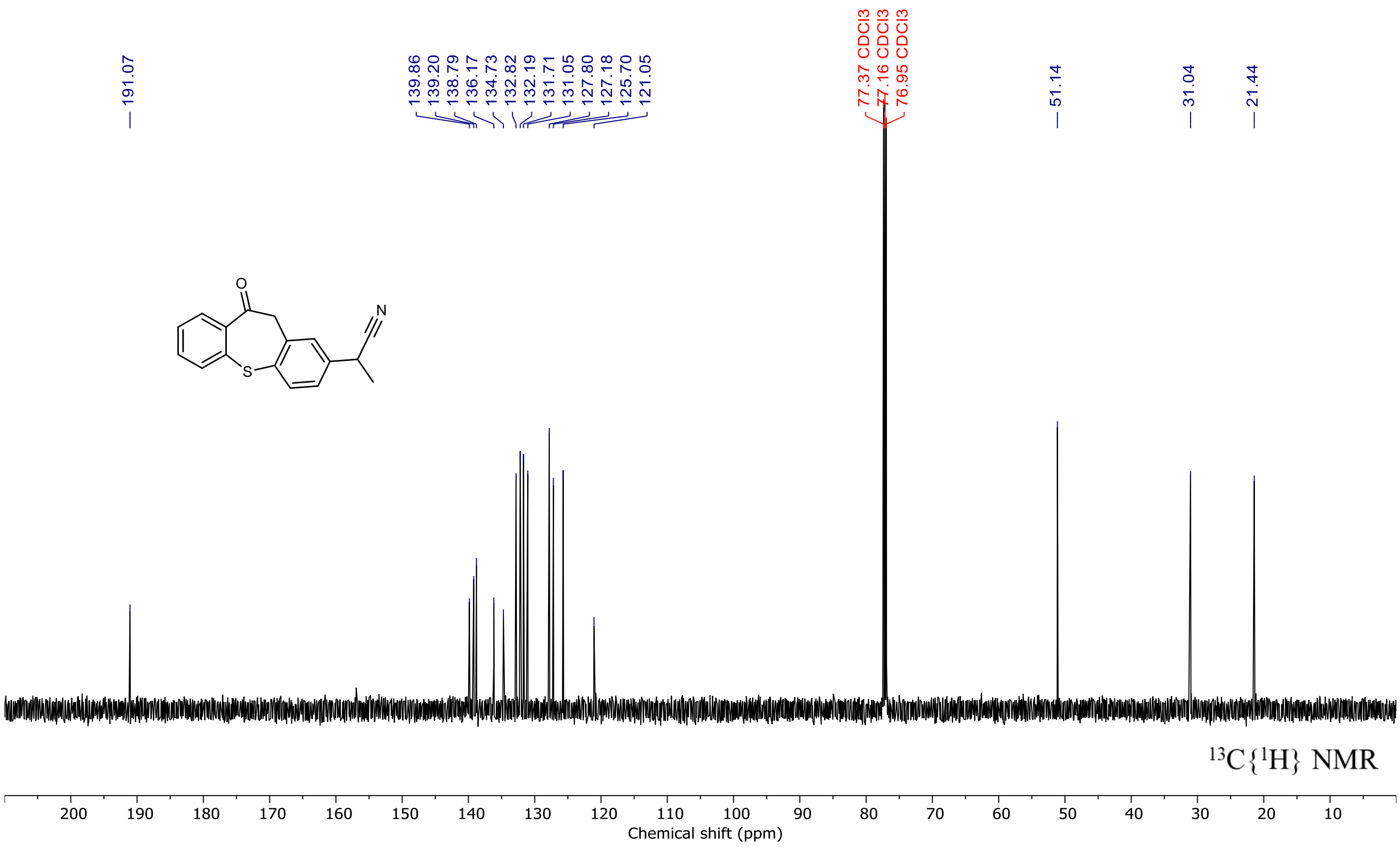

(*R*)-5-(1,2-Dithiolan-3-yl)pentanenitrile (**4ag**)

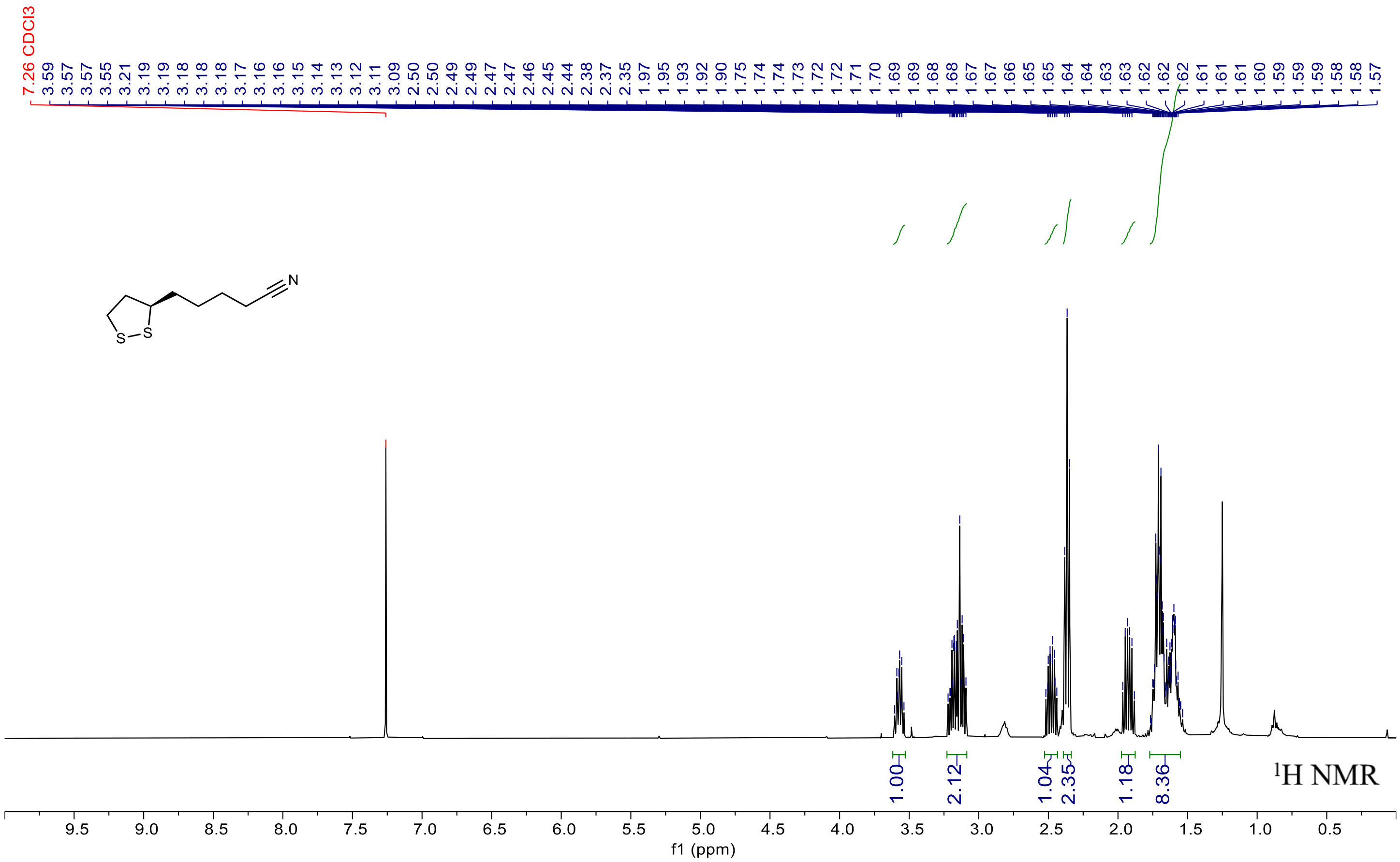


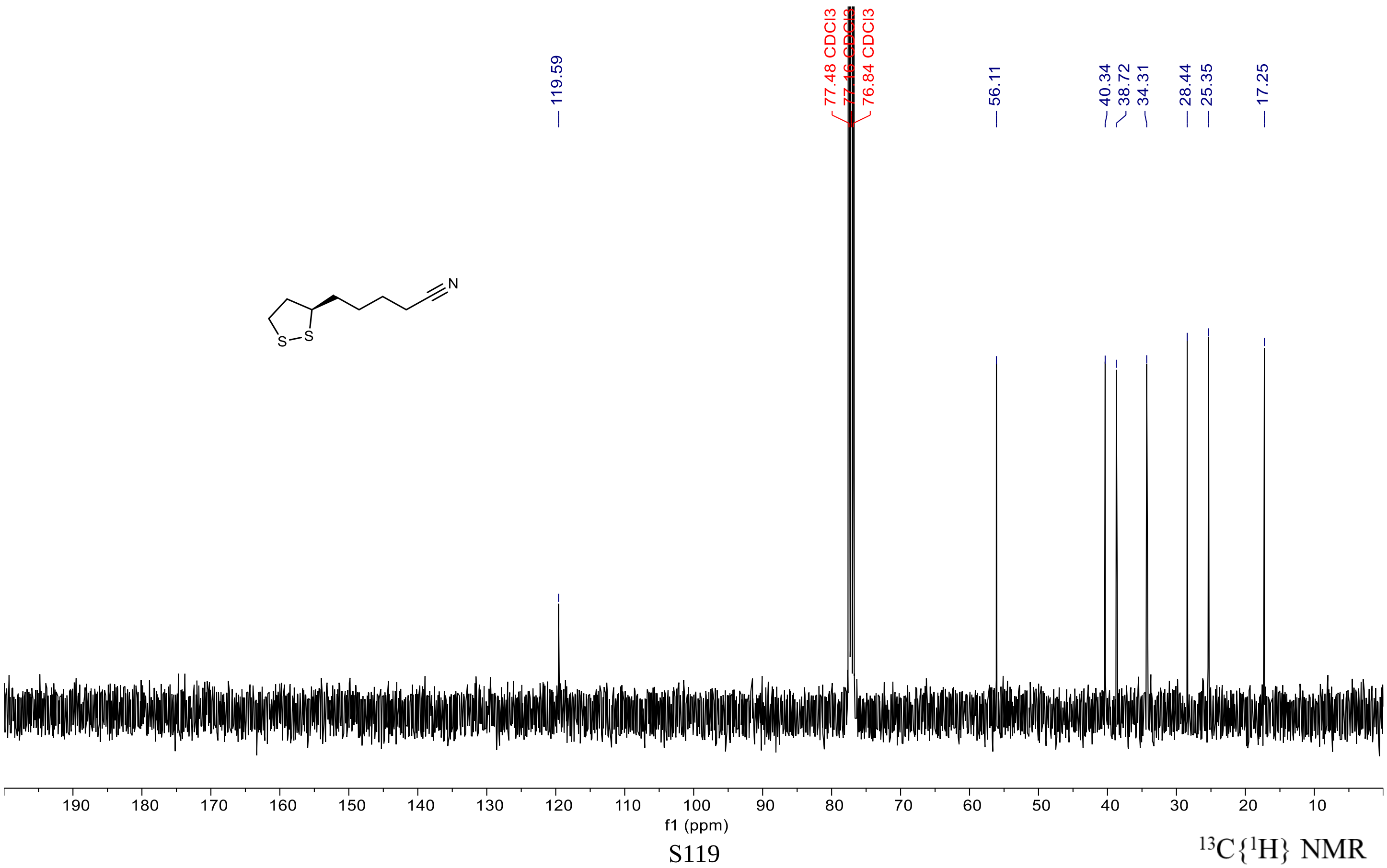


$^{13}C\{^{1}H\}$ NMR

5-[(3a*S*,4*S*,6a*R*)-2-oxohexahydro-1*H*-thieno[3,4-d]imidazol-4-yl]pentanenitrile (**4ah**)

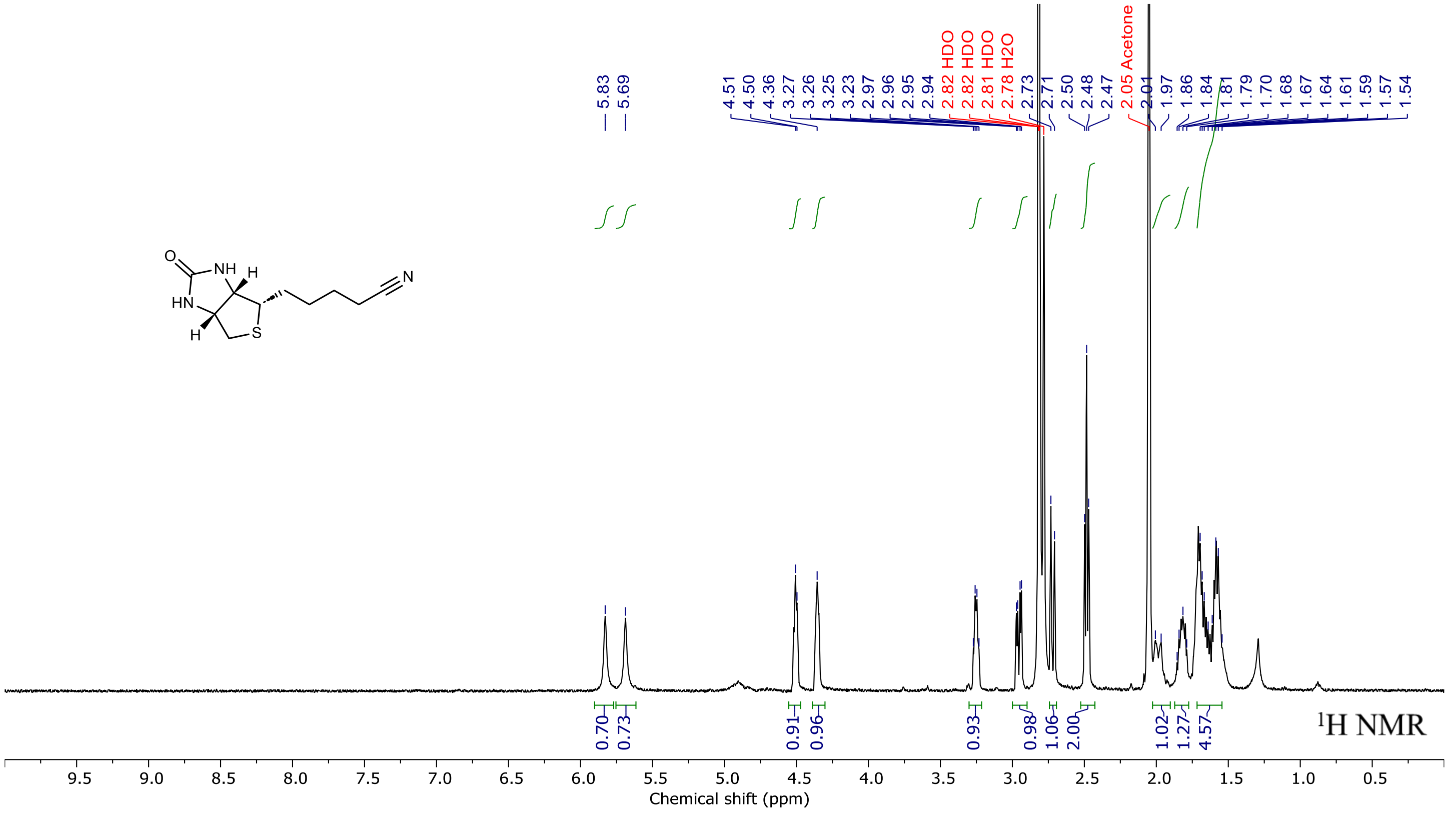


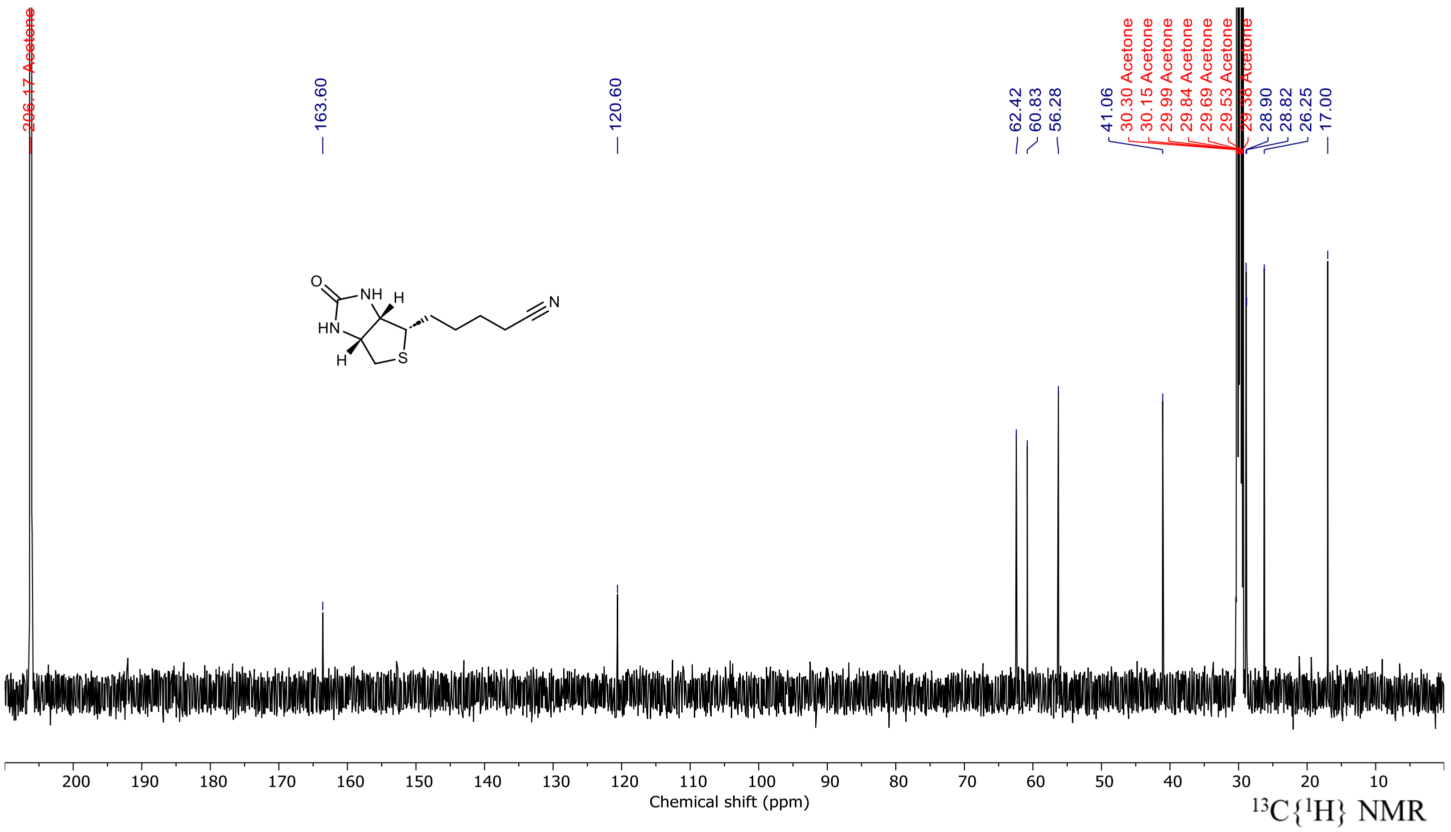

Supplement: Supplementary file 1 — Supporting File 1: advs75144‐sup‐0001‐SuppMat.pdf. [file ADVS-13-e75144-s002.pdf]
